# Chronic treatment with D2-antagonist haloperidol leads to inhibitory/excitatory imbalance in striatal D1-neurons

**DOI:** 10.1038/s41398-023-02609-w

**Published:** 2023-10-06

**Authors:** Cátia Santa, Diana Rodrigues, Joana F. Coelho, Sandra I. Anjo, Vera M. Mendes, Diogo Bessa-Neto, Michael J. Dunn, David Cotter, Graça Baltazar, Patrícia Monteiro, Bruno Manadas

**Affiliations:** 1 CNC-Center for Neuroscience and Cell Biology, University of Coimbra, 3004-504 Coimbra, Portugal; 2 III - Institute of Interdisciplinary Research, University of Coimbra, 3030-789 Coimbra, Portugal; 3 Life and Health Sciences Research Institute (ICVS), School of Medicine, University of Minho, Braga, Portugal; 4 ICVS/3B’s-PT Government Associate Laboratory, Braga/Guimaraes, Portugal; 5 Faculty of Medicine, University of Coimbra, Coimbra, Portugal; 6 CICS-UBI - Health Sciences Research Centre, University of Beira Interior, Covilhã, Portugal; 7 Proteome Research Centre, UCD Conway Institute of Biomolecular and Biomedical Research, School of Medicine, and Medical Sciences, University College Dublin, Dublin, Ireland; 8 RCSI Psychiatry, Royal College of Surgeons in Ireland, Education and Research Centre Beaumont, Dublin, Ireland; 9 Department of Biomedicine, Faculty of Medicine, University of Porto, 4200-319 Porto, Portugal

**Keywords:** Schizophrenia, Molecular neuroscience

## Abstract

Striatal dysfunction has been implicated in the pathophysiology of schizophrenia, a disorder characterized by positive symptoms such as hallucinations and delusions. Haloperidol is a typical antipsychotic medication used in the treatment of schizophrenia that is known to antagonize dopamine D2 receptors, which are abundantly expressed in the striatum. However, haloperidol’s delayed therapeutic effect also suggests a mechanism of action that may go beyond the acute blocking of D2 receptors. Here, we performed proteomic analysis of striatum brain tissue and found more than 400 proteins significantly altered after 30 days of chronic haloperidol treatment in mice, namely proteins involved in glutamatergic and GABAergic synaptic transmission. Cell-type specific electrophysiological recordings further revealed that haloperidol not only reduces the excitability of striatal medium spiny neurons expressing dopamine D2 receptors (D2-MSNs) but also affects D1-MSNs by increasing the ratio of inhibitory/excitatory synaptic transmission (I/E ratio) specifically onto D1-MSNs but not D2-MSNs. Therefore, we propose the slow remodeling of D1-MSNs as a mechanism mediating the delayed therapeutic effect of haloperidol over striatum circuits. Understanding how haloperidol exactly contributes to treating schizophrenia symptoms may help to improve therapeutic outcomes and elucidate the molecular underpinnings of this disorder.

## Introduction

Schizophrenia is a chronic brain disorder expressed as a combination of psychotic symptoms and cognitive and behavioral dysfunctions, that is among the world’s top 20 causes of years lost to disability [1]. Treatments for schizophrenia usually involve antipsychotic drugs that target D2 receptors, namely haloperidol, one of the first FDA-approved drugs that is still widely used [2]. Despite acting as a dopamine D2 receptor antagonist [3], haloperidol has been shown to interact with other receptors, such as dopamine D3 and D4 [4, 5], α-adrenergic receptor 1 [6], and to some extent 5HT_2A_ [7].

Although the classically described pathophysiology of schizophrenia involves midbrain dopamine projections to limbic regions of the brain, new findings suggest striatal dysfunction as a key mechanism contributing to the symptoms of schizophrenia [8]. The dorsal striatum is a brain region involved in motor, cognitive, and motivational functions [9–13], highly impacted by antipsychotic drugs. Indeed, it is well-recognized that chronic haloperidol administration has a significant impact not only on the volume of the dorsal striatum [14–18] and the number of striatal neurons [19, 20], but also at the synaptic level [21–25], both in humans and rodents.

Notwithstanding the evidence linking chronic haloperidol administration with several striatum changes, the exact neuronal mechanism by which haloperidol exerts its effects over the striatum remains elusive. Dopamine D2 receptors are highly expressed in a subset of striatal neurons, but despite the fact that D2 blockade can be achieved within hours after haloperidol administration [26], haloperidol’s onset of action is delayed by weeks. Unraveling the exact neuronal mechanism behind these delayed clinical effects of haloperidol in the striatum may not only pave the way to improving treatment outcomes but also shed light on the neurobiology of schizophrenia. Through the use of proteomic analysis and cell-type specific patch-clamp recordings in the dorsal striatum of mice treated with haloperidol, we demonstrate that chronic D2 receptor antagonism by haloperidol administration leads to a gradual remodeling of D1 neurons. This finding may help to explain the delayed therapeutic effects of haloperidol observed after 30 days of chronic administration in mice.

## Results

### Distinct striatal proteomic profile after chronic administration of haloperidol

**Fig. 1:**
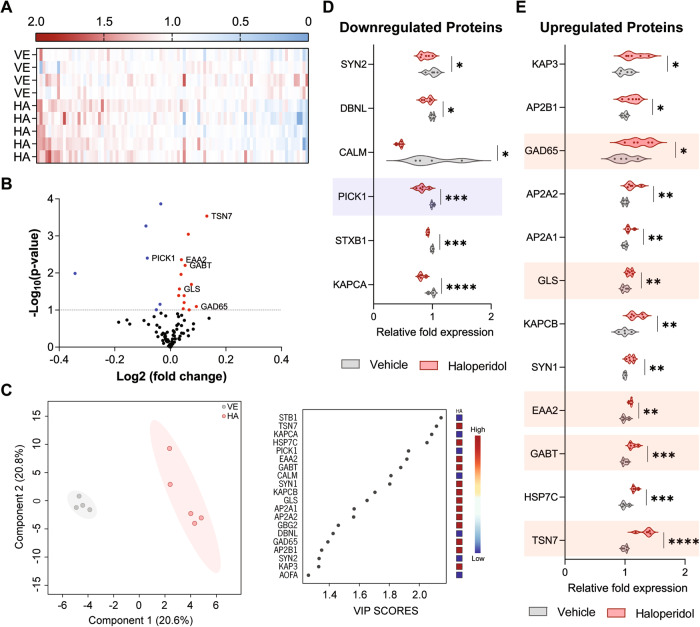
Proteomic analysis revealed synaptic modulation upon chronic exposure to haloperidol. **A** Heatmap view of 93 proteins (*x* axis) from the “neuronal system” family of the Reactome database. Protein fold enrichment is color-coded relative to the control average (blue: decreased expression; red: increased expression). The Y-axis represents biological replicates (Vehicles (VE): 1–4; Haloperidol (HA): 1–5). **B** Volcano plot of the 93 proteins with neuronal-related functions according to the Reactome database. Proteins statistically altered (*p* < 0.1) upon chronic exposure to haloperidol are color-coded (blue: decreased expression; red: increased expression). Proteins involved in glutamatergic and GABAergic synaptic transmission are highlighted. **C** Supervised multivariate analysis (PLS-DA) with the representation of the first two components accounting for 58.7% of the variability in the dataset (left panel). Variable importance in projection (VIP) score plot for the top 20 most important proteins identified by PLS-DA analysis (right panel). Fold enrichment of each protein in the haloperidol group is color-coded relative to the control average (blue: decreased expression; red: increased expression). **D** Violin plots of the significantly downregulated neuronal proteins in haloperidol-treated mice. Proteins involved in glutamatergic and GABAergic synaptic transmission are highlighted. **E** Violin plots of the significantly upregulated neuronal proteins in haloperidol-treated mice. Proteins involved in glutamatergic and GABAergic synaptic transmission are highlighted. Welch’s unpaired *t* test for **D**, **E**; *p < 0.1, **p < 0.05, ***p < 0.01, ****p < 0.001. Statistical details are shown in Supplementary Table S2.

To better understand the molecular impact of long-term haloperidol administration on striatal circuits, we started by injecting mice with haloperidol or vehicle for 30 consecutive days (Supplementary Fig. S1). After that period, brain samples were extracted from all mice to perform an unbiased proteomic screening in the striatum. A total of 1482 striatal proteins were quantified (Supplementary Table S1 and S3) and subsequently grouped based on their biological function by using the online platform Reactome [27]. We then focused our analysis on the 93 proteins that were involved in neuronal pathways (Fig. 1A, B, Supplementary Fig. S2). Supervised multivariate analysis of these proteins revealed a clear separation between the two experimental groups (haloperidol vs vehicle) and identified the 20 proteins that contributed most to the groups’ separation (those with the highest VIPs scores) (Fig. 1C). Six of those 20 corresponded to significantly altered proteins involved in glutamatergic and GABAergic synaptic transmission (highlighted in Fig. 1D, E, Supplementary Fig. S3). Specifically, we observed a downregulation of the PRKCA-binding protein (PICK1) (Fig. 1D), and upregulation of tetraspanin-7 (TSN7), excitatory amino acid transporter 2 (EAA2), glutaminase kidney isoform (GLSK), glutamic acid decarboxylase 2 (GAD2), and 4-aminobutyrate aminotransferase (GABT) (Fig. 1E), in haloperidol-treated mice. Given the physiological role of these proteins, which are particularly important for the synthesis and recycling/transport of glutamate and GABA neurotransmitters, and internalization of the AMPA receptors (depicted in Supplementary Fig. S3), our findings strongly suggest that chronic administration of haloperidol has a profound effect in the modulation of GABAergic and glutamatergic synaptic transmission (i.e. inhibitory and excitatory synaptic transmission) in the dorsal striatum. Moreover, several proteins involved in regulating neuronal excitability were also found to be altered, namely SCN2B (regulatory subunit of voltage-gated sodium channels), AT2B4 (plasma membrane calcium-transporting ATPase 4), and AT1A1, AT1A2, and AT1A3 (three sodium/potassium-transporting ATPase subunits) (Supplementary Fig. S4).

### Chronic treatment with haloperidol impairs excitatory synaptic transmission onto D2-MSNs, but not D1-MSNs

**Fig. 2:**
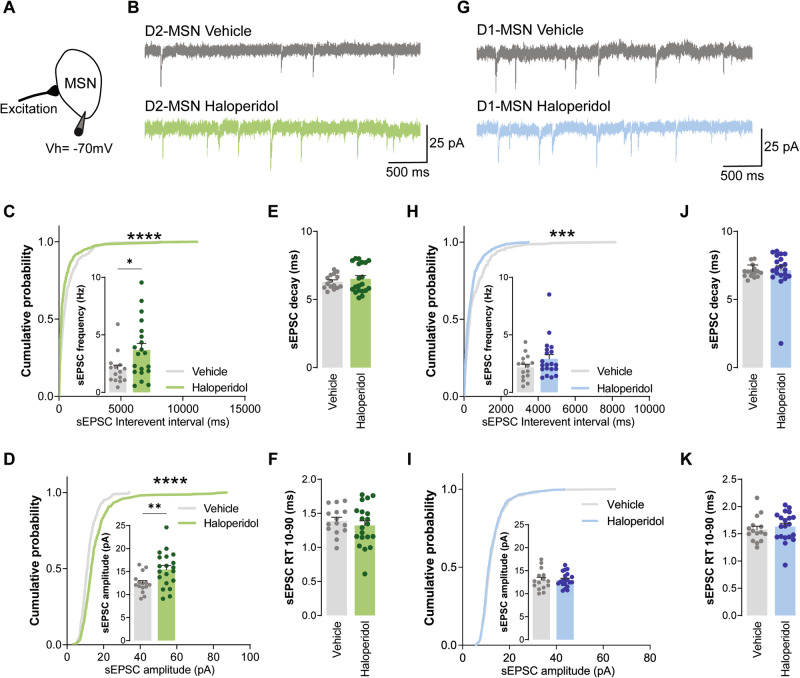
Chronic administration of haloperidol increases excitatory synaptic transmission onto D2-MSNs, but not D1-MSNs. **A** Illustration of the protocol used to record sEPSC. **B**, **G** Representative traces of sEPSC obtained from D2-MSNs and D1-MSNs, respectively. **C**, **H** Cumulative distribution of sEPSC inter-event intervals in vehicle (*n* = 15 cells) or haloperidol-treated (*n* = 20 cells) D2- and D1-MSNs, respectively. Inset shows average sEPSC frequency. **D**, **I** Cumulative distribution of sEPSC amplitudes in vehicle (*n* = 15 cells) or haloperidol-treated (*n* = 20 cells) D2- and D1-MSNs, respectively. Inset shows average sEPSC amplitude. **E**, **J** Average decay time of sEPSC recorded from D2- and D1-MSNs of vehicle (*n* = 15 cells) or haloperidol-treated (*n* = 20 cells) mice. **F**, **K** Average 10–90% rise time (RT) of sEPSC recorded from D2- and D1-MSNs of vehicle (*n* = 15 cells) or haloperidol-treated (*n* = 20 cells) mice. Welch’s unpaired *t* test for all panels except for cumulative distributions (Kolmogorov–Smirnov test for cumulatives). *p < 0.05, **p < 0.01, ***p < 0.001, ****p < 0.0001. Statistical details are shown in Supplementary Table S2.

Nearly 90% of striatal neurons are medium spiny neurons (MSNs) that can be categorized into D1- and D2-MSNs, depending on whether they express dopamine D_1_ or dopamine D_2_ receptors, respectively [28]. These distinct cell populations project different basal ganglia targets forming two interrelated and behaviorally competing circuit pathways: the direct pathway composed of D1-MSNs neurons; and the indirect pathway composed of D2-MSNs neurons [28]. Our proteomics data pointed to several synaptic alterations that occur in the striatum upon 30 days of haloperidol administration, but it does not provide cell-type specific information on whether these molecular changes stem either from D1 or D2-MSNs, or both. Thus, to further scrutinize how chronic haloperidol administration might affect these two distinct neuronal populations, we performed cell-type specific electrophysiology recordings in acute brain slices from BAC transgenic mice chronically administrated with haloperidol for 30 consecutive days. These transgenic mice express the fluorescent protein eGFP in D2-MSNs, allowing targeted whole-cell recordings from both striatal populations: fluorescent D2-MSNs and nonfluorescent putative D1-MSNs. We start by assessing the intrinsic excitability of both D1- and D2-MSNs. Similarly to what has been reported after 15 days of chronic haloperidol administration [29], 30 days of haloperidol treatment had no effect on D1-MSNs intrinsic excitability but decreased the excitability of D2-MSNs (Supplementary Fig. S5 and S6). Next, to evaluate whether chronic haloperidol administration impacts glutamatergic synaptic transmission, as suggested by our proteomics data, we recorded AMPA-mediated synaptic transmission from both D1- and D2- MSNs (Fig. 2). Compared to control animals, D2-MSNs from haloperidol-treated mice showed increased frequency and amplitude of spontaneous excitatory postsynaptic transmission (sEPSC) (Fig. 2C, D). In contrast, D1-MSNs showed similar synaptic rate coding (average frequency and amplitude; small inset in Fig. 2H, I) but distinct synaptic temporal coding (Fig. 2H cumulative curves). sEPSC decay kinetics and 10–90% rise time were not different between groups, both in D1 and D2 neurons (Fig. 2E, F, J, K). Taken together, our data suggest that chronic haloperidol administration specifically affects glutamatergic AMPAR-mediated synaptic transmission onto D2-MSNs, without strongly affecting excitatory synaptic inputs onto D1-MSNs.

### Chronic administration of haloperidol increases inhibitory inputs over both D1 and D2-MSNs

**Fig. 3:**
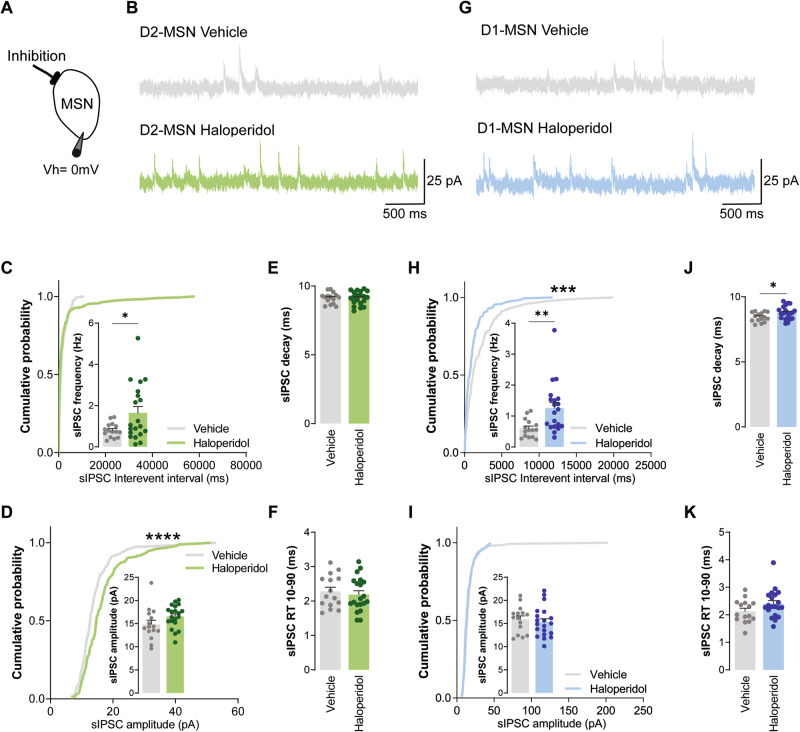
Chronic administration of haloperidol increases inhibitory synaptic transmission onto both D2- and D1-MSNs. **A** Illustration of the protocol used to record sIPSC. **B**, **G** Representative traces of sIPSC obtained from D2-MSNs and D1-MSNs, respectively. **C**, **H** Cumulative distribution of sIPSC inter-event intervals in vehicle (*n* = 15 cells) or haloperidol-treated (*n* = 20 cells) D2- and D1-MSNs, respectively. Inset shows average sIPSC frequency. **D**, **I** Cumulative distribution of sIPSC amplitudes in vehicle (*n* = 15 cells) or haloperidol-treated (*n* = 20 cells) D2- and D1-MSNs, respectively. Inset shows average sIPSC amplitude. **E**, **J** Average decay time of sIPSC recorded from D2- and D1-MSNs of vehicle (*n* = 15 cells) or haloperidol-treated (*n* = 20 cells) mice. **F**, **K** Average 10–90% rise time (RT) of sIPSC recorded from D2- and D1-MSNs of vehicle (*n* = 15 cells) or haloperidol-treated (*n* = 20 cells) mice. Welch’s unpaired *t* test for all panels except for cumulative distributions (Kolmogorov–Smirnov test for cumulatives). *p < 0.05, **p < 0.01, ****p < 0.0001. Statistical details are shown in Supplementary Table S2.

**Fig. 4:**
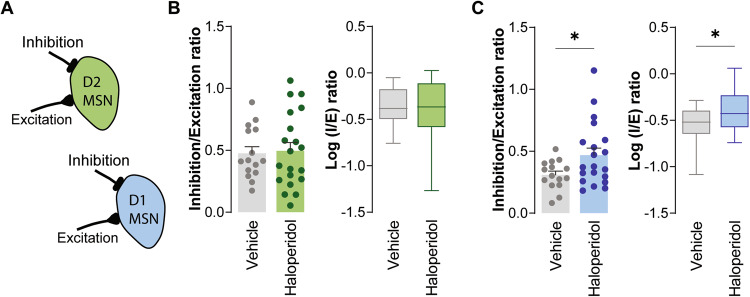
Chronic administration of haloperidol leads to increased I/E ratio in D1- but not D2-MSNs. **A** Illustration of I/E ratio in D1- (blue) and D2-MSNs (green). **B**, **C** Average IPSC/EPSC (I/E) frequency ratio (left) and the logarithm of the ratio between IPSC/EPSC frequencies (right) in vehicle and haloperidol-treated D2- and D1-MSNs, respectively. Welch’s unpaired *t* test for all panels. *p < 0.05. Statistical details are shown in Supplementary Table S2.

Next, we assessed inhibitory synaptic transmission in haloperidol-treated mice by recording spontaneous inhibitory postsynaptic currents (sIPSC; Fig. 3). Surprisingly, we found heightened sIPSC frequency in both D2- and D1-MSNs, after 30 days of treatment with haloperidol (Fig. 3C, H). sIPSC amplitude was unchanged in all conditions (Fig. 3D, I). In line with our proteomics data, where we did not observe alterations in GABA receptor subunits between groups, no major changes were observed in the decay or 10–90 RT kinetics of sIPSC (Fig. 3E, F, J, K). The decreased inhibitory synaptic currents onto D1-MSNs, coupled with the absence of alterations in excitatory synaptic currents, can lead to an imbalance of the synaptic inhibitory-excitatory ratio (known as I/E ratio). Since we recorded both AMPA and GABA receptor-mediated currents in the same cell, it was possible to directly address this possibility at the individual cell level (Fig. 4A). Spontaneous AMPA receptor-mediated currents were recorded at a holding potential of −70 mV (Fig. 2A), followed by recordings at 0 mV to isolate GABA receptor-mediated currents (Fig. 3A). We observed a reduced I/E ratio in D1-MSNs from mice treated for 30 days with haloperidol (Fig. 4C). No differences were observed in the I/E ratio of D2-MSNs from haloperidol-treated mice (Fig. 4B). These results reveal a cell-type-specific imbalance of synaptic excitation and inhibition onto D1-MSNs of the dorsal striatum caused by chronic administration of haloperidol.

## Discussion

Early models of schizophrenia proposed striatal dysfunctions as being central to psychotic symptoms [8]. Not surprisingly, the striatal dopaminergic system is a common target of most antipsychotic drugs. Indeed, the striatum presents a high density of dopamine D_2_ receptors, which are strongly antagonized by conventional antipsychotic drugs, such as haloperidol [30, 31]. However, the delayed therapeutic effect of antipsychotic drugs calls into question the precise synaptic mechanism by which they exert their effects [32]. Although several studies consistently highlight that chronic treatment with haloperidol profoundly impacts striatal synaptic plasticity and glutamatergic synaptic transmission [22–25, 29, 33–37], it has been unclear how it impacts the striatal plasticity of inhibitory circuits. Accordingly, several human studies have consistently reported elevated levels of glutamate in the associative striatum of patients with schizophrenia [38, 39], as well as in those at familial high risk for schizophrenia [40]. In addition, non-responders to antipsychotic drugs have been found to have higher glutamate levels than responders both before and after treatment, indicating a potential link between modulation of this neurotransmitter and the dopamine-blocking effects of antipsychotics in the striatum [41]. Furthermore, antipsychotic-naïve patients at a clinically high risk for psychosis have been found to exhibit higher levels of both glutamate and GABA in the dorsal caudate [42]. Collectively, these studies strongly suggest dysregulation of glutamate and GABA levels, and consequent I/E imbalance, as a possible mechanism in the pathophysiology of psychosis in schizophrenia. Our own findings are consistent with these observations and demonstrate, for the first time, that chronic blocking of D2 receptors with haloperidol can modulate not only glutamatergic but also GABAergic synaptic transmission in the striatum. These findings underscore the importance of our study and highlight the potential therapeutic benefits of modulating both glutamatergic and GABAergic synaptic transmission in the striatum, which could lead to more effective treatments for schizophrenia.

Despite not being a selective antagonist, haloperidol presents a high affinity for dopamine D_2_ receptors [43], highly expressed in the indirect pathway neurons (D2-MSNs) of basal ganglia [28]. Not surprisingly, chronic administration of haloperidol has been reported to have a strong effect on these neurons [29, 37, 44–46]. Concordantly, our data revealed that chronic administration of haloperidol increases glutamatergic synaptic transmission, specifically onto D2-MSNs, without affecting D1-MSNs. These findings are supported by our proteomics data, where we observed an orchestrated altered protein expression that points to an increase of glutamate and glutamatergic receptors at synapses. Specifically, we observed an upregulation of TSN7, EAA2, and GLS and a downregulation of PICK1 in haloperidol-treated mice. The TSN7 and PICK1 are two proteins that play opposite roles in GluA2 trafficking. Whereas PICK1 binds to GluA2 and promotes its internalization, TSN7 competes with GluA2 to bind PICK1 and, therefore, stabilizes GluA2 at the synaptic surface [47]. Thus, an increase of TSN7 accompanied by a decrease of PICK1 points for a possible enrichment of GluA2 at the synapses, an AMPA receptor subunit that has a clear tendency to be increased in haloperidol-treated mice (Supplementary Table S1). Furthermore, the detected changes in EAA2 and GLSK expression suggest an increase in glutamate uptake and recycling from neuronal terminals. Altogether, our data support the hypothesis that haloperidol facilitates glutamatergic synaptic transmission, specifically onto D2-MSNs.

Reduced glutamate-mediated synaptic transmission has been recognized to mediate some schizophrenia symptoms [48]. However, recent data from *post-mortem* tissue, animal models, and human studies have also linked GABAergic synaptic transmission alterations to the pathobiology of schizophrenia [49]. Interestingly, our proteomic data showed that the chronic treatment with haloperidol induces upregulation of GAD2 in the striatum, an enzyme that regulates GABA (γ-aminobutyric acid) synthesis and whose mRNA and protein expression, along with GAD67, has been consistently shown to be reduced in several brain regions of patients suffering from schizophrenia and related disorders [50–59]. Additionally, an increase in GAD2 expression may also be responsible for the observed increase in GABA levels in animal models and schizophrenia patients treated with typical antipsychotic drugs [60, 61]. Furthermore, we also observed an upregulation of GABT, an enzyme that in GABAergic neurons is responsible for the catabolism of GABA. As a result, glutamate is produced directly from the conversion of GABA to semialdehyde succinate, and further from the metabolism of semialdehyde succinate to succinate that enters the citric acid cycle. The resulting glutamate from these reactions can be converted back to GABA by GAD2. In line with these observations, our ex vivo electrophysiology recordings reveal for the first time increased GABAergic synaptic transmission onto both D1- and D2-MSNs caused by chronic haloperidol administration, corroborating previous studies suggesting that haloperidol decreases the activity of the GABAergic striatonigral system [62]. The increase of GABAergic synaptic transmission onto D1-MSNs changes their I/E balance, which may provide a mechanistic explanation for the delayed positive effects of chronic haloperidol treatment in which it compensates for the disruption of excitatory/inhibitory activity reported in schizophrenia [63, 64]. These findings are in accordance with previous studies showing that using haloperidol in adjuvant with selective modulators of the GABAergic system (such as benzodiazepines) improves the therapeutic effect of haloperidol in the acute phase of symptom exacerbation or relapse. Moreover, there is evidence that this co-adjuvant treatment may not be beneficial in the long term, and the discontinuation of benzodiazepine in antipsychotic-treated patients is advised after the acute phase [65, 66].

Understanding and identifying the precise mechanisms by which haloperidol exerts its beneficial effects represents an important step towards paving the way for more effective therapeutics that can ultimately improve patients’ lives. Here, we propose a mechanism by which chronic administration of haloperidol leads to a slow remodeling of the synaptic connectivity of striatal D1-MSNs due to the modulation of GABAergic synaptic transmission.

## Materials and methods

### Animals

All animal procedures were approved by local authorities Direção Geral de Alimentação e Veterinária (DGAV) and performed in accordance with European Community Council Directives (2010/63/EU) and the Portuguese law DL N° 113/2013 for the care and use of laboratory animals. Mice were group-housed under a 12 h light: 12 h dark cycle (8:00 a.m., lights on; 8:00 p.m., lights off) and an ambient temperature of 20–22 °C with *ad libitum* access to food and water.

Mice were randomly assigned into experimental groups without a systematic randomization approach. Bacterial artificial chromosome (BAC) transgenic mice expressing eGFP under *Drd2* promotor, and C57BL/6 mice were randomly assigned to the haloperidol-treated group or control group for electrophysiological (VE: 1 male and 2 females; HA: 3 males and 1 female) and proteomic (VE: 4 males; HA: 5 males) characterization, respectively. Haloperidol was dissolved in ethanol to a final concentration of 10 mM and injected at 0.665 mM in 0.13% HCl and 0.9% NaCl. At 11 weeks old, mice started receiving 100 µL of 0.9% NaCl by intraperitoneal (IP) injection for habituation purposes during 7 days. Afterwards, haloperidol (1 mg/Kg) or vehicle was administrated by IP injection for 30 consecutive days. This haloperidol dosage is extensively used throughout the literature [29, 67] and was selected to recapitulate dosages used in humans [29]. Bodyweight was measured and used to calculate the required drug for each consecutive day.

### Proteomics

Proteomic analysis was performed from striatal samples. Briefly, 24 h after the last drug/vehicle administration, mice were decapitated following Ketamine/Xylazine (87.5 mg/Kg and 12.5 mg/Kg, respectively) anesthesia, and the striatum were rapidly macrodissected and frozen in liquid nitrogen in 500 mM TEAB (Triethylammonium bicarbonate buffer, Sigma). All the buffers used for subproteome fractionation were supplemented with phosphatases (PhosSTOP cocktail pills, Roche) and proteases (cOmplete, EDTA-free Protease Inhibitor Cocktail, Roche) inhibitors. The striatal tissue was homogenized in 850 μL of ice-cold buffer (0.05 M Tris, pH 7.4), at 4 °C by ultrasonication (Vibra Cell 130 watts, Sonics) with a 2 mm probe for 30 sec at 40% amplitude followed by 30 sec at 50% both with on/off cycles of 1 sec. Homogenates were centrifuged for 5 min at 5000 × *g* at 4 °C. The supernatants were kept at 4 °C, and the pellets were homogenized in 500 μL of 0.05 M Tris and subsequently centrifuged for 5 min at 5000 × *g* at 4 °C. The supernatants of both centrifugations were joined to form one replicate.

For the library generation, 150 μL from each group replicate were pooled together, and 250 μL Tris was added. The pooled samples were then ultracentrifuged for 1 h at 144,000 × *g* at 4 °C [68]. The supernatant was stored at 4 °C, and the pellet was dissolved in 500 μL 0.5 M TEAB by ultrasonication (Vibra Cell 130 watts, Sonics) with a 2 mm probe with 30 sec cycles at 40−50% amplitude.

Protein precipitation was performed from all samples by adding six volumes of cold acetone [69] and incubating for at least 20 min at −80 °C. Proteins from individual samples were subsequently recovered by centrifugation for 20 min at 20,000 × *g* at 4 °C, whereas soluble and membrane-enriched fractions were centrifuged for 20 min at 4100 × *g* at 4 °C. The protein pellets were resuspended in 2× Laemmli Sample Buffer (5% glycerol, 1.7% SDS, 100 mM DTT, and bromophenol blue in 50 mM Tris buffer at pH 6.8) aided by ultrasonication using a cup horn device (Vibra-cell 750 watt, Sonics) at 40% amplitude for 2 min [70]. Protein quantification was assessed using the 2-D Quant kit (GE Healthcare), according to the manufacturer’s instructions.

Short GeLC-SWATH-MS (Sequential Window Acquisition of all Theoretical Mass Spectra) was previously described by us [69, 71], with minor modifications. Briefly, 50 µg of each sample was subjected to in-gel digestion after a partial SDS-PAGE run using precast gel (4–20% Mini-Protean® TGX™ Gel, Bio-Rad). LC-MS information was acquired in two different acquisition modes: information-dependent acquisition (IDA) of the pooled samples and SWATH (Sequential Windowed data independent Acquisition of the Total High-resolution Mass Spectra) acquisition of each individual sample. Protein identification and library construction were performed using ProteinPilot™ (v5.0.1, Sciex), and the relative quantification was performed using SWATH™ processing plug-in for PeakView™ (v2.2, Sciex). The mass spectrometry proteomics data have been deposited to the ProteomeXchange Consortium via the PRIDE [72] partner repository with the dataset identifier PXD038471.

### Whole-cell patch-clamp recordings

Before brain slicing, mice were deeply anaesthetized with Avertin (tribromoethanol; 20 mg/mL; Sigma–Aldrich) with a dose of 0.5 mg/g body weight by IP injection, and perfused with an oxygenated (95% O_2_, 5% CO_2_) *N*-methyl-d-glucamine (NMDG)-based artificial cerebrospinal fluid (aCSF) solution containing (in mM): 92 NMDG, 2.5 KCl, 1.2 NaH_2_PO_4_, 30 NaHCO_3_, 20 HEPES, 25 glucose, 5 sodium ascorbate, 2 thiourea, 3 sodium pyruvate, 10 MgSO_4_.7H_2_O and 0.5 CaCl_2_.2H_2_O (7.2–7.4 pH and 300–310 mOsm/L). The brain was rapidly removed from the skull and coronal striatal slices of 300 µM were cut on a vibratome (Leica VT1000, Leica Microsystems) filled with oxygenated NMDG. The slices were then transferred to an auxiliary chamber in which they were kept at 32 °C for 11 min in oxygenated NMDG, and subsequently transferred to a holding chamber filled with oxygenated aCSF containing (in mM): 119 NaCl, 2.5 KCl, 1.2 NaH_2_PO_4_, 24 NaHCO_3_, 12.5 glucose, 2 MgSO_4_.7H_2_O and 2 CaCl_2_.2H_2_O (7.2–7.4 pH and 300–310 mOsm/L), to recover for at least 1 h at room temperature (RT). After recovering, slices were transferred to the recording chamber mounted on an Olympus BX51 upright fluorescent microscope equipped with IR-1000E camera (DAGE-MTI). All recordings were performed in the dorsolateral striatum, continually perfused with oxygenated aCSF at a rate of 2 mL min^−1^ at 33 °C. MSNs were selected based on their morphology and D2- and D1-MSNs were distinguished by the presence or absence of eGFP fluorescence, respectively.

Glutamatergic and GABAergic synaptic transmission was assessed in the same cell with borosilicate glass recording electrodes (2–5 MΩ; Science Products) filled with a cesium-based internal solution containing (in mM): 110 CsOH, 110 D-Gluconic acid, 15 KCl, 4 NaCl, 5 TEA-Cl, 20 HEPES, 0.2 EGTA, 5 Lidocaine N-ethyl chloride, 4 ATP and 0.3 GTP (7.2–7.4 pH and 300–310 mOsm/L). To monitor spontaneous glutamatergic synaptic transmission, MSNs were held at −70 mV. The holding was subsequently changed to 0 mV to record spontaneous GABAergic synaptic transmission. Fluctuations in current were used to determine spontaneous excitatory or inhibitory postsynaptic current (sEPSC or sIPSC, respectively) frequency, amplitude, decay, and 10–90 RT, as well as to calculate sIPSC/sEPSC frequency ratio. The effect of haloperidol in intrinsic properties was assessed as described in Monteiro et al. [73] using borosilicate glass recording electrodes (3–5 MΩ; Science Products) filed with a K-gluconate solution containing (in mM): 131 K-gluconate, 17.5 KCL, 9 NaCl, 1 MgCl_2_.6H_2_O, 10 HEPES, 1.1 EGTA, 2 MgATP and 0.2 Na_2_GTP (pH adjusted to 7.4 with KOH and osmolarity adjusted to ~300 mOsm L^–1^ with sucrose). Briefly, intrinsic properties were measured in response to a series of hyperpolarizing and depolarizing current injections (10 pA steps, starting at −150 pA). Voltage-current plots were constructed from the equilibrium potentials reached for different somatic current injections, and IF plots from the number of action potentials fired in response to each injected current. Rheobase current was defined as the first current step capable of eliciting one action potential. Input resistance was calculated with a −150 pA hyperpolarizing step from the resting membrane potential. Whole-cell capacitance was measured using a 5 mV, 200 ms step from −90 mV, with a Bessel filter of 10 kHz. A bi-exponential fit of membrane relaxation following the 5 mV depolarization was used to calculate the weighted tau and consequent whole-cell capacitance as described by Gertler et al. [74]. Inwardly rectifying currents present during a voltage ramp were also performed as described by Gertler et al. [74] where neurons were initially voltage-gated at −80 mV and then voltage ramps were applied from −150 to −30 mV at a rate of ±0.54 V ms^−1^ (Supplementary Fig. S5K and S6K). Current-voltage plots were determined by stepping cells from a holding potential of −70 mV to different voltages (10 mV steps, starting at −150 mV). Series resistance was monitored, and cells were discarded if they presented a series resistance higher than 25 MΩ). The bridge balance was adjusted for current-clamp recordings.

Electrophysiological recordings were performed using a MultiClamp 700B amplifier (Axon Instruments) and acquired using a Digidata 1440A digitizer (Axon instruments), filtered at 2 kHz, and digitized at 10 kHz. Analysis of intrinsic properties, sIPSCs, and sEPSCs was performed using pClamp (Clampfit; Axon Instruments) and Minianalysis software (Synaptosoft) by manually clicking on individual events.

### Statistical Analysis

Statistical analyses were performed using GraphPad Prism 9 software (GraphPad Software Inc.). Supervised multivariate analysis (PLS-DA) was performed using the online platform Metaboanalyst 5.0 [75]. The sample size was estimated based on recent literature in the same field. Data are plotted as mean ± SEM. Non-normal distributions were considered for all the data sets, regardless of variance and sample size. The variation within each sample was estimated to be at similar levels. For proteomics data analysis it was used a confidence level of 90%. Experimenters were blinded to the experimental groups when analyzing data. Further details on particular analyses are shown in Sup. Table S2 and figure legends.

### Supplementary information

Supplementary information

Supplementary Table S1

Supplementary Table S2

Supplementary Table S3
