# Supplementary material for: Chronic treatment with D2-antagonist haloperidol leads to inhibitory/excitatory imbalance in striatal D1-neurons
Source: Transl Psychiatry. 2023 Oct 6;13:312. doi: 10.1038/s41398-023-02609-w (PMC10558446; doi:10.1038/s41398-023-02609-w)
Supplement: Supplementary file 1 — Supplementary information [file 41398_2023_2609_MOESM1_ESM.pdf]

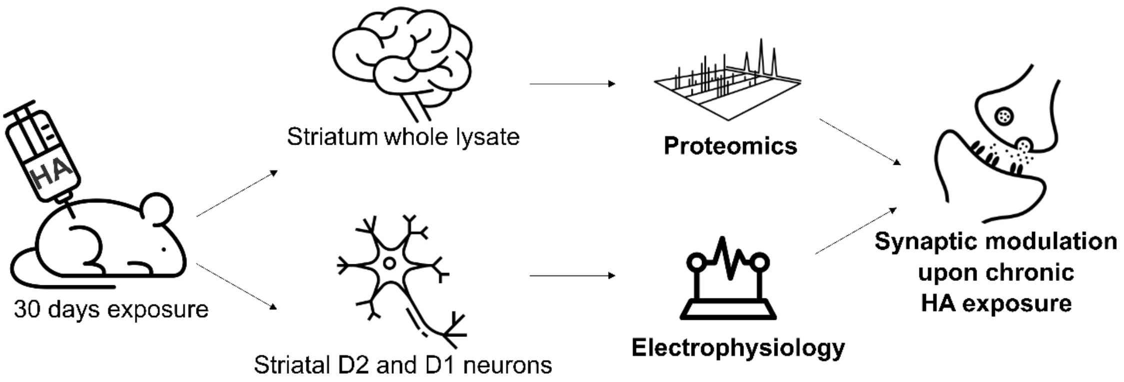

Figure S1 – General scheme of the approach adopted in the present study.

Mice were exposed to haloperidol or saline for 30 days, followed by a proteomics analysis of the striatum and a parallel electrophysiology analysis of D2 and D1 neurons in the striatum. All icons were obtained from the Noun Project.

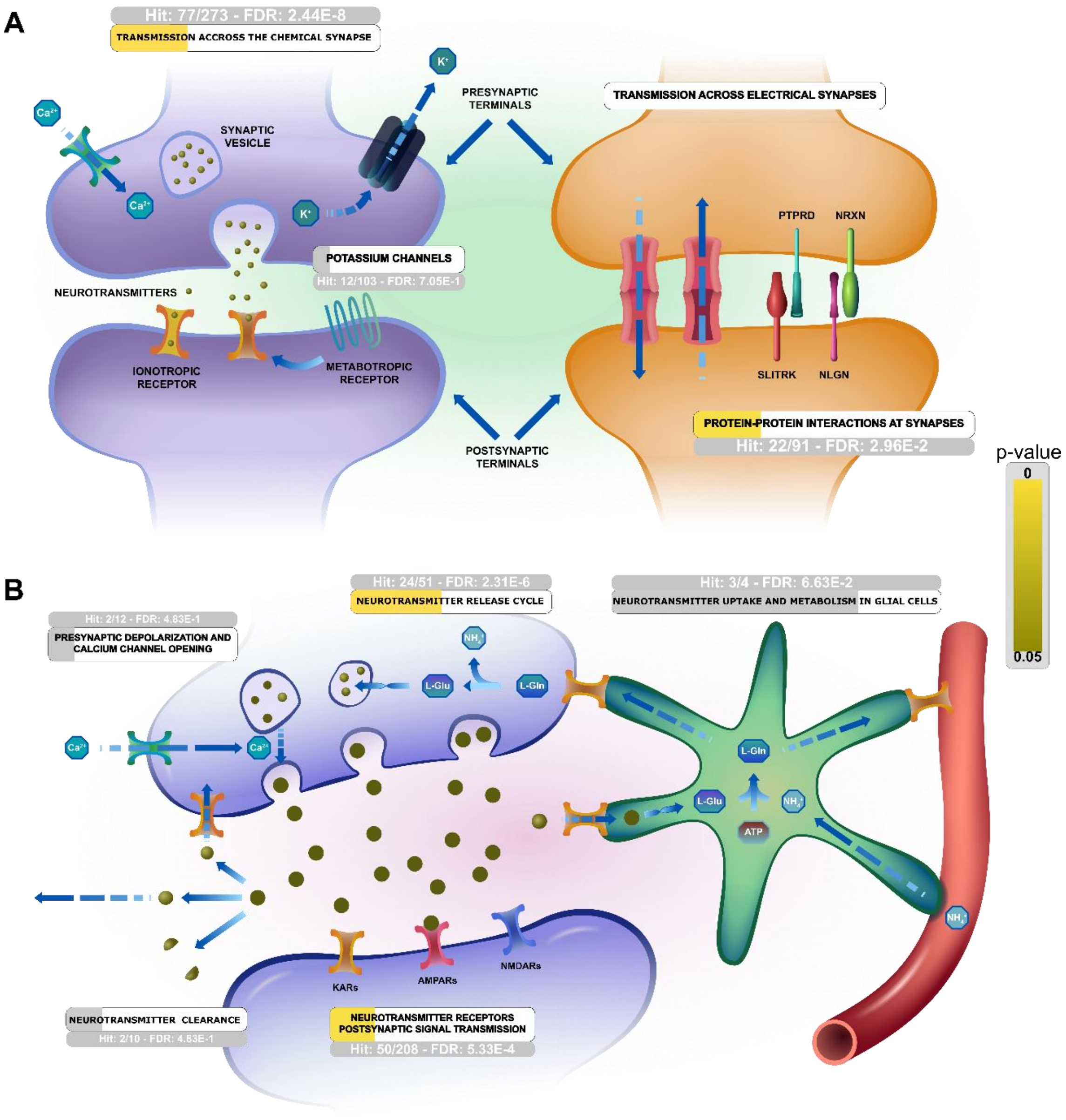

Figure S2 – Neuronal-related functions of the 1482 striatal proteins analysed with Reactome[1].

(**A**) Simplified representation of the neuronal system, with the representation of chemical and electrical synapses, where the proteins quantified in this dataset belong mainly to the chemical synapse. (**B**) Overview of the chemical synapse, where it is visible that many of the quantified proteins belong to the neurotransmitter release cycle and the postsynaptic signal transmission.

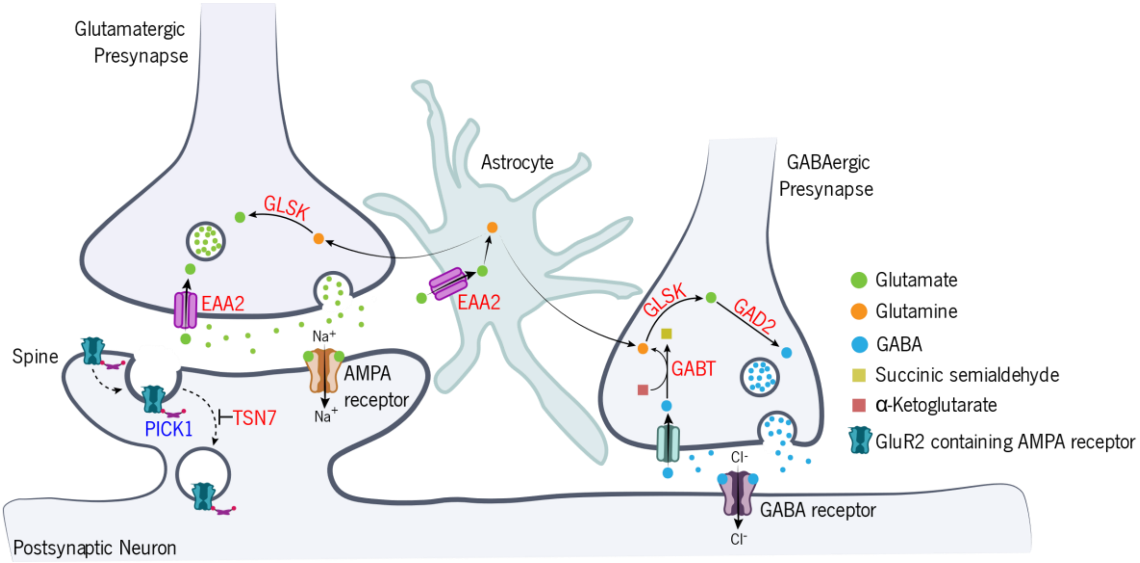

Figure S3 – Schematic representation of the excitatory and inhibitory synaptic proteins altered after chronic administration of haloperidol.

Up-regulated proteins in haloperidol-treated mice are depicted in red, while down-regulated proteins are depicted in blue. Glutamatergic synapses are the main excitatory synapses in the mammalian brain. Here, glutamate is released and binds to glutamate receptors present in the postsynaptic membrane, such as AMPA receptors, promoting local depolarization of the postsynaptic membrane and, consequently, generating excitatory synaptic currents. After its action, glutamate is removed from the synaptic cleft through the excitatory amino acid 2 (EAA2) present in neurons and astrocytes. In neurons, captured glutamate is recycled, while in astrocytes, glutamate is converted to glutamine, which is subsequently transported to presynaptic (excitatory and inhibitory) neuronal terminals. Once in neurons, glutamine is deaminated by the glutaminase kidney isoform (GLSK) to generate glutamate, which in inhibitory neurons is subsequently catalysed by glutamate decarboxylase 2 (GAD2) to produce inhibitory γ-aminobutyric acid (GABA). In the postsynaptic excitatory neuron, PRKCA-binding protein (PICK1) interacts with glutamate ionotropic receptor AMPA type 2 (GluR2) through its PDZ domain, promoting the internalization of AMPA receptors containing the GluR2 subunit. This internalization is regulated by tetraspanin-7 (TSN7) which interacts with the PDZ domain of PICK1, preventing its interaction with GluR2 receptors and their internalization. At inhibitory synapses, after being released into the synaptic cleft, GABA is removed by GABA transporters and, once inside the presynaptic inhibitory neuron, is metabolized by 4-aminobutyrate aminotransferase (GABT). From this reaction, glutamate and succinic semialdehyde are produced. The succinic semialdehyde can be further oxidized to become succinic acid, which enters the citric acid cycle and produces more glutamate. Glutamate can therefore be converted to GABA, as explained above.

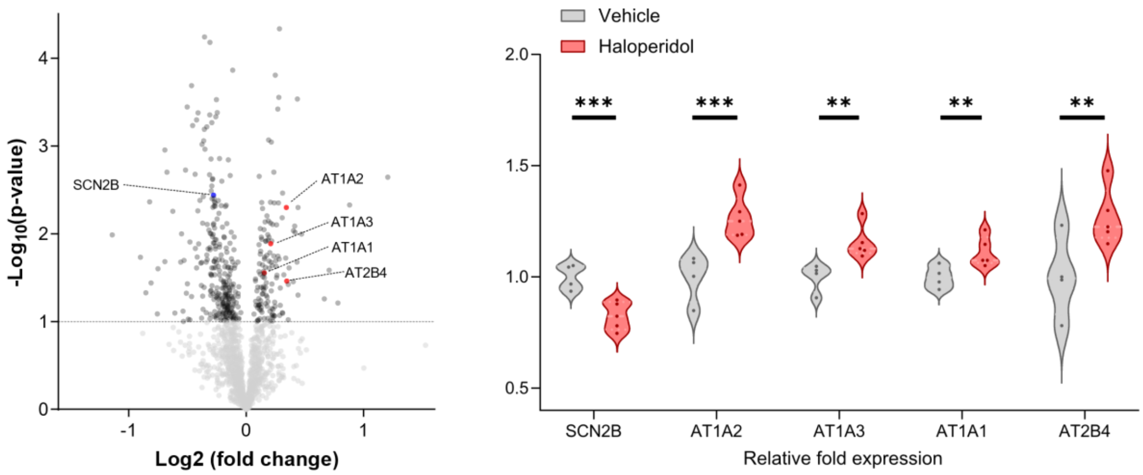

Figure S4. Proteomic analysis revealed a total of 405 proteins significantly altered upon chronic exposure to haloperidol.

Volcano plot of the 1482 proteins detected by proteomic analysis. Proteins statistically altered ($p<0.1$) upon chronic exposure to haloperidol are above the dashed line (405 proteins). Proteins involved in regulating neuronal excitability are highlighted in the volcano plot and further depicted in the violin plots (reduced expression of SCN2B in the haloperidol group, and increased expression of AT1A1, AT1A2, AT1A3, and AT2B4 in the haloperidol group).

SCN2B is a regulatory subunit of voltage-gated sodium channels, which are responsible for the initiation and propagation of action potentials in neurons. The beta-2 subunit modulates the activity of sodium channels by increasing their cell surface expression, slowing their inactivation, and shifting their voltage dependence[2,3]. A reduction in the levels of SCN2B will thus cause a decrease in the cell surface expression of sodium channels, which can decrease neuronal excitability by decreasing the number of channels available for activation.

AT2B4 is the plasma membrane calcium-transporting ATPase 4 (PMCA4), which is a protein that pumps calcium ions out of cells, thereby regulating calcium signalling and homeostasis. PMCA4 is widely expressed in many tissues, including the brain, and has been implicated in a variety of cellular processes, including neurotransmitter release, neuronal excitability, and synaptic plasticity[4–6]. The role of PMCA4 in regulating neuronal excitability is complex and depends on various factors, such as the specific neuronal population and the cellular context, hence, more research is needed to fully understand the role of PMCA4 in regulating neuronal excitability in different cellular and physiological contexts.

Furthermore, we found increased expression of ATP1A1, ATP1A2 and ATP1A3, three sodium/potassium-transporting ATPase subunits that help to maintain the resting membrane potential of neurons by actively transporting sodium ions out of the cell and potassium ions into the cell. An increase in the activity of ATP1A1, ATP1A2 and ATP1A3 can decrease neuronal excitability by hyperpolarizing the cell membrane and increasing the threshold for action potential generation. This is because the outward movement of sodium ions and inward movement of potassium ions generated by ATP1A2/ATP1A3 activity leads to a net negative charge inside the cell, making it more difficult for the membrane potential to depolarize and reach the threshold for action potential firing.

Lastly, several proteins related to mitochondrial metabolism were also found to be changed, which can indirectly influence neuronal excitability. Changes in mitochondrial respiration, reactive oxygen species (ROS) production, and ATP production, can alter the availability of ATP, a key energy source for cellular processes, including ion channel activity and neurotransmitter release. For example, reduced ATP production due to impaired mitochondrial metabolism can lead to decreased activity of ATP-dependent potassium channels and increased activity of voltage-gated calcium channels, leading to an increase in neuronal excitability. Conversely, increased ATP production due to enhanced mitochondrial metabolism can lead to increased activity of ATP-dependent potassium channels and decreased activity of voltage-gated calcium channels, leading to a decrease in neuronal excitability.

A list of all proteins detected by our proteomic analysis can be found in Supplementary Table S1. Furthermore, proteins significantly altered upon chronic exposure to haloperidol can be easily found in another table (Supplementary Table S3) where proteins are ordered by their p values relative to control.

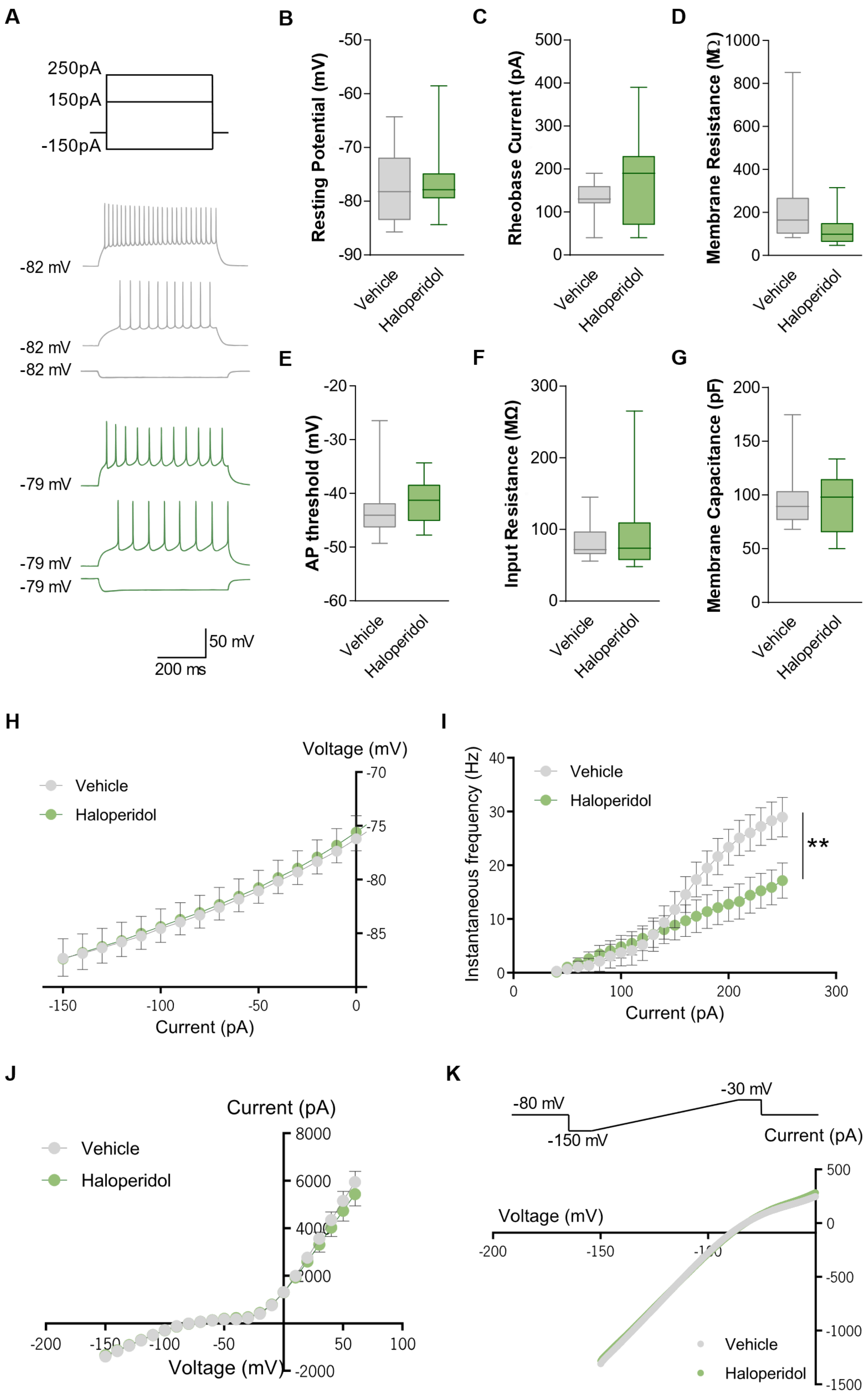
A
250pA
150pA
-150pA
-82 mV
-82 mV
-82 mV
-79 mV
-79 mV
-79 mV
50 mV
200 ms
B
Resting Potential (mV)
Vehicle
Haloperidol
C
Rheobase Current (pA)
D
Membrane Resistance (MΩ)
E
AP threshold (mV)
F
Input Resistance (MΩ)
G
Membrane Capacitance (pF)
H
Voltage (mV)
Current (pA)
I
Instantaneous frequency (Hz)
Current (pA)
**
J
Current (pA)
Voltage (mV)
K
-80 mV
-150 mV
-30 mV
Current (pA)
Voltage (mV)
Vehicle
Haloperidol

Figure S5 – Haloperidol decreases intrinsic excitability in D2-MSNs.

(A) Representative voltage responses to hyperpolarizing (−150 pA) and depolarizing (+150 pA and +250 pA) current pulses in D2-MSNs treated with vehicle (grey) and haloperidol (green) for 30 days. Comparison of **(B)** resting membrane potential, **(C)** action potential triggering current (rheobase), **(D)** membrane resistance, **(E)** action potential (AP) threshold, **(F)** input resistance, and **(G)** membrane capacitance, between vehicle and haloperidol treated mice. **(H)** Current-voltage plot recorded from D2-MSNs. **(I)** Action potential firing frequency (Hz) plotted as a function of injected current steps **(J)** Voltage-current plot recorded from D2-MSNs. **(K)** Mean current response to a 200ms voltage ramp (from −150 to −30 mV, inset, top). Data are mean ± SEM; n=15 vehicle and n=19 haloperidol-treated D2-MSNs for panel B-J and n=15 vehicle and n=19 haloperidol-treated D2-MSNs for panel K; Welch's unpaired t-test for panel B-G. Two-way repeated-measures ANOVA for panel H-K.

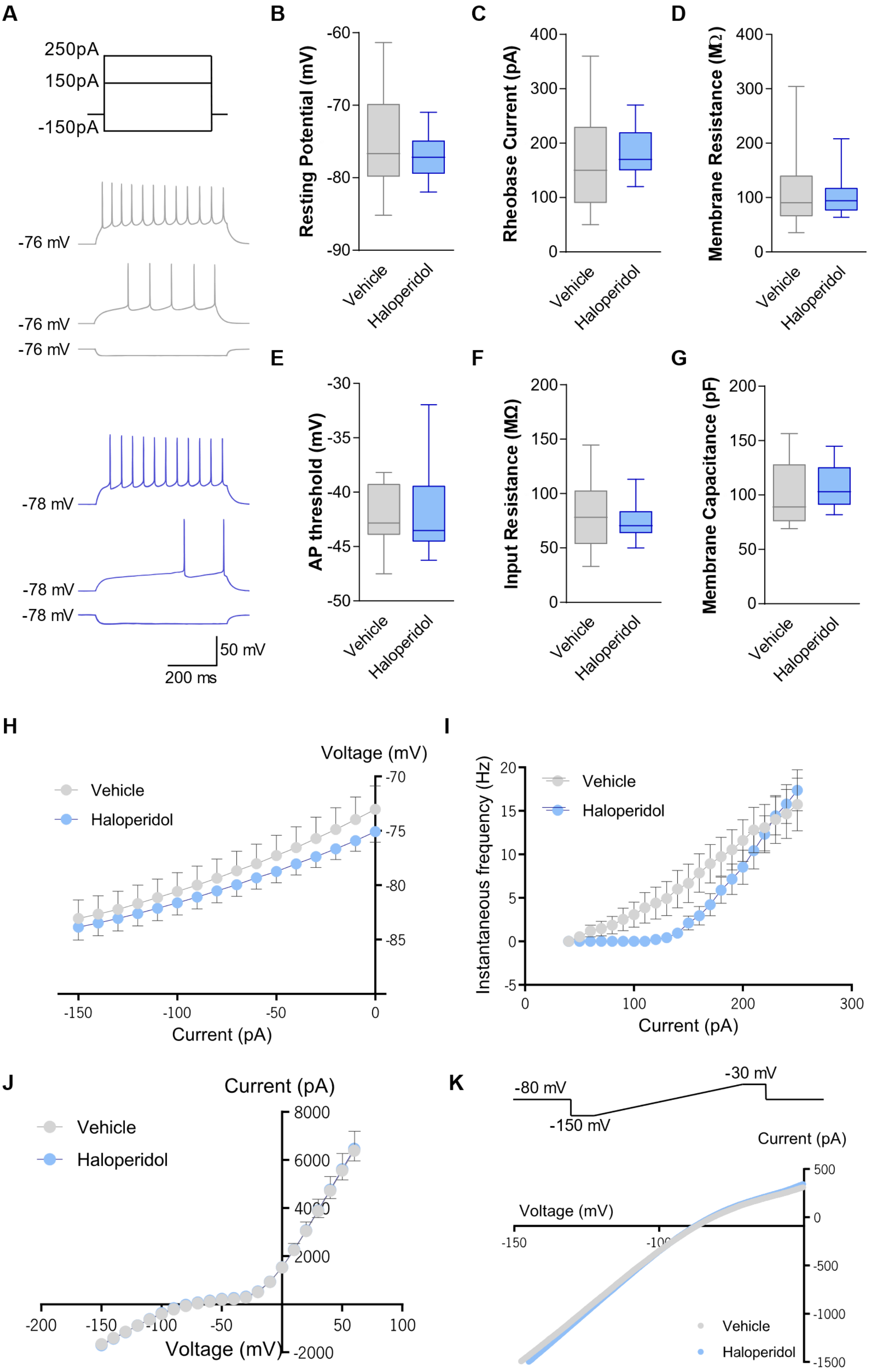
A
250pA
150pA
-150pA
-76 mV
-76 mV
-76 mV
-78 mV
-78 mV
-78 mV
50 mV
200 ms
B
Resting Potential (mV)
Vehicle
Haloperidol
C
Rheobase Current (pA)
D
Membrane Resistance (MΩ)
E
AP threshold (mV)
F
Input Resistance (MΩ)
G
Membrane Capacitance (pF)
H
Voltage (mV)
Current (pA)
I
Instantaneous frequency (Hz)
Current (pA)
J
Current (pA)
Voltage (mV)
K
-80 mV
-150 mV
-30 mV
Current (pA)
Voltage (mV)

Figure S6 – Haloperidol treatment for 30 days did not alter the intrinsic properties of D1-MSNs.

(A) Representative voltage responses to hyperpolarizing (−150 pA) and depolarizing (+150 pA and +250 pA) current pulses in D1-MSNs treated with vehicle (grey) and haloperidol (blue) for 30 days. Comparison of **(B)** resting membrane potential, **(C)** action potential triggering current (rheobase), **(D)** membrane resistance, **(E)** action potential (AP) threshold, **(F)** input resistance, and **(G)** membrane capacitance, between vehicle and haloperidol treated mice. **(H)** Current-voltage plot recorded from D1-MSNs. **(I)** Action potential firing frequency (Hz) plotted as a function of injected current steps. **(J)** Voltage-current plot recorded from D1-MSNs. **(K)** Mean current response to a 200ms voltage ramp (from −150 to −30 mV, inset, top). Data are mean ± SEM; n=15 vehicle and n=19 haloperidol-treated D1-MSNs; Welch's unpaired t-test for panel B-G. Two-way repeated-measures ANOVA for panel H-K.

Supplementary Bibliography

1. Gillespie, M. *et al.* The reactome pathway knowledgebase 2022. *Nucleic Acids Res* **50**, D687–D692 (2022).

2. Lopez-Santiago, L. F. *et al.* Sodium Channel β2 Subunits Regulate Tetrodotoxin-Sensitive Sodium Channels in Small Dorsal Root Ganglion Neurons and Modulate the Response to Pain. *The Journal of Neuroscience* **26**, 7984–7994 (2006).

3. Chen, C. *et al.* Reduced sodium channel density, altered voltage dependence of inactivation, and increased susceptibility to seizures in mice lacking sodium channel β2-subunits. *Proceedings of the National Academy of Sciences* **99**, 17072–17077 (2002).

4. Ghosh, B., Li, Y. & Thayer, S. A. Inhibition of the Plasma Membrane Ca2+ Pump by CD44 Receptor Activation of Tyrosine Kinases Increases the Action Potential Afterhyperpolarization in Sensory Neurons. *The Journal of Neuroscience* **31**, 2361–2370 (2011).

5. Usachev, Y. M., DeMarco, S. J., Campbell, C., Strehler, E. E. & Thayer, S. A. Bradykinin and ATP Accelerate Ca2+ Efflux from Rat Sensory Neurons via Protein Kinase C and the Plasma Membrane Ca2+ Pump Isoform 4. *Neuron* **33**, 113–122 (2002).

6. Khariv, V. & Elkabes, S. Contribution of Plasma Membrane Calcium ATPases to neuronal maladaptive responses: Focus on spinal nociceptive mechanisms and neurodegeneration. *Neurosci Lett* **663**, 60–65 (2018).
