# Supplementary material for: Chronic treatment with D2-antagonist haloperidol leads to inhibitory/excitatory imbalance in striatal D1-neurons
Source: Transl Psychiatry. 2023 Oct 6;13:312. doi: 10.1038/s41398-023-02609-w (PMC10558446; doi:10.1038/s41398-023-02609-w)
Supplement: Supplementary file 2 — Supplementary Table S1 [file 41398_2023_2609_MOESM2_ESM.pdf]

Sup.Table S1 – List of all proteins quantified by proteomic screening of striatal samples from the vehicle and haloperidol-treated mice. A total of 93 proteins were found to be involved in neuronal pathways according to Reactome.

Protein Accession Number	Protein Accession Name	Protein Name	CT#1	CT#2	CT#3	CT#4	Average CT	Stdev CT	% CV CT	30HA#1	30HA#2	30HA#3	30HA#4	30HA#5	Average 30HA	Stdev 30HA	% CV 30HA	Neuronal System in Reactome
[illegible]599C82A1A4:CA4:C1	STXB1_MOUSE	Syntaxin-binding protein 1	0,006214	0,006256	0,00632	0,006083	0,006218351	0,000100116	1,610	0,005783	0,005844	0,005614	0,00578	0,005688	0,00574184	9,03346E-05	1,573	YES
P46460	NSF_MOUSE	Vesicle-fusing ATPase	0,004252	0,004855	0,004785	0,004814	0,004676677	0,000284404	6,081	0,004895	0,004463	0,004666	0,004598	0,005032	0,004730902	0,000229548	4,852	YES
Q7TMM9	TBB2A_MOUSE	Tubulin beta-2A chain	7,45E-05	0,000203	9,48E-05	0,000156	0,000131866	5,83762E-05	44,269	0,000375	0,000307	0,000228	0,000306	0,000305	0,000304347	5,22E-05	17,151	YES
Q9WV92	E41L3_MOUSE	Band 4.1-like protein 3	0,001513	0,001514	0,001291	0,001369	0,001421875	0,000110532	7,774	0,001548	0,001523	0,001418	0,00133	0,001384	0,00144043	9,25074E-05	6,422	YES
Q9Z1B3	PLCB1_MOUSE	1-phosphatidylinositol 4,5-bisphosphate phosphodiesterase beta-1	0,000117	0,000131	0,000171	0,000141	0,000139854	2,28464E-05	16,336	0,000146	0,000144	0,000122	0,00014	0,000171	0,000144601	1,78105E-05	12,317	YES
P08551	NFL_MOUSE	Neurofilament light polypeptide	0,006362	0,005579	0,003533	0,004653	0,005031922	0,001218959	24,225	0,006073	0,005776	0,005469	0,005436	0,004224	0,005395614	0,000704225	13,052	YES
P63017	HSP7C_MOUSE	Heat shock cognate 71 kDa protein	0,004738	0,00523	0,004691	0,004857	0,004879099	0,000244038	5,002	0,005561	0,005523	0,005554	0,005712	0,005968	0,005663518	0,000185062	3,268	YES
P17426	AP2A1_MOUSE	AP-2 complex subunit alpha-1	0,001263	0,001328	0,001344	0,001296	0,00130752	3,58602E-05	2,743	0,001385	0,001353	0,001373	0,001375	0,001517	0,001400606	6,624E-05	4,729	YES
O88935	SYN1_MOUSE	Synapsin-1	0,004012	0,004173	0,004125	0,004076	0,004096457	6,91699E-05	1,689	0,004676	0,004194	0,004431	0,00439	0,004695	0,004477209	0,000210212	4,695	YES
P61922	GABT_MOUSE	4-aminobutyrate aminotransferase, mitochondrial	0,004859	0,005021	0,004626	0,004675	0,004795169	0,000181247	3,780	0,005201	0,005536	0,005242	0,005857	0,005262	0,005419508	0,00027795	5,129	YES
P63318	KPCG_MOUSE	Protein kinase C gamma type	0,000422	0,000414	0,000464	0,000459	0,000439699	2,51932E-05	5,730	0,000305	0,000384	0,000448	0,00042	0,000454	0,000402298	6,11973E-05	15,212	YES
Q62108	DLG4_MOUSE	Disks large homolog 4	0,000199	0,000187	0,000253	0,00022	0,000214911	2,88554E-05	13,427	0,000238	0,000201	0,000217	0,000212	0,000252	0,000223987	2,06611E-05	9,224	YES
P68404	KPCB_MOUSE	Protein kinase C beta type	0,000608	0,000556	0,000504	0,000534	0,000550352	4,3799E-05	7,958	0,000646	0,000491	0,000588	0,000652	0,000714	0,000618158	8,3997E-05	13,588	YES
P11798	KCC2A_MOUSE	Calcium/calmodulin-dependent protein kinase type II subunit alpha	0,003212	0,003692	0,004424	0,003907	0,003808844	0,000502935	13,204	0,003381	0,00262	0,003635	0,003641	0,003773	0,003410144	0,00046373	13,599	YES
P15105	GLNA_MOUSE	Glutamine synthetase	0,003305	0,003388	0,003492	0,003689	0,003468564	0,000165396	4,768	0,003461	0,003449	0,003645	0,003449	0,003235	0,003447717	0,000145238	4,213	YES
D3Z7P3	GLSK_MOUSE	Glutaminase kidney isoform, mitochondrial	0,000481	0,00049	0,00053	0,000514	0,000503858	2,2188E-05	4,404	0,000528	0,00052	0,000542	0,000567	0,000561	0,00054347	2,03825E-05	3,750	YES
P46096	SYT1_MOUSE	Synaptotagmin-1	0,001939	0,002018	0,002119	0,002205	0,00207017	0,000116009	5,604	0,002244	0,001714	0,002055	0,002321	0,00224	0,002114686	0,00024459	11,566	YES
Q8BWF0	SSDH_MOUSE	Succinate-semialdehyde dehydrogenase, mitochondrial	0,001138	0,001162	0,001108	0,001145	0,001138248	2,25556E-05	1,982	0,001099	0,001236	0,001149	0,001058	0,001074	0,001123189	7,17988E-05	6,392	YES
O70318	E41L2_MOUSE	Band 4.1-like protein 2	0,000233	0,000271	0,000247	0,000239	0,000247401	1,67601E-05	6,774	0,000227	0,000284	0,000263	0,000259	0,000272	0,000260965	2,10209E-05	8,055	YES
P48320	DCE2_MOUSE	Glutamate decarboxylase 2	0,000267	0,000345	0,000236	0,000291	0,000284677	4,61364E-05	16,207	0,000284	0,000328	0,000396	0,000414	0,000345	0,00035305	5,2531E-05	14,879	YES
Q9ES97	RTN3_MOUSE	Reticulon-3	0,000496	0,000511	0,000501	0,000493	0,000500488	7,75915E-06	1,550	0,000562	0,000522	0,000489	0,000486	0,000489	0,000509667	3,28231E-05	6,440	YES
Q64332	SYN2_MOUSE	Synapsin-2	0,000964	0,001091	0,001187	0,001124	0,001091463	9,37598E-05	8,590	0,000973	0,000873	0,001025	0,000889	0,001097	0,000971529	9,35788E-05	9,632	YES
Q60634	FLOT2_MOUSE	Flotillin-2	0,000139	0,000148	0,000148	0,000164	0,000149879	1,01025E-05	6,740	0,000161	0,000144	0,000142	0,000161	0,000166	0,000154933	1,08639E-05	7,012	YES
P62874	GBB1_MOUSE	Guanine nucleotide-binding protein G(I)/G(S)/G(T) subunit beta-1	0,00045	0,000548	0,000732	0,000709	0,000609685	0,000134389	22,042	0,000465	0,000687	0,000517	0,000491	0,000554	0,000542662	8,6878E-05	16,010	YES
P47738	ALDH2_MOUSE	Aldehyde dehydrogenase, mitochondrial	0,000509	0,000521	0,000447	0,000465	0,000485619	3,52729E-05	7,263	0,000454	0,00049	0,000507	0,000489	0,000488	0,000485497	1,95919E-05	4,035	YES
Q9D6F9	TBB4A_MOUSE	Tubulin beta-4A chain	0,001962	0,001942	0,001769	0,001784	0,00186391	0,000101975	5,471	0,002231	0,001998	0,00218	0,002188	0,001993	0,002117934	0,000113434	5,356	YES
O08917	FLOT1_MOUSE	Flotillin-1	0,000141	0,000133	0,000123	0,000136	0,000133173	7,54113E-06	5,663	0,000136	0,000104	0,000137	0,000131	0,000131	0,000127839	1,36731E-05	10,696	YES
P28652	KCC2B_MOUSE	Calcium/calmodulin-dependent protein kinase type II subunit beta	0,000215	0,000256	0,000226	0,000231	0,000232248	1,71704E-05	7,393	0,000249	0,000225	0,000237	0,000254	0,000276	0,000248065	1,91794E-05	7,732	YES
P43006	EAA2_MOUSE	Excitatory amino acid transporter 2	0,002515	0,002282	0,002285	0,002344	0,002356444	0,000109592	4,651	0,002575	0,002628	0,002498	0,002596	0,002615	0,002582495	5,13114E-05	1,987	YES
P31324	KAP3_MOUSE	cAMP-dependent protein kinase type II-beta regulatory subunit	0,000561	0,000492	0,000598	0,000503	0,000538335	4,95694E-05	9,208	0,000678	0,00055	0,000568	0,000599	0,000752	0,000629319	8,39399E-05	13,338	YES
P60879	SNP25_MOUSE	Synaptosomal-associated protein 25	0,002368	0,002095	0,001983	0,002116	0,002140256	0,000162698	7,602	0,002373	0,002078	0,002337	0,002329	0,002174	0,002258014	0,000126274	5,592	YES
Q64133	AOFA_MOUSE	Amine oxidase [flavin-containing] A	0,000452	0,000453	0,000429	0,000404	0,000434555	2,31299E-05	5,323	0,000365	0,000407	0,00044	0,000419	0,00038	0,000402256	3,01203E-05	7,488	YES
P31650	S6A11_MOUSE	Sodium- and chloride-dependent GABA transporter 3	0,000674	0,000673	0,000499	0,000648	0,000623355	8,41015E-05	13,492	0,000649	0,000877	0,00071	0,000588	0,000554	0,000675264	0,000127381	18,864	YES
P48318	DCE1_MOUSE	Glutamate decarboxylase 1	0,000179	0,000207	0,000168	0,000183	0,000184199	1,62544E-05	8,824	0,000175	0,000173	0,000175	0,000196	0,000181	0,000180059	9,20938E-06	5,115	YES
P68181	KAPCB_MOUSE	cAMP-dependent protein kinase catalytic subunit beta	0,000165	0,000148	0,000184	0,000165	0,000165448	1,454E-05	8,788	0,000216	0,00019	0,000182	0,000184	0,000213	0,000197001	1,65879E-05	8,420	YES
P08752	GNAI2_MOUSE	Guanine nucleotide-binding protein G(i) subunit alpha-2	0,000441	0,000452	0,000442	0,000416	0,000437814	1,51511E-05	3,461	0,000416	0,000442	0,000432	0,00041	0,000408	0,000421368	1,47164E-05	3,493	YES
Q9ERD7	TBB3_MOUSE	Tubulin beta-3 chain	0,005836	0,005767	0,005491	0,005588	0,005670501	0,000158796	2,800	0,005922	0,005314	0,006205	0,006751	0,007086	0,006255601	0,000695559	11,119	YES
O35633	VIAAT_MOUSE	Vesicular inhibitory amino acid transporter	0,00029	0,000354	0,000299	0,000314	0,000314189	2,83908E-05	9,036	0,000285	0,000329	0,000323	0,000332	0,000296	0,000313102	2,14333E-05	6,845	YES
Q9Z2Y3	HOME1_MOUSE	Homer protein homolog 1	0,00015	0,000137	0,000227	0,000168	0,000170638	3,94951E-05	23,146	0,000181	0,000123	0,000139	0,000167	0,000192	0,000160262	2,90281E-05	18,113	YES
Q3TXX4	VGLU1_MOUSE	Vesicular glutamate transporter 1	0,000209	0,000189	0,000227	0,000209	0,000208607	1,54256E-05	7,395	0,000235	0,000204	0,000183	0,000171	0,000204	0,000199147	2,4427E-05	12,266	YES
P62881	GBB5_MOUSE	Guanine nucleotide-binding protein subunit beta-5	0,000131	0,000122	0,000131	0,00013	0,00012858	4,14715E-06	3,225	0,00014	0,00013	0,000131	0,00014	0,000122	0,000132603	7,67913E-06	5,791	YES
P62880	GBB2_MOUSE	Guanine nucleotide-binding protein G(I)/G(S)/G(T) subunit beta-2	0,000463	0,000504	0,000592	0,000528	0,000521684	5,4236E-05	10,396	0,000409	0,000589	0,0005	0,000502	0,000521	0,000504357	6,42254E-05	12,734	YES
O35526	STX1A_MOUSE	Syntaxin-1A	0,000537	0,000434	0,000563	0,00046	0,000498275	6,13051E-05	12,303	0,000645	0,000524	0,000495	0,000528	0,000542	0,000546746	5,76716E-05	10,548	YES
P56564	EAA1_MOUSE	Excitatory amino acid transporter 1	0,001149	0,00128	0,001425	0,001268	0,001280505	0,000113277	8,846	0,001023	0,001335	0,001215	0,001185	0,001386	0,001228773	0,000141962	11,553	YES
P62204	CALM_MOUSE	Calmodulin	0,000688	0,00076	0,001445	0,000953	0,000961731	0,000340787	35,435	0,000467	0,00043	0,000456	0,000373	0,000457	0,000436502	3,82053E-05	8,753	YES
P46097	SYT2_MOUSE	Synaptotagmin-2	0,000279	0,000314	0,000258	0,000275	0,000281516	2,36473E-05	8,400	0,000276	0,000276	0,000293	0,000198	0,000222	0,000253083	4,07845E-05	16,115	YES
P31648	SC6A1_MOUSE	Sodium- and chloride-dependent GABA transporter 1	0,000151	0,000182	0,000182	0,000174	0,000172292	1,45421E-05	8,440	0,000168	0,00018	0,000173	0,000129	0,000162	0,000162126	1,97911E-05	12,207	YES
P12367	KAP2_MOUSE	cAMP-dependent protein kinase type II-alpha regulatory subunit	0,000195	0,000214	0,000217	0,000192	0,000204451	1,26117E-05	6,169	0,000225	0,000199	0,000235	0,000223	0,000213	0,000218843	1,37907E-05	6,302	YES
P60904	DNJC5_MOUSE	DnaJ homolog subfamily C member 5	0,000123	0,000123	9,6E-05	0,000106	0,000112136	1,33668E-05	11,920	0,000114	0,000122	0,000106	9,25E-05	0,000104	0,000107824	1,09909E-05	10,193	YES
P63044	VAMP2_MOUSE	Vesicle-associated membrane protein 2	0,000324	0,000375	0,000363	0,000348	0,000352778	2,21345E-05	6,274	0,000391	0,000354	0,000367	0,000448	0,000377	0,000387532	3,63223E-05	9,373	YES
P68368	TBA4A_MOUSE	Tubulin alpha-4A chain	0,000202	0,000187	0,000193	0,000174	0,00018875	1,1585E-05	6,138	0,000148	0,000217	0,000166	0,000187	0,000221	0,000187797	3,16982E-05	16,879	YES
Q923T9	KCC2G_MOUSE	Calcium/calmodulin-dependent protein kinase type II subunit gamma	0,000334	0,000292	0,000264	0,000276	0,000291671	3,05831E-05	10,485	0,000281	0,000291	0,000289	0,000281	0,000255	0,000279263	1,4337E-05	5,134	YES
Q9JM63	KCJ10_MOUSE	ATP-sensitive inward rectifier potassium channel 10	0,000195	0,000213	0,000164	0,000193	0,000191214	1,99463E-05	10,431	0,000213	0,000319	0,000185	0,000193	0,000194	0,000220688	5,58533E-05	25,309	YES
P05132	KAPCA_MOUSE	cAMP-dependent protein kinase catalytic subunit alpha	0,000243	0,000245	0,000222	0,000241	0,000237858	1,07806E-05	4,532	0,000212	0,000191	0,000195	0,000189	0,000184	0,000194318	1,0837E-05	5,577	YES
P63213	GBG2_MOUSE	Guanine nucleotide-binding protein G(I)/G(S)/G(O) subunit gamma-2	0,000203	0,000258	0,000264	0,000256	0,000245454	2,82474E-05	11,508	0,000258	0,000281	0,000281	0,000281	0,000274	0,000275141	1,01092E-05	3,674	YES
Q62283	TSN7_MOUSE	Tetraspanin-7	6,19E-05	5,91E-05	6,16E-05	5,73E-05	5,99713E-05	2,16931E-06	3,617	8,53E-05	7,07E-05	8,33E-05	8,19E-05	8,46E-05	8,11771E-05	6,00517E-06	7,398	YES
Q811D0	DLG1_MOUSE	Disks large homolog 1	0,000184	0,000192	0,000189	0,000187	0,000188221	3,33271E-06	1,771	0,000194	0,000185	0,000185	0,000168	0,000186	0,000183642	9,55389E-06	5,202	YES
Q9DBC7	KAP0_MOUSE	cAMP-dependent protein kinase type I-alpha regulatory subunit	0,000111	9,47E-05	9,6E-05	9,24E-05	9,85818E-05	8,61826E-06	8,742	0,00011	8,91E-05	9,82E-05	0,000108	9,34E-05	9,9878E-05	9,27795E-06	9,289	YES
B2RSH2	GNAI1_MOUSE	Guanine nucleotide-binding protein G(i) subunit alpha-1	0,000435	0,00045	0,000497	0,000454	0,00045888	2,68862E-05	5,859	0,00049	0,000451	0,000479	0,000471	0,000456	0,000469624	1,60363E-05	3,415	YES
Q91YS8	KCC1A_MOUSE	Calcium/calmodulin-dependent protein kinase type 1	2,45E-05	2,82E-05	2,93E-05	2,75E-05	2,73894E-05	2,05827E-06	7,515	3,15E-05	2,75E-05	2,56E-05	2,72E-05	2,68E-05	2,77184E-05	2,21574E-06	7,994	YES
P63141	KCNA2_MOUSE	Potassium voltage-gated channel subfamily A member 2	9,59E-05	0,000105	0,000104	9,57E-05	0,000100321	5,19893E-06	5,182	0,000102	0,000109	9,14E-05	9,45E-05	9,46E-05	9,8206E-05	7,08732E-06	7,217	YES
Q6PHZ2	KCC2D_MOUSE	Calcium/calmodulin-dependent protein kinase type II subunit delta	0,000194	0,000224	0,000257	0,00022	0,000224019	2,5784E-05	11,510	0,000219	0,000203	0,000213	0,000233	0,000224	0,000218541	1,13948E-05	5,214	YES
P20444	KPCA_MOUSE	Protein kinase C alpha type	2,2E-05	3,92E-05	3,65E-05	4,03E-05	3,44887E-05	8,45565E-06	24,517	2,76E-05	2,28E-05	3,44E-05	3,04E-05	4,04E-05	3,11156E-05	6,68707E-06	21,491	YES
Q8R5J9	PRAF3_MOUSE	PRA1 family protein 3	0,000173	0,000175	0,000168	0,000169	0,000171249	3,62899E-06	2,119	0,000177	0,000157	0,000177	0,000171	0,000185	0,000173412	1,05108E-05	6,061	YES
P23819	GRIA2_MOUSE	Glutamate receptor 2	0,000119	3,96E-05	0,000162	0,000165	0,000121569	5,84911E-05	48,114	0,000133	0,000122	0,000135	0,000144	0,000172	0,000141213	1,88069E-05	13,318	YES
Q64487	PTPRD_MOUSE	Receptor-type tyrosine-protein phosphatase delta	2,42E-05	2,46E-05	2,64E-05	2,38E-05	2,47693E-05	1,14199E-06	4,610	2,54E-05	2,98E-05	2,45E-05	2,3E-05	2,35E-05	2,52314E-05	2,70182E-06	10,708	YES

Q9WV18	GABR1_MOUSE	Gamma-aminobutyric acid type B receptor subunit 1	4,1E-05	4,69E-05	3,45E-05	4,44E-05	4,17056E-05	5,3984E-06	12,944	5,11E-05	2,13E-05	3,81E-05	5,89E-05	3,71E-05	4,13026E-05	1,44357E-05	34,951	YES
Q62418	DBNL_MOUSE	Drebrin-like protein	5,57E-05	5,5E-05	5,84E-05	5,79E-05	5,67504E-05	1,64507E-06	2,899	5,42E-05	4,67E-05	4,89E-05	5,46E-05	5,61E-05	5,20841E-05	4,06566E-06	7,806	YES
Q8CGK7	GNAL_MOUSE	Guanine nucleotide-binding protein G(olf) subunit alpha	3,56E-05	4,11E-05	8,37E-05	4,43E-05	5,11846E-05	2,19724E-05	42,928	5,71E-05	1,08E-05	4,17E-05	7,14E-05	7,58E-05	5,13592E-05	2,63171E-05	51,241	YES
Q6PHS9	CA2D2_MOUSE	Voltage-dependent calcium channel subunit alpha-2/delta-2	3,36E-05	7,54E-05	6,9E-05	7,28E-05	6,2728E-05	1,95758E-05	31,207	5,44E-05	6,5E-05	3,86E-05	6,81E-05	8,29E-05	6,18089E-05	1,64823E-05	26,667	YES
Q6P9K9	NRX3A_MOUSE	Neurexin-3	0,000191	0,000205	0,00018	0,000185	0,000190411	1,056E-05	5,546	0,000209	0,000182	0,000186	0,000178	0,000197	0,000190603	1,26031E-05	6,612	YES
P62482	KCAB2_MOUSE	Voltage-gated potassium channel subunit beta-2	6,67E-05	6,51E-05	5,2E-05	6,44E-05	6,20306E-05	6,75619E-06	10,892	6,13E-05	6,97E-05	5,81E-05	5,61E-05	5,72E-05	6,04916E-05	5,52142E-06	9,128	YES
P35438	NMDZ1_MOUSE	Glutamate receptor ionotropic, NMDA 1	7,93E-05	7,98E-05	9,31E-05	8,29E-05	8,37793E-05	6,39199E-06	7,630	9,04E-05	6,72E-05	6,12E-05	6,77E-05	7,96E-05	7,32136E-05	1,16823E-05	15,956	YES
Q9Z1L5	CA2D3_MOUSE	Voltage-dependent calcium channel subunit alpha-2/delta-3	3,48E-05	2,91E-05	3,25E-05	2,7E-05	3,08493E-05	3,48093E-06	11,284	2,85E-05	3,24E-05	2,64E-05	3,05E-05	3,79E-05	3,11268E-05	4,36981E-06	14,039	YES
Q61016	GBG7_MOUSE	Guanine nucleotide-binding protein G(I)/G(S)/G(O) subunit gamma-7	9,86E-05	8,06E-05	0,000136	7,77E-05	9,83134E-05	2,70015E-05	27,465	0,000101	0,000112	0,000102	0,000129	0,000151	0,000119053	2,10284E-05	17,663	YES
Q80T41	GABR2_MOUSE	Gamma-aminobutyric acid type B receptor subunit 2	6,19E-05	5,86E-05	5,7E-05	5,3E-05	5,76256E-05	3,72776E-06	6,469	6,37E-05	6,33E-05	5,05E-05	4,5E-05	4,64E-05	5,37797E-05	9,08901E-06	16,900	YES
P84309	ADCY5_MOUSE	Adenylate cyclase type 5	4,76E-05	4,32E-05	5,92E-05	3,66E-05	4,66322E-05	9,50241E-06	20,377	5,62E-05	4,28E-05	3,86E-05	6,64E-05	7,78E-05	5,63877E-05	1,62735E-05	28,860	YES
Q9Z2W9	GRIA3_MOUSE	Glutamate receptor 3	4E-05	4,34E-05	6,78E-05	4,78E-05	4,97317E-05	1,24329E-05	25,000	4,83E-05	3,76E-05	2,82E-05	4,02E-05	4,55E-05	3,99642E-05	7,82767E-06	19,587	YES
Q8K377	LRRT1_MOUSE	Leucine-rich repeat transmembrane neuronal protein 1	1,28E-05	1,12E-05	9,98E-06	1,32E-05	1,17638E-05	1,46971E-06	12,493	1,78E-05	1,34E-05	8,97E-06	1,17E-05	1,16E-05	1,26931E-05	3,26522E-06	25,724	YES
P63040	CPLX1_MOUSE	Complexin-1	1,34E-05	1,45E-05	1,75E-05	1,92E-05	1,61536E-05	2,6791E-06	16,585	1,46E-05	2,32E-05	1,97E-05	1,81E-05	1,45E-05	1,80223E-05	3,6694E-06	20,360	YES
P70175	DLG3_MOUSE	Disks large homolog 3	2,74E-05	2,87E-05	3,23E-05	3,06E-05	2,97654E-05	2,12512E-06	7,140	3,1E-05	2,6E-05	2,66E-05	2,73E-05	3,32E-05	2,88138E-05	3,11042E-06	10,795	YES
Q91X97	NCALD_MOUSE	Neurocalcin-delta	0,000251	0,000186	0,000212	0,000188	0,000209148	3,01075E-05	14,395	0,000237	0,00015	0,000211	0,000141	0,000132	0,000174536	4,68417E-05	26,838	YES
Q64331	MYO6_MOUSE	Unconventional myosin-VI	2,91E-05	3,4E-05	2,94E-05	3,14E-05	3,09779E-05	2,27007E-06	7,328	3,27E-05	4,12E-05	3,31E-05	3,41E-05	2,12E-06	2,86594E-05	1,52363E-05	53,164	YES
Q62083	PICK1_MOUSE	PRKCA-binding protein	3,08E-05	3,19E-05	3,06E-05	3,03E-05	3,09002E-05	6,81269E-07	2,205	2,56E-05	2,25E-05	2,46E-05	2,92E-05	2,58E-05	2,55404E-05	2,43671E-06	9,541	YES
Q99NE5	RIMS1_MOUSE	Regulating synaptic membrane exocytosis protein 1	2,6E-05	2,64E-05	2,76E-05	2,31E-05	2,57843E-05	1,90424E-06	7,385	3,32E-05	2,56E-05	2,23E-05	2,33E-05	2,55E-05	2,5983E-05	4,28392E-06	16,487	YES
P62812	GBRA1_MOUSE	Gamma-aminobutyric acid receptor subunit alpha-1	1,87E-05	1,93E-05	1,55E-05	2,33E-05	1,9205E-05	3,21702E-06	16,751	2,32E-05	2,46E-05	2E-05	2,17E-05	1,6E-05	2,1101E-05	3,35149E-06	15,883	YES
Q8VBY2	KKCC1_MOUSE	Calcium/calmodulin-dependent protein kinase kinase 1	3,8E-05	4,05E-05	3,69E-05	4,32E-05	3,96407E-05	2,82164E-06	7,118	2,18E-05	2,2E-06	3,62E-05	3,89E-05	3,92E-05	2,76542E-05	1,59194E-05	57,566	YES
P16388	KCNA1_MOUSE	Potassium voltage-gated channel subfamily A member 1	5,21E-05	8,35E-06	3,9E-05	3,7E-05	3,41026E-05	1,84253E-05	54,029	5,05E-05	5,11E-05	4,61E-05	4,69E-05	4,05E-05	4,70138E-05	4,2305E-06	8,998	YES
P63216	GBG3_MOUSE	Guanine nucleotide-binding protein G(I)/G(S)/G(O) subunit gamma-3	3,18E-05	3,64E-05	5,36E-05	3,79E-05	3,99257E-05	9,48285E-06	23,751	3,03E-05	2,7E-06	3,3E-05	3,23E-05	4,43E-05	2,85244E-05	1,54383E-05	54,123	YES
Q4ACU6	SHAN3_MOUSE	SH3 and multiple ankyrin repeat domains protein 3	5,22E-06	3,95E-06	4,86E-06	5,55E-06	4,8941E-06	6,87245E-07	14,042	5,45E-06	2,29E-06	3,96E-06	4,31E-06	7,14E-06	4,62978E-06	1,8021E-06	38,924	YES
P63085	MK01_MOUSE	Mitogen-activated protein kinase 1	0,000145	0,000194	0,000241	0,000201	0,00019533	3,90304E-05	19,982	0,000189	0,000172	0,00017	0,000168	0,000189	0,000177756	1,03895E-05	5,845	YES
O70589	CSKP_MOUSE	Peripheral plasma membrane protein CASK	1,8E-05	1,67E-05	1,82E-05	1,84E-05	1,77984E-05	7,46707E-07	4,195	2,05E-05	1,93E-05	1,82E-05	1,78E-05	1,86E-05	1,88885E-05	1,05557E-06	5,588	YES
P23818	GRIA1_MOUSE	Glutamate receptor 1	3,34E-05	8,43E-05	7,83E-05	9,59E-05	7,29535E-05	2,73904E-05	37,545	5,52E-05	4,88E-05	5,25E-05	2,36E-06	7,88E-05	4,75452E-05	2,78569E-05	58,590	YES
Q9JHU4	DYHC1_MOUSE	Cytoplasmic dynein 1 heavy chain 1	0,001206	0,001316	0,001281	0,00122	0,001255846	5,18228E-05	4,127	0,001404	0,001264	0,001162	0,001199	0,001204	0,001246417	9,5261E-05	7,643	
P16546	SPTN1_MOUSE	Spectrin alpha chain, non-erythrocytic 1	0,003291	0,00357	0,003678	0,003514	0,003513241	0,0001632	4,645	0,003785	0,003561	0,003372	0,003394	0,003825	0,003587547	0,000212316	5,918	
Q62261	SPTB2_MOUSE	Spectrin beta chain, non-erythrocytic 1	0,002733	0,003056	0,002911	0,002808	0,002877016	0,000139786	4,859	0,003245	0,002701	0,002825	0,00285	0,003035	0,002931369	0,000212405	7,246	
Q8C8R3	ANK2_MOUSE	Ankyrin-2	0,000392	0,000447	0,000443	0,000441	0,000430533	2,60164E-05	6,043	0,000459	0,000449	0,000446	0,000444	0,000498	0,000458941	2,24095E-05	4,883	
Q68FD5	CLH1_MOUSE	Clathrin heavy chain 1	0,005602	0,005855	0,005974	0,005775	0,005801523	0,000155982	2,689	0,006706	0,0059	0,005754	0,00619	0,006575	0,006224927	0,000413467	6,642	
P19096	FAS_MOUSE	Fatty acid synthase	0,000602	0,000681	0,00063	0,000647	0,000640219	3,31424E-05	5,177	0,00071	0,000729	0,000652	0,000636	0,00064	0,000673284	4,27877E-05	6,355	
Q9QYR6	MAP1A_MOUSE	Microtubule-associated protein 1A	0,001278	0,00138	0,001323	0,001312	0,001323671	4,23696E-05	3,201	0,001394	0,001376	0,001274	0,001261	0,001284	0,001317861	6,20319E-05	4,707	
I00001	GFP_MALE	0	0,05402	0,040752	0,047206	0,037049	0,044756761	0,007466413	16,682	0,042783	0,049554	0,040484	0,046207	0,048966	0,045598922	0,003919351	8,595	
Q8BPN8	DMXL2_MOUSE	DmX-like protein 2	0,000302	0,000289	0,000336	0,000324	0,000312782	2,13979E-05	6,841	0,000337	0,000262	0,000224	0,000306	0,000326	0,000290978	4,74605E-05	16,311	
P14873	MAP1B_MOUSE	Microtubule-associated protein 1B	0,000951	0,001032	0,00099	0,000973	0,000986663	3,43859E-05	3,485	0,001043	0,001037	0,000987	0,000961	0,001007	0,001007036	3,4121E-05	3,388	
P20357	MTAP2_MOUSE	Microtubule-associated protein 2	0,0007	0,000875	0,001057	0,000891	0,000880751	0,00014575	16,548	0,000906	0,000747	0,00081	0,000756	0,000874	0,00081866	7,02451E-05	8,580	
Q61879	MYH10_MOUSE	Myosin-10	0,000396	0,000424	0,000478	0,000395	0,000423286	3,86794E-05	9,138	0,000482	0,00044	0,000396	0,000379	0,000494	0,000438168	5,06313E-05	11,555	
Q99104	MYO5A_MOUSE	Unconventional myosin-Va	0,000547	0,000578	0,000586	0,000545	0,000564066	2,10612E-05	3,734	0,000654	0,000589	0,000449	0,000563	0,000641	0,000579238	8,20165E-05	14,159	
Q6PIC6	AT1A3_MOUSE	Sodium/potassium-transporting ATPase subunit alpha-3	0,007029	0,006817	0,006084	0,006912	0,006710364	0,000426746	6,360	0,007744	0,008622	0,007515	0,007343	0,007566	0,007758005	0,00050334	6,488	
P39053	DYN1_MOUSE	Dynamin-1	0,002634	0,003103	0,002895	0,003009	0,002910386	0,000203	6,975	0,003457	0,003081	0,003219	0,003351	0,003625	0,003346837	0,000210211	6,281	
Q80TJ1	CAPS1_MOUSE	Calcium-dependent secretion activator 1	0,000501	0,000517	0,000564	0,000555	0,000534259	3,00168E-05	5,618	0,000608	0,000519	0,000547	0,000559	0,000605	0,000567506	3,82415E-05	6,739	
Q8CHC4	SYNJ1_MOUSE	Synaptojanin-1	0,000813	0,000942	0,000853	0,000884	0,000872888	5,44753E-05	6,241	0,000925	0,000797	0,000788	0,000843	0,000853	0,00084132	5,47535E-05	6,508	
Q99KI0	ACON_MOUSE	Aconitate hydratase, mitochondrial	0,006951	0,006763	0,006165	0,006248	0,006531823	0,000384925	5,893	0,006947	0,007018	0,006478	0,006342	0,006873	0,006731682	0,000301834	4,484	
P52480	KPYM_MOUSE	Pyruvate kinase PKM	0,015219	0,016206	0,014443	0,015264	0,015283086	0,00072163	4,722	0,015906	0,014907	0,015328	0,01532	0,015145	0,015321074	0,000368861	2,408	
P08553	NFM_MOUSE	Neurofilament medium polypeptide	0,002432	0,002223	0,001417	0,001857	0,001982147	0,000445482	22,475	0,002384	0,002673	0,002224	0,002097	0,00172	0,002219507	0,000352917	15,901	
Q91V92	ACLY_MOUSE	ATP-citrate synthase	0,000806	0,000921	0,000854	0,000891	0,000867987	4,9342E-05	5,685	0,001053	0,000924	0,000924	0,000979	0,001095	0,000994947	7,66981E-05	7,709	
Q9R0K7	AT2B2_MOUSE	Plasma membrane calcium-transporting ATPase 2	0,000611	0,000547	0,000581	0,000526	0,000566411	3,76508E-05	6,647	0,000676	0,000611	0,000531	0,000517	0,000584	0,000583754	6,399E-05	10,962	
P17710	HXK1_MOUSE	Hexokinase-1	0,005221	0,005734	0,005363	0,005463	0,00544528	0,000216612	3,978	0,00549	0,005633	0,005599	0,005376	0,005973	0,005614209	0,000224513	3,999	
P07901	HS90A_MOUSE	Heat shock protein HSP 90-alpha	0,002953	0,003339	0,002907	0,003043	0,003060621	0,000194199	6,345	0,003236	0,003262	0,00324	0,00309	0,003374	0,00324036	0,000100941	3,115	
P12960	CNTN1_MOUSE	Contactin-1	0,00182	0,001925	0,001839	0,001894	0,001869409	4,85639E-05	2,598	0,002078	0,002042	0,001985	0,001897	0,002083	0,002016816	7,76595E-05	3,851	
Q7TSJ2	MAP6_MOUSE	Microtubule-associated protein 6	0,002234	0,002105	0,001819	0,001912	0,002017437	0,000187148	9,277	0,002087	0,001687	0,001929	0,001819	0,001857	0,001875818	0,000146958	7,834	
O08553	DPYL2_MOUSE	Dihydropyrimidinase-related protein 2	0,010738	0,011815	0,010775	0,010993	0,011080264	0,000502581	4,536	0,011136	0,011375	0,011266	0,012057	0,012117	0,011590285	0,000461761	3,984	
Q6PB66	LPPRC_MOUSE	Leucine-rich PPR motif-containing protein, mitochondrial	9,28E-05	9,66E-05	7,1E-05	9,22E-05	8,81512E-05	1,15876E-05	13,145	0,000104	9,65E-05	9,38E-05	8,32E-05	8,53E-05	9,24593E-05	8,34446E-06	9,025	
Q60597	ODO1_MOUSE	2-oxoglutarate dehydrogenase, mitochondrial	0,00107	0,000933	0,000937	0,000975	0,000978766	6,38487E-05	6,523	0,000962	0,000907	0,000861	0,000873	0,000943	0,000909187	4,33846E-05	4,772	
Q02053	UBA1_MOUSE	Ubiquitin-like modifier-activating enzyme 1	0,001	0,001126	0,001064	0,001068	0,001064613	5,16479E-05	4,851	0,001225	0,001181	0,001145	0,001146	0,001257	0,001190811	4,93954E-05	4,148	
P50516	VATA_MOUSE	V-type proton ATPase catalytic subunit A	0,004206	0,00452	0,004436	0,004531	0,004423337	0,000150734	3,408	0,004836	0,004594	0,004767	0,004792	0,005056	0,004809107	0,000166138	3,455	
Q61316	HSP74_MOUSE	Heat shock 70 kDa protein 4	0,001285	0,00141	0,001323	0,001317	0,001333721	5,35604E-05	4,016	0,001472	0,00142	0,001441	0,001381	0,001531	0,001448922	5,63712E-05	3,891	
P05064	ALDOA_MOUSE	Fructose-bisphosphate aldolase A	0,005339	0,005816	0,005174	0,005533	0,005465455	0,000275871	5,048	0,005993	0,005619	0,00572	0,005853	0,005735	0,005783838	0,000143307	2,478	
O55143	AT2A2_MOUSE	Sarcoplasmic/endoplasmic reticulum calcium ATPase 2	0,000908	0,000856	0,000852	0,00079	0,00085159	4,84311E-05	5,687	0,0011	0,000995	0,000924	0,000973	0,001102	0,00101892	7,93547E-05	7,788	
Q03265	ATPA_MOUSE	ATP synthase subunit alpha, mitochondrial	0,011846	0,01137	0,010834	0,010801	0,01121279	0,000496516	4,428	0,010655	0,010848	0,011101	0,010693	0,010685	0,010796449	0,000186287	1,725	
Q9DBG3	AP2B1_MOUSE	AP-2 complex subunit beta	0,001243	0,00114	0,001194	0,001223	0,001200037	4,51065E-05	3,759	0,001354	0,001145	0,001274	0,001422	0,001479	0,001335033	0,000130901	9,805	
Q01853	TERA_MOUSE	Transitional endoplasmic reticulum ATPase	0,001404	0,00147	0,001467	0,001445	0,001446674	3,05368E-05	2,111	0,001625	0,001566	0,001544	0,001557	0,00175	0,001608228	8,49612E-05	5,283	
Q8CAQ8	MIC60_MOUSE	MICOS complex subunit Mic60	0,000759	0,000767	0,000771	0,000705	0,000750717	3,06754E-05	4,086	0,000729	0,000782	0,000775	0,000733	0,000826	0,000768856	3,97502E-05	5,170	
Q8BYI9	TENR_MOUSE	Tenascin-R	0,001426	0,001576	0,001548	0,001523	0,001517989	6,51386E-05	4,291	0,001703	0,001518	0,001506	0,001579	0,001576	0,001576349	7,78255E-05	4,937	
Q64514	TPP2_MOUSE	Tripeptidyl-peptidase 2	9,27E-05	9,76E-05	0,000104	8,99E-05	9,60891E-05	6,26349E-06	6,518	0,000104	9,27E-05	9,59E-05	8,15E-05	9,76E-05	9,43622E-05	8,31116E-06	8,808	
P16858	G3P_MOUSE	Glyceraldehyde-3-phosphate dehydrogenase	0,019503	0,021183	0,019676	0,02065	0,020252884	0,000799586	3,948	0,021962	0,021471	0,021746	0,021246	0,020607	0,021406236	0,000522788	2,442	
Q810U3	NFASC_MOUSE	Neurofascin	0,001619	0,001728	0,001606	0,001563	0,001629047	7,03842E-05	4,321	0,001741	0,001747	0,001655	0,001599	0,001661	0,00168087	6,27128E-05	3,731	
P08113	ENPL_MOUSE	Endoplasmin	0,000583	0,000601	0,000602	0,000601	0,000597121	9,17007E-06	1,536	0,000545	0,000553	0,000582	0,00052	0,000583	0,000556835	2,66883E-05	4,793	
Q8CI94	PYGB_MOUSE	Glycogen phosphorylase, brain form	0,001174	0,001483	0,001407	0,001391	0,001363886	0,000132539	9,718	0,001377	0,001465	0,001341	0,001306	0,001442	0,001386193	6,67694E-05	4,817	

P60710	ACTB_MOUSE	Actin, cytoplasmic 1	0,006785	0,009546	0,009254	0,00973	0,008828896	0,001376474	15,591	0,010198	0,009738	0,010266	0,009807	0,010146	0,010030817	0,000241184	2,404
Q6ZQ38	CAND1_MOUSE	Cullin-associated NEDD8-dissociated protein 1	0,001202	0,001339	0,001367	0,001355	0,00131571	7,67037E-05	5,830	0,001449	0,001474	0,00131	0,001248	0,001427	0,001381406	9,75053E-05	7,058
Q6PIE5	AT1A2_MOUSE	Sodium/potassium-transporting ATPase subunit alpha-2	0,001551	0,001578	0,001236	0,001461	0,001456311	0,00015511	10,651	0,001884	0,002058	0,001822	0,001735	0,001729	0,001845457	0,000135188	7,325
Q60864	STIP1_MOUSE	Stress-induced-phosphoprotein 1	0,001056	0,001181	0,001057	0,00111	0,001101105	5,91157E-05	5,369	0,001089	0,001036	0,001152	0,00115	0,001168	0,001119074	5,51624E-05	4,929
O54991	CNTP1_MOUSE	Contactin-associated protein 1	0,000615	0,00066	0,000499	0,000612	0,000596494	6,8763E-05	11,528	0,000574	0,000665	0,000593	0,000616	0,000582	0,000606016	3,63305E-05	5,995
P48722	HS74L_MOUSE	Heat shock 70 kDa protein 4L	0,000722	0,000865	0,000776	0,000811	0,000793156	6,01132E-05	7,579	0,000903	0,00087	0,000792	0,000868	0,000893	0,000865328	4,35145E-05	5,029
P58252	EF2_MOUSE	Elongation factor 2	0,000812	0,000976	0,000866	0,00091	0,000890979	6,93616E-05	7,785	0,000906	0,000906	0,000895	0,000829	0,000945	0,000896307	4,20083E-05	4,687
Q11011	PSA_MOUSE	Puromycin-sensitive aminopeptidase	0,001194	0,00131	0,00127	0,001216	0,001247388	5,24094E-05	4,202	0,001303	0,001162	0,001236	0,001204	0,001348	0,001250563	7,49615E-05	5,994
P19246	NFH_MOUSE	Neurofilament heavy polypeptide	0,001397	0,001264	0,000815	0,001007	0,001121004	0,000260269	23,217	0,001414	0,001528	0,00126	0,001257	0,000995	0,001290645	0,000200577	15,541
Q4KMM3	OXR1_MOUSE	Oxidation resistance protein 1	0,000251	0,000314	0,000289	0,000329	0,000295747	3,41663E-05	11,553	0,000358	0,000293	0,000304	0,000304	0,000339	0,000319674	2,76613E-05	8,653
P13595	NCAM1_MOUSE	Neural cell adhesion molecule 1	0,003037	0,003621	0,003147	0,003549	0,003338629	0,000289683	8,677	0,003525	0,003709	0,003709	0,003847	0,003804	0,003718641	0,000123811	3,329
P50396	GDIA_MOUSE	Rab GDP dissociation inhibitor alpha	0,002325	0,002687	0,002496	0,002578	0,002521703	0,000152547	6,049	0,002789	0,002659	0,003005	0,00314	0,003065	0,002931594	0,000200779	6,849
Q9QWI6	SRCN1_MOUSE	SRC kinase signaling inhibitor 1	0,000333	0,000335	0,000356	0,000316	0,000334764	1,62108E-05	4,842	0,000384	0,000253	0,000325	0,000316	0,00034	0,000323288	4,72553E-05	14,617
Q920I9	WDR7_MOUSE	WD repeat-containing protein 7	0,00018	0,000187	0,000194	0,00019	0,000187666	5,98281E-06	3,188	0,000196	0,000167	0,000169	0,000176	0,000184	0,000178179	1,19772E-05	6,722
P16330	CN37_MOUSE	2',3'-cyclic-nucleotide 3'-phosphodiesterase	0,012819	0,012382	0,009525	0,011584	0,011577604	0,001460751	12,617	0,012944	0,013101	0,013659	0,013336	0,010646	0,012737117	0,001199815	9,420
P63038	CH60_MOUSE	60 kDa heat shock protein, mitochondrial	0,002539	0,002597	0,002333	0,002577	0,002511541	0,000121479	4,837	0,002412	0,002529	0,002423	0,002386	0,002482	0,002446513	5,81965E-05	2,379
Q64521	GPDM_MOUSE	Glycerol-3-phosphate dehydrogenase, mitochondrial	0,000846	0,001058	0,00099	0,001023	0,000979341	9,34696E-05	9,544	0,000842	0,000842	0,000896	0,000874	0,000883	0,000867389	2,44673E-05	2,821
P56399	UBP5_MOUSE	Ubiquitin carboxyl-terminal hydrolase 5	0,001265	0,001325	0,001304	0,001257	0,001287813	3,26023E-05	2,532	0,001236	0,001259	0,001276	0,001313	0,001396	0,001295972	6,28079E-05	4,846
Q8K1M6	DNM1L_MOUSE	Dynamin-1-like protein	0,001081	0,001219	0,001178	0,001157	0,001158917	5,77138E-05	4,980	0,001218	0,001099	0,00111	0,001071	0,001206	0,001140721	6,66239E-05	5,841
P07724	ALBU_MOUSE	Serum albumin	0,004826	0,002757	0,003824	0,004026	0,003858213	0,000852055	22,084	0,002402	0,002001	0,002228	0,001767	0,002513	0,002182405	0,000302285	13,851
Q99P72	RTN4_MOUSE	Reticulon-4	0,00042	0,000412	0,000393	0,000382	0,00040187	1,7221E-05	4,285	0,00049	0,000431	0,000433	0,000424	0,000439	0,000443476	2,65411E-05	5,985
P97427	DPYL1_MOUSE	Dihydropyrimidinase-related protein 1	0,001071	0,001198	0,001153	0,001173	0,001148923	5,48545E-05	4,774	0,001267	0,001178	0,001293	0,001378	0,001369	0,001297019	8,17324E-05	6,302
P09411	PGK1_MOUSE	Phosphoglycerate kinase 1	0,003393	0,003204	0,003471	0,003575	0,003410689	0,000157025	4,604	0,003126	0,002888	0,003249	0,003372	0,003097	0,003146385	0,000180811	5,747
P58281	OPA1_MOUSE	Dynamin-like 120 kDa protein, mitochondrial	0,000368	0,000393	0,000401	0,000382	0,000385751	1,41619E-05	3,671	0,000394	0,000387	0,000392	0,00039	0,000473	0,00040748	3,68995E-05	9,056
P05202	AATM_MOUSE	Aspartate aminotransferase, mitochondrial	0,009099	0,008805	0,008577	0,008569	0,00876261	0,000249427	2,846	0,008956	0,008156	0,008094	0,007845	0,007662	0,008142712	0,000495767	6,088
Q8VDN2	AT1A1_MOUSE	Sodium/potassium-transporting ATPase subunit alpha-1	0,003156	0,003035	0,003298	0,00293	0,0031048	0,000158274	5,098	0,003559	0,003264	0,003337	0,003337	0,003763	0,003452123	0,000206279	5,975
P47857	PFKAM_MOUSE	ATP-dependent 6-phosphofructokinase, muscle type	0,00126	0,001266	0,001211	0,001138	0,001218875	5,9289E-05	4,864	0,001374	0,00128	0,001335	0,001284	0,001367	0,001328121	4,45945E-05	3,358
O70133	DHX9_MOUSE	ATP-dependent RNA helicase A	0,000148	0,000175	0,000162	0,00014	0,000156378	1,56503E-05	10,008	0,000128	0,000115	0,000118	0,000149	0,000146	0,000130874	1,57884E-05	12,064
O88342	WDR1_MOUSE	WD repeat-containing protein 1	0,000829	0,000948	0,000834	0,00097	0,000895384	7,41923E-05	8,286	0,000874	0,000802	0,000873	0,000829	0,000844	0,000844389	3,06885E-05	3,634
Q61699	HS105_MOUSE	Heat shock protein 105 kDa	0,000603	0,000703	0,000688	0,000646	0,000660074	4,4879E-05	6,799	0,000702	0,000757	0,000691	0,000656	0,000801	0,000721496	5,73457E-05	7,948
P38647	GRP75_MOUSE	Stress-70 protein, mitochondrial	0,001864	0,002632	0,002322	0,002437	0,002313527	0,000326176	14,099	0,002339	0,002419	0,001881	0,002325	0,00258	0,002308637	0,000259639	11,246
P27773	PDIA3_MOUSE	Protein disulfide-isomerase A3	0,00098	0,001071	0,000937	0,000991	0,000994964	5,56913E-05	5,597	0,000943	0,000936	0,000995	0,000932	0,000957	0,000952423	2,53763E-05	2,664
Q9Z1G4	VPP1_MOUSE	V-type proton ATPase 116 kDa subunit a isoform 1	0,00238	0,002586	0,00265	0,002606	0,002555353	0,000120269	4,707	0,002839	0,002588	0,002549	0,002586	0,002929	0,002698267	0,000173465	6,429
Q6ZPJ3	UBE2O_MOUSE	(E3-independent) E2 ubiquitin-conjugating enzyme UBE2O	2,45E-05	2,75E-05	2,69E-05	2,77E-05	2,66297E-05	1,48364E-06	5,571	2,99E-05	2,49E-05	2,93E-05	3,32E-05	3,16E-05	2,97846E-05	3,12427E-06	10,490
P26443	DHE3_MOUSE	Glutamate dehydrogenase 1, mitochondrial	0,004079	0,004234	0,003934	0,004158	0,004101126	0,00012814	3,125	0,004238	0,003619	0,004677	0,004634	0,004086	0,004250928	0,000434421	10,219
Q8BHN3	GANAB_MOUSE	Neutral alpha-glucosidase AB	0,000198	0,000191	0,000198	0,000205	0,000198036	5,69826E-06	2,877	0,000209	0,000183	0,000194	0,000186	0,000193	0,000192963	1,01205E-05	5,245
Q8R0Y6	AL1L1_MOUSE	Cytosolic 10-formyltetrahydrofolate dehydrogenase	0,000595	0,000648	0,000568	0,00061	0,00060509	3,33705E-05	5,515	0,000584	0,000651	0,000599	0,00058	0,000571	0,00059687	3,19124E-05	5,347
Q91VD9	NDUS1_MOUSE	NADH-ubiquinone oxidoreductase 75 kDa subunit, mitochondrial	0,001944	0,00182	0,00175	0,001689	0,001800949	0,000109668	6,089	0,001729	0,001797	0,001722	0,00172	0,001807	0,001754896	4,30225E-05	2,452
Q04447	KCRB_MOUSE	Creatine kinase B-type	0,007275	0,006526	0,009916	0,007795	0,007877782	0,001455007	18,470	0,006809	0,005586	0,005646	0,006498	0,006174	0,006142549	0,000530995	8,645
Q9Z2H5	E41L1_MOUSE	Band 4.1-like protein 1	0,000371	0,00041	0,000418	0,000414	0,000403061	2,174E-05	5,394	0,000413	0,000428	0,000436	0,000421	0,000485	0,000436749	2,85015E-05	6,526
P20029	GRP78_MOUSE	78 kDa glucose-regulated protein	0,001603	0,001769	0,001689	0,001617	0,001669394	7,65355E-05	4,585	0,001721	0,001709	0,00171	0,001615	0,001922	0,001735561	0,000112766	6,497
Q8BZ98	DYN3_MOUSE	Dynamin-3	0,000244	0,000249	0,000245	0,000257	0,000248618	6,27216E-06	2,523	0,000283	0,000298	0,000308	0,000295	0,000322	0,000301419	1,45632E-05	4,832
Q8K0U4	HS12A_MOUSE	Heat shock 70 kDa protein 12A	0,00214	0,002023	0,001715	0,002061	0,001984827	0,000186056	9,374	0,002165	0,001841	0,001952	0,002073	0,00189	0,001984326	0,00013305	6,705
P70704	AT8A1_MOUSE	Phospholipid-transporting ATPase IA	0,000267	0,000339	0,000324	0,000304	0,000308563	3,11638E-05	10,100	0,000299	0,000314	0,000287	0,000302	0,000336	0,000307609	1,8468E-05	6,004
P14824	ANXA6_MOUSE	Annexin A6	0,000457	0,000495	0,000478	0,000485	0,000478807	1,62976E-05	3,404	0,000529	0,000511	0,000513	0,000512	0,000521	0,000516982	7,57352E-06	1,465
Q9EQF6	DPYL5_MOUSE	Dihydropyrimidinase-related protein 5	0,000375	0,000421	0,000353	0,00041	0,000389787	3,14045E-05	8,057	0,000459	0,000439	0,000485	0,000486	0,000403	0,000454768	3,47756E-05	7,647
Q8BH59	CMC1_MOUSE	Calcium-binding mitochondrial carrier protein Aralar1	0,002746	0,002762	0,002644	0,002561	0,002678419	9,41184E-05	3,514	0,002428	0,002306	0,002409	0,002423	0,002484	0,002410282	6,48548E-05	2,691
P40142	TKT_MOUSE	Transketolase	0,001911	0,00213	0,001793	0,001974	0,001951881	0,000140088	7,177	0,00197	0,00193	0,002083	0,002083	0,001893	0,0019915	8,76994E-05	4,404
P46660	AINX_MOUSE	Alpha-internexin	0,002562	0,002236	0,001572	0,001751	0,002030588	0,000452092	22,264	0,002422	0,002336	0,00217	0,002172	0,001868	0,002193378	0,000211683	9,651
P03995	GFAP_MOUSE	Glial fibrillary acidic protein	0,001829	0,00233	0,001511	0,002028	0,001924416	0,000344154	17,884	0,001717	0,001604	0,002063	0,001753	0,001431	0,001713494	0,000232173	13,550
Q9QYC0	ADDA_MOUSE	Alpha-adducin	0,000578	0,000688	0,000637	0,000613	0,000629238	4,65096E-05	7,391	0,000646	0,000585	0,000611	0,000613	0,000695	0,000630117	4,24313E-05	6,734
Q9WUB3	PYGM_MOUSE	Glycogen phosphorylase, muscle form	0,000555	0,000591	0,000477	0,000555	0,000544357	4,7929E-05	8,805	0,000594	0,000652	0,0006	0,000529	0,00051	0,00057688	5,74867E-05	9,965
Q91V14	S12A5_MOUSE	Solute carrier family 12 member 5	0,000894	0,000971	0,001023	0,000938	0,00095667	5,4569E-05	5,704	0,001044	0,000914	0,000898	0,000899	0,000973	0,00094557	6,26772E-05	6,629
P06745	G6PI_MOUSE	Glucose-6-phosphate isomerase	0,003891	0,003664	0,003479	0,003882	0,003728692	0,000196998	5,283	0,003764	0,003928	0,004099	0,004295	0,004005	0,004018108	0,000197551	4,917
P56480	ATPB_MOUSE	ATP synthase subunit beta, mitochondrial	0,010283	0,009976	0,009883	0,00986	0,01000057	0,000194754	1,947	0,009946	0,009461	0,009945	0,010238	0,010232	0,009964288	0,000316444	3,176
P80318	TCPG_MOUSE	T-complex protein 1 subunit gamma	0,000744	0,000836	0,00082	0,000812	0,000803041	4,04957E-05	5,043	0,000802	0,000745	0,00083	0,000841	0,000797	0,000802805	3,70622E-05	4,617
Q80SW1	SAHH2_MOUSE	Putative adenosylhomocysteinase 2	0,000391	0,000395	0,000363	0,000419	0,000391928	2,27308E-05	5,800	0,000375	0,000436	0,000407	0,000382	0,000326	0,00038546	4,08093E-05	10,587
O08532	CA2D1_MOUSE	Voltage-dependent calcium channel subunit alpha-2/delta-1	0,000241	0,000298	0,000313	0,000288	0,000284927	3,0986E-05	10,875	0,000275	0,000292	0,000281	0,000282	0,000289	0,000283868	6,80116E-06	2,396
P17182	ENOA_MOUSE	Alpha-enolase	0,005045	0,005032	0,005629	0,005707	0,005353523	0,000364984	6,818	0,004858	0,004707	0,004863	0,00528	0,00531	0,005003577	0,000273259	5,461
Q9Z218	DPP6_MOUSE	Dipeptidyl aminopeptidase-like protein 6	0,000485	0,000562	0,000576	0,000552	0,000543877	4,06764E-05	7,479	0,000536	0,000583	0,000593	0,000563	0,000654	0,000585977	4,39215E-05	7,495
O55131	SEPT7_MOUSE	Septin-7	0,001338	0,00117	0,001173	0,001147	0,001206863	8,79375E-05	7,286	0,001322	0,001163	0,001282	0,001315	0,001213	0,001259295	6,88034E-05	5,464
G5E829	AT2B1_MOUSE	Plasma membrane calcium-transporting ATPase 1	0,000584	0,000479	0,000567	0,000523	0,000538165	4,72721E-05	8,784	0,000774	0,000541	0,000512	0,000615	0,000709	0,000630331	0,000110762	17,572
P24527	LKHA4_MOUSE	Leukotriene A-4 hydrolase	0,000359	0,00038	0,000379	0,000339	0,000364368	1,93249E-05	5,304	0,000368	0,00039	0,000387	0,000391	0,000397	0,000386545	1,09879E-05	2,843
P05063	ALDOC_MOUSE	Fructose-bisphosphate aldolase C	0,003553	0,002743	0,00292	0,00333	0,003136446	0,00037119	11,835	0,0033	0,003219	0,00311	0,003128	0,002566	0,003064639	0,000288838	9,425
P42932	TCPQ_MOUSE	T-complex protein 1 subunit theta	0,000956	0,001131	0,001002	0,001049	0,001034442	7,44663E-05	7,199	0,001082	0,001012	0,001108	0,001167	0,001077	0,001089177	5,60204E-05	5,143
Q8BIJ6	SYIM_MOUSE	Isoleucine--tRNA ligase, mitochondrial	0,000176	0,000189	0,000167	0,000166	0,000174712	1,09538E-05	6,270	0,000172	0,000178	0,000168	0,000159	0,000187	0,000172795	1,02734E-05	5,945
Q9CZU6	CISY_MOUSE	Citrate synthase, mitochondrial	0,003566	0,003675	0,003435	0,003508	0,003545913	0,000101338	2,858	0,003671	0,003438	0,003596	0,003581	0,003477	0,003552541	9,39278E-05	2,644
O88343	S4A4_MOUSE	Electrogenic sodium bicarbonate cotransporter 1	0,000901	0,001204	0,00095	0,000993	0,001011989	0,000133613	13,203	0,00126	0,001152	0,001204	0,001095	0,001161	0,001174441	6,17849E-05	5,261
Q76MZ3	2AAA_MOUSE	Serine/threonine-protein phosphatase 2A 65 kDa regulatory subunit A alpha isoform	0,001293	0,00148	0,001372	0,001374	0,001379888	7,68959E-05	5,573	0,0012	0,001408	0,001437	0,001478	0,001515	0,001407748	0,000122719	8,717
Q9QXL2	KI21A_MOUSE	Kinesin-like protein KIF21A	6,62E-05	6,57E-05	6,84E-05	7,18E-05	6,80352E-05	2,77782E-06	4,083	8,38E-05	7,22E-05	6,04E-05	6,15E-05	7,52E-05	7,06185E-05	9,80155E-06	13,880
Q61598	GDIB_MOUSE	Rab GDP dissociation inhibitor beta	0,001039	0,001076	0,001049	0,001035	0,00104972	1,83841E-05	1,751	0,00106	0,00099	0,001173	0,001158	0,001148	0,001105792	7,81151E-05	7,064

P57780	ACTN4_MOUSE	Alpha-actinin-4	6,24E-05	6,39E-05	7,45E-05	5,82E-05	6,4731E-05	6,94113E-06	10,723	6,08E-05	5,67E-05	6,14E-05	5,32E-05	6,16E-05	5,87364E-05	3,70385E-06	6,306
P35564	CALX_MOUSE	Calnexin	0,00074	0,000807	0,000731	0,000752	0,000757323	3,39439E-05	4,482	0,000766	0,000815	0,000794	0,000741	0,000819	0,000786992	3,29794E-05	4,191
Q9WUA3	PFKAP_MOUSE	ATP-dependent 6-phosphofructokinase, platelet type	0,000617	0,000607	0,000596	0,000624	0,000610804	1,19924E-05	1,963	0,000742	0,000685	0,000711	0,00073	0,00076	0,00072538	2,89239E-05	3,987
Q91WC3	ACSL6_MOUSE	Long-chain-fatty-acid--CoA ligase 6	0,000613	0,000463	0,00047	0,000425	0,000492846	8,25298E-05	16,746	0,00058	0,00059	0,000559	0,000597	0,000542	0,000573497	2,23745E-05	3,901
Q61548	AP180_MOUSE	Clathrin coat assembly protein AP180	0,000565	0,000605	0,000645	0,000642	0,000614429	3,77723E-05	6,148	0,000685	0,000603	0,000687	0,000623	0,000694	0,000658175	4,2247E-05	6,419
P35486	ODPA_MOUSE	Pyruvate dehydrogenase E1 component subunit alpha, somatic form, mitochondrial	0,001663	0,001606	0,001632	0,001653	0,001638571	2,50454E-05	1,528	0,001633	0,001492	0,001675	0,00163	0,001524	0,001590971	7,82869E-05	4,921
Q61644	PACN1_MOUSE	Protein kinase C and casein kinase substrate in neurons protein 1	0,000628	0,000679	0,00068	0,000647	0,000658437	2,56221E-05	3,891	0,000699	0,000668	0,000687	0,000645	0,000703	0,000680429	2,41012E-05	3,542
Q9Z2I9	SUCB1_MOUSE	Succinate--CoA ligase [ADP-forming] subunit beta, mitochondrial	0,001065	0,001104	0,001075	0,001158	0,001100465	4,21193E-05	3,827	0,00109	0,001013	0,001176	0,001203	0,001149	0,001126251	7,59936E-05	6,747
P17427	AP2A2_MOUSE	AP-2 complex subunit alpha-2	0,000728	0,000734	0,000778	0,000777	0,000754211	2,69804E-05	3,577	0,000826	0,000789	0,000805	0,000846	0,000961	0,000845395	6,79359E-05	8,036
Q61361	PGCB_MOUSE	Brevican core protein	0,000508	0,000444	0,000577	0,000525	0,000513668	5,4988E-05	10,705	0,000498	0,00049	0,000476	0,000476	0,000507	0,0004896	1,34328E-05	2,744
P05201	AATC_MOUSE	Aspartate aminotransferase, cytoplasmic	0,00474	0,00408	0,004691	0,004901	0,004603216	0,000359904	7,819	0,004218	0,003639	0,004049	0,004216	0,003873	0,003999189	0,000246533	6,165
P62814	VATB2_MOUSE	V-type proton ATPase subunit B, brain isoform	0,003169	0,003444	0,003686	0,003386	0,003421064	0,000212534	6,212	0,003472	0,003046	0,003517	0,003713	0,003608	0,003471186	0,000255001	7,346
Q61838	PZP_MOUSE	Pregnancy zone protein	0,000141	8,31E-05	8,66E-05	9,61E-05	0,000101572	2,65561E-05	26,145	8,1E-05	5,82E-05	6,26E-05	4,96E-05	6,29E-05	6,28495E-05	1,14798E-05	18,265
Q9EQH3	VPS35_MOUSE	Vacuolar protein sorting-associated protein 35	0,000782	0,000859	0,000856	0,000864	0,000840104	3,90491E-05	4,648	0,000811	0,00074	0,000819	0,000777	0,000892	0,000807624	5,67095E-05	7,022
P09405	NUCL_MOUSE	Nucleolin	0,000599	0,00066	0,000673	0,000622	0,000638458	3,43774E-05	5,384	0,000659	0,000591	0,000623	0,000614	0,000713	0,00063967	4,75369E-05	7,431
Q922D8	C1TC_MOUSE	C-1-tetrahydrofolate synthase, cytoplasmic	0,000109	0,000131	0,000111	0,000113	0,000116234	1,02404E-05	8,810	0,000127	0,00014	0,000126	0,000122	0,000139	0,000130743	8,25694E-06	6,315
P80314	TCPB_MOUSE	T-complex protein 1 subunit beta	0,000562	0,000624	0,000593	0,000604	0,000595777	2,61172E-05	4,384	0,00059	0,000557	0,000622	0,000621	0,000595	0,000596971	2,66747E-05	4,468
Q9QXY6	EHD3_MOUSE	EH domain-containing protein 3	7,12E-05	0,000124	0,000116	0,000105	0,000103894	2,31968E-05	22,327	0,000123	9,77E-05	0,000113	0,000115	0,000117	0,000113058	9,27833E-06	8,207
Q8CAA7	PGM2L_MOUSE	Glucose 1,6-bisphosphate synthase	0,000385	0,000453	0,000435	0,000425	0,000424596	2,85593E-05	6,726	0,00045	0,000397	0,000427	0,000385	0,000393	0,000410453	2,74022E-05	6,676
P11499	HS90B_MOUSE	Heat shock protein HSP 90-beta	0,001826	0,002116	0,001823	0,001893	0,001914489	0,000138076	7,212	0,002102	0,002127	0,002025	0,002016	0,002168	0,002087759	6,58606E-05	3,155
P80316	TCPE_MOUSE	T-complex protein 1 subunit epsilon	0,000281	0,000307	0,00027	0,000282	0,000284987	1,53795E-05	5,397	0,000304	0,000274	0,000276	0,000291	0,000274	0,000283913	1,33561E-05	4,704
Q9WU78	PDC6I_MOUSE	Programmed cell death 6-interacting protein	0,000152	0,000168	0,000149	0,00016	0,000157504	8,64528E-06	5,489	0,000161	0,000145	0,000152	0,000149	0,000156	0,000152721	6,19762E-06	4,058
O88569	ROA2_MOUSE	Heterogeneous nuclear ribonucleoproteins A2/B1	0,00093	0,000965	0,000969	0,001034	0,000974325	4,36048E-05	4,475	0,000989	0,000886	0,000954	0,000928	0,000939	0,000939245	3,78937E-05	4,034
Q9EQ20	MMSA_MOUSE	Methylmalonate-semialdehyde dehydrogenase [acylating], mitochondrial	0,000322	0,000304	0,000256	0,000301	0,000295615	2,83852E-05	9,602	0,000274	0,000301	0,000321	0,000318	0,000287	0,000300106	2,0187E-05	6,727
Q8K2B3	SDHA_MOUSE	Succinate dehydrogenase [ubiquinone] flavoprotein subunit, mitochondrial	0,001004	0,00098	0,000929	0,000981	0,000973446	3,16226E-05	3,249	0,000996	0,001049	0,001068	0,000994	0,000993	0,001019904	3,59487E-05	3,525
Q9QYB8	ADDB_MOUSE	Beta-adducin	0,000442	0,000515	0,000475	0,000479	0,000477906	2,96425E-05	6,203	0,00052	0,000447	0,000467	0,000474	0,000511	0,000483886	3,04834E-05	6,300
P62631	EF1A2_MOUSE	Elongation factor 1-alpha 2	0,000652	0,000784	0,000657	0,000746	0,000709947	6,55725E-05	9,236	0,000826	0,000832	0,00082	0,00082	0,000799	0,000819287	1,2225E-05	1,492
Q02248	CTNB1_MOUSE	Catenin beta-1	0,000363	0,000382	0,00037	0,00037	0,000371088	8,21837E-06	2,215	0,000405	0,000399	0,000365	0,000373	0,000387	0,000385604	1,68057E-05	4,358
P20152	VIME_MOUSE	Vimentin	0,000331	0,000459	0,000371	0,000405	0,00039144	5,41269E-05	13,828	0,000385	0,000331	0,000478	0,000388	0,000365	0,000389617	5,43655E-05	13,954
Q9R111	GUAD_MOUSE	Guanine deaminase	0,000659	0,000649	0,000954	0,000717	0,000744905	0,000142422	19,120	0,000629	0,000562	0,000689	0,000736	0,000813	0,00068577	9,65713E-05	14,082
P62259	1433E_MOUSE	14-3-3 protein epsilon	0,002881	0,002653	0,002613	0,002973	0,00277975	0,00017483	6,289	0,003022	0,003347	0,003117	0,003039	0,002509	0,003006792	0,000306746	10,202
Q61553	FSCN1_MOUSE	Fascin	0,000943	0,000863	0,000883	0,000869	0,000889828	3,66994E-05	4,124	0,000912	0,000869	0,000887	0,000934	0,000824	0,000885182	4,24377E-05	4,794
P97807	FUMH_MOUSE	Fumarate hydratase, mitochondrial	0,001384	0,001316	0,001286	0,001332	0,001329411	4,13227E-05	3,108	0,001241	0,001251	0,001332	0,001271	0,001245	0,001267809	3,75914E-05	2,965
Q921I1	TRFE_MOUSE	Serotransferrin	0,000212	0,000172	0,000151	0,00017	0,000176207	2,55029E-05	14,473	0,000149	0,000144	0,00014	0,000121	0,000141	0,000139205	1,09005E-05	7,831
Q60932	VDAC1_MOUSE	Voltage-dependent anion-selective channel protein 1	0,003923	0,003759	0,003837	0,003917	0,003858858	7,73034E-05	2,003	0,00357	0,004006	0,003547	0,003213	0,003203	0,00350774	0,000329043	9,380
Q9Z0E0	NCDN_MOUSE	Neurochondrin	0,001912	0,002388	0,002374	0,002352	0,002256581	0,000230192	10,201	0,002149	0,00192	0,002152	0,00201	0,002393	0,002125004	0,000179083	8,427
Q8VEK3	HNRPU_MOUSE	Heterogeneous nuclear ribonucleoprotein U	0,000225	0,000283	0,000248	0,000252	0,000251996	2,37626E-05	9,430	0,000309	0,000317	0,000322	0,000323	0,000407	0,00033559	4,018E-05	11,973
Q9D0F9	PGM1_MOUSE	Phosphoglucomutase-1	0,000869	0,00103	0,001254	0,001261	0,001103318	0,000189401	17,166	0,000836	0,000843	0,001043	0,000986	0,000962	0,000933975	9,12096E-05	9,766
P63005	LIS1_MOUSE	Platelet-activating factor acetylhydrolase IB subunit alpha	0,000328	0,00031	0,000319	0,000299	0,000314192	1,2538E-05	3,991	0,000328	0,000289	0,000301	0,000305	0,000302	0,000305107	1,44377E-05	4,732
Q9R0P9	UCHL1_MOUSE	Ubiquitin carboxyl-terminal hydrolase isozyme L1	0,000663	0,000656	0,000757	0,000812	0,000722232	7,58914E-05	10,508	0,000566	0,000523	0,000695	0,000578	0,000585	0,000589372	6,3904E-05	10,843
P11983	TCPA_MOUSE	T-complex protein 1 subunit alpha	0,000612	0,000698	0,000652	0,000702	0,000661	4,57423E-05	6,919	0,000683	0,000652	0,000711	0,000765	0,000684	0,000699098	4,24305E-05	6,069
Q9Z1S5	SEPT3_MOUSE	Neuronal-specific septin-3	0,001022	0,000979	0,000963	0,000974	0,000984188	2,58724E-05	2,629	0,001106	0,000975	0,000949	0,000998	0,000855	0,000976631	9,04951E-05	9,266
P55012	S12A2_MOUSE	Solute carrier family 12 member 2	5,41E-05	5,12E-05	3,97E-05	5,28E-05	4,94667E-05	6,61919E-06	13,381	4,41E-05	4,54E-05	5,78E-05	4,16E-05	4,34E-05	4,64782E-05	6,49753E-06	13,980
Q62188	DPYL3_MOUSE	Dihydropyrimidinase-related protein 3	0,000814	0,000963	0,000852	0,000987	0,000904144	8,41629E-05	9,309	0,001088	0,001038	0,001114	0,001175	0,001055	0,001093808	5,39923E-05	4,936
Q9Z2Q6	SEPT5_MOUSE	Septin-5	0,000699	0,000596	0,000664	0,000625	0,000645799	4,49708E-05	6,964	0,000695	0,000593	0,00062	0,000604	0,000602	0,000622635	4,15893E-05	6,680
Q80U28	MADD_MOUSE	MAP kinase-activating death domain protein	0,000134	0,00015	0,00014	0,000155	0,000145048	9,6529E-06	6,655	0,000158	0,000116	9,93E-05	0,000135	0,000144	0,000130522	2,33455E-05	17,886
Q8BMS1	ECHA_MOUSE	Trifunctional enzyme subunit alpha, mitochondrial	0,000649	0,00068	0,000552	0,000652	0,000633005	5,59609E-05	8,841	0,000635	0,000762	0,000705	0,000646	0,000647	0,000678863	5,41787E-05	7,981
Q9CZ30	OLA1_MOUSE	Obg-like ATPase 1	0,000257	0,000309	0,000273	0,000315	0,000288523	2,77785E-05	9,628	0,000282	0,000227	0,000262	0,000299	0,00028	0,000269749	2,74134E-05	10,163
Q8K596	NAC2_MOUSE	Sodium/calcium exchanger 2	0,000337	0,000308	0,000339	0,000349	0,000333142	1,77225E-05	5,320	0,000377	0,000309	0,000315	0,000315	0,000363	0,000335567	3,16852E-05	9,442
O88844	IDHC_MOUSE	Isocitrate dehydrogenase [NADP] cytoplasmic	0,000147	0,00015	0,000169	0,000176	0,00016068	1,39402E-05	8,676	0,00014	0,00012	0,000157	0,000146	0,000152	0,000143157	1,43299E-05	10,010
P35235	PTN11_MOUSE	Tyrosine-protein phosphatase non-receptor type 11	0,000123	0,00014	0,000113	0,000124	0,000125045	1,08364E-05	8,666	0,000132	0,000131	0,000128	0,000135	0,000127	0,000130512	3,30123E-06	2,529
O08539	BIN1_MOUSE	Myc box-dependent-interacting protein 1	0,000931	0,00094	0,000827	0,00088	0,000894359	5,24011E-05	5,859	0,000969	0,000839	0,00097	0,001027	0,000961	0,000953158	6,88551E-05	7,224
P84091	AP2M1_MOUSE	AP-2 complex subunit mu	0,00048	0,000489	0,000525	0,000496	0,000497306	1,96875E-05	3,959	0,000533	0,000458	0,000525	0,000525	0,000528	0,000513637	3,14757E-05	6,128
Q8CGY8	OGT1_MOUSE	UDP-N-acetylglucosamine--peptide N-acetylglucosaminyltransferase 110 kDa subunit	7,95E-05	8,38E-05	9,19E-05	8,79E-05	8,5771E-05	5,35423E-06	6,242	8,41E-05	7,11E-05	8,24E-05	7,88E-05	8,63E-05	8,05178E-05	5,97028E-06	7,415
Q91YT0	NDUV1_MOUSE	NADH dehydrogenase [ubiquinone] flavoprotein 1, mitochondrial	0,000976	0,000912	0,00088	0,000898	0,000916819	4,1862E-05	4,566	0,000796	0,000813	0,00087	0,000815	0,000784	0,000815513	3,2988E-05	4,045
P70168	IMB1_MOUSE	Importin subunit beta-1	0,000374	0,000398	0,000375	0,000355	0,000375648	1,75265E-05	4,666	0,000401	0,000388	0,000396	0,000394	0,000422	0,00040033	1,29162E-05	3,226
P80315	TCPD_MOUSE	T-complex protein 1 subunit delta	0,000325	0,00033	0,000331	0,000262	0,000312004	3,31358E-05	10,620	0,000321	0,000314	0,000341	0,000308	0,000331	0,000323142	1,32046E-05	4,086
P48962	ADT1_MOUSE	ADP/ATP translocase 1	0,004825	0,004074	0,00403	0,004096	0,004256266	0,000380058	8,929	0,00348	0,003622	0,003762	0,003531	0,003168	0,003512507	0,000220357	6,273
Q80TZ3	AUXI_MOUSE	Putative tyrosine-protein phosphatase auxilin	0,000302	0,000303	0,000315	0,000319	0,000309576	8,39513E-06	2,712	0,000343	0,00033	0,000318	0,000326	0,000357	0,000334679	1,52378E-05	4,553
P61264	STX1B_MOUSE	Syntaxin-1B	0,001803	0,001521	0,001446	0,001762	0,001633012	0,000176047	10,781	0,001731	0,001697	0,00169	0,001801	0,001536	0,001690988	9,72737E-05	5,752
Q6NXK7	DPP10_MOUSE	Inactive dipeptidyl peptidase 10	0,00036	0,000382	0,000429	0,00035	0,000380328	3,50954E-05	9,228	0,000425	0,000411	0,000366	0,000388	0,000413	0,000400515	2,38484E-05	5,954
P14211	CALR_MOUSE	Calreticulin	0,000822	0,000872	0,000793	0,000873	0,000840074	3,92611E-05	4,674	0,000802	0,000818	0,000841	0,000803	0,000829653	3,41795E-05	4,120
Q8VDQ8	SIR2_MOUSE	NAD-dependent protein deacetylase sirtuin-2	0,000668	0,000608	0,00048	0,000641	0,000599217	8,3498E-05	13,935	0,000692	0,000745	0,00073	0,000676	0,000547	0,000677831	7,8334E-05	11,557
Q9DBF1	AL7A1_MOUSE	Alpha-aminoadipic semialdehyde dehydrogenase	0,000438	0,000451	0,00043	0,000426	0,000436242	1,09834E-05	2,518	0,000457	0,000524	0,000472	0,000453	0,000451	0,000471409	3,01733E-05	6,401
Q8BFR5	EFTU_MOUSE	Elongation factor Tu, mitochondrial	0,000861	0,000869	0,000848	0,000816	0,00084874	2,32911E-05	2,744	0,000848	0,000814	0,000826	0,000809	0,000815	0,000822322	1,53965E-05	1,872
P10649	GSTM1_MOUSE	Glutathione S-transferase Mu 1	0,001516	0,001673	0,001594	0,001735	0,001629458	9,48632E-05	5,822	0,001565	0,002062	0,001903	0,001697	0,001539	0,001753077	0,00022473	12,819
P48036	ANXA5_MOUSE	Annexin A5	0,000563	0,000538	0,000454	0,000528	0,000520701	4,70823E-05	9,042	0,000539	0,000497	0,000556	0,00049	0,000427	0,000502025	5,02828E-05	10,016
P18760	COF1_MOUSE	Cofilin-1	0,001327	0,001441	0,001327	0,00148	0,001393917	7,8595E-05	5,638	0,001479	0,001545	0,001443	0,001582	0,001528	0,001515402	5,48523E-05	3,620
Q8BMF4	ODP2_MOUSE	Dihydrolipoyllysine-residue acetyltransferase component of pyruvate dehydrogenase [illegible]	0,001398	0,001433	0,001294	0,001347	0,001367857	6,05095E-05	4,424	0,00128	0,00116	0,001274	0,001226	0,001279	0,00124377	5,19161E-05	4,174
P06151	LDHA_MOUSE	L-lactate dehydrogenase A chain	0,001913	0,001747	0,00191	0,001982	0,001888344	9,97945E-05	5,285	0,001844	0,001743	0,001664	0,001571	0,001439	0,001652257	0,000155733	9,425
O35643	AP1B1_MOUSE	AP-1 complex subunit beta-1	0,000438	0,000446	0,000451	0,000441	0,000443747	5,63248E-06	1,269	0,000499	0,000436	0,000487	0,000521	0,000522	0,000492933	3,49491E-05	7,090

O08749	DLDH_MOUSE	Dihydrolipoyl dehydrogenase, mitochondrial	0,002279	0,002369	0,002176	0,002187	0,002252688	9,01662E-05	4,003	0,002315	0,00221	0,002193	0,002282	0,002232	0,002246577	5,08048E-05	2,261
P09103	PDIA1_MOUSE	Protein disulfide-isomerase	0,000323	0,000355	0,000322	0,000336	0,000334114	1,54885E-05	4,636	0,000365	0,000229	0,000368	0,000308	0,000362	0,000326277	6,00366E-05	18,400
Q00612	G6PD1_MOUSE	Glucose-6-phosphate 1-dehydrogenase X	0,000298	0,00031	0,000269	0,000286	0,000290576	1,76072E-05	6,059	0,000278	0,000271	0,000282	0,000307	0,000247	0,000277149	2,1443E-05	7,737
Q9JMA1	UBP14_MOUSE	Ubiquitin carboxyl-terminal hydrolase 14	5,34E-05	7,13E-05	5,61E-05	6,23E-05	6,07725E-05	7,93843E-06	13,063	5,77E-05	6,28E-05	7,23E-05	8,19E-05	6,91E-05	6,87544E-05	9,25234E-06	13,457
P10637	TAU_MOUSE	Microtubule-associated protein tau	0,000662	0,000681	0,000628	0,00067	0,000660138	2,27365E-05	3,444	0,000685	0,000645	0,000617	0,000568	0,000647	0,000632545	4,33625E-05	6,855
Q9D6R2	IDH3A_MOUSE	Isocitrate dehydrogenase [NAD] subunit alpha, mitochondrial	0,001158	0,001181	0,001193	0,001233	0,001191183	3,15962E-05	2,653	0,001192	0,001167	0,001227	0,001193	0,001142	0,001184155	3,16718E-05	2,675
Q8QZT1	THIL_MOUSE	Acetyl-CoA acetyltransferase, mitochondrial	0,001027	0,001102	0,001064	0,001101	0,001073389	3,56462E-05	3,321	0,00113	0,001073	0,001091	0,001088	0,001154	0,001107364	3,38057E-05	3,053
Q9CVB6	ARPC2_MOUSE	Actin-related protein 2/3 complex subunit 2	0,000336	0,00048	0,000462	0,000555	0,000458221	9,11072E-05	19,883	0,000463	0,000438	0,00052	0,000487	0,000454	0,0004723	3,17346E-05	6,719
P08249	MDHM_MOUSE	Malate dehydrogenase, mitochondrial	0,004674	0,004543	0,004636	0,005084	0,004734108	0,000239552	5,060	0,004348	0,005122	0,004351	0,004267	0,004392	0,004496	0,000352722	7,845
Q62420	SH3G2_MOUSE	Endophilin-A1	0,00044	0,000383	0,000376	0,000404	0,00040055	2,87036E-05	7,166	0,000479	0,000429	0,000469	0,000456	0,000443	0,000455327	2,01277E-05	4,421
Q8C1B7	SEP11_MOUSE	Septin-11	0,000421	0,000358	0,000389	0,000331	0,000374567	3,91041E-05	10,440	0,000401	0,000385	0,000375	0,000369	0,000383	0,000382479	1,18471E-05	3,097
P30275	KCRU_MOUSE	Creatine kinase U-type, mitochondrial	0,002002	0,00195	0,00185	0,002009	0,001952845	7,32888E-05	3,753	0,002103	0,001811	0,002072	0,002116	0,001928	0,002005912	0,000132083	6,585
P47708	RP3A_MOUSE	Rabphilin-3A	0,000121	0,000111	0,0001	0,000103	0,000108898	9,29552E-06	8,536	0,000138	0,000124	0,000111	0,000101	0,000105	0,000115708	1,53529E-05	13,269
O88643	PAK1_MOUSE	Serine/threonine-protein kinase PAK 1	0,000263	0,000291	0,000274	0,000264	0,000273057	1,29669E-05	4,749	0,000305	0,000281	0,000257	0,000258	0,000245	0,00026924	2,3854E-05	8,860
Q3UHL1	CAMKV_MOUSE	CaM kinase-like vesicle-associated protein	0,000444	0,000527	0,000602	0,000627	0,000550091	8,21634E-05	14,936	0,000521	0,000431	0,00047	0,000517	0,0007	0,000528001	0,000102962	19,500
P53810	PIPNA_MOUSE	Phosphatidylinositol transfer protein alpha isoform	0,000182	0,000199	0,000183	0,000232	0,000198937	2,33819E-05	11,753	0,000212	0,000174	0,000199	0,000194	0,000163	0,000188465	1,9528E-05	10,362
Q9D819	IPYR_MOUSE	Inorganic pyrophosphatase	0,000285	0,000283	0,000303	0,000323	0,000298333	1,88519E-05	6,319	0,0003	0,000275	0,00029	0,000268	0,00025	0,000276613	1,96643E-05	7,109
P18872	GNAO_MOUSE	Guanine nucleotide-binding protein G(o) subunit alpha	0,001146	0,001146	0,001301	0,001276	0,001217335	8,27096E-05	6,794	0,001246	0,001248	0,001305	0,001278	0,001228	0,001261017	3,01574E-05	2,392
B9EKR1	PRPTZ_MOUSE	Receptor-type tyrosine-protein phosphatase zeta	0,000595	0,00067	0,000656	0,000675	0,000648981	3,71577E-05	5,726	0,000674	0,000652	0,000638	0,000574	0,00061	0,000629345	3,89263E-05	6,185
Q9R0Q6	ARC1A_MOUSE	Actin-related protein 2/3 complex subunit 1A	9,83E-05	9,39E-05	9,9E-05	0,000103	9,84311E-05	3,56913E-06	3,626	9,2E-05	7,61E-05	7,99E-05	8,09E-05	7,43E-05	8,06444E-05	6,91996E-06	8,581
Q7TQF7	AMPH_MOUSE	Amphiphysin	0,001276	0,001361	0,001495	0,00136	0,00137322	9,05606E-05	6,595	0,001432	0,001216	0,001232	0,001159	0,001414	0,001290458	0,000124057	9,613
P80313	TCPH_MOUSE	T-complex protein 1 subunit eta	0,000335	0,000403	0,00035	0,000366	0,000363737	2,93006E-05	8,055	0,000359	0,000315	0,000395	0,000375	0,000342	0,000357106	3,03262E-05	8,492
Q8VDM4	PSMD2_MOUSE	26S proteasome non-ATPase regulatory subunit 2	0,000167	0,000195	0,000201	0,000196	0,000189569	1,54878E-05	8,170	0,000204	0,000197	0,000211	0,000213	0,000222	0,000209244	9,18213E-06	4,388
Q9DBJ1	PGAM1_MOUSE	Phosphoglycerate mutase 1	0,001887	0,001665	0,002232	0,002389	0,002043152	0,000327823	16,045	0,001654	0,001801	0,001851	0,001905	0,001753	0,001792852	9,60764E-05	5,359
P17183	ENOG_MOUSE	Gamma-enolase	0,002905	0,00302	0,004168	0,003937	0,003507323	0,000638159	18,195	0,002764	0,002336	0,002826	0,003272	0,002999	0,002839484	0,000343299	12,090
P63101	1433Z_MOUSE	14-3-3 protein zeta/delta	0,002391	0,002126	0,002163	0,002222	0,002225522	0,00011738	5,274	0,002384	0,002524	0,002254	0,002251	0,002259	0,002334403	0,00012003	5,142
P28663	SNAB_MOUSE	Beta-soluble NSF attachment protein	0,000857	0,000899	0,001051	0,001039	0,000961375	9,79232E-05	10,186	0,000806	0,000827	0,000952	0,000908	0,000848	0,000868013	6,0385E-05	6,957
P32921	SYWC_MOUSE	Tryptophan--tRNA ligase, cytoplasmic	0,000164	0,00019	0,000165	0,000161	0,000170035	1,35006E-05	7,940	0,000174	0,000183	0,000178	0,000167	0,000181	0,000176789	6,31025E-06	3,569
Q9DC69	NDUA9_MOUSE	NADH dehydrogenase [ubiquinone] 1 alpha subcomplex subunit 9, mitochondrial	0,001007	0,000925	0,000924	0,001043	0,000974551	5,97289E-05	6,129	0,000805	0,000842	0,000824	0,000711	0,000589	0,000754062	0,000105222	13,954
Q62059	CSPG2_MOUSE	Versican core protein	0,0002	0,00022	0,000195	0,000198	0,000203312	1,11918E-05	5,505	0,000176	0,000138	0,000172	0,000191	0,000227	0,000181	3,21813E-05	17,780
O89053	COR1A_MOUSE	Coronin-1A	0,000246	0,000256	0,000279	0,000254	0,000258992	1,39681E-05	5,393	0,000264	0,00023	0,000235	0,000242	0,000255	0,000245023	1,43176E-05	5,843
Q9D154	ILEUA_MOUSE	Leukocyte elastase inhibitor A	0,000195	0,000178	0,000144	0,000158	0,000168757	2,26866E-05	13,443	0,000205	0,000184	0,000176	0,000175	0,00015	0,000177982	1,97593E-05	11,102
O54774	AP3D1_MOUSE	AP-3 complex subunit delta-1	7,24E-05	9,98E-05	0,000105	0,000112	9,7311E-05	1,74218E-05	17,903	0,000133	0,000117	0,000121	0,000136	0,000142	0,000129958	1,02536E-05	7,890
Q9QYG0	NDRG2_MOUSE	Protein NDRG2	0,001306	0,001393	0,001179	0,001258	0,001283917	8,94431E-05	6,966	0,001277	0,00159	0,001209	0,001218	0,001115	0,001281899	0,00018191	14,191
Q9DB77	QCR2_MOUSE	Cytochrome b-c1 complex subunit 2, mitochondrial	0,001797	0,001599	0,001654	0,001578	0,001657193	9,87622E-05	5,960	0,001382	0,001487	0,001465	0,001407	0,001433	0,001434742	4,25581E-05	2,966
P29341	PABP1_MOUSE	Polyadenylate-binding protein 1	0,000246	0,000297	0,000279	0,000276	0,000274827	2,10808E-05	7,671	0,000292	0,000303	0,000289	0,000292	0,000291	0,000293186	5,54879E-06	1,893
Q922B2	SYDC_MOUSE	Aspartate--tRNA ligase, cytoplasmic	0,000262	0,000284	0,000273	0,000277	0,000274005	8,99924E-06	3,284	0,000266	0,000252	0,000268	0,000272	0,000255	0,000262645	8,44993E-06	3,217
Q9CQQ7	AT5F1_MOUSE	ATP synthase F(0) complex subunit B1, mitochondrial	0,001238	0,001267	0,001174	0,001301	0,001245171	5,37628E-05	4,318	0,001097	0,001202	0,001251	0,001202	0,001101	0,001170668	6,81602E-05	5,822
Q91V12	BACH_MOUSE	Cytosolic acyl coenzyme A thioester hydrolase	0,002243	0,002303	0,00216	0,002256	0,002240335	5,95514E-05	2,658	0,00232	0,001999	0,002074	0,002094	0,001987	0,002094632	0,000134364	6,415
Q99JY9	ARP3_MOUSE	Actin-related protein 3	0,000418	0,000502	0,000489	0,000536	0,000486245	4,97705E-05	10,236	0,000468	0,000441	0,000505	0,000528	0,000532	0,000494741	3,95717E-05	7,998
Q9CZC8	SCRN1_MOUSE	Secernin-1	0,000596	0,000516	0,00065	0,000611	0,000592946	5,61484E-05	9,469	0,000522	0,000503	0,000552	0,000533	0,000522	0,000526277	1,79224E-05	3,406
Q91WD5	NDUS2_MOUSE	NADH dehydrogenase [ubiquinone] iron-sulfur protein 2, mitochondrial	0,000682	0,000655	0,000652	0,000632	0,000655155	2,0697E-05	3,159	0,00061	0,000557	0,000652	0,000635	0,000661	0,00062325	4,18679E-05	6,718
P16125	LDHB_MOUSE	L-lactate dehydrogenase B chain	0,003539	0,00318	0,003145	0,003386	0,003312644	0,000184648	5,574	0,003513	0,003324	0,003339	0,003194	0,002912	0,003256138	0,000223495	6,864
P10852	4F2_MOUSE	4F2 cell-surface antigen heavy chain	0,000893	0,000972	0,000838	0,000938	0,000910252	5,79351E-05	6,365	0,000987	0,000772	0,000924	0,000926	0,000912	0,000904342	7,94696E-05	8,788
Q9CPV4	GLOD4_MOUSE	Glyoxalase domain-containing protein 4	0,000394	0,000394	0,000405	0,000438	0,000407591	2,08825E-05	5,123	0,000415	0,000387	0,000437	0,00041	0,000387	0,00040714	2,11856E-05	5,204
P04370	MBP_MOUSE	Myelin basic protein	0,006619	0,007166	0,0054	0,006884	0,006517329	0,000777684	11,933	0,007239	0,007118	0,007329	0,007394	0,006135	0,007043124	0,000518196	7,357
P17751	TPIS_MOUSE	Triosephosphate isomerase	0,002148	0,002072	0,002686	0,002828	0,002433428	0,00037929	15,587	0,001772	0,002128	0,002117	0,002019	0,001956	0,001998303	0,000145347	7,273
Q9Z268	RASL1_MOUSE	RasGAP-activating-like protein 1	0,000126	0,000126	0,000158	0,000144	0,000138459	1,53138E-05	11,060	0,000154	0,000128	0,000128	0,000138	0,000156	0,000140961	1,3351E-05	9,471
P14152	MDHC_MOUSE	Malate dehydrogenase, cytoplasmic	0,007952	0,007589	0,007537	0,008022	0,007774866	0,000247387	3,182	0,008124	0,007785	0,007974	0,007741	0,006902	0,007704942	0,000474473	6,158
Q8BVE3	VATH_MOUSE	V-type proton ATPase subunit H	0,000556	0,0006	0,000613	0,000593	0,000590645	2,48191E-05	4,202	0,000555	0,000548	0,00058	0,000578	0,000601	0,000572242	2,11646E-05	3,699
P63328	PP2BA_MOUSE	Serine/threonine-protein phosphatase 2B catalytic subunit alpha isoform	0,001146	0,001143	0,001124	0,001152	0,001141402	1,20686E-05	1,057	0,001208	0,000995	0,00116	0,001292	0,001346	0,001200353	0,000135521	11,290
P12382	PFKAL_MOUSE	ATP-dependent 6-phosphofructokinase, liver type	0,000539	0,000562	0,000543	0,000552	0,000549137	1,00229E-05	1,825	0,00058	0,000526	0,000536	0,000526	0,000612	0,000556097	3,86188E-05	6,945
Q9JIA1	LGI1_MOUSE	Leucine-rich glioma-inactivated protein 1	0,000254	0,000257	0,000264	0,000261	0,00025894	4,41819E-06	1,706	0,000237	0,00024	0,000251	0,000257	0,000274	0,000251859	1,51081E-05	5,999
Q91XV3	BASP1_MOUSE	Brain acid soluble protein 1	0,000342	0,000414	0,000334	0,000381	0,000367725	3,70074E-05	10,064	0,000374	0,000493	0,000381	0,000516	0,000485	0,00044977	6,68315E-05	14,859
Q8VEM8	MPCP_MOUSE	Phosphate carrier protein, mitochondrial	0,003686	0,003173	0,00312	0,002966	0,003236347	0,000312228	9,648	0,002882	0,00302	0,002904	0,002796	0,002711	0,002862695	0,000116579	4,072
P31938	MP2K1_MOUSE	Dual specificity mitogen-activated protein kinase kinase 1	0,000313	0,000358	0,000391	0,000373	0,000358698	3,34066E-05	9,313	0,000326	0,000326	0,000357	0,00035	0,00036	0,00034374	1,65827E-05	4,824
P40124	CAP1_MOUSE	Adenylyl cyclase-associated protein 1	0,000393	0,000427	0,000439	0,000405	0,000415822	2,08718E-05	5,019	0,000437	0,000409	0,000436	0,000486	0,000504	0,000454834	3,92461E-05	8,629
Q91YQ5	RPN1_MOUSE	Dolichyl-diphosphooligosaccharide--protein glycosyltransferase subunit 1	0,000199	0,000194	0,00021	0,000217	0,000204966	1,05967E-05	5,170	0,000202	0,000213	0,000215	0,000197	0,000215	0,000208798	8,37761E-06	4,012
Q9CZW5	TOM70_MOUSE	Mitochondrial import receptor subunit TOM70	0,000836	0,000792	0,000846	0,000776	0,000812667	3,36499E-05	4,141	0,000813	0,000759	0,000806	0,000869	0,000945	0,000838378	7,12022E-05	8,493
Q9D1A2	CNDP2_MOUSE	Cytosolic non-specific dipeptidase	0,000275	0,000282	0,000287	0,000271	0,000278705	7,08142E-06	2,541	0,000286	0,000269	0,000292	0,000296	0,000274	0,000283481	1,17661E-05	4,151
Q08331	CALB2_MOUSE	Calretinin	0,000343	0,000546	0,000471	0,000532	0,000473104	9,2258E-05	19,501	0,000488	0,000502	0,000515	0,00047	0,000403	0,000475744	4,38862E-05	9,225
Q8R366	IGSF8_MOUSE	Immunoglobulin superfamily member 8	0,001124	0,001235	0,001149	0,001192	0,001175145	4,90042E-05	4,170	0,001299	0,001383	0,001386	0,001336	0,001306	0,001341743	4,11666E-05	3,068
Q99PU5	ACBG1_MOUSE	Long-chain-fatty-acid--CoA ligase ACSBG1	0,000299	0,000311	0,000257	0,0003	0,000291825	2,37812E-05	8,149	0,000291	0,000344	0,000293	0,000296	0,000284	0,000301579	2,38649E-05	7,913
Q9R1V6	ADA22_MOUSE	Disintegrin and metalloproteinase domain-containing protein 22	0,000138	0,000138	0,000147	0,000114	0,000134187	1,44286E-05	10,753	0,000126	0,000121	0,000124	0,000109	0,000137	0,000123421	1,03192E-05	8,361
Q9JIS5	SV2A_MOUSE	Synaptic vesicle glycoprotein 2A	0,000737	0,000782	0,000711	0,000764	0,000748363	3,09278E-05	4,133	0,00093	0,000869	0,000836	0,000838	0,000933	0,000881331	4,76847E-05	5,411
P62702	RS4X_MOUSE	40S ribosomal protein S4, X isoform	0,000231	0,000212	0,000234	0,000271	0,000236895	2,468E-05	10,418	0,000245	0,000203	0,00026	0,000225	0,000213	0,000229386	2,32748E-05	10,147
Q99K85	SERC_MOUSE	Phosphoserine aminotransferase	0,000958	0,000842	0,000912	0,000961	0,000918457	5,54144E-05	6,033	0,000955	0,000777	0,000945	0,000914	0,000776	0,000873325	8,96402E-05	10,264
P02088	HBB1_MOUSE	Hemoglobin subunit beta-1	0,016287	0,008043	0,010281	0,011518	0,011532066	0,003480712	30,183	0,008529	0,00744	0,006685	0,005328	0,006296	0,006855783	0,001206029	17,591
Q01065	PDE1B_MOUSE	Calcium/calmodulin-dependent 3',5'-cyclic nucleotide phosphodiesterase 1B	0,000499	0,000423	0,000502	0,000397	0,000455137	5,31911E-05	11,687	0,000499	0,000376	0,000445	0,000583	0,000661	0,000512833	0,000112277	21,893
P22892	AP1G1_MOUSE	AP-1 complex subunit gamma-1	0,000215	0,000229	0,000222	0,000228	0,000223523	6,73776E-06	3,014	0,000218	0,000204	0,000207	0,000198	0,000221	0,000209708	9,62494E-06	4,590
Q9CPY7	AMPL_MOUSE	Cytosol aminopeptidase	0,000569	0,000576	0,000508	0,00055	0,00055056	3,04375E-05	5,528	0,000559	0,000546	0,000583	0,000564	0,000499	0,000550222	3,14739E-05	5,720

P80317	TCPZ_MOUSE	T-complex protein 1 subunit zeta	0,000284	0,00033	0,000289	0,000327	0,000307403	2,44745E-05	7,962	0,000303	0,000297	0,00032	0,000297	0,0003	0,000303349	9,62813E-06	3,174
Q921M7	FA49B_MOUSE	Protein FAM49B	0,000779	0,000693	0,000704	0,000695	0,000717623	4,125E-05	5,748	0,000769	0,00076	0,000701	0,00065	0,00062	0,000700132	6,59823E-05	9,424
Q8CGP1	H2B1K_MOUSE	Histone H2B type 1-K	0,00016	0,000213	0,000188	0,000215	0,000194038	2,5997E-05	13,398	0,000173	0,0002	0,000241	0,000185	0,000197	0,000199318	2,57176E-05	12,903
Q80TL4	PHF24_MOUSE	PHD finger protein 24	0,00031	0,000284	0,000363	0,00029	0,000311729	3,58961E-05	11,515	0,000273	0,000238	0,000289	0,000278	0,000307	0,000276856	2,5401E-05	9,175
Q99JY0	ECHB_MOUSE	Trifunctional enzyme subunit beta, mitochondrial	0,00056	0,000558	0,000532	0,000561	0,000552735	1,38745E-05	2,510	0,000473	0,00057	0,000559	0,00049	0,000475	0,000513512	4,74415E-05	9,239
Q3UM45	PP1R7_MOUSE	Protein phosphatase 1 regulatory subunit 7	0,00018	0,000177	0,000188	0,000179	0,00018092	4,78799E-06	2,646	0,000189	0,00017	0,000205	0,000213	0,000212	0,000197652	1,82709E-05	9,244
Q99LX0	PARK7_MOUSE	Protein DJ-1	0,000564	0,000592	0,000559	0,000663	0,000594475	4,78503E-05	8,049	0,000622	0,000621	0,000727	0,000688	0,000614	0,000654632	5,06131E-05	7,732
Q9Z2I0	LETM1_MOUSE	LETM1 and EF-hand domain-containing protein 1, mitochondrial	0,000119	0,000126	0,000128	0,000113	0,000121566	6,72059E-06	5,528	0,000114	0,000113	0,000111	0,000109	0,000114	0,000112331	1,95896E-06	1,744
Q6P1F6	2ABA_MOUSE	Serine/threonine-protein phosphatase 2A 55 kDa regulatory subunit B alpha isoform	0,00025	0,000258	0,000276	0,000249	0,000258024	1,24901E-05	4,841	0,000272	0,000259	0,000294	0,000304	0,000304	0,000286451	2,01315E-05	7,028
Q8K0T0	RTN1_MOUSE	Reticulon-1	0,000779	0,000882	0,000857	0,000776	0,000823648	5,42264E-05	6,584	0,000834	0,000835	0,000858	0,000831	0,0008	0,00083195	2,06913E-05	2,487
P67778	PHB_MOUSE	Prohibitin	0,000908	0,000843	0,000841	0,000828	0,000854977	3,61719E-05	4,231	0,000775	0,000817	0,000798	0,000721	0,000706	0,000763288	4,81472E-05	6,308
Q9DCN2	NB5R3_MOUSE	NADH-cytochrome b5 reductase 3	0,000108	0,000116	0,000131	0,000125	0,000120051	1,02839E-05	8,566	9,62E-05	0,000109	0,000107	8,55E-05	7,87E-05	9,51883E-05	1,31015E-05	13,764
P48774	GSTM5_MOUSE	Glutathione S-transferase Mu 5	0,000413	0,000412	0,000446	0,000492	0,00044063	3,76121E-05	8,536	0,000479	0,000454	0,000604	0,000561	0,000475	0,000514739	6,43907E-05	12,509
P26638	SYSC_MOUSE	Serine--tRNA ligase, cytoplasmic	0,000461	0,000487	0,000466	0,000449	0,000465527	1,58416E-05	3,403	0,000496	0,000386	0,000464	0,000442	0,000441	0,000445698	4,0116E-05	9,001
O08709	PRDX6_MOUSE	Peroxiredoxin-6	0,000775	0,000873	0,00088	0,000977	0,000875845	8,25044E-05	9,420	0,000943	0,001005	0,000991	0,000925	0,000901	0,000952875	4,39422E-05	4,612
P50518	VATE1_MOUSE	V-type proton ATPase subunit E 1	0,00052	0,000534	0,000544	0,000563	0,000540357	1,81474E-05	3,358	0,000649	0,00063	0,000671	0,000684	0,000656	0,000657882	2,07784E-05	3,158
P62908	RS3_MOUSE	40S ribosomal protein S3	0,00021	0,000214	0,000224	0,000231	0,000219727	9,55984E-06	4,351	0,000225	0,000231	0,000173	0,000228	0,000228	0,000217	2,45798E-05	11,327
Q9D051	ODPB_MOUSE	Pyruvate dehydrogenase E1 component subunit beta, mitochondrial	0,001828	0,001774	0,001723	0,001807	0,001782854	4,57917E-05	2,568	0,001837	0,001778	0,001867	0,001884	0,001727	0,001818732	6,54836E-05	3,601
P51150	RAB7A_MOUSE	Ras-related protein Rab-7a	0,000716	0,000735	0,000742	0,000741	0,000733376	1,2083E-05	1,648	0,000727	0,000741	0,000754	0,000744	0,000656	0,000724299	3,93382E-05	5,431
P47757	CAPZB_MOUSE	F-actin-capping protein subunit beta	0,00047	0,000514	0,00053	0,000598	0,00052808	5,33658E-05	10,106	0,000525	0,000553	0,000588	0,000586	0,000569	0,000564107	2,6129E-05	4,632
P68040	RACK1_MOUSE	Receptor of activated protein C kinase 1	0,000305	0,000317	0,000322	0,00033	0,000318554	1,05704E-05	3,318	0,00031	0,000308	0,0003	0,000258	0,000233	0,000281964	3,43331E-05	12,176
Q62167	DDX3X_MOUSE	ATP-dependent RNA helicase DDX3X	4,46E-05	5,13E-05	5,24E-05	5,72E-05	5,13769E-05	5,15724E-06	10,038	4,85E-05	4,71E-05	5,18E-05	4,58E-05	5,14E-05	4,89214E-05	2,64122E-06	5,399
Q99LC3	NDUAA_MOUSE	NADH dehydrogenase [ubiquinone] 1 alpha subcomplex subunit 10, mitochondrial	0,000823	0,000788	0,000789	0,000767	0,000792018	2,29823E-05	2,902	0,000696	0,000617	0,000679	0,000655	0,000543	0,000638017	6,09464E-05	9,552
P35700	PRDX1_MOUSE	Peroxiredoxin-1	0,001003	0,001	0,000975	0,001023	0,001000416	1,97911E-05	1,978	0,001003	0,001104	0,001138	0,001059	0,000882	0,001037284	0,00010066	9,704
Q80TB8	VAT1L_MOUSE	Synaptic vesicle membrane protein VAT-1 homolog-like	0,000698	0,000698	0,00056	0,000661	0,000654195	6,5452E-05	10,005	0,000583	0,000519	0,000688	0,000835	0,000614	0,000647984	0,000121129	18,693
P42669	PURA_MOUSE	Transcriptional activator protein Pur-alpha	0,000627	0,000738	0,000618	0,000696	0,00066985	5,69499E-05	8,502	0,000675	0,000657	0,000658	0,000667	0,000623	0,000655973	2,00729E-05	3,060
P61979	HNRPK_MOUSE	Heterogeneous nuclear ribonucleoprotein K	0,001219	0,001463	0,001464	0,001454	0,001400231	0,000120958	8,638	0,001356	0,001194	0,001441	0,001356	0,001402	0,001349759	9,38823E-05	6,955
O35129	PHB2_MOUSE	Prohibitin-2	0,001308	0,001222	0,00124	0,00128	0,001262226	3,87321E-05	3,069	0,001182	0,001162	0,00121	0,001144	0,001079	0,00115539	4,9357E-05	4,272
P62761	VISL1_MOUSE	Visinin-like protein 1	0,000914	0,00095	0,000955	0,000968	0,000946983	2,29812E-05	2,427	0,000863	0,000941	0,001034	0,000996	0,000806	0,00092806	9,35445E-05	10,080
P14094	AT1B1_MOUSE	Sodium/potassium-transporting ATPase subunit beta-1	0,004273	0,004216	0,004205	0,004196	0,004222413	3,47564E-05	0,823	0,00385	0,004297	0,004126	0,004154	0,004198	0,004125024	0,000166606	4,039
Q9D0K2	SCOT1_MOUSE	Succinyl-CoA:3-ketoacid coenzyme A transferase 1, mitochondrial	0,000501	0,000514	0,000544	0,000552	0,000527621	2,44665E-05	4,637	0,000568	0,000576	0,000609	0,000651	0,000605	0,000601924	3,27544E-05	5,442
P48758	CBR1_MOUSE	Carbonyl reductase [NADPH] 1	0,000426	0,000432	0,000443	0,000464	0,0004411	1,67444E-05	3,796	0,000447	0,000462	0,000498	0,000436	0,00045	0,000458655	2,40676E-05	5,247
O08529	CAN2_MOUSE	Calpain-2 catalytic subunit	0,000119	0,000138	0,000114	0,00013	0,000125207	1,05986E-05	8,465	8,76E-05	0,000123	0,00012	0,000125	0,000119	0,000114918	1,54162E-05	13,415
P45376	ALDR_MOUSE	Aldose reductase	0,000155	0,000243	0,00033	0,000275	0,000250818	7,32371E-05	29,199	0,000239	0,000194	0,000214	0,000219	0,000207	0,000214487	1,62534E-05	7,578
Q9CZ13	QCR1_MOUSE	Cytochrome b-c1 complex subunit 1, mitochondrial	0,002663	0,002531	0,002471	0,00249	0,002538851	8,6253E-05	3,397	0,002305	0,002273	0,002477	0,002378	0,00238	0,002362657	7,90656E-05	3,346
P20917	MAG_MOUSE	Myelin-associated glycoprotein	0,001186	0,000939	0,000707	0,000862	0,000923479	0,000199949	21,652	0,000949	0,000892	0,001023	0,00094	0,000823	0,000925584	7,39712E-05	7,992
P50247	SAHH_MOUSE	Adenosylhomocysteinase	0,00044	0,000451	0,000453	0,000462	0,000451811	9,21877E-06	2,040	0,000444	0,000391	0,000451	0,000435	0,000434	0,000430923	2,3537E-05	5,462
Q3UHB1	NT5D3_MOUSE	5'-nucleotidase domain-containing protein 3	0,000137	0,000154	6,75E-05	0,000152	0,000127789	4,09192E-05	32,021	0,000123	0,000108	0,000129	0,000129	0,000102	0,000118582	1,23657E-05	10,428
Q99KJ8	DCTN2_MOUSE	Dynactin subunit 2	0,000321	0,000377	0,000285	0,000351	0,000333865	3,95683E-05	11,852	0,000402	0,000364	0,000375	0,000375	0,000374	0,0003778	1,43725E-05	3,804
Q60930	VDAC2_MOUSE	Voltage-dependent anion-selective channel protein 2	0,002274	0,002214	0,002085	0,002224	0,002199392	8,06896E-05	3,669	0,001907	0,001972	0,002012	0,001756	0,001609	0,001851129	0,000166933	9,018
Q6Q477	AT2B4_MOUSE	Plasma membrane calcium-transporting ATPase 4	6,94E-05	8,77E-05	0,000109	8,89E-05	8,88761E-05	1,6386E-05	18,437	0,000107	0,000102	0,000109	0,000115	0,000131	0,00011295	1,13181E-05	10,020
Q9Z1N5	DX39B_MOUSE	Spliceosome RNA helicase Ddx39b	0,000435	0,000461	0,000501	0,000505	0,000475711	3,36472E-05	7,073	0,000489	0,000413	0,0005	0,000502	0,00052	0,000484769	4,16405E-05	8,590
Q80Y17	L2GL1_MOUSE	Lethal(2) giant larvae protein homolog 1	2,97E-05	4,01E-05	3,15E-05	3,36E-05	3,37106E-05	4,5313E-06	13,442	3,67E-05	3,34E-05	4,21E-05	3,5E-05	3,52E-05	3,648E-05	3,34494E-06	9,169
Q8BLK3	LSAMP_MOUSE	Limbic system-associated membrane protein	0,000484	0,000578	0,000577	0,000584	0,00055603	4,7922E-05	8,619	0,00052	0,000554	0,000576	0,000539	0,000598	0,000557191	3,04658E-05	5,468
P06837	NEUM_MOUSE	Neuromodulin	0,000228	0,000267	0,000209	0,000229	0,000233372	2,44168E-05	10,463	0,000233	0,000239	0,000247	0,000229	0,000265	0,000242603	1,40189E-05	5,779
Q60676	PPP5_MOUSE	Serine/threonine-protein phosphatase 5	9,67E-05	8,75E-05	0,000106	9,15E-05	9,53421E-05	7,82387E-06	8,206	7,71E-05	7,21E-05	7,54E-05	7,59E-05	6,7E-05	7,34927E-05	4,07815E-06	5,549
Q8BGH2	SAM50_MOUSE	Sorting and assembly machinery component 50 homolog	0,000413	0,000367	0,000398	0,00039	0,00039187	1,91835E-05	4,895	0,000334	0,000337	0,000369	0,000342	0,000352	0,000346861	1,43156E-05	4,127
Q9R1Q8	TAGL3_MOUSE	Transgelin-3	0,000106	8,93E-05	7,91E-05	0,000104	9,44279E-05	1,25653E-05	13,307	9,7E-05	0,000107	0,000105	0,0001	7,07E-05	9,60471E-05	1,47526E-05	15,360
Q8BH95	ECHM_MOUSE	Enoyl-CoA hydratase, mitochondrial	0,000119	0,00012	0,000111	0,000134	0,000120888	9,74042E-06	8,057	0,000134	0,000143	0,000156	0,000165	0,000151	0,00014953	1,21427E-05	8,121
Q9CWZ7	SNAG_MOUSE	Gamma-soluble NSF attachment protein	0,000406	0,000429	0,000426	0,000452	0,000428301	1,872E-05	4,371	0,000437	0,000407	0,000443	0,000401	0,000397	0,000416836	2,15228E-05	5,163
Q93092	TALDO_MOUSE	Transaldolase	0,000246	0,000255	0,000239	0,000269	0,000252221	1,28533E-05	5,096	0,000283	0,000257	0,000257	0,000249	0,000214	0,000251756	2,47628E-05	9,836
Q8BG39	SV2B_MOUSE	Synaptic vesicle glycoprotein 2B	0,000425	0,000366	0,000486	0,000434	0,000427638	4,93475E-05	11,540	0,000538	0,000424	0,000447	0,000437	0,000537	0,000476552	5,59442E-05	11,739
P51174	ACADL_MOUSE	Long-chain specific acyl-CoA dehydrogenase, mitochondrial	0,000208	0,000211	0,000181	0,000203	0,000200914	1,35225E-05	6,730	0,000205	0,000211	0,000232	0,000223	0,000218	0,000217584	1,02706E-05	4,720
Q60854	SPB6_MOUSE	Serpin B6	6,58E-05	7,37E-05	6,43E-05	6,98E-05	6,84187E-05	4,23851E-06	6,195	7,01E-05	7,05E-05	7,52E-05	6,84E-05	7,4E-05	7,16562E-05	2,84348E-06	3,968
Q9QYB5	ADDG_MOUSE	Gamma-adducin	0,00026	0,000292	0,000279	0,000256	0,000271635	1,67576E-05	6,169	0,00027	0,000276	0,000267	0,000252	0,000288	0,00027081	1,29639E-05	4,787
P62806	H4_MOUSE	Histone H4	0,00297	0,003937	0,003814	0,003671	0,003598057	0,000432864	12,030	0,003041	0,003306	0,003834	0,003405	0,003461	0,003409407	0,000286818	8,413
Q8BG05	ROA3_MOUSE	Heterogeneous nuclear ribonucleoprotein A3	0,000762	0,000795	0,000841	0,00086	0,000814877	4,43685E-05	5,445	0,000803	0,000669	0,0008	0,00074	0,000718	0,00074597	5,68061E-05	7,615
Q9WUM4	COR1C_MOUSE	Coronin-1C	0,000169	0,000187	0,000177	0,000168	0,000175082	8,78676E-06	5,019	0,000175	0,000167	0,000183	0,000186	0,000182	0,00017867	7,54942E-06	4,225
Q8CGK3	LONM_MOUSE	Lon protease homolog, mitochondrial	0,000271	0,000302	0,00029	0,000285	0,000287123	1,25562E-05	4,373	0,000286	0,000252	0,000281	0,000286	0,000296	0,000279948	1,67406E-05	5,980
P21279	GNAQ_MOUSE	Guanine nucleotide-binding protein G(q) subunit alpha	0,000211	0,000238	0,000233	0,000228	0,000227452	1,18038E-05	5,190	0,00022	0,000214	0,000214	0,000219	0,000218	0,000217064	2,70439E-06	1,246
P12023	A4_MOUSE	Amyloid beta A4 protein	2,72E-05	2,66E-05	2,79E-05	2,82E-05	2,746E-05	6,94001E-07	2,527	2,95E-05	2,46E-05	2,76E-05	2,31E-05	3,15E-05	2,72438E-05	3,45522E-06	12,683
Q61656	DDX5_MOUSE	Probable ATP-dependent RNA helicase DDX5	0,000117	0,000131	0,000141	0,000134	0,000130609	1,0078E-05	7,716	0,000138	0,000108	0,000138	0,000129	0,000137	0,000129773	1,29307E-05	9,964
Q9DCX2	ATP5H_MOUSE	ATP synthase subunit d, mitochondrial	0,000988	0,000924	0,000928	0,000965	0,000951084	3,05616E-05	3,213	0,000889	0,000946	0,000944	0,000871	0,000904	0,000910522	3,33865E-05	3,667
P62137	PP1A_MOUSE	Serine/threonine-protein phosphatase PP1-alpha catalytic subunit	0,000137	0,000137	0,000157	0,000147	0,000144781	9,47377E-06	6,544	0,000149	0,000126	0,000121	0,000125	0,000129	0,000129932	1,0871E-05	8,367
Q9D8N0	EF1G_MOUSE	Elongation factor 1-gamma	0,000407	0,000448	0,00044	0,000418	0,000428263	1,92393E-05	4,492	0,000417	0,000395	0,000439	0,000416	0,000406	0,000414554	1,60502E-05	3,872
Q6PER3	MARE3_MOUSE	Microtubule-associated protein RP/EB family member 3	0,000326	0,000319	0,0003	0,000342	0,0003216	1,72895E-05	5,376	0,00032	0,000328	0,000284	0,000274	0,000272	0,000295515	2,64058E-05	8,936
P51863	VA0D1_MOUSE	V-type proton ATPase subunit d 1	0,001655	0,00184	0,001865	0,001903	0,00181555	0,000110199	6,070	0,001841	0,001627	0,001651	0,001706	0,001601	0,001685158	9,54134E-05	5,662
P70414	NAC1_MOUSE	Sodium/calcium exchanger 1	0,000193	0,000219	0,00018	0,000217	0,000201822	1,887E-05	9,350	0,000223	0,000206	0,000202	0,000205	0,000186	0,000204474	1,34054E-05	6,556
Q9CZ44	NSF1C_MOUSE	NSFL1 cofactor p47	0,000221	0,000241	0,000242	0,000246	0,000237345	1,1136E-05	4,692	0,00025	0,000232	0,000221	0,000225	0,00023	0,00023162	1,11505E-05	4,814
P47754	CAZA2_MOUSE	F-actin-capping protein subunit alpha-2	0,000638	0,000656	0,000672	0,000714	0,000669929	3,28471E-05	4,903	0,000642	0,000601	0,000609	0,000547	0,000578	0,000595376	3,55732E-05	5,975
P70404	IDHG1_MOUSE	Isocitrate dehydrogenase [NAD] subunit gamma 1, mitochondrial	0,000617	0,000635	0,000629	0,000674	0,000638945	2,45991E-05	3,850	0,000648	0,000627	0,000669	0,000632	0,000621	0,000639401	1,93996E-05	3,034

Q00493	CBPE_MOUSE	Carboxypeptidase E	7,46E-05	8,62E-05	8,27E-05	8,7E-05	8,26196E-05	5,65065E-06	6,839	7,35E-05	6,75E-05	7,9E-05	7,7E-05	7,7E-05	7,48106E-05	4,54545E-06	6,076
Q62465	VAT1_MOUSE	Synaptic vesicle membrane protein VAT-1 homolog	0,000403	0,000452	0,000448	0,000452	0,000438767	2,41084E-05	5,495	0,00035	0,000445	0,000375	0,000425	0,000435	0,00040598	4,10472E-05	10,111
Q9D0I9	SYRC_MOUSE	Arginine--tRNA ligase, cytoplasmic	0,0001	0,00011	9,81E-05	9,86E-05	0,00010182	5,75255E-06	5,650	9,24E-05	9,13E-05	9,47E-05	8,43E-05	9,84E-05	9,22148E-05	5,21748E-06	5,658
O55125	NIPS1_MOUSE	Protein NipSnap homolog 1	0,000215	0,000218	0,000201	0,000237	0,000217424	1,49986E-05	6,898	0,000211	0,000137	0,000231	0,000224	0,000223	0,000205164	3,87076E-05	18,867
P54071	IDHP_MOUSE	Isocitrate dehydrogenase [NADP], mitochondrial	0,000321	0,000375	0,000332	0,00035	0,000344514	2,33914E-05	6,790	0,000303	0,000249	0,000344	0,000282	0,000327	0,000300891	3,71171E-05	12,336
Q9DCT2	NDUS3_MOUSE	NADH dehydrogenase [ubiquinone] iron-sulfur protein 3, mitochondrial	0,000613	0,000543	0,00054	0,000549	0,000561247	3,46477E-05	6,173	0,00053	0,000532	0,000547	0,000497	0,000465	0,000514184	3,29763E-05	6,413
Q9CWS0	DDAH1_MOUSE	N(G),N(G)-dimethylarginine dimethylaminohydrolase 1	0,000317	0,000293	0,000291	0,000309	0,000302484	1,25195E-05	4,139	0,000346	0,000331	0,000288	0,000309	0,000281	0,000310969	2,76662E-05	8,897
Q8BW75	AOFB_MOUSE	Amine oxidase [flavin-containing] B	0,000247	0,000251	0,000231	0,00019	0,000229582	2,79323E-05	12,167	0,000249	0,000246	0,000273	0,000256	0,000274	0,000259839	1,31317E-05	5,054
Q80UG5	SEPT9_MOUSE	Septin-9	6,83E-05	6,54E-05	6,77E-05	6,38E-05	6,63245E-05	2,07075E-06	3,122	7,76E-05	5,69E-05	6,9E-05	6,43E-05	6,76E-05	6,70764E-05	7,49982E-06	11,181
P27546	MAP4_MOUSE	Microtubule-associated protein 4	0,000132	0,000124	0,000108	0,000114	0,000119356	1,05702E-05	8,856	0,00012	0,000112	0,000119	9,61E-05	0,000108	0,000110953	9,76432E-06	8,800
P60487	PLPP_MOUSE	Pyridoxal phosphate phosphatase	0,000296	0,000283	0,000334	0,000324	0,000308982	2,38023E-05	7,703	0,000298	0,000279	0,000279	0,000289	0,000288	0,000286733	7,99387E-06	2,788
Q91V41	RAB14_MOUSE	Ras-related protein Rab-14	0,00052	0,00053	0,000556	0,000559	0,000541397	1,95216E-05	3,606	0,000533	0,000513	0,000594	0,000555	0,000527	0,000544481	3,16372E-05	5,811
P61161	ARP2_MOUSE	Actin-related protein 2	0,000492	0,000604	0,000622	0,000649	0,00059182	6,90366E-05	11,665	0,000627	0,000569	0,000666	0,000661	0,000691	0,000642949	4,71721E-05	7,337
Q9DB20	ATPO_MOUSE	ATP synthase subunit O, mitochondrial	0,001304	0,001198	0,001337	0,001208	0,001261778	6,91868E-05	5,483	0,001206	0,001245	0,001254	0,001328	0,00129	0,001264785	4,65604E-05	3,681
Q8C437	PEX5R_MOUSE	PEX5-related protein	0,000121	0,000125	0,000102	0,000119	0,00011688	1,0035E-05	8,586	0,000124	0,000121	0,000116	0,000111	0,000101	0,00011449	9,23072E-06	8,062
P14231	AT1B2_MOUSE	Sodium/potassium-transporting ATPase subunit beta-2	0,00166	0,001516	0,001451	0,001581	0,0015522	8,92339E-05	5,749	0,001384	0,001561	0,001609	0,001594	0,001355	0,001500588	0,000121378	8,089
Q9CQA3	SDHB_MOUSE	Succinate dehydrogenase [ubiquinone] iron-sulfur subunit, mitochondrial	0,00043	0,000396	0,000387	0,000418	0,000407757	1,97554E-05	4,845	0,000388	0,000425	0,000417	0,000396	0,000354	0,000395929	2,79585E-05	7,061
Q61753	SERA_MOUSE	D-3-phosphoglycerate dehydrogenase	0,000607	0,00065	0,000578	0,000578	0,000603374	3,42338E-05	5,674	0,000613	0,00061	0,000623	0,00063	0,000554	0,000605814	3,00154E-05	4,955
Q9D8E6	RL4_MOUSE	60S ribosomal protein L4	9,69E-05	0,000105	0,000102	0,000107	0,000102709	4,25206E-06	4,140	9,52E-05	9,05E-05	0,000101	9,12E-05	9,77E-05	9,51563E-05	4,48689E-06	4,715
Q9CR62	M2OM_MOUSE	Mitochondrial 2-oxoglutarate/malate carrier protein	0,001142	0,001077	0,001048	0,001084	0,001087732	3,96503E-05	3,645	0,000858	0,000818	0,000949	0,000876	0,000735	0,000846954	7,86035E-05	9,281
P68254	1433T_MOUSE	14-3-3 protein theta	0,000964	0,000596	0,000864	0,000738	0,000790238	0,000159076	20,130	0,000745	0,00084	0,000696	0,000689	0,000605	0,000715183	8,61526E-05	12,046
Q9QXS6	DREB_MOUSE	Drebrin	0,000462	0,000502	0,000597	0,000544	0,000526238	5,78235E-05	10,988	0,000491	0,000403	0,000468	0,000487	0,000523	0,000474492	4,45909E-05	9,398
Q91VR2	ATPG_MOUSE	ATP synthase subunit gamma, mitochondrial	0,001478	0,001344	0,001392	0,001504	0,001429542	7,41112E-05	5,184	0,001377	0,001443	0,001464	0,001332	0,001257	0,001374688	8,4033E-05	6,113
P17742	PPIA_MOUSE	Peptidyl-prolyl cis-trans isomerase A	0,003599	0,003696	0,004655	0,004631	0,00414531	0,000576116	13,898	0,00388	0,003851	0,004237	0,0042	0,004014	0,004036307	0,000177897	4,407
Q99PT1	GDIR1_MOUSE	Rho GDP-dissociation inhibitor 1	0,000822	0,000785	0,000792	0,000823	0,00080558	1,97449E-05	2,451	0,00085	0,000894	0,000917	0,000856	0,000791	0,000861331	4,83777E-05	5,617
Q9JHI5	IVD_MOUSE	Isovaleryl-CoA dehydrogenase, mitochondrial	0,000279	0,000303	0,000285	0,000297	0,000291131	1,07855E-05	3,705	0,000262	0,000296	0,000298	0,000278	0,000261	0,000278958	1,78721E-05	6,407
Q99PJ0	NTRI_MOUSE	Neurotrimin	0,000246	0,000248	0,000309	0,000264	0,000266601	2,92194E-05	10,960	0,000257	0,000236	0,00023	0,000237	0,000262	0,00024458	1,39285E-05	5,695
Q8R1Q8	DC1L1_MOUSE	Cytoplasmic dynein 1 light intermediate chain 1	0,000149	0,000155	0,000152	0,000104	0,000140211	2,41324E-05	17,211	0,000165	0,000145	0,000156	0,00015	0,00015	0,00015327	7,90335E-06	5,156
Q9R0Y5	KAD1_MOUSE	Adenylate kinase isoenzyme 1	0,000534	0,000541	0,000558	0,00054	0,000543102	1,01225E-05	1,864	0,000545	0,00054	0,000541	0,000512	0,000484	0,000524259	2,60393E-05	4,967
P17879	HS71B_MOUSE	Heat shock 70 kDa protein 1B	6,82E-05	0,000106	0,000101	9E-05	9,12488E-05	1,67448E-05	18,351	7,39E-05	6,82E-05	8,15E-05	7,44E-05	8,03E-05	7,56847E-05	5,37558E-06	7,103
Q9Z0X1	AIFM1_MOUSE	Apoptosis-inducing factor 1, mitochondrial	2,95E-05	2,69E-05	2,49E-05	2,5E-05	2,65843E-05	2,16692E-06	8,151	2,47E-05	2,16E-05	2,33E-05	2,03E-05	2,02E-05	2,20258E-05	1,94996E-06	8,853
O55234	PSB5_MOUSE	Proteasome subunit beta type-5	0,000267	0,000303	0,000308	0,000306	0,000296051	1,97952E-05	6,686	0,000297	0,0003	0,000323	0,000294	0,000266	0,000296082	2,01874E-05	6,818
P12658	CALB1_MOUSE	Calbindin	6,23E-05	6,47E-05	6,51E-05	7,12E-05	6,58224E-05	3,81691E-06	5,799	5,37E-05	5,86E-05	6,13E-05	6,56E-05	5,65E-05	5,91397E-05	4,5625E-06	7,715
Q9QZQ8	H2AY_MOUSE	Core histone macro-H2A.1	0,000247	0,000282	0,000258	0,000296	0,000270958	2,21376E-05	8,170	0,000264	0,000234	0,000283	0,000249	0,000243	0,000254776	1,92228E-05	7,545
Q8VE33	GD1L1_MOUSE	Ganglioside-induced differentiation-associated protein 1-like 1	7,91E-05	9,82E-05	0,000101	9,5E-05	9,33396E-05	9,82386E-06	10,525	9,72E-05	8,59E-05	8,93E-05	9,04E-05	9,65E-05	9,18461E-05	4,88071E-06	5,314
P14148	RL7_MOUSE	60S ribosomal protein L7	0,000232	0,000273	0,000296	0,000285	0,000271403	2,76803E-05	10,199	0,000238	0,000253	0,00028	0,00024	0,000242	0,000250591	1,72179E-05	6,871
Q61885	MOG_MOUSE	Myelin-oligodendrocyte glycoprotein	0,000865	0,00079	0,00075	0,000842	0,000811682	5,21513E-05	6,425	0,000794	0,001106	0,001091	0,000974	0,000806	0,000954338	0,000149951	15,713
P19157	GSTP1_MOUSE	Glutathione S-transferase P 1	0,001605	0,001584	0,001651	0,001719	0,00163997	5,98189E-05	3,648	0,001654	0,001745	0,001914	0,001822	0,001526	0,001731976	0,000149968	8,659
P00920	CAH2_MOUSE	Carbonic anhydrase 2	0,002219	0,001847	0,001784	0,002089	0,001984733	0,000204024	10,280	0,001891	0,001522	0,001974	0,001807	0,001435	0,001725687	0,000235322	13,636
Q8BH44	COR2B_MOUSE	Coronin-2B	0,000178	0,000159	0,00013	0,000144	0,000152744	2,03697E-05	13,336	0,000148	0,000118	0,000138	0,000156	0,000143	0,000140438	1,43888E-05	10,246
Q91ZU6	DYST_MOUSE	Dystonin	3,3E-05	4,43E-05	4,85E-05	4,57E-05	4,28873E-05	6,81859E-06	15,899	4,5E-05	3,5E-05	4,14E-05	3,96E-05	3,83E-05	3,98647E-05	3,67665E-06	9,223
Q8BVI4	DHPR_MOUSE	Dihydropteridine reductase	0,001055	0,000978	0,000835	0,000951	0,000954669	9,13292E-05	9,567	0,001053	0,000942	0,001063	0,000949	0,000717	0,000944635	0,000139293	14,746
Q9D0M3	CY1_MOUSE	Cytochrome c1, heme protein, mitochondrial	0,001368	0,00136	0,001308	0,0013	0,001333973	3,48689E-05	2,614	0,001304	0,001307	0,001343	0,001259	0,001276	0,001297689	3,2332E-05	2,492
P57746	VATD_MOUSE	V-type proton ATPase subunit D	0,000586	0,000622	0,000629	0,000657	0,000623185	2,92585E-05	4,695	0,000669	0,000584	0,000647	0,000623	0,000528	0,000610168	5,56521E-05	9,121
O88533	DDC_MOUSE	Aromatic-L-amino-acid decarboxylase	4,77E-05	4,43E-05	4,33E-05	4,39E-05	4,48072E-05	2,00516E-06	4,475	3,76E-05	2,95E-05	4,3E-05	5,59E-05	5,29E-05	4,37826E-05	1,08746E-05	24,838
P24369	PPIB_MOUSE	Peptidyl-prolyl cis-trans isomerase B	0,000164	0,000187	0,000187	0,000152	0,000172291	1,74123E-05	10,106	0,000174	0,000181	0,000187	0,000173	0,000169	0,000176854	7,18425E-06	4,062
P08226	APOE_MOUSE	Apolipoprotein E	0,000215	0,000197	0,000186	0,000183	0,000195011	1,45318E-05	7,452	0,00019	0,000181	0,000194	0,000187	0,000181	0,000186585	5,79452E-06	3,106
P61982	1433G_MOUSE	14-3-3 protein gamma	0,001764	0,001863	0,001784	0,001786	0,001799218	4,38767E-05	2,439	0,001648	0,001924	0,001826	0,00169	0,001624	0,001742494	0,000128231	7,359
O54983	CRYM_MOUSE	Ketimine reductase mu-crystallin	0,000434	0,000438	0,000527	0,000455	0,000463468	4,36226E-05	9,412	0,00034	0,000378	0,000502	0,000524	0,000698	0,000488467	0,000141039	28,874
P29758	OAT_MOUSE	Ornithine aminotransferase, mitochondrial	0,000245	0,00025	0,000266	0,000262	0,0002554	9,95831E-06	3,899	0,000244	0,00023	0,000252	0,000259	0,000235	0,000243889	1,20287E-05	4,932
P49312	ROA1_MOUSE	Heterogeneous nuclear ribonucleoprotein A1	0,000319	0,000359	0,000372	0,000401	0,000362833	3,38579E-05	9,332	0,000402	0,000326	0,000396	0,000376	0,000333	0,000366521	3,54432E-05	9,670
Q8R3V5	SHLB2_MOUSE	Endophilin-B2	0,000259	0,000286	0,000296	0,000264	0,000276241	1,78113E-05	6,448	0,000267	0,000241	0,000274	0,000244	0,000282	0,000261517	1,83951E-05	7,034
Q9R0P5	DEST_MOUSE	Destrin	7,93E-05	9,3E-05	8,42E-05	9E-05	8,66212E-05	6,07356E-06	7,012	8,17E-05	8,94E-05	0,000108	9,84E-05	8,5E-05	9,24098E-05	1,05338E-05	11,399
P15532	NDKA_MOUSE	Nucleoside diphosphate kinase A	0,000427	0,000496	0,000523	0,000466	0,000478266	4,10937E-05	8,592	0,000416	0,000403	0,000439	0,000419	0,000358	0,000407094	3,01197E-05	7,399
O70443	GNAZ_MOUSE	Guanine nucleotide-binding protein G(z) subunit alpha	0,00028	0,0003	0,000322	0,000298	0,000300088	1,74821E-05	5,826	0,000326	0,000299	0,000314	0,000308	0,000323	0,000314084	1,0964E-05	3,491
P97300	NPTN_MOUSE	Neuroplastin	0,00074	0,000688	0,000864	0,000782	0,000768672	7,44884E-05	9,691	0,000815	0,000677	0,00071	0,000729	0,000865	0,00075895	7,79418E-05	10,270
P23242	CXA1_MOUSE	Gap junction alpha-1 protein	0,000158	0,000141	0,000138	0,000167	0,000150827	1,36605E-05	9,057	0,000142	0,000122	0,000165	0,000129	0,000108	0,000133373	2,15655E-05	16,169
Q8VIJ6	SFPQ_MOUSE	Splicing factor, proline- and glutamine-rich	0,000107	0,000128	0,000138	0,000117	0,00012256	1,36976E-05	11,176	0,000137	0,000123	0,000135	0,000134	0,000143	0,000134534	7,32682E-06	5,446
Q9ESJ4	SPN90_MOUSE	NCK-interacting protein with SH3 domain	9,75E-05	0,000116	0,000104	0,000118	0,000108835	9,75102E-06	8,959	0,000125	9,22E-05	0,000112	0,000128	0,000128	0,000116931	1,52595E-05	13,050
Q9DCW4	ETFB_MOUSE	Electron transfer flavoprotein subunit beta	0,000366	0,000331	0,000327	0,000337	0,000340345	1,78361E-05	5,241	0,000324	0,000403	0,000353	0,00032	0,000322	0,000344485	3,53396E-05	10,259
Q9JII6	AK1A1_MOUSE	Alcohol dehydrogenase [NADP(+)]	0,000449	0,00045	0,000548	0,000513	0,000489988	4,89447E-05	9,989	0,000446	0,000369	0,000438	0,000414	0,000352	0,000403886	4,17257E-05	10,331
Q8C0M9	ASGL1_MOUSE	Isoaspartyl peptidase/L-asparaginase	0,000445	0,000462	0,000476	0,000469	0,00046313	1,36434E-05	2,946	0,000425	0,000492	0,000476	0,000417	0,000381	0,000438333	4,52207E-05	10,317
Q7TQD2	TPPP_MOUSE	Tubulin polymerization-promoting protein	0,00092	0,000869	0,000708	0,000836	0,000833282	9,02899E-05	10,835	0,000961	0,001013	0,000933	0,00098	0,000849	0,000947381	6,23306E-05	6,579
P09528	FRIH_MOUSE	Ferritin heavy chain	0,000133	0,000142	0,000124	0,000126	0,000131412	8,07347E-06	6,144	0,000111	0,000118	0,00014	0,000111	0,000104	0,000116881	1,40163E-05	11,992
Q9Z2U0	PSA7_MOUSE	Proteasome subunit alpha type-7	0,000197	0,000193	0,000199	0,00022	0,000202357	1,18674E-05	5,865	0,000218	0,00022	0,000202	0,000195	0,000197	0,000206251	1,18379E-05	5,740
Q9WV55	VAPA_MOUSE	Vesicle-associated membrane protein-associated protein A	0,000252	0,000271	0,00029	0,000273	0,000271358	1,57367E-05	5,799	0,000264	0,000286	0,00029	0,000267	0,000259	0,000273192	1,38739E-05	5,078
P58389	PTPA_MOUSE	Serine/threonine-protein phosphatase 2A activator	0,000467	0,000503	0,000474	0,000507	0,000487706	1,99679E-05	4,094	0,000486	0,000446	0,000449	0,000431	0,000396	0,000441677	3,26195E-05	7,385
P25444	RS2_MOUSE	40S ribosomal protein S2	0,000167	0,000167	0,00017	0,00018	0,000171118	5,94296E-06	3,473	0,000158	0,000169	0,000168	0,00014	0,000128	0,000152435	1,77797E-05	11,664
Q8R016	BLMH_MOUSE	Bleomycin hydrolase	0,000143	0,000142	0,00014	0,000138	0,000141087	2,19422E-06	1,555	0,000149	0,000134	0,00015	0,00015	0,000146	0,000145865	6,6808E-06	4,580
Q8C0E2	VP26B_MOUSE	Vacuolar protein sorting-associated protein 26B	0,00022	0,000227	0,00021	0,000223	0,000219758	7,41026E-06	3,372	0,000176	0,000162	0,000236	0,000205	0,000235	0,000203064	3,36002E-05	16,547
Q99N28	CADM3_MOUSE	Cell adhesion molecule 3	0,000472	0,000502	0,000526	0,000487	0,000496711	2,29598E-05	4,622	0,00055	0,000544	0,000536	0,000487	0,000554	0,000534168	2,73537E-05	5,121

P19783	COX41_MOUSE	Cytochrome c oxidase subunit 4 isoform 1, mitochondrial	0,001192	0,001235	0,001302	0,001173	0,001225551	5,70954E-05	4,659	0,001157	0,001165	0,001366	0,0013	0,001187	0,001235034	9,34163E-05	7,564
P28740	KIF2A_MOUSE	Kinesin-like protein KIF2A	0,000103	0,000121	0,000107	0,000115	0,000111343	7,91336E-06	7,107	0,000115	0,000107	0,000116	0,000111	0,000111	0,000111964	3,54251E-06	3,164
O55126	NIPS2_MOUSE	Protein NipSnap homolog 2	0,000379	0,000378	0,000366	0,000378	0,000374884	6,22929E-06	1,662	0,000367	0,00032	0,000375	0,000383	0,000389	0,00036681	2,73836E-05	7,465
Q9D8Y0	EFHD2_MOUSE	EF-hand domain-containing protein D2	0,001	0,000805	0,000765	0,000828	0,000849632	0,000103878	12,226	0,000975	0,000866	0,000833	0,000847	0,000728	0,000849645	8,80373E-05	10,362
Q91V61	SFXN3_MOUSE	Sideroflexin-3	0,000597	0,000523	0,000629	0,000517	0,000566495	5,5321E-05	9,765	0,000555	0,000508	0,000499	0,000513	0,000573	0,000529696	3,2092E-05	6,059
P09671	SODM_MOUSE	Superoxide dismutase [Mn], mitochondrial	0,000647	0,000619	0,000576	0,000588	0,000607923	3,19444E-05	5,255	0,000633	0,000672	0,000611	0,000553	0,000553	0,000604283	5,19425E-05	8,596
P62821	RAB1A_MOUSE	Ras-related protein Rab-1A	0,000548	0,000426	0,000156	0,000513	0,000410576	0,000177515	43,236	0,000504	0,000629	0,000451	0,000486	0,000449	0,000503929	7,36612E-05	14,617
Q99LC5	ETFA_MOUSE	Electron transfer flavoprotein subunit alpha, mitochondrial	0,000425	0,00042	0,000365	0,000423	0,000408284	2,90663E-05	7,119	0,000393	0,00046	0,000402	0,000378	0,000389	0,000404441	3,24981E-05	8,035
Q91VM5	RMXL1_MOUSE	RNA binding motif protein, X-linked-like-1	0,000214	0,000227	0,000259	0,000248	0,000236713	2,03946E-05	8,616	0,000228	0,00021	0,000234	0,000216	0,00024	0,000225651	1,26565E-05	5,609
P63158	HMGB1_MOUSE	High mobility group protein B1	3,83E-05	3,8E-05	3,59E-05	4,68E-05	3,97425E-05	4,8033E-06	12,086	3,98E-05	3,86E-05	5,12E-05	3,78E-05	3,74E-05	4,09623E-05	5,7924E-06	14,141
Q61792	LASP1_MOUSE	LIM and SH3 domain protein 1	4,08E-05	3,73E-05	3,13E-05	4E-05	3,73521E-05	4,27536E-06	11,446	4E-05	3,9E-05	3,77E-05	3,3E-05	2,49E-05	3,49229E-05	6,22489E-06	17,825
O70161	PI51C_MOUSE	Phosphatidylinositol 4-phosphate 5-kinase type-1 gamma	9,16E-05	9,3E-05	8,08E-05	9,42E-05	8,98969E-05	6,17926E-06	6,874	0,000103	8,74E-05	8,82E-05	9,46E-05	0,000109	9,64059E-05	9,36672E-06	9,716
Q8BLQ9	CADM2_MOUSE	Cell adhesion molecule 2	0,000416	0,000403	0,000398	0,000373	0,000397484	1,79947E-05	4,527	0,000457	0,000429	0,000416	0,000445	0,000431	0,00043549	1,56607E-05	3,596
Q6ZWN5	RS9_MOUSE	40S ribosomal protein S9	9,82E-05	0,000108	0,000114	0,000115	0,000108861	7,9044E-06	7,261	9,53E-05	9,85E-05	0,000108	0,000103	8,94E-05	9,86972E-05	6,95986E-06	7,052
Q80Z24	NEGR1_MOUSE	Neuronal growth regulator 1	0,000232	0,000252	0,000315	0,00025	0,000262348	3,6451E-05	13,894	0,000271	0,000242	0,000247	0,000265	0,000284	0,000261652	1,73951E-05	6,648
P35279	RAB6A_MOUSE	Ras-related protein Rab-6A	6,47E-05	6,51E-05	7,43E-05	7,5E-05	6,97737E-05	5,65791E-06	8,109	8,6E-05	8,2E-05	1E-04	0,000109	8,49E-05	9,22917E-05	1,14767E-05	12,435
P27601	GNA13_MOUSE	Guanine nucleotide-binding protein subunit alpha-13	7,79E-05	7,42E-05	7,91E-05	7,58E-05	7,673E-05	2,16205E-06	2,818	7,44E-05	7,26E-05	7,58E-05	8,02E-05	7,16E-05	7,49353E-05	3,36769E-06	4,494
P46638	RB11B_MOUSE	Ras-related protein Rab-11B	0,00104	0,001068	0,001072	0,00113	0,001077493	3,78118E-05	3,509	0,000971	0,001036	0,001114	0,000986	0,000869	0,000995386	9,01143E-05	9,053
Q9Z2U1	PSA5_MOUSE	Proteasome subunit alpha type-5	0,000206	0,00022	0,00022	0,000222	0,000217064	7,44874E-06	3,432	0,000207	0,000199	0,000234	0,000217	0,000205	0,00021253	1,36733E-05	6,434
Q91Z53	GRHPR_MOUSE	Glyoxylate reductase/hydroxypyruvate reductase	0,000229	0,000245	0,000171	0,000259	0,000225829	3,8407E-05	17,007	0,000254	0,000222	0,0002	0,000243	0,00015	0,000213713	4,11424E-05	19,251
P63001	RAC1_MOUSE	Ras-related C3 botulinum toxin substrate 1	7,3E-05	7,03E-05	0,000101	9E-05	8,34489E-05	1,43393E-05	17,183	7,15E-05	0,000103	8,89E-05	8,53E-05	0,000105	9,08331E-05	1,39039E-05	15,307
Q9D172	ES1_MOUSE	ES1 protein homolog, mitochondrial	0,000197	0,000202	0,000186	0,000204	0,000197499	8,12076E-06	4,112	0,000205	0,000231	0,000199	0,000191	0,000174	0,000199988	2,08271E-05	10,414
P68510	1433F_MOUSE	14-3-3 protein eta	0,000722	0,000735	0,000746	0,000763	0,000741551	1,71806E-05	2,317	0,000759	0,000729	0,000748	0,000658	0,000588	0,000696471	7,22883E-05	10,379
Q9D6M3	GHC1_MOUSE	Mitochondrial glutamate carrier 1	0,00037	0,00032	0,000405	0,000392	0,000371705	3,75212E-05	10,094	0,000348	0,00034	0,000372	0,000317	0,000329	0,000341112	2,07301E-05	6,077
Q9DB05	SNAA_MOUSE	Alpha-soluble NSF attachment protein	0,000417	0,000402	0,000386	0,000408	0,000403363	1,31317E-05	3,256	0,000386	0,000385	0,000387	0,000344	0,000307	0,000361756	3,58476E-05	9,909
Q6PDM2	SRSF1_MOUSE	Serine/arginine-rich splicing factor 1	0,000141	0,000156	0,000155	0,000157	0,000152577	7,55495E-06	4,952	0,000151	0,000144	0,000164	0,000146	0,000135	0,000148067	1,07059E-05	7,230
P00493	HPRT_MOUSE	Hypoxanthine-guanine phosphoribosyltransferase	0,00027	0,000312	0,000311	0,000331	0,000306197	2,55978E-05	8,360	0,000289	0,000301	0,000352	0,000389	0,000325	0,000331422	4,023E-05	12,139
O55023	IMPA1_MOUSE	Inositol monophosphatase 1	0,000488	0,000466	0,000572	0,000554	0,00052018	5,10208E-05	9,808	0,000449	0,000437	0,000457	0,000422	0,000376	0,000428399	3,18372E-05	7,432
Q9Z204	HNRPC_MOUSE	Heterogeneous nuclear ribonucleoproteins C1/C2	0,000356	0,000372	0,000388	0,000392	0,000377287	1,66382E-05	4,410	0,000381	0,000336	0,000375	0,000406	0,000408	0,000381069	2,92091E-05	7,665
P10126	EF1A1_MOUSE	Elongation factor 1-alpha 1	0,000955	0,001104	0,000978	0,000996	0,001007994	6,59415E-05	6,542	0,001096	0,00119	0,001181	0,001281	0,001204	0,001190383	6,58978E-05	5,536
P97315	CSRP1_MOUSE	Cysteine and glycine-rich protein 1	0,000323	0,000331	0,000272	0,000304	0,000307534	2,64476E-05	8,600	0,000339	0,000306	0,000312	0,000284	0,000265	0,000301174	2,83704E-05	9,420
Q8CAY6	THIC_MOUSE	Acetyl-CoA acetyltransferase, cytosolic	7,65E-05	0,000111	0,000127	0,000121	0,000108818	2,25493E-05	20,722	0,000106	8,49E-05	0,000113	6,04E-05	8,3E-05	8,94446E-05	2,07893E-05	23,243
Q8K183	PDXK_MOUSE	Pyridoxal kinase	0,000407	0,00048	0,000455	0,000477	0,000454675	3,3421E-05	7,351	0,000393	0,000442	0,000475	0,000438	0,000456	0,000440937	3,04034E-05	6,895
Q8CDN6	TXNL1_MOUSE	Thioredoxin-like protein 1	0,000102	0,000118	0,000109	0,000116	0,000111307	7,25532E-06	6,518	0,000114	0,000111	0,000123	0,000109	0,00011	0,000113335	5,79145E-06	5,110
Q9CQJ8	NDUB9_MOUSE	NADH dehydrogenase [ubiquinone] 1 beta subcomplex subunit 9	0,000222	0,000191	0,000207	0,000202	0,000205375	1,25573E-05	6,114	0,000174	0,000146	0,000172	0,000135	0,000134	0,000151999	1,934E-05	12,724
Q7TQI3	OTUB1_MOUSE	Ubiquitin thioesterase OTUB1	0,001024	0,00113	0,001107	0,001114	0,001093753	4,76339E-05	4,355	0,001081	0,000992	0,001025	0,000951	0,000857	0,000981256	8,39975E-05	8,560
Q8R164	BPHL_MOUSE	Valacyclovir hydrolase	0,000212	0,000215	0,000229	0,000226	0,000220554	8,36065E-06	3,791	0,000225	0,000239	0,000238	0,000232	0,00023	0,000232879	5,60235E-06	2,406
Q3ULJ0	GPD1L_MOUSE	Glycerol-3-phosphate dehydrogenase 1-like protein	0,000113	0,000122	0,000129	0,000123	0,000121707	6,7386E-06	5,537	0,00013	0,000112	0,000136	0,000125	0,000123	0,000125421	9,0523E-06	7,218
Q8CHH9	SEPT8_MOUSE	Septin-8	0,000399	0,000399	0,000348	0,00038	0,000381629	2,44755E-05	6,413	0,000425	0,000372	0,000396	0,000418	0,000384	0,000399013	2,20992E-05	5,538
Q64105	SPRE_MOUSE	Sepiapterin reductase	0,000264	0,000293	0,000278	0,000283	0,000279454	1,19E-05	4,258	0,000288	0,000305	0,000289	0,000281	0,000267	0,000286072	1,36309E-05	4,765
P63094	GNAS2_MOUSE	Guanine nucleotide-binding protein G(s) subunit alpha isoforms short	0,000255	0,000264	0,000221	0,000277	0,000254278	2,4046E-05	9,457	0,000234	0,000202	0,000262	0,000262	0,000157	0,000223397	4,45118E-05	19,925
Q60931	VDAC3_MOUSE	Voltage-dependent anion-selective channel protein 3	0,001319	0,001181	0,001269	0,001224	0,001248291	5,91038E-05	4,735	0,001101	0,001264	0,001161	0,000981	0,000937	0,001088709	0,000132845	12,202
P62827	RAN_MOUSE	GTP-binding nuclear protein Ran	0,000225	0,000239	0,000254	0,000251	0,000242361	1,31427E-05	5,423	0,00022	0,0002	0,000222	0,000218	0,000233	0,000218381	1,1908E-05	5,453
Q9DCL9	PUR6_MOUSE	Multifunctional protein ADE2	0,000156	0,000152	0,000134	0,000138	0,000144691	1,07011E-05	7,396	0,000145	0,000133	0,000142	0,000149	0,000127	0,000139101	8,84797E-06	6,361
Q9WUM5	SUCA_MOUSE	Succinate--CoA ligase [ADP/GDP-forming] subunit alpha, mitochondrial	0,000346	0,000333	0,000331	0,000352	0,00034058	1,01676E-05	2,985	0,000352	0,00036	0,000357	0,000336	0,000349	0,000350896	9,59116E-06	2,733
P45591	COF2_MOUSE	Cofilin-2	0,000275	0,000272	0,000282	0,000302	0,000282671	1,34545E-05	4,760	0,000267	0,000214	0,000256	0,000298	0,000232	0,000253339	3,25248E-05	12,838
P52196	THTR_MOUSE	Thiosulfate sulfurtransferase	0,000306	0,000303	0,000291	0,000307	0,000301619	7,64089E-06	2,533	0,000306	0,000297	0,000301	0,000274	0,000225	0,000280309	3,34561E-05	11,935
P70441	NHRF1_MOUSE	Na(+)/H(+) exchange regulatory cofactor NHE-RF1	8,74E-05	8,94E-05	9,56E-05	8,93E-05	9,04082E-05	3,5626E-06	3,941	9,08E-05	7,72E-05	8,63E-05	7,94E-05	8,78E-05	8,43071E-05	5,73991E-06	6,808
Q9CRB9	MIC19_MOUSE	MICOS complex subunit Mic19	5,31E-05	5,4E-05	6,27E-05	5,53E-05	5,62743E-05	4,39497E-06	7,810	5,24E-05	4,89E-05	5,47E-05	4,37E-05	4,24E-05	4,84174E-05	5,31127E-06	10,970
Q9CQM9	GLRX3_MOUSE	Glutaredoxin-3	0,000117	0,00012	0,000107	0,000112	0,000114068	5,82226E-06	5,104	0,000119	0,000115	0,000121	0,000112	0,000109	0,00011523	5,30538E-06	4,604
Q62348	TSN_MOUSE	Translin	0,000134	0,000146	0,000166	0,000182	0,00015679	2,10126E-05	13,402	0,000133	0,000125	0,000145	0,000138	0,000119	0,000132155	1,04769E-05	7,928
P14206	RSSA_MOUSE	40S ribosomal protein SA	0,00036	0,000409	0,000412	0,000393	0,000393604	2,35155E-05	5,974	0,000385	0,000353	0,000394	0,000374	0,000383	0,000377775	1,59046E-05	4,210
Q63844	MK03_MOUSE	Mitogen-activated protein kinase 3	0,000155	0,000175	7,87E-05	0,000167	0,000143997	4,43267E-05	30,783	0,00017	0,000144	0,000159	0,000166	0,000158	0,000159449	9,85175E-06	6,179
P62823	RAB3C_MOUSE	Ras-related protein Rab-3C	0,000289	7,96E-05	0,000167	0,000109	0,000160945	9,25373E-05	57,496	0,000101	9,92E-05	0,000111	0,000163	0,000121	0,000118966	2,58402E-05	21,721
Q9WTX5	SKP1_MOUSE	S-phase kinase-associated protein 1	0,000145	0,000152	0,000173	0,00017	0,000160084	1,35335E-05	8,454	0,000139	0,000138	0,000149	0,000127	0,000115	0,000133535	1,27416E-05	9,542
P99029	PRDX5_MOUSE	Peroxiredoxin-5, mitochondrial	0,001396	0,001488	0,001458	0,001462	0,001451065	3,92736E-05	2,707	0,001376	0,001526	0,001474	0,001519	0,00144	0,001466655	6,17038E-05	4,207
Q922R8	PDIA6_MOUSE	Protein disulfide-isomerase A6	0,000185	0,000207	0,000193	0,000188	0,000193059	9,58762E-06	4,966	0,000176	0,000178	0,000185	0,000187	0,000184	0,000182195	4,83149E-06	2,652
P70296	PEBP1_MOUSE	Phosphatidylethanolamine-binding protein 1	0,001044	0,001265	0,002167	0,00147	0,00148639	0,000485652	32,673	0,001121	0,000853	0,001166	0,000957	0,001021	0,00102374	0,000125888	12,297
Q8BGN3	ENPP6_MOUSE	Ectonucleotide pyrophosphatase/phosphodiesterase family member 6	0,00012	0,00017	0,000114	0,000123	0,000131732	2,58641E-05	19,634	0,00015	7,04E-05	0,000147	0,000153	0,000145	0,000133203	3,52386E-05	26,455
Q9DCS9	NDUBA_MOUSE	NADH dehydrogenase [ubiquinone] 1 beta subcomplex subunit 10	0,000534	0,0005	0,000497	0,000527	0,000514355	1,88482E-05	3,664	0,000482	0,000502	0,000534	0,000489	0,000473	0,00049595	2,38056E-05	4,800
Q9R1T4	SEPT6_MOUSE	Septin-6	0,00031	0,000252	0,000265	0,000281	0,000276873	2,49457E-05	9,010	0,000316	0,000293	0,000278	0,000276	0,000269	0,000286288	1,88024E-05	6,568
Q61425	HCDH_MOUSE	Hydroxyacyl-coenzyme A dehydrogenase, mitochondrial	0,000176	0,000146	0,000132	0,000157	0,000152446	1,85068E-05	12,140	0,000164	0,000198	0,000153	0,00017	0,00015	0,000166959	1,89062E-05	11,324
O55091	IMPCT_MOUSE	Protein IMPACT	0,000117	0,000123	0,000131	0,000132	0,000125903	7,16944E-06	5,694	0,000111	0,000117	0,000142	0,000126	0,000127	0,000124739	1,19685E-05	9,595
Q9QUI0	RHOA_MOUSE	Transforming protein RhoA	0,00019	0,000213	0,00024	0,00024	0,000220767	2,41158E-05	10,924	0,000216	0,000257	0,000302	0,000272	0,000261	0,000261742	3,09661E-05	11,831
Q9D2G2	ODO2_MOUSE	Dihydrolipoyllysine-residue succinyltransferase component of 2-oxoglutarate dehydro	0,001041	0,000945	0,000935	0,000957	0,000969454	4,83137E-05	4,984	0,000904	0,000917	0,000936	0,00095	0,000855	0,000912577	3,64619E-05	3,995
Q9CPU0	LGUL_MOUSE	Lactoylglutathione lyase	0,00047	0,000415	0,000471	0,000551	0,000476728	5,61216E-05	11,772	0,000378	0,000372	0,000451	0,000397	0,000333	0,000386174	4,32258E-05	11,193
Q9ESM3	HPLN2_MOUSE	Hyaluronan and proteoglycan link protein 2	0,000172	0,000161	0,000109	0,000109	0,000137585	3,36356E-05	24,447	0,000119	0,000118	0,000107	9,43E-05	8,99E-05	0,000105716	1,34664E-05	12,738
Q9JJK2	LANC2_MOUSE	LanC-like protein 2	0,000251	0,000247	0,000269	0,000247	0,000253486	1,06216E-05	4,190	0,000256	0,000223	0,000239	0,000219	0,000235	0,000234298	1,48602E-05	6,342
P14869	RLA0_MOUSE	60S acidic ribosomal protein P0	0,00022	0,000259	0,000236	0,000252	0,000241895	1,76329E-05	7,289	0,000254	0,000244	0,000217	0,000223	0,000205	0,000228568	1,98782E-05	8,697
P54227	STMN1_MOUSE	Stathmin	0,000185	0,00017	0,000193	0,000199	0,000186968	1,24628E-05	6,666	0,000187	0,000181	0,000188	0,00019	0,000169	0,00018297	8,44269E-06	4,614
Q9D6J6	NDUV2_MOUSE	NADH dehydrogenase [ubiquinone] flavoprotein 2, mitochondrial	0,000727	0,000704	0,000603	0,000656	0,000672396	5,49404E-05	8,171	0,000589	0,000645	0,000655	0,000633	0,000612	0,000626939	2,65086E-05	4,228

Q3U0V1	FUBP2_MOUSE	Far upstream element-binding protein 2	0,00014	0,000211	0,000204	0,000187	0,000185441	3,18344E-05	17,167	0,000184	0,000163	0,000171	0,000176	0,000203	0,000179453	1,54215E-05	8,594
Q9DBP5	KCY_MOUSE	UMP-CMP kinase	0,000373	0,00037	0,000389	0,0004	0,000382959	1,44726E-05	3,779	0,000389	0,000383	0,000407	0,000408	0,000385	0,0003943	1,22803E-05	3,114
Q61171	PRDX2_MOUSE	Peroxiredoxin-2	0,001491	0,001542	0,001785	0,001551	0,001592473	0,000131369	8,249	0,001321	0,001286	0,001309	0,001276	0,001277	0,001293841	2,01572E-05	1,558
P62962	PROF1_MOUSE	Profilin-1	0,001042	0,001354	0,001355	0,001284	0,001258636	0,000148483	11,797	0,00114	0,001141	0,001233	0,001163	0,001127	0,001160909	4,24301E-05	3,655
P59999	ARPC4_MOUSE	Actin-related protein 2/3 complex subunit 4	0,000165	0,000192	0,000205	0,0002	0,000190307	1,76258E-05	9,262	0,000156	0,000136	0,00017	0,000159	0,000159	0,000155939	1,23969E-05	7,950
Q9QYJ0	DNJA2_MOUSE	DnaJ homolog subfamily A member 2	8,52E-05	8,84E-05	8,55E-05	8,51E-05	8,60381E-05	1,5514E-06	1,803	7,82E-05	7,98E-05	8,4E-05	7,99E-05	8,84E-05	8,20778E-05	4,14043E-06	5,045
Q8BVQ5	PPME1_MOUSE	Protein phosphatase methylesterase 1	0,000119	0,000116	0,00012	0,000122	0,000119277	2,1906E-06	1,837	0,000125	9,87E-05	0,000113	0,000123	0,000118	0,000115346	1,03917E-05	9,009
P62983	RS27A_MOUSE	Ubiquitin-40S ribosomal protein S27a	0,001663	0,001905	0,001786	0,001751	0,00177637	0,000100236	5,643	0,001741	0,001601	0,001581	0,001574	0,001595	0,001618299	6,93762E-05	4,287
Q9WTT4	VATG2_MOUSE	V-type proton ATPase subunit G 2	4,06E-05	4,82E-05	5,17E-05	5,65E-05	4,92248E-05	6,71287E-06	13,637	5,95E-05	5,52E-05	6,66E-05	5,75E-05	5,23E-05	5,82365E-05	5,39375E-06	9,262
O70435	PSA3_MOUSE	Proteasome subunit alpha type-3	0,000213	0,000213	0,000211	0,000231	0,000216995	9,15358E-06	4,218	0,000216	0,000227	0,000235	0,000216	0,000196	0,000217831	1,44853E-05	6,650
P62897	CYC_MOUSE	Cytochrome c, somatic	0,000828	0,000985	0,000978	0,000982	0,000943244	7,71983E-05	8,184	0,00092	0,001006	0,001049	0,000974	0,00094	0,000977748	5,15501E-05	5,272
P14131	RS16_MOUSE	40S ribosomal protein S16	0,000204	0,000239	0,000235	0,000234	0,000228212	1,60399E-05	7,029	0,000194	0,000176	0,000236	0,000201	0,000189	0,000199276	2,24041E-05	11,243
O55022	PGRC1_MOUSE	Membrane-associated progesterone receptor component 1	0,000111	0,000131	0,000149	0,000147	0,000134404	1,73616E-05	12,917	0,000104	0,000124	0,00014	0,000126	0,000122	0,000123099	1,30532E-05	10,604
Q60668	HNRPD_MOUSE	Heterogeneous nuclear ribonucleoprotein D0	0,000131	0,000136	0,000138	0,000172	0,000143961	1,87525E-05	13,026	0,000143	0,00012	0,000165	0,000174	0,00017	0,00015458	2,2579E-05	14,607
Q64433	CH10_MOUSE	10 kDa heat shock protein, mitochondrial	0,00018	0,000251	0,000257	0,000225	0,000228081	3,51205E-05	15,398	0,000174	0,000218	0,00022	0,000228	0,000205	0,000209184	2,12721E-05	10,169
P47911	RL6_MOUSE	60S ribosomal protein L6	0,00028	0,000322	0,000311	0,000326	0,000309977	2,08163E-05	6,715	0,000316	0,000288	0,000297	0,000285	0,000309	0,00029894	1,33353E-05	4,461
Q5PR73	DIRA2_MOUSE	GTP-binding protein Di-Ras2	0,000208	0,000184	0,000191	0,000195	0,000194574	1,02507E-05	5,268	0,000246	0,000194	0,000201	0,000215	0,000185	0,000208203	2,37546E-05	11,409
Q9CPQ1	COX6C_MOUSE	Cytochrome c oxidase subunit 6C	0,000474	0,000512	0,000562	0,000513	0,000515385	3,62425E-05	7,032	0,000463	0,000449	0,000506	0,000495	0,000418	0,00046613	3,53713E-05	7,588
P63321	RALA_MOUSE	Ras-related protein Ral-A	0,00027	0,000252	0,000262	0,000263	0,000261656	7,52408E-06	2,876	0,000278	0,000281	0,00028	0,000261	0,000255	0,000271207	1,19569E-05	4,409
P35802	GPM6A_MOUSE	Neuronal membrane glycoprotein M6-a	0,000301	0,000296	0,000383	0,000342	0,000330414	4,05223E-05	12,264	0,000328	0,000344	0,000324	0,000232	0,000245	0,000294544	5,19045E-05	17,622
Q8BJI1	S6A17_MOUSE	Sodium-dependent neutral amino acid transporter SLC6A17	0,000101	0,000118	0,000123	0,000115	0,000114126	9,7275E-06	8,523	0,000117	0,000101	0,000107	0,0001	0,000126	0,000110168	1,11407E-05	10,112
Q922Q1	MARC2_MOUSE	Mitochondrial amidoxime reducing component 2	0,000109	0,000113	0,000121	0,00011	0,000113179	5,46744E-06	4,831	8,65E-05	0,000105	9,98E-05	8,89E-05	8,9E-05	9,376E-05	7,93805E-06	8,466
Q62433	NDRG1_MOUSE	Protein NDRG1	0,000253	0,000251	0,0002	0,000214	0,000229504	2,67806E-05	11,669	0,000292	0,000247	0,000288	0,000303	0,000266	0,000279037	2,25508E-05	8,082
O09061	PSB1_MOUSE	Proteasome subunit beta type-1	0,000246	0,00022	0,000234	0,000245	0,00023641	1,22276E-05	5,172	0,000228	0,000216	0,000247	0,00026	0,000245	0,000239382	1,71013E-05	7,144
Q9ERS2	NDUAD_MOUSE	NADH dehydrogenase [ubiquinone] 1 alpha subcomplex subunit 13	0,000496	0,000556	0,000533	0,000528	0,000528365	2,48018E-05	4,694	0,000488	0,000532	0,000572	0,000568	0,000524	0,00053668	3,46636E-05	6,459
Q99LR1	ABD12_MOUSE	Monoacylglycerol lipase ABHD12	5,97E-05	5,79E-05	5,91E-05	5,65E-05	5,82753E-05	1,41206E-06	2,423	5,23E-05	5,27E-05	5,85E-05	5,11E-05	5,25E-05	5,34355E-05	2,90486E-06	5,436
Q99MR8	MCCA_MOUSE	Methylcrotonoyl-CoA carboxylase subunit alpha, mitochondrial	9,74E-05	0,000117	0,000101	9,68E-05	0,000103059	9,68435E-06	9,397	9,49E-05	0,000101	9,72E-05	8,82E-05	9,33E-05	9,49287E-05	4,71307E-06	4,965
P18242	CATD_MOUSE	Cathepsin D	0,000526	0,00054	0,000563	0,000558	0,000546908	1,68897E-05	3,088	0,000399	0,000481	0,000547	0,00044	0,000454	0,000463791	5,49015E-05	11,838
P20108	PRDX3_MOUSE	Thioredoxin-dependent peroxide reductase, mitochondrial	0,000603	0,000613	0,000887	0,000754	0,000714229	0,000134403	18,818	0,000528	0,0005	0,000522	0,000537	0,000475	0,000512389	2,49603E-05	4,871
Q99L47	F10A1_MOUSE	Hsc70-interacting protein	0,000326	0,00034	0,000303	0,000323	0,000323295	1,52889E-05	4,729	0,000323	0,000309	0,000332	0,000338	0,000335	0,000327431	1,18319E-05	3,614
P60202	MYPR_MOUSE	Myelin proteolipid protein	0,002925	0,002822	0,002013	0,002663	0,002605602	0,000409736	15,725	0,002976	0,003273	0,003359	0,00265	0,002198	0,002890942	0,000477135	16,504
Q9D710	TMX2_MOUSE	Thioredoxin-related transmembrane protein 2	0,000159	0,000159	0,00016	0,000163	0,000160143	1,64714E-06	1,029	0,000146	0,000159	0,000139	0,00014	0,000126	0,000141963	1,20182E-05	8,466
Q9QYF9	NDRG3_MOUSE	Protein NDRG3	0,000424	0,000409	0,000423	0,000423	0,000419707	7,16877E-06	1,708	0,000402	0,000391	0,000417	0,000402	0,000387	0,000399896	1,16322E-05	2,909
P97797	SHPS1_MOUSE	Tyrosine-protein phosphatase non-receptor type substrate 1	0,000374	0,000345	0,000397	0,000342	0,000364614	2,57562E-05	7,064	0,000302	0,000311	0,000274	0,000283	0,00035	0,000303909	2,98522E-05	9,823
Q9QY76	VAPB_MOUSE	Vesicle-associated membrane protein-associated protein B	0,00014	0,000149	0,000136	0,000135	0,000140022	6,47371E-06	4,623	0,000161	0,000159	0,000157	0,000148	0,000138	0,000152511	9,67246E-06	6,342
P57759	ERP29_MOUSE	Endoplasmic reticulum resident protein 29	0,000169	0,000178	0,000191	0,000188	0,000181415	9,7758E-06	5,389	0,000173	0,000151	0,000191	0,00018	0,000176	0,000174215	1,4688E-05	8,431
P84096	RHOG_MOUSE	Rho-related GTP-binding protein RhoG	0,000213	0,000186	0,000181	0,000193	0,000193255	1,41691E-05	7,332	0,000197	0,000209	0,000237	0,000193	0,000175	0,000202292	2,28519E-05	11,297
Q791V5	MTCH2_MOUSE	Mitochondrial carrier homolog 2	0,000541	0,000511	0,000506	0,000519	0,00051906	1,56578E-05	3,017	0,000476	0,000495	0,000503	0,000455	0,000423	0,000470444	3,23839E-05	6,884
P62270	RS18_MOUSE	40S ribosomal protein S18	0,00011	0,000135	0,000144	0,000135	0,000131063	1,48788E-05	11,352	0,000112	0,000123	0,00014	0,000122	0,000118	0,000122768	1,05811E-05	8,619
P61089	UBE2N_MOUSE	Ubiquitin-conjugating enzyme E2 N	0,000613	0,000738	0,000706	0,000703	0,000690105	5,39091E-05	7,812	0,000654	0,000712	0,000709	0,000621	0,000642	0,000667596	4,07913E-05	6,110
Q9EPJ9	ARFG1_MOUSE	ADP-ribosylation factor GTPase-activating protein 1	9,79E-05	8,67E-05	9,69E-05	9,42E-05	9,39287E-05	5,10735E-06	5,437	9,36E-05	8,09E-05	8,02E-05	8,58E-05	8,59E-05	8,52927E-05	5,36869E-06	6,294
Q62421	SH3G3_MOUSE	Endophilin-A3	0,000599	0,000499	0,000486	0,000499	0,000520682	5,23514E-05	10,054	0,000602	0,000462	0,000488	0,000506	0,000496	0,000510519	5,35937E-05	10,498
Q9CRB6	TPPP3_MOUSE	Tubulin polymerization-promoting protein family member 3	9E-05	0,00011	8,95E-05	9,95E-05	9,71664E-05	9,4903E-06	9,767	0,000105	0,00011	0,000103	0,000107	8,43E-05	0,00010185	1,01099E-05	9,926
Q3UGR5	HDHD2_MOUSE	Haloacid dehalogenase-like hydrolase domain-containing protein 2	0,000118	9,57E-05	0,000108	0,000116	0,000109243	1,00341E-05	9,185	0,000101	6,91E-05	9,78E-05	9,69E-05	7,91E-05	8,88045E-05	1,39733E-05	15,735
Q9CQH3	NDUB5_MOUSE	NADH dehydrogenase [ubiquinone] 1 beta subcomplex subunit 5, mitochondrial	0,000446	0,000494	0,000426	0,000457	0,000455438	2,85801E-05	6,275	0,000371	0,000385	0,000409	0,000353	0,000351	0,000373804	2,42523E-05	6,488
Q9DC70	NDUS7_MOUSE	NADH dehydrogenase [ubiquinone] iron-sulfur protein 7, mitochondrial	0,000234	0,000241	0,000237	0,000237	0,000236996	2,98636E-06	1,260	0,000205	0,000216	0,000222	0,000207	0,000179	0,000205943	1,66729E-05	8,096
Q8BKZ9	ODPX_MOUSE	Pyruvate dehydrogenase protein X component, mitochondrial	0,000126	0,000117	0,000118	0,000114	0,00011878	5,26132E-06	4,429	0,000104	9,38E-05	9,87E-05	0,000103	9,69E-05	9,92688E-05	4,27059E-06	4,302
Q9D898	ARP5L_MOUSE	Actin-related protein 2/3 complex subunit 5-like protein	0,000206	0,000208	0,00024	0,000224	0,00021967	1,5665E-05	7,131	0,000222	0,000198	0,000205	0,000198	0,000203	0,000205239	9,95009E-06	4,848
Q9R1P0	PSA4_MOUSE	Proteasome subunit alpha type-4	6,89E-05	7,22E-05	6,88E-05	7,51E-05	7,12621E-05	3,02997E-06	4,252	6,49E-05	2,99E-05	6,53E-05	7,69E-05	5,65E-05	5,86842E-05	1,76561E-05	30,087
P23927	CRYAB_MOUSE	Alpha-crystallin B chain	0,000136	0,000157	0,00012	0,000168	0,000145253	2,12965E-05	14,662	0,000176	0,000188	0,0002	0,000173	0,000152	0,000177776	1,7908E-05	10,073
O54734	OST48_MOUSE	Dolichyl-diphosphooligosaccharide--protein glycosyltransferase 48 kDa subunit	9,01E-05	8,91E-05	8,98E-05	9,2E-05	9,02534E-05	1,26151E-06	1,398	9,21E-05	7,19E-05	9,15E-05	8,17E-05	8,08E-05	8,35963E-05	8,42708E-06	10,081
Q9CQD1	RAB5A_MOUSE	Ras-related protein Rab-5A	0,000135	0,000136	0,000152	0,000139	0,000140707	7,94317E-06	5,645	0,000133	0,000144	0,000143	0,000121	0,000121	0,000132418	1,11911E-05	8,451
P51881	ADT2_MOUSE	ADP/ATP translocase 2	0,001996	0,001691	0,001763	0,001763	0,001802971	0,000132912	7,372	0,00164	0,001674	0,001644	0,001523	0,001461	0,001588435	9,16138E-05	5,768
Q80YN3	BCAS1_MOUSE	Breast carcinoma-amplified sequence 1 homolog	0,000112	0,00011	7,53E-05	0,000105	0,000100461	1,70724E-05	16,994	0,000108	0,0001	0,000101	0,000101	0,000101	0,00010228	3,43752E-06	3,361
P26645	MARCS_MOUSE	Myristoylated alanine-rich C-kinase substrate	0,00018	0,000213	0,000254	0,000217	0,000216006	3,00375E-05	13,906	0,000176	0,000188	0,000208	0,000182	0,000209	0,000192495	1,50545E-05	7,821
P42125	ECI1_MOUSE	Enoyl-CoA delta isomerase 1, mitochondrial	0,000197	0,000183	0,000166	0,000166	0,000177814	1,4881E-05	8,369	0,000169	0,000211	0,00019	0,000162	0,000171	0,000180694	1,98818E-05	11,003
Q8BWG8	ARRB1_MOUSE	Beta-arrestin-1	7,94E-05	7,91E-05	9E-05	8,07E-05	8,2301E-05	5,20752E-06	6,327	8,01E-05	6,72E-05	8,39E-05	9,13E-05	8,34E-05	8,11728E-05	8,84161E-06	10,892
Q8K3H0	DP13A_MOUSE	DCC-interacting protein 13-alpha	4,74E-05	5,2E-05	5,27E-05	5,71E-05	5,22806E-05	3,9837E-06	7,620	5,19E-05	4,3E-05	5,01E-05	5E-05	5,36E-05	4,97432E-05	4,02576E-06	8,093
O70252	HMOX2_MOUSE	Heme oxygenase 2	3,02E-05	2,94E-05	2,82E-05	3,09E-05	2,96679E-05	1,14583E-06	3,862	3,43E-05	3,28E-05	3,81E-05	3,94E-05	3,94E-05	3,67894E-05	3,08618E-06	8,389
Q9CPP6	NDUA5_MOUSE	NADH dehydrogenase [ubiquinone] 1 alpha subcomplex subunit 5	0,000133	0,000177	0,000169	0,00019	0,000167195	2,47729E-05	14,817	0,000137	0,00016	0,000162	0,000156	0,000163	0,000155371	1,07985E-05	6,950
Q8BGB7	ENOPH_MOUSE	Enolase-phosphatase E1	0,00013	0,00013	0,000151	0,000137	0,000137002	9,81914E-06	7,167	0,000131	0,000124	0,000127	0,000115	9,86E-05	0,000119038	1,28026E-05	10,755
P11404	FABPH_MOUSE	Fatty acid-binding protein, heart	3,13E-05	3,91E-05	8,14E-05	5,21E-05	5,09554E-05	2,20287E-05	43,231	3,52E-05	2,53E-05	3,72E-05	3,28E-05	3,52E-05	3,31299E-05	4,66878E-06	14,092
Q8C522	ENDD1_MOUSE	Endonuclease domain-containing 1 protein	0,00015	0,000152	0,000137	0,000132	0,000142476	9,69297E-06	6,803	0,000137	0,000127	0,000143	0,00014	0,00012	0,000133339	9,56816E-06	7,176
Q9CXZ1	NDUS4_MOUSE	NADH dehydrogenase [ubiquinone] iron-sulfur protein 4, mitochondrial	0,00022	0,000208	0,000198	0,00022	0,000211569	1,05431E-05	4,983	0,00017	0,000181	0,000213	0,000166	0,000148	0,000175656	2,4281E-05	13,823
Q9R0Q7	TEBP_MOUSE	Prostaglandin E synthase 3	0,000246	0,00025	0,00025	0,000276	0,000255451	1,41779E-05	5,550	0,00023	0,000183	0,000243	0,000191	0,000168	0,000203079	3,20321E-05	15,773
P02535	K1C10_MOUSE	Keratin, type I cytoskeletal 10	5,75E-05	6,54E-05	6,43E-05	5,52E-05	6,05946E-05	5,00817E-06	8,265	7,47E-05	0,00033	8,03E-05	6,45E-05	5,78E-05	0,000121374	0,000116727	96,171
O88685	PRS6A_MOUSE	26S protease regulatory subunit 6A	6,62E-05	6,64E-05	7,5E-05	6,92E-05	6,92107E-05	4,10485E-06	5,931	6,78E-05	6,04E-05	8,47E-05	7,67E-05	6,44E-05	7,0822E-05	9,82176E-06	13,868
Q63810	CANB1_MOUSE	Calcineurin subunit B type 1	0,000603	0,000625	0,000713	0,000584	0,000631242	5,68286E-05	9,003	0,00048	0,000432	0,000471	0,000432	0,000413	0,000445705	2,86337E-05	6,424
P19536	COX5B_MOUSE	Cytochrome c oxidase subunit 5B, mitochondrial	0,000398	0,000372	0,00046	0,00045	0,000420162	4,20689E-05	10,013	0,000421	0,000442	0,000515	0,000461	0,000423	0,000452531	3,84187E-05	8,490
P54823	DDX6_MOUSE	Probable ATP-dependent RNA helicase DDX6	0,000109	0,00012	0,000126	0,000111	0,000116398	8,01031E-06	6,882	0,000118	9,84E-05	0,000113	0,00012	0,000119	0,000113959	9,09503E-06	7,981
Q640R3	HECAM_MOUSE	Hepatocyte cell adhesion molecule	0,000193	0,000193	0,000159	0,000178	0,000180858	1,65112E-05	9,129	0,000176	0,000236	0,000198	0,000184	0,000177	0,000194295	2,50517E-05	12,894

Q61205	PA1B3_MOUSE	Platelet-activating factor acetylhydrolase IB subunit gamma	5,92E-05	6,23E-05	6,85E-05	7,89E-05	6,72579E-05	8,69979E-06	12,935	5,18E-05	5,79E-05	5,56E-05	5,45E-05	4,87E-05	5,36826E-05	3,57226E-06	6,654
Q8C166	CPNE1_MOUSE	Copine-1	3E-05	2,61E-05	3,17E-05	3,15E-05	2,98209E-05	2,5758E-06	8,638	2,86E-05	2,12E-05	3,1E-05	3,43E-05	2,72E-05	2,84337E-05	4,87307E-06	17,138
Q61206	PA1B2_MOUSE	Platelet-activating factor acetylhydrolase IB subunit beta	0,000557	0,000561	0,000644	0,000612	0,000593341	4,17723E-05	7,040	0,000505	0,000489	0,000534	0,000527	0,000472	0,000505262	2,58399E-05	5,114
P62242	RS8_MOUSE	40S ribosomal protein S8	0,0003	0,000346	0,00036	0,000366	0,000342839	3,0021E-05	8,757	0,0003	0,000324	0,000348	0,000294	0,000303	0,00031372	2,21338E-05	7,055
Q8K3J1	NDUS8_MOUSE	NADH dehydrogenase [ubiquinone] iron-sulfur protein 8, mitochondrial	0,000751	0,000715	0,000671	0,000715	0,000712879	3,28329E-05	4,606	0,000596	0,00057	0,000716	0,000688	0,000617	0,000637383	6,21275E-05	9,747
Q99LB6	MAT2B_MOUSE	Methionine adenosyltransferase 2 subunit beta	6,04E-05	5,47E-05	5,69E-05	5,36E-05	5,63927E-05	2,98647E-06	5,296	5,91E-05	5,13E-05	5,3E-05	5,12E-05	4,75E-05	5,24308E-05	4,24083E-06	8,088
Q99LY9	NDUS5_MOUSE	NADH dehydrogenase [ubiquinone] iron-sulfur protein 5	0,000124	0,000119	0,000126	0,000128	0,00012411	3,83323E-06	3,089	0,000102	0,000113	0,000121	9,75E-05	9,74E-05	0,000106048	1,04588E-05	9,862
P32037	GTR3_MOUSE	Solute carrier family 2, facilitated glucose transporter member 3	0,000199	0,000202	0,000176	0,000179	0,000189257	1,32858E-05	7,020	0,000231	0,000211	0,000216	0,000214	0,000221	0,000218499	7,87423E-06	3,604
Q6PDL0	DC1L2_MOUSE	Cytoplasmic dynein 1 light intermediate chain 2	0,000103	9,43E-05	8,82E-05	8,75E-05	9,32803E-05	7,21932E-06	7,739	9,83E-05	8,36E-05	8,78E-05	8,8E-05	7,92E-05	8,73753E-05	7,09354E-06	8,118
Q3THE2	ML12B_MOUSE	Myosin regulatory light chain 12B	0,000146	0,000135	0,000142	0,000126	0,000137394	8,85656E-06	6,446	0,000144	0,000145	0,000137	0,000108	0,000125	0,000132012	1,53406E-05	11,621
P47963	RL13_MOUSE	60S ribosomal protein L13	0,00032	0,000379	0,000385	0,000426	0,000377546	4,35797E-05	11,543	0,000357	0,000349	0,000382	0,000332	0,000332	0,000350646	2,0681E-05	5,898
P10605	CATB_MOUSE	Cathepsin B	0,000107	0,000115	0,000136	0,000131	0,000122237	1,36915E-05	11,201	0,000129	0,000134	0,000131	0,000107	0,000102	0,000120436	1,45824E-05	12,108
P35979	RL12_MOUSE	60S ribosomal protein L12	9,83E-05	0,000116	0,000121	0,000123	0,000114606	1,1332E-05	9,888	0,00011	0,000132	0,000125	0,000113	0,000128	0,000121536	9,60659E-06	7,904
O35344	IMA4_MOUSE	Importin subunit alpha-4	3,83E-05	4,41E-05	4,33E-05	4,21E-05	4,19651E-05	2,56275E-06	6,107	4,33E-05	3,96E-05	4,22E-05	4,19E-05	3,88E-05	4,11439E-05	1,87152E-06	4,549
Q05816	FABP5_MOUSE	Fatty acid-binding protein, epidermal	0,000333	0,0004	0,000368	0,000391	0,000372941	2,95275E-05	7,917	0,000353	0,000341	0,000407	0,000375	0,000325	0,000360604	3,19602E-05	8,863
Q6ZWV3	RL10_MOUSE	60S ribosomal protein L10	5,43E-05	6,02E-05	6,92E-05	7,43E-05	6,44919E-05	8,975E-06	13,916	6,99E-05	6,77E-05	8,97E-05	7,94E-05	7,5E-05	7,63336E-05	8,7538E-06	11,468
P51410	RL9_MOUSE	60S ribosomal protein L9	0,000163	0,000194	0,00019	0,000216	0,000190769	2,18379E-05	11,447	0,000174	0,000169	0,000197	0,000173	0,000148	0,00017213	1,75337E-05	10,186
Q9JKC6	CEND_MOUSE	Cell cycle exit and neuronal differentiation protein 1	7,2E-05	7,52E-05	4,7E-05	6,25E-05	6,41578E-05	1,264E-05	19,701	7,19E-05	8,82E-05	7,72E-05	7,62E-05	6,49E-05	7,56874E-05	8,49919E-06	11,229
P24288	BCAT1_MOUSE	Branched-chain-amino-acid aminotransferase, cytosolic	0,000179	0,000181	0,000203	0,000195	0,000189348	1,12671E-05	5,950	0,000172	0,000177	0,000175	0,000171	0,000153	0,000169536	9,50921E-06	5,609
Q8JZU2	TXTP_MOUSE	Tricarboxylate transport protein, mitochondrial	0,00013	0,000121	0,000127	0,000134	0,000128066	5,69093E-06	4,444	0,000109	0,000124	0,00013	0,000125	0,000108	0,000119208	1,0155E-05	8,519
P60766	CDC42_MOUSE	Cell division control protein 42 homolog	0,000525	0,000575	0,000507	0,000546	0,000538287	2,9093E-05	5,405	0,000485	0,000522	0,000514	0,000517	0,000455	0,000498557	2,8463E-05	5,709
Q91XL9	OSBL1_MOUSE	Oxysterol-binding protein-related protein 1	7,87E-05	7,81E-05	7,21E-05	6,11E-05	7,24884E-05	8,15309E-06	11,247	8,03E-05	7,26E-05	7,33E-05	7,42E-05	6,89E-05	7,3856E-05	4,15801E-06	5,630
P97370	AT1B3_MOUSE	Sodium/potassium-transporting ATPase subunit beta-3	0,000175	0,000158	0,000134	0,000152	0,00015489	1,68327E-05	10,867	0,000164	0,000165	0,000165	0,000157	0,000133	0,000156926	1,35692E-05	8,647
Q9CPR4	RL17_MOUSE	60S ribosomal protein L17	0,00012	0,000135	0,000126	0,000126	0,000126673	6,44712E-06	5,090	0,000115	0,000121	0,000129	0,000107	0,000106	0,000115698	9,35401E-06	8,085
Q9JM76	ARPC3_MOUSE	Actin-related protein 2/3 complex subunit 3	0,00013	0,000245	0,000242	0,000293	0,000227652	6,90146E-05	30,316	0,000244	0,000258	0,000284	0,000339	0,000307	0,000286358	3,76593E-05	13,151
Q80UW2	FBX2_MOUSE	F-box only protein 2	0,00021	0,000206	0,000199	0,000195	0,000202553	6,59631E-06	3,257	0,000178	0,000169	0,000179	0,000168	0,000138	0,00016635	1,67686E-05	10,080
Q9CQ92	FIS1_MOUSE	Mitochondrial fission 1 protein	7,58E-05	9,73E-05	8,72E-05	8E-05	8,50638E-05	9,40449E-06	11,056	8,83E-05	0,000137	9,74E-05	8,42E-05	9,37E-05	0,000100097	2,11424E-05	21,122
Q9DD18	DTD1_MOUSE	D-tyrosyl-tRNA(Tyr) deacylase 1	9,68E-05	9,23E-05	9,92E-05	0,000102	9,77058E-05	4,26804E-06	4,368	9,52E-05	0,000102	9,61E-05	8,65E-05	7,98E-05	9,18414E-05	8,64549E-06	9,413
Q62277	SYPH_MOUSE	Synaptophysin	0,001115	0,001083	0,001416	0,001409	0,001255747	0,00018136	14,442	0,000989	0,001155	0,001111	0,00119	0,001243	0,001137474	9,59452E-05	8,435
Q99KB8	GLO2_MOUSE	Hydroxyacylglutathione hydrolase, mitochondrial	0,000176	0,000158	0,000177	0,000194	0,000176121	1,45315E-05	8,251	0,000172	0,000195	0,000173	0,000145	0,00013	0,0001628	2,5326E-05	15,557
Q9QZ06	TOLIP_MOUSE	Toll-interacting protein	0,000181	0,000193	0,000193	0,000206	0,000193364	1,05492E-05	5,456	0,000194	0,000184	0,000189	0,00019	0,000168	0,000184991	9,97473E-06	5,392
Q9CQC7	NDUB4_MOUSE	NADH dehydrogenase [ubiquinone] 1 beta subcomplex subunit 4	0,000192	0,000195	0,00019	0,000165	0,000185263	1,35997E-05	7,341	0,000145	0,000185	0,000184	0,000155	0,000163	0,000166292	1,74857E-05	10,515
Q99JY8	PLPP3_MOUSE	Phospholipid phosphatase 3	0,000148	0,000158	0,000167	0,000152	0,000155973	8,21834E-06	5,269	0,000149	0,000179	0,000136	0,000137	0,000137	0,000147531	1,84009E-05	12,473
P01831	THY1_MOUSE	Thy-1 membrane glycoprotein	0,001792	0,001711	0,001892	0,001834	0,001807136	7,62735E-05	4,221	0,001864	0,001766	0,001801	0,001644	0,001574	0,001729714	0,000118269	6,837
Q9CQV8	1433B_MOUSE	14-3-3 protein beta/alpha	0,001272	0,001011	0,00101	0,001262	0,00113859	0,000148027	13,001	0,001103	0,001204	0,00113	0,000963	0,000733	0,0010266	0,000186104	18,128
Q9CR95	NECP1_MOUSE	Adaptin ear-binding coat-associated protein 1	8,85E-05	0,000111	0,000102	0,000102	0,000100885	9,34052E-06	9,259	0,000105	9,42E-05	9,22E-05	9,04E-05	9,89E-05	9,61587E-05	5,8857E-06	6,121
P84244	H33_MOUSE	#N/A	0,000674	0,000926	0,000806	0,000946	0,00083786	0,000125719	15,005	0,000626	0,000765	0,000941	0,00075	0,000687	0,000753914	0,000118133	15,669
P62141	PP1B_MOUSE	Serine/threonine-protein phosphatase PP1-beta catalytic subunit	0,000223	0,000228	0,000232	0,000231	0,000228659	4,224E-06	1,847	0,000244	0,000255	0,000268	0,00024	0,000253	0,000251944	1,08585E-05	4,310
O70493	SNX12_MOUSE	Sorting nexin-12	6,76E-05	6,94E-05	6,97E-05	7,35E-05	7,00454E-05	2,48151E-06	3,543	7,02E-05	7,32E-05	6,98E-05	7,36E-05	6,72E-05	7,07906E-05	2,63336E-06	3,720
P56391	CX6B1_MOUSE	Cytochrome c oxidase subunit 6B1	0,000262	0,000301	0,000392	0,000316	0,000317855	5,45486E-05	17,161	0,000198	0,000299	0,000254	0,000243	0,00029	0,000256585	4,03773E-05	15,736
Q7TNG5	EMAL2_MOUSE	Echinoderm microtubule-associated protein-like 2	3,96E-05	4,16E-05	3,38E-05	3,41E-05	3,72958E-05	3,92405E-06	10,521	3,9E-05	3,92E-05	3,18E-05	3,16E-05	3,17E-05	3,46726E-05	4,03528E-06	11,638
Q9CQ75	NDUA2_MOUSE	NADH dehydrogenase [ubiquinone] 1 alpha subcomplex subunit 2	0,000114	0,000132	0,000151	0,000117	0,000128309	1,70374E-05	13,278	0,000103	0,000123	0,000135	0,000112	0,000118	0,000118149	1,1998E-05	10,155
Q99M71	EPDR1_MOUSE	Mammalian ependymin-related protein 1	0,000214	0,000194	0,000215	0,000237	0,00021516	1,78136E-05	8,279	0,000193	0,000219	0,000187	0,000171	0,000153	0,000184454	2,46887E-05	13,385
P57776	EF1D_MOUSE	Elongation factor 1-delta	0,000191	0,000201	0,000187	0,000199	0,000194382	6,38488E-06	3,285	0,000196	0,000191	0,000201	0,000197	0,000184	0,000193763	6,43869E-06	3,323
Q8BG51	MIRO1_MOUSE	Mitochondrial Rho GTPase 1	7,95E-05	9,16E-05	7,98E-05	8,16E-05	8,31287E-05	5,73784E-06	6,902	7,46E-05	6,66E-05	7,7E-05	8,05E-05	7,63E-05	7,49746E-05	5,17158E-06	6,898
P63028	TCTP_MOUSE	Translationally-controlled tumor protein	0,000116	0,000117	0,000116	0,000117	0,000116529	1,88084E-07	0,161	0,000113	0,000115	0,000114	0,000109	0,000104	0,000111163	4,42153E-06	3,978
P55088	AQP4_MOUSE	Aquaporin-4	7,71E-05	8,72E-05	6,35E-05	7,37E-05	7,53842E-05	9,78252E-06	12,977	0,000105	0,000106	0,000116	9,73E-05	8,87E-05	0,000102404	1,01606E-05	9,922
Q62048	PEA15_MOUSE	Astrocytic phosphoprotein PEA-15	0,000153	0,000199	0,000224	0,000218	0,000198493	3,17876E-05	16,014	0,000155	0,000171	0,000229	0,000213	0,000201	0,000194034	3,05664E-05	15,753
P62301	RS13_MOUSE	40S ribosomal protein S13	0,000254	0,000308	0,000307	0,000317	0,000296545	2,86566E-05	9,663	0,000259	0,000298	0,00026	0,000262	0,000271	0,000270103	1,64235E-05	6,080
Q91WS0	CISD1_MOUSE	CDGSH iron-sulfur domain-containing protein 1	0,0002	0,000226	0,000234	0,000227	0,000221725	1,4704E-05	6,632	0,000194	0,000249	0,000295	0,00027	0,000257	0,000253111	3,71693E-05	14,685
Q5M8N0	CNRP1_MOUSE	CB1 cannabinoid receptor-interacting protein 1	0,000105	0,000115	0,000143	0,000122	0,000121144	1,57716E-05	13,019	0,00013	0,000112	0,000127	0,000113	0,000128	0,000122087	8,76709E-06	7,181
Q9D5T0	ATAD1_MOUSE	ATPase family AAA domain-containing protein 1	3,59E-05	4,17E-05	3,15E-05	3,99E-05	3,72781E-05	4,53113E-06	12,155	3,45E-05	3,35E-05	3,16E-05	4,01E-05	2,73E-05	3,3375E-05	4,6518E-06	13,938
Q9QUR7	PIN1_MOUSE	Peptidyl-prolyl cis-trans isomerase NIMA-interacting 1	0,000104	0,000117	0,00012	0,000126	0,000116573	9,36176E-06	8,031	0,000113	0,000118	0,000118	0,000121	0,000116	0,000117098	3,22576E-06	2,755
Q9DCZ4	MIC26_MOUSE	MICOS complex subunit Mic26	0,000186	0,000188	0,000207	0,000199	0,000194611	9,9108E-06	5,093	0,000156	0,000145	0,000178	0,000166	0,000147	0,000158349	1,39111E-05	8,785
Q9D6J5	NDUB8_MOUSE	NADH dehydrogenase [ubiquinone] 1 beta subcomplex subunit 8, mitochondrial	4,88E-05	5,25E-05	4,07E-05	5,08E-05	4,81752E-05	5,23081E-06	10,858	4,09E-05	1,3E-05	6,26E-05	6,61E-05	5,28E-05	4,70951E-05	2,14119E-05	45,465
P28571	SC6A9_MOUSE	Sodium- and chloride-dependent glycine transporter 1	0,000152	0,000137	0,000101	0,000126	0,000128928	2,1403E-05	16,601	0,000149	0,000137	0,000127	0,000119	0,00012	0,000130638	1,27854E-05	9,787
Q9JJV2	PROF2_MOUSE	Profilin-2	0,000346	0,000568	0,000728	0,000622	0,000566095	0,000160715	28,390	0,000474	0,000486	0,000623	0,000607	0,000581	0,000554216	6,95078E-05	12,542
O89112	LANC1_MOUSE	LanC-like protein 1	2,54E-05	2,51E-05	2,46E-05	2,31E-05	2,45512E-05	1,0268E-06	4,182	2,35E-05	2,14E-05	2,5E-05	1,87E-05	2,35E-05	2,24295E-05	2,42464E-06	10,810
O08585	CLCA_MOUSE	Clathrin light chain A	0,00047	0,000493	0,0005	0,000514	0,000494234	1,82005E-05	3,683	0,000416	0,000373	0,000394	0,000372	0,000376	0,000386323	1,90645E-05	4,935
O08756	HCD2_MOUSE	3-hydroxyacyl-CoA dehydrogenase type-2	0,000245	0,000217	0,000212	0,000213	0,000221686	1,56946E-05	7,080	0,00021	0,00024	0,00021	0,000217	0,000205	0,00021636	1,4093E-05	6,514
O88696	CLPP_MOUSE	ATP-dependent Clp protease proteolytic subunit, mitochondrial	0,000136	0,000138	0,000147	0,000138	0,000140026	5,0117E-06	3,579	0,000139	0,000152	0,00014	0,000136	0,000129	0,000139029	8,36933E-06	6,020
O54984	ASNA_MOUSE	ATPase Asna1	0,000192	0,000207	0,000212	0,000197	0,000202116	8,96204E-06	4,434	0,000204	0,000183	0,000196	0,000192	0,000196	0,00019412	7,69343E-06	3,963
O70251	EF1B_MOUSE	Elongation factor 1-beta	0,00023	0,000248	0,000257	0,000259	0,000248715	1,33816E-05	5,380	0,000245	0,000231	0,000252	0,000211	0,000204	0,000228632	2,10983E-05	9,228
Q06185	ATP5I_MOUSE	ATP synthase subunit e, mitochondrial	0,000323	0,000458	0,000469	0,000406	0,000414146	6,64587E-05	16,047	0,000333	0,000388	0,000459	0,000429	0,000413	0,000404276	4,73972E-05	11,724
P56959	FUS_MOUSE	RNA-binding protein FUS	0,000179	0,000201	0,000214	0,000199	0,000198129	1,45752E-05	7,356	0,000178	0,000157	0,000177	0,000171	0,0002	0,000176797	1,54657E-05	8,748
Q99020	ROAA_MOUSE	Heterogeneous nuclear ribonucleoprotein A/B	0,00024	0,000257	0,000275	0,000265	0,000259144	1,48981E-05	5,749	0,000244	0,00017	0,000223	0,000215	0,00024	0,000218199	2,96755E-05	13,600
Q9CR57	RL14_MOUSE	60S ribosomal protein L14	0,000245	0,000274	0,000266	0,000295	0,000269874	2,06759E-05	7,661	0,00026	0,000267	0,00029	0,000284	0,000248	0,000269809	1,75069E-05	6,489
O35658	C1QBP_MOUSE	Complement component 1 Q subcomponent-binding protein, mitochondrial	0,00029	0,000276	0,000277	0,00029	0,000283222	7,70252E-06	2,720	0,000283	0,000284	0,000282	0,000247	0,000235	0,000266123	2,33486E-05	8,774
P62331	ARF6_MOUSE	ADP-ribosylation factor 6	0,000164	0,000204	0,000222	0,000188	0,00019464	2,43903E-05	12,531	0,000185	0,000134	0,000208	0,000201	0,000171	0,000179949	2,94007E-05	16,338
P62264	RS14_MOUSE	40S ribosomal protein S14	6,47E-05	7,77E-05	8,28E-05	7,28E-05	7,44979E-05	7,69297E-06	10,326	6,79E-05	6,96E-05	7,85E-05	5,8E-05	6,98E-05	6,87693E-05	7,28518E-06	10,594
P99024	TBB5_MOUSE	Tubulin beta-5 chain	0,004638	0,004048	0,004261	0,004711	0,004414639	0,000314027	7,113	0,004625	0,005209	0,005105	0,00581	0,005241	0,00519833	0,000422185	8,122

P62743	AP2S1_MOUSE	AP-2 complex subunit sigma	0,000174	0,000212	0,000251	0,000207	0,000210794	3,1741E-05	15,058	0,000191	0,000217	0,000237	0,000237	0,000193	0,000215046	2,24241E-05	10,428
P17809	GTR1_MOUSE	Solute carrier family 2, facilitated glucose transporter member 1	0,000212	0,000194	0,000174	0,000177	0,000189301	1,72331E-05	9,104	0,000198	0,000204	0,000183	0,000188	0,000179	0,000190365	1,05051E-05	5,518
P70349	HINT1_MOUSE	Histidine triad nucleotide-binding protein 1	6,91E-05	0,000106	0,00026	0,000115	0,000137404	8,41377E-05	61,234	6,79E-05	6,55E-05	8,98E-05	7,19E-05	7,82E-05	7,46548E-05	9,71393E-06	13,012
Q4VAE3	TMM65_MOUSE	Transmembrane protein 65	0,000169	0,000178	0,00018	0,00015	0,000169198	1,3685E-05	8,088	0,000141	0,00014	0,00014	0,000132	7,8E-05	0,000126208	2,72122E-05	21,561
P08228	SODC_MOUSE	Superoxide dismutase [Cu-Zn]	0,00058	0,000601	0,000916	0,000866	0,000740405	0,000174782	23,606	0,000473	0,000477	0,000582	0,000491	0,000492	0,000503075	4,51431E-05	8,973
Q61735	CD47_MOUSE	Leukocyte surface antigen CD47	0,000278	0,000177	0,00019	0,000173	0,000204332	4,95603E-05	24,255	0,000224	0,000258	0,000222	0,000246	0,000243	0,000238711	1,52506E-05	6,389
P63011	RAB3A_MOUSE	Ras-related protein Rab-3A	0,001049	0,000957	0,000995	0,001072	0,001017932	5,19926E-05	5,108	0,001104	0,001112	0,00083	0,001044	0,00105	0,001028022	0,000114847	11,172
P62774	MTPN_MOUSE	Myotrophin	6,7E-05	8,58E-05	0,000106	8,71E-05	8,64272E-05	1,58572E-05	18,347	6,52E-05	7,22E-05	7,26E-05	7,43E-05	8,39E-05	7,36279E-05	6,73283E-06	9,144
Q60692	PSB6_MOUSE	Proteasome subunit beta type-6	7,31E-05	7,2E-05	7,69E-05	7,48E-05	7,4177E-05	2,13829E-06	2,883	7,36E-05	7,89E-05	8,02E-05	6,92E-05	6,01E-05	7,24111E-05	8,15327E-06	11,260
Q61234	SNTA1_MOUSE	Alpha-1-syntrophin	3,16E-05	3,11E-05	3,17E-05	3,4E-05	3,21153E-05	1,32025E-06	4,111	3,2E-05	2,97E-05	2,98E-05	3,01E-05	2,76E-05	2,98414E-05	1,54317E-06	5,171
P63163	RSMN_MOUSE	Small nuclear ribonucleoprotein-associated protein N	5,52E-05	6,14E-05	6,46E-05	6,71E-05	6,20663E-05	5,17066E-06	8,331	5,66E-05	5,18E-05	6,04E-05	5,56E-05	5,27E-05	5,544E-05	3,43043E-06	6,188
Q9D3D9	ATPD_MOUSE	ATP synthase subunit delta, mitochondrial	0,000359	0,000354	0,000353	0,000362	0,000356887	4,44694E-06	1,246	0,000295	0,000382	0,000409	0,000305	0,0003	0,000338374	5,32871E-05	15,748
Q9CQR4	ACO13_MOUSE	Acyl-coenzyme A thioesterase 13	0,000102	0,000124	0,000114	0,000115	0,000113529	9,09321E-06	8,010	0,000102	0,000118	0,000124	0,000112	0,000101	0,000111411	1,01559E-05	9,116
Q62425	NDUA4_MOUSE	Cytochrome c oxidase subunit NDUFA4	0,000552	0,000604	0,000616	0,000562	0,000583321	3,12219E-05	5,352	0,000515	0,000536	0,000551	0,000568	0,000504	0,000534778	2,61965E-05	4,899
Q9D1G1	RAB1B_MOUSE	Ras-related protein Rab-1B	0,000189	0,000229	0,000223	0,000206	0,000211867	1,81031E-05	8,545	0,000223	0,000215	0,000189	0,000214	0,000216	0,000211509	1,3077E-05	6,183
P26369	U2AF2_MOUSE	Splicing factor U2AF 65 kDa subunit	3,5E-05	3,68E-05	4,33E-05	3,79E-05	3,82355E-05	3,56477E-06	9,323	2,32E-05	2,29E-05	2,9E-05	3,08E-05	2,77E-05	2,67312E-05	3,52529E-06	13,188
Q9CRB8	MTFP1_MOUSE	Mitochondrial fission process protein 1	5,06E-05	6,16E-05	4,38E-05	5,27E-05	5,22069E-05	7,3222E-06	14,025	4,74E-05	4,79E-05	4,31E-05	4,62E-05	3,41E-05	4,37408E-05	5,71228E-06	13,059
P10639	THIO_MOUSE	Thioredoxin	0,000136	0,000153	0,000172	0,000145	0,000151321	1,5238E-05	10,070	0,00012	0,000134	0,000124	0,000123	0,000107	0,00012147	9,8682E-06	8,124
Q8C0P5	COR2A_MOUSE	Coronin-2A	4,85E-05	3,06E-05	2,94E-05	3,23E-05	3,52051E-05	8,95368E-06	25,433	3,37E-05	2,92E-05	2,71E-05	3,21E-05	2,6E-05	2,95966E-05	3,27149E-06	11,054
Q9CQX2	CYB5B_MOUSE	Cytochrome b5 type B	0,000176	0,000194	0,000186	0,000169	0,00018121	1,08241E-05	5,973	0,000149	0,000161	0,000166	0,000148	0,000157	0,000156342	7,58107E-06	4,849
Q91ZZ3	SYUB_MOUSE	Beta-synuclein	0,000677	0,000716	0,000759	0,000728	0,000719918	3,41708E-05	4,746	0,000615	0,000695	0,000648	0,00059	0,000616	0,000632949	4,04078E-05	6,384
P63082	VATL_MOUSE	V-type proton ATPase 16 kDa proteolipid subunit	4,03E-05	3,68E-05	7,27E-05	4,72E-05	4,92354E-05	1,62299E-05	32,964	6,81E-05	8,54E-05	9,74E-05	9,93E-05	0,000103	9,06988E-05	1,42877E-05	15,753
Q8BJH1	ZC21A_MOUSE	Zinc finger C2HC domain-containing protein 1A	7E-06	7,89E-06	8,65E-06	7,27E-06	7,70077E-06	7,33553E-07	9,526	8,48E-06	8,73E-06	8,89E-06	8,55E-06	9,43E-06	8,81672E-06	3,75241E-07	4,256
Q60771	CLD11_MOUSE	Claudin-11	4,88E-05	5,51E-05	4,48E-05	4,26E-05	4,78304E-05	5,49862E-06	11,496	5,85E-05	7,32E-05	7,58E-05	5,51E-05	5,32E-05	6,3166E-05	1,05861E-05	16,759
O35857	TIM44_MOUSE	Mitochondrial import inner membrane translocase subunit TIM44	2,75E-05	2,37E-05	2,98E-05	2,48E-05	2,64321E-05	2,76765E-06	10,471	2,13E-05	2,13E-05	2,11E-05	1,97E-05	1,61E-05	1,98706E-05	2,22119E-06	11,178
E9PUL5	PRRT2_MOUSE	Proline-rich transmembrane protein 2	6,34E-05	5,46E-05	4,37E-05	4,74E-05	5,22565E-05	8,6652E-06	16,582	5,85E-05	5,33E-05	4,7E-05	4,57E-05	4,29E-05	4,94949E-05	6,31041E-06	12,750
P10922	H10_MOUSE	Histone H1.0	0,000203	0,00028	0,000245	0,000284	0,000252951	3,77497E-05	14,924	0,00021	0,000255	0,000303	0,00033	0,000283	0,000276209	4,60592E-05	16,675
Q8VDP6	CDIPT_MOUSE	CDP-diacylglycerol--inositol 3-phosphatidyltransferase	6,5E-05	6,67E-05	8,07E-05	6,65E-05	6,97528E-05	7,35045E-06	10,538	7,74E-05	7,88E-05	8,18E-05	8,68E-05	7,92E-05	8,08141E-05	3,71216E-06	4,593
P26883	FKB1A_MOUSE	Peptidyl-prolyl cis-trans isomerase FKBP1A	0,000187	0,000297	0,000492	0,000325	0,000325358	0,000126108	38,760	0,000203	0,000222	0,000305	0,000266	0,000318	0,000262908	5,0318E-05	19,139
P03888	NU1M_MOUSE	NADH-ubiquinone oxidoreductase chain 1	9,29E-05	7,66E-05	7,29E-05	6,48E-05	7,67963E-05	1,18213E-05	15,393	7,14E-05	6,54E-05	5,58E-05	5,7E-05	6,22E-05	6,23518E-05	6,36517E-06	10,208
O08919	NUMBL_MOUSE	Numb-like protein	3,81E-05	4,82E-05	5,39E-05	5,25E-05	4,81555E-05	7,13126E-06	14,809	4,98E-05	4,41E-05	4,96E-05	4,51E-05	5,36E-05	4,8451E-05	3,87419E-06	7,996
Q8VD33	SGTB_MOUSE	Small glutamine-rich tetratricopeptide repeat-containing protein beta	3,46E-05	3,31E-05	2,97E-05	3,06E-05	3,19962E-05	2,27693E-06	7,116	3,43E-05	3,1E-05	3,06E-05	3,38E-05	2,82E-05	3,15638E-05	2,50947E-06	7,950
Q9CPU4	MGST3_MOUSE	Microsomal glutathione S-transferase 3	0,000287	0,000262	0,000306	0,000236	0,000272671	3,02912E-05	11,109	0,000263	0,00022	0,000236	0,000243	0,000227	0,000237954	1,66596E-05	7,001
P03930	ATP8_MOUSE	ATP synthase protein 8	5,36E-05	7,56E-05	8,87E-05	6,68E-05	7,11801E-05	1,47356E-05	20,702	5,82E-05	5,6E-05	6,82E-05	7,08E-05	5,62E-05	6,18891E-05	7,08671E-06	11,451
Q80WJ7	LYRIC_MOUSE	Protein LYRIC	5,55E-05	6,99E-05	5,88E-05	6,68E-05	6,27365E-05	6,71929E-06	10,710	5,18E-05	4,79E-05	5,37E-05	4,81E-05	5,8E-05	5,19067E-05	4,22093E-06	8,132
P62320	SMD3_MOUSE	Small nuclear ribonucleoprotein Sm D3	7,89E-05	0,000111	0,000109	9,53E-05	9,84301E-05	1,47047E-05	14,939	9,19E-05	8,43E-05	0,000112	0,000115	0,0001	0,000100738	1,30505E-05	12,955
Q99JI6	RAP1B_MOUSE	Ras-related protein Rap-1b	5,82E-05	6,15E-05	6,07E-05	6,91E-05	6,23878E-05	4,70475E-06	7,541	6,47E-05	7,22E-05	8,59E-05	9,06E-05	7,61E-05	7,78826E-05	1,04191E-05	13,378
Q62442	VAMP1_MOUSE	Vesicle-associated membrane protein 1	0,000137	0,000209	0,000176	0,000169	0,000172988	2,94999E-05	17,053	0,000162	0,000172	0,000188	0,000157	0,000137	0,000163035	1,907E-05	11,697
Q922J6	TSN2_MOUSE	Tetraspanin-2	4,28E-05	3,92E-05	3,16E-05	4,48E-05	3,9593E-05	5,82957E-06	14,724	4,08E-05	6,08E-05	5,23E-05	4,28E-05	3,6E-05	4,6557E-05	9,93375E-06	21,337
Q6PFR5	TRA2A_MOUSE	Transformer-2 protein homolog alpha	3,35E-05	3,31E-05	3,85E-05	3,63E-05	3,53669E-05	2,52849E-06	7,149	3,21E-05	1,71E-05	3,42E-05	3,71E-05	3,02E-05	3,01353E-05	7,70105E-06	25,555
P62838	UB2D2_MOUSE	Ubiquitin-conjugating enzyme E2 D2	0,000144	0,000178	0,000178	0,000148	0,000162066	1,82557E-05	11,264	0,000137	0,000144	0,000149	0,000146	0,000132	0,000140547	6,3535E-06	4,521
P0DN34	NDUB1_MOUSE	NADH dehydrogenase [ubiquinone] 1 beta subcomplex subunit 1	5,53E-05	5,23E-05	6,04E-05	5,74E-05	5,63543E-05	3,42058E-06	6,070	4,4E-05	4,63E-05	4,75E-05	4,18E-05	4,04E-05	4,39976E-05	2,95122E-06	6,708
Q9R1Q9	VAS1_MOUSE	V-type proton ATPase subunit S1	0,00026	0,000244	0,000279	0,00027	0,000263277	1,50163E-05	5,704	0,000222	0,000219	0,000268	0,000289	0,000241	0,000247771	2,9985E-05	12,102
O55100	SNG1_MOUSE	Synaptogyrin-1	0,000654	0,000602	0,000681	0,000643	0,000645005	3,28246E-05	5,089	0,000708	0,000619	0,000588	0,000601	0,000627	0,000628472	4,68594E-05	7,456
Q9CR26	VTA1_MOUSE	Vacuolar protein sorting-associated protein VTA1 homolog	3,59E-05	3,59E-05	3,4E-05	3,33E-05	3,47731E-05	1,34621E-06	3,871	4,15E-05	3,32E-05	3,86E-05	3,71E-05	3,77E-05	3,76188E-05	3,01044E-06	8,003
P56135	ATPK_MOUSE	ATP synthase subunit f, mitochondrial	0,000486	0,000542	0,000702	0,000522	0,000562705	9,54293E-05	16,959	0,000444	0,000525	0,000579	0,00054	0,000556	0,000528687	5,1145E-05	9,674
Q61990	PCBP2_MOUSE	Poly(rC)-binding protein 2	0,000214	0,000225	0,000227	0,000228	0,000223563	6,51337E-06	2,913	0,000196	0,000195	0,000205	0,000182	0,000178	0,000191149	1,11686E-05	5,843
P48771	CX7A2_MOUSE	Cytochrome c oxidase subunit 7A2, mitochondrial	0,000138	0,000151	0,00016	0,00013	0,000144743	1,33435E-05	9,219	0,000125	0,000146	0,000156	0,00017	0,00014	0,000147367	1,67134E-05	11,341
P43274	H14_MOUSE	Histone H1.4	0,000151	0,000171	0,000162	0,000165	0,000162092	8,02401E-06	4,950	0,000139	0,000136	0,000176	0,000152	0,000174	0,000155307	1,86578E-05	12,013
P43275	H11_MOUSE	Histone H1.1	0,000211	0,000263	0,000261	0,000291	0,000256332	3,30214E-05	12,882	0,000224	0,000234	0,000311	0,000253	0,000266	0,000257575	3,39412E-05	13,177
Q9ER58	TICN2_MOUSE	Testican-2	2,08E-05	2,04E-05	1,73E-05	2,1E-05	1,98726E-05	1,73096E-06	8,710	1,88E-05	1,3E-05	1,88E-05	1,72E-05	1,77E-05	1,71054E-05	2,40827E-06	14,079
Q80X50	UBP2L_MOUSE	Ubiquitin-associated protein 2-like	2,17E-05	2,32E-05	2,07E-05	2,26E-05	2,20675E-05	1,09733E-06	4,973	2,41E-05	1,72E-05	1,86E-05	1,8E-05	1,91E-05	1,94216E-05	2,73367E-06	14,075
Q9JKV1	ADRM1_MOUSE	Proteasomal ubiquitin receptor ADRM1	8,59E-06	1,03E-05	7,36E-06	9,11E-06	8,83352E-06	1,21213E-06	13,722	8,85E-06	9,81E-06	9,9E-06	6,31E-06	6,55E-06	8,28342E-06	1,74438E-06	21,059
P48725	PCNT_MOUSE	#N/A	5,34E-05	5,69E-05	5,75E-05	5,78E-05	5,6383E-05	1,99652E-06	3,541	5,44E-05	5,32E-05	5,6E-05	5,7E-05	5,42E-05	5,49513E-05	1,51452E-06	2,756
Q8BI08	MAL2_MOUSE	#N/A	0,000179	0,000156	0,000173	0,000156	0,000166032	1,16747E-05	7,032	0,000174	0,000164	0,000154	0,000157	0,000159	0,000161717	8,1223E-06	5,023
Q9CPX8	QCR10_MOUSE	#N/A	8,91E-05	0,000113	0,000106	8,71E-05	9,8814E-05	1,26142E-05	12,766	9,48E-05	8,34E-05	8,42E-05	0,000103	7,52E-05	8,81264E-05	1,08399E-05	12,300
Q05920	PYC_MOUSE	Pyruvate carboxylase, mitochondrial	0,000413	0,000416	0,000394	0,000404	0,00040662	9,58415E-06	2,357	0,000446	0,000452	0,000426	0,000421	0,000386	0,000426322	2,59307E-05	6,082
P28660	NCKP1_MOUSE	Nck-associated protein 1	0,000395	0,00042	0,000433	0,000426	0,000418402	1,67628E-05	4,006	0,000443	0,000371	0,00037	0,000379	0,000467	0,00040615	4,57083E-05	11,254
Q8BGQ7	SYAC_MOUSE	Alanine--tRNA ligase, cytoplasmic	0,0002	0,000229	0,000209	0,000211	0,000212076	1,2077E-05	5,695	0,000221	0,000218	0,000235	0,000229	0,000239	0,000228363	9,24296E-06	4,047
Q78PY7	SND1_MOUSE	Staphylococcal nuclease domain-containing protein 1	4,58E-05	5,37E-05	5,38E-05	5,82E-05	5,28883E-05	5,13391E-06	9,707	4,99E-05	5,05E-05	5,92E-05	5,14E-05	5,65E-05	5,35261E-05	4,10576E-06	7,671
P28738	KIF5C_MOUSE	Kinesin heavy chain isoform 5C	5,99E-05	7,16E-05	6,27E-05	6,7E-05	6,53063E-05	5,11568E-06	7,833	7,75E-05	6,8E-05	6,96E-05	7,53E-05	7,75E-05	7,35906E-05	4,49579E-06	6,109
Q9Z1G3	VATC1_MOUSE	V-type proton ATPase subunit C 1	5,73E-05	5,6E-05	4,87E-05	5,8E-05	5,49954E-05	4,25405E-06	7,735	6,54E-05	5,21E-05	6,5E-05	6,03E-05	4,76E-05	5,80949E-05	7,95591E-06	13,695
Q922S4	PDE2A_MOUSE	cGMP-dependent 3',5'-cyclic phosphodiesterase	0,00022	0,000251	0,000259	0,00019	0,000230117	3,18178E-05	13,827	0,000214	0,000243	0,000237	0,000266	0,000303	0,000252511	3,38363E-05	13,400
O35098	DPYL4_MOUSE	Dihydropyrimidinase-related protein 4	5,92E-05	7,74E-05	7,05E-05	6,99E-05	6,9247E-05	7,4915E-06	10,819	7,75E-05	6,99E-05	8,07E-05	8,19E-05	7,4E-05	7,68015E-05	4,94398E-06	6,437
O35136	NCAM2_MOUSE	Neural cell adhesion molecule 2	0,000153	0,000165	0,000168	0,000165	0,000162712	6,92405E-06	4,255	0,000191	0,000184	0,000178	0,000165	0,000179	0,000179229	9,4481E-06	5,272
P14685	PSMD3_MOUSE	26S proteasome non-ATPase regulatory subunit 3	9,78E-05	9,59E-05	0,000101	0,000105	0,000100016	4,07836E-06	4,078	0,00011	0,000101	0,000121	0,000114	0,000106	0,000110545	7,56691E-06	6,845
P10630	IF4A2_MOUSE	Eukaryotic initiation factor 4A-II	0,000103	0,000111	0,000119	0,000109	0,000110631	6,68186E-06	6,040	9,14E-05	9,99E-05	0,000108	0,000106	9,53E-05	0,000100097	6,95557E-06	6,949
Q9CYT6	CAP2_MOUSE	Adenylyl cyclase-associated protein 2	0,00047	0,000475	0,000478	0,000464	0,000471857	6,14116E-06	1,301	0,000492	0,00042	0,000454	0,00047	0,000477	0,000462578	2,77688E-05	6,003
Q9CWJ9	PUR9_MOUSE	Bifunctional purine biosynthesis protein PURH	0,00019	0,000182	0,000203	0,000212	0,000196806	1,36601E-05	6,941	0,000125	0,000166	0,00018	0,000197	0,000183	0,000170283	2,76371E-05	16,230
Q9DCD0	6PGD_MOUSE	6-phosphogluconate dehydrogenase, decarboxylating	0,000108	0,000119	0,000116	0,000119	0,000115473	5,27975E-06	4,572	0,000116	0,000102	0,00013	0,000139	0,000121	0,000121615	1,38506E-05	11,389
Q9JLB0	MPP6_MOUSE	MAGUK p55 subfamily member 6	0,000302	0,000291	0,000252	0,000286	0,000282651	2,15104E-05	7,610	0,000316	0,000307	0,000295	0,000309	0,000283	0,000302355	1,3224E-05	4,374

P06801	MAOX_MOUSE	NADP-dependent malic enzyme	0,000136	0,00014	0,000122	0,000135	0,000133398	7,78884E-06	5,839	0,000138	0,000133	0,000149	0,000144	0,000135	0,000140011	6,77314E-06	4,838
O88544	CSN4_MOUSE	COP9 signalosome complex subunit 4	0,000244	0,000285	0,000248	0,00026	0,000259242	1,85066E-05	7,139	0,000217	0,000249	0,000253	0,000268	0,000265	0,000250537	2,03152E-05	8,109
P16460	ASSY_MOUSE	Argininosuccinate synthase	0,000158	0,000164	0,000177	0,000165	0,000166014	7,74062E-06	4,663	0,000146	0,000161	0,000194	0,000172	0,000173	0,000169371	1,74614E-05	10,310
Q8BUV3	GEPH_MOUSE	Gephyrin	2,71E-05	2,82E-05	2,67E-05	2,74E-05	2,73681E-05	6,08401E-07	2,223	2,91E-05	2,61E-05	2,89E-05	2,86E-05	2,75E-05	2,80419E-05	1,25327E-06	4,469
P61027	RAB10_MOUSE	Ras-related protein Rab-10	0,000145	0,000184	0,000276	0,000164	0,000192145	5,82158E-05	30,298	0,000161	0,00015	0,000195	0,000217	0,000164	0,000177425	2,77221E-05	15,625
Q8BG32	PSD11_MOUSE	26S proteasome non-ATPase regulatory subunit 11	0,000155	0,000164	0,000172	0,000162	0,000163316	6,94269E-06	4,251	0,000174	0,00015	0,000165	0,000182	0,000161	0,00016638	1,2466E-05	7,492
Q9CR68	UCRI_MOUSE	Cytochrome b-c1 complex subunit Rieske, mitochondrial	2,35E-05	2,13E-05	1,45E-05	2,13E-05	2,01751E-05	3,90083E-06	19,335	1,81E-05	3E-05	1,91E-05	1,97E-05	1,54E-05	2,04639E-05	5,60184E-06	27,374
P24549	AL1A1_MOUSE	Retinal dehydrogenase 1	0,000211	0,000158	0,000164	0,000193	0,000181226	2,49332E-05	13,758	0,000186	0,000142	0,000179	0,000211	0,000207	0,000184682	2,74404E-05	14,858
O88485	DC1I1_MOUSE	Cytoplasmic dynein 1 intermediate chain 1	0,000246	0,000286	0,000266	0,000253	0,000262982	1,77957E-05	6,767	0,000281	0,00024	0,000273	0,000258	0,000269	0,000264366	1,59014E-05	6,015
Q8JZN5	ACAD9_MOUSE	Acyl-CoA dehydrogenase family member 9, mitochondrial	7,4E-05	7,12E-05	6,25E-05	6,8E-05	6,89419E-05	4,91899E-06	7,135	7,58E-05	6,47E-05	6,53E-05	5,58E-05	6,88E-05	6,60675E-05	7,22213E-06	10,931
Q61481	PDE1A_MOUSE	Calcium/calmodulin-dependent 3',5'-cyclic nucleotide phosphodiesterase 1A	0,00013	0,000134	0,000153	0,000139	0,000138764	1,00206E-05	7,221	0,000129	0,000108	0,000137	0,000135	0,000134	0,000128477	1,18615E-05	9,232
P53994	RAB2A_MOUSE	Ras-related protein Rab-2A	8,11E-05	9,55E-05	0,000106	9,2E-05	9,36041E-05	1,01873E-05	10,883	7,9E-05	8,81E-05	9,03E-05	0,0001	8,96E-05	8,94581E-05	7,55459E-06	8,445
P12970	RL7A_MOUSE	60S ribosomal protein L7a	0,000102	0,000112	0,000122	0,000115	0,000112591	8,27512E-06	7,350	0,000107	0,000107	0,000106	9,41E-05	9,33E-05	0,000101337	7,00616E-06	6,914
Q9QUP5	HPLN1_MOUSE	Hyaluronan and proteoglycan link protein 1	4,86E-05	6,05E-05	6,12E-05	6,08E-05	5,77773E-05	6,11182E-06	10,578	5,25E-05	5,17E-05	4,51E-05	4,46E-05	4,53E-05	4,78383E-05	3,88501E-06	8,121
Q7TMK9	HNRPQ_MOUSE	Heterogeneous nuclear ribonucleoprotein Q	0,000111	0,000113	9,49E-05	0,000119	0,000109479	1,02813E-05	9,391	0,000123	0,00011	0,000122	0,000112	0,000125	0,000118433	7,23776E-06	6,111
Q9Z140	CPNE6_MOUSE	Copine-6	0,000146	0,000273	0,000298	0,000332	0,000262037	8,12617E-05	31,011	0,000182	0,000113	0,000247	0,00022	0,000227	0,000197978	5,28632E-05	26,701
O88712	CTBP1_MOUSE	C-terminal-binding protein 1	0,00029	0,000298	0,00031	0,000304	0,00030046	8,48883E-06	2,825	0,000325	0,000277	0,000306	0,00028	0,000312	0,000300007	2,08057E-05	6,935
P53026	RL10A_MOUSE	60S ribosomal protein L10a	4,1E-05	4,14E-05	4,56E-05	4,88E-05	4,42057E-05	3,70125E-06	8,373	4,55E-05	5,31E-05	5,15E-05	4,02E-05	4,15E-05	4,63663E-05	5,76443E-06	12,432
Q9Z2W0	DNPEP_MOUSE	Aspartyl aminopeptidase	7,43E-05	6,65E-05	7,08E-05	6,82E-05	6,9962E-05	3,37905E-06	4,830	6,01E-05	5,44E-05	5,59E-05	6,39E-05	6,24E-05	5,93406E-05	4,08218E-06	6,879
O55042	SYUA_MOUSE	Alpha-synuclein	0,000141	0,000612	0,000757	0,000654	0,000540993	0,000273469	50,549	0,0005	0,000532	0,000554	0,000562	0,000608	0,00055116	3,95151E-05	7,169
Q61081	CDC37_MOUSE	Hsp90 co-chaperone Cdc37	7,9E-05	9,03E-05	9,02E-05	9,13E-05	8,76975E-05	5,83326E-06	6,652	9,25E-05	8,75E-05	0,000104	0,0001	9,71E-05	9,62691E-05	6,45643E-06	6,707
Q3TC72	FAHD2_MOUSE	Fumarylacetoacetate hydrolase domain-containing protein 2A	5,85E-05	6,09E-05	6,83E-05	6,07E-05	6,2097E-05	4,25803E-06	6,857	6,11E-05	6E-05	5,46E-05	5,86E-05	5,6E-05	5,80689E-05	2,72291E-06	4,689
Q91VM9	IPYR2_MOUSE	Inorganic pyrophosphatase 2, mitochondrial	6,89E-05	6,9E-05	6,88E-05	6,99E-05	6,9157E-05	4,85806E-07	0,702	6,59E-05	3,88E-05	6,33E-05	5,64E-05	5,61E-05	5,60697E-05	1,05831E-05	18,875
Q9QVP9	FAK2_MOUSE	Protein-tyrosine kinase 2-beta	2,56E-05	3,58E-05	3,47E-05	3,54E-05	3,28707E-05	4,88962E-06	14,875	2,43E-05	1,93E-05	2,54E-05	2,69E-05	2,72E-05	2,46236E-05	3,22082E-06	13,080
Q9WV60	GSK3B_MOUSE	Glycogen synthase kinase-3 beta	0,000135	0,000128	0,000147	0,000127	0,000134147	9,01117E-06	6,717	0,000128	0,000113	0,000145	0,00013	0,000135	0,000130153	1,17202E-05	9,005
Q8BTG7	NDRG4_MOUSE	Protein NDRG4	3,38E-05	3,85E-05	3,7E-05	3,62E-05	3,63871E-05	1,98102E-06	5,444	4,28E-05	3,61E-05	4,95E-05	4,32E-05	4,61E-05	4,35438E-05	4,98218E-06	11,442
Q8BLF1	NCEH1_MOUSE	Neutral cholesterol ester hydrolase 1	5,64E-05	4,54E-05	5,22E-05	4,57E-05	4,99501E-05	5,34535E-06	10,701	4,34E-05	4,1E-05	5,38E-05	6,15E-05	5,61E-05	5,11545E-05	8,68542E-06	16,979
Q9R0X4	ACOT9_MOUSE	Acyl-coenzyme A thioesterase 9, mitochondrial	0,000137	0,000134	0,000141	0,000136	0,00013706	2,97293E-06	2,169	0,000132	0,000127	0,000132	0,000138	0,000124	0,000130697	5,21952E-06	3,994
Q8R0A7	K0513_MOUSE	Uncharacterized protein KIAA0513	2,85E-05	3,37E-05	4,17E-05	2,79E-05	3,29324E-05	6,37735E-06	19,365	2,71E-05	3,1E-05	3,17E-05	2,93E-05	3,07E-05	2,99659E-05	1,81712E-06	6,064
P42208	SEPT2_MOUSE	Septin-2	0,000159	0,000147	0,000148	0,000154	0,000151756	5,59209E-06	3,685	0,000156	0,000143	0,000164	0,000188	0,000158	0,000161723	1,62531E-05	10,050
Q9Z2I8	SUCB2_MOUSE	Succinate--CoA ligase [GDP-forming] subunit beta, mitochondrial	2,98E-05	3,49E-05	3,1E-05	2,99E-05	3,13952E-05	2,38429E-06	7,594	3,44E-05	2,74E-05	3,37E-05	2,75E-05	3,03E-05	3,0656E-05	3,31127E-06	10,801
Q9QXV0	PCSK1_MOUSE	ProSAAS	0,000109	0,00013	0,000158	0,000155	0,000137871	2,30986E-05	16,754	9,32E-05	0,000106	0,000143	0,000131	0,000118	0,00011823	1,95341E-05	16,522
Q8BIZ1	ANS1B_MOUSE	Ankyrin repeat and sterile alpha motif domain-containing protein 1B	8,53E-05	8,7E-05	0,000126	0,000125	0,00010596	2,29089E-05	21,620	0,000107	6,33E-05	8,96E-05	9,58E-05	0,000112	9,34633E-05	1,90433E-05	20,375
Q8K0S0	PHYIP_MOUSE	Phytanoyl-CoA hydroxylase-interacting protein	7,54E-05	9,41E-05	0,00011	9,59E-05	9,39056E-05	1,43E-05	15,228	8,79E-05	7,87E-05	8,21E-05	7,6E-05	9,39E-05	8,37101E-05	7,22354E-06	8,629
O35381	AN32A_MOUSE	Acidic leucine-rich nuclear phosphoprotein 32 family member A	5,96E-05	5,43E-05	6,42E-05	5,79E-05	5,90286E-05	4,09269E-06	6,933	6,27E-05	6,48E-05	6,35E-05	6,51E-05	5,77E-05	6,27554E-05	2,97512E-06	4,741
P19253	RL13A_MOUSE	60S ribosomal protein L13a	5,9E-05	7,27E-05	8,54E-05	8,33E-05	7,50834E-05	1,20679E-05	16,073	6,52E-05	7,74E-05	8,2E-05	6,98E-05	7,23E-05	7,33245E-05	6,55488E-06	8,940
P61082	UBC12_MOUSE	NEDD8-conjugating enzyme Ubc12	5,01E-05	5,75E-05	5,76E-05	5,62E-05	5,53545E-05	3,55557E-06	6,423	4,49E-05	5,09E-05	5,59E-05	4,78E-05	4,18E-05	4,82557E-05	5,45458E-06	11,303
Q9JKB1	UCHL3_MOUSE	Ubiquitin carboxyl-terminal hydrolase isozyme L3	3,78E-05	3,5E-05	3,67E-05	3,86E-05	3,69989E-05	1,54809E-06	4,184	4,15E-05	3,59E-05	4,03E-05	4,14E-05	3,89E-05	3,95957E-05	2,3051E-06	5,822
P46471	PRS7_MOUSE	26S protease regulatory subunit 7	0,000107	0,000114	0,000126	0,000114	0,000115232	7,9961E-06	6,939	0,000125	0,000106	0,000119	0,000115	0,000108	0,000114592	7,96142E-06	6,948
Q8CGF6	WDR47_MOUSE	WD repeat-containing protein 47	0,000113	0,000117	0,000119	0,000106	0,000113852	5,66297E-06	4,974	0,000107	0,000104	0,000115	0,000102	0,000107	0,000107124	4,78928E-06	4,471
E9Q8I9	FRY_MOUSE	Protein furry homolog	1,59E-05	1,59E-05	1,63E-05	1,54E-05	1,58738E-05	3,41931E-07	2,154	1,7E-05	1,38E-05	1,33E-05	1,27E-05	1,91E-05	1,51712E-05	2,75472E-06	18,158
Q8BLE7	VGLU2_MOUSE	Vesicular glutamate transporter 2	8,17E-05	0,000117	9,41E-05	9,73E-05	9,75204E-05	1,4643E-05	15,015	0,000101	0,00011	9,85E-05	8,73E-05	9,68E-05	9,86609E-05	8,1474E-06	8,258
P45952	ACADM_MOUSE	Medium-chain specific acyl-CoA dehydrogenase, mitochondrial	9,55E-05	9,21E-05	9,77E-05	9,74E-05	9,56672E-05	2,57318E-06	2,690	9,34E-05	9,81E-05	9,45E-05	8,63E-05	8,94E-05	9,23314E-05	4,56363E-06	4,943
Q8BJU0	SGTA_MOUSE	Small glutamine-rich tetratricopeptide repeat-containing protein alpha	3,49E-05	3,56E-05	4,07E-05	3,46E-05	3,64645E-05	2,87876E-06	7,895	3,7E-05	3,59E-05	3,46E-05	3,4E-05	3,04E-05	3,4391E-05	2,49619E-06	7,258
P52760	UK114_MOUSE	Ribonuclease UK114	0,000176	0,000203	0,000202	0,000206	0,000196586	1,39287E-05	7,085	0,000183	0,000264	0,000213	0,000197	0,000155	0,000202422	4,03554E-05	19,936
Q9JM14	NT5C_MOUSE	5'(3')-deoxyribonucleotidase, cytosolic type	8,92E-05	9,78E-05	0,000115	0,000127	0,000107252	1,68809E-05	15,739	9,72E-05	9,44E-05	0,000101	9,62E-05	9,62E-05	9,7047E-05	2,5384E-06	2,616
Q8BFZ9	ERLN2_MOUSE	Erlin-2	5,86E-05	6,22E-05	5,97E-05	6,08E-05	6,03366E-05	1,55137E-06	2,571	5,66E-05	5,78E-05	6,08E-05	5,52E-05	6,25E-05	5,85876E-05	3,02423E-06	5,162
O54865	GCYB1_MOUSE	Guanylate cyclase soluble subunit beta-1	0,000137	0,000166	0,000168	0,000118	0,00014712	2,42106E-05	16,456	0,00016	0,000108	0,000142	0,000141	0,00018	0,000146398	2,65776E-05	18,154
Q8BL97	SRSF7_MOUSE	Serine/arginine-rich splicing factor 7	4,89E-05	6,04E-05	6,32E-05	6,48E-05	5,93166E-05	7,16032E-06	12,071	5,53E-05	5,35E-05	5,74E-05	5,45E-05	6,18E-05	5,64872E-05	3,26634E-06	5,782
Q8BP67	RL24_MOUSE	60S ribosomal protein L24	5,19E-05	7,67E-05	8,04E-05	8,28E-05	7,29635E-05	1,42803E-05	19,572	7,86E-05	7,82E-05	9,78E-05	9,39E-05	9,63E-05	8,89721E-05	9,74326E-06	10,951
Q9DCH4	EIF3F_MOUSE	Eukaryotic translation initiation factor 3 subunit F	3,84E-05	4,09E-05	4,46E-05	4,14E-05	4,13131E-05	2,54888E-06	6,170	4,14E-05	3,48E-05	3,81E-05	3,91E-05	4,15E-05	3,90026E-05	2,75099E-06	7,053
P97765	WBP2_MOUSE	WW domain-binding protein 2	2,5E-05	2,72E-05	2,87E-05	3,08E-05	2,79373E-05	2,441E-06	8,737	3,24E-05	2,82E-05	2,85E-05	2,58E-05	2,36E-05	2,76911E-05	3,29133E-06	11,886
Q99KF1	TMED9_MOUSE	Transmembrane emp24 domain-containing protein 9	2,14E-05	2,67E-05	2,26E-05	2,49E-05	2,38695E-05	2,35077E-06	9,848	2,04E-05	2,07E-05	2,74E-05	2,82E-05	2,41E-05	2,41701E-05	3,63488E-06	15,039
P68037	UB2L3_MOUSE	Ubiquitin-conjugating enzyme E2 L3	7,16E-05	8,21E-05	7,27E-05	7,62E-05	7,56564E-05	4,70712E-06	6,222	7,34E-05	6,8E-05	7,23E-05	7,4E-05	6,33E-05	7,02265E-05	4,512E-06	6,425
Q60996	2A5G_MOUSE	Serine/threonine-protein phosphatase 2A 56 kDa regulatory subunit gamma isoform	2,4E-05	2,48E-05	2,51E-05	2,77E-05	2,54129E-05	1,60776E-06	6,327	2,9E-05	2,92E-05	2,89E-05	2,79E-05	2,59E-05	2,81824E-05	1,37006E-06	4,861
Q9D8B4	NDUAB_MOUSE	NADH dehydrogenase [ubiquinone] 1 alpha subcomplex subunit 11	7,46E-05	7,46E-05	7,3E-05	6,26E-05	7,11863E-05	5,75242E-06	8,081	5,53E-05	4,96E-05	4,86E-05	5,39E-05	5,06E-05	5,1586E-05	2,30851E-06	4,475
P40237	CD82_MOUSE	CD82 antigen	0,000134	0,000122	0,000106	0,000115	0,000119387	1,19905E-05	10,043	0,000121	0,00014	0,000136	0,000137	0,000122	0,000131279	8,80386E-06	6,706
Q8R5C5	ACTY_MOUSE	Beta-centractin	0,00022	0,000236	0,000223	0,000199	0,00021967	1,52072E-05	6,923	0,000237	0,000224	0,000212	0,000221	0,00023	0,000224752	9,28629E-06	4,132
O35609	SCAM3_MOUSE	Secretory carrier-associated membrane protein 3	3,71E-05	4,8E-05	4,16E-05	4,08E-05	4,18723E-05	4,56272E-06	10,897	3,98E-05	3,93E-05	3,99E-05	3,63E-05	4,37E-05	3,98067E-05	2,63527E-06	6,620
P97447	FHL1_MOUSE	Four and a half LIM domains protein 1	3,01E-05	2,72E-05	3,22E-05	3,63E-05	3,1467E-05	3,83774E-06	12,196	2,69E-05	3,06E-05	3,04E-05	2,95E-05	2,85E-05	2,91681E-05	1,51284E-06	5,187
Q9CQZ6	NDUB3_MOUSE	NADH dehydrogenase [ubiquinone] 1 beta subcomplex subunit 3	7,77E-05	8,04E-05	8,21E-05	8,26E-05	8,07063E-05	2,22731E-06	2,760	6,12E-05	6,91E-05	7,57E-05	6,49E-05	5,92E-05	6,60132E-05	6,60382E-06	10,004
Q8VEJ9	VPS4A_MOUSE	Vacuolar protein sorting-associated protein 4A	4,68E-05	4,73E-05	4,84E-05	4,5E-05	4,6861E-05	1,44856E-06	3,091	4,72E-05	4,24E-05	4,5E-05	4,98E-05	4E-05	4,48866E-05	3,83114E-06	8,535
Q9CQW2	ARL8B_MOUSE	ADP-ribosylation factor-like protein 8B	7,42E-05	8,43E-05	6,47E-05	8,08E-05	7,60105E-05	8,61242E-06	11,331	6,25E-05	5,27E-05	8,16E-05	7,21E-05	6,58E-05	6,69521E-05	1,07665E-05	16,081
Q9CPW0	CNTP2_MOUSE	Contactin-associated protein-like 2	5,68E-05	6,47E-05	5,63E-05	5,37E-05	5,78919E-05	4,75532E-06	8,214	5,44E-05	5,47E-05	5,31E-05	5,31E-05	5,67E-05	5,44048E-05	1,46421E-06	2,691
Q3TXS7	PSMD1_MOUSE	26S proteasome non-ATPase regulatory subunit 1	3,63E-05	9,57E-05	9,13E-05	5,24E-05	6,89135E-05	2,92055E-05	42,380	9,34E-05	7,79E-05	9,41E-05	8,15E-05	8,14E-05	8,5659E-05	7,51427E-06	8,772
Q8BKC5	IPO5_MOUSE	Importin-5	5,98E-05	6,36E-05	6,45E-05	6,3E-05	6,27478E-05	2,04611E-06	3,261	5,84E-05	5,85E-05	6,02E-05	6,02E-05	6,66E-05	6,0781E-05	3,39313E-06	5,583
Q9JLV5	CUL3_MOUSE	Cullin-3	6,23E-05	6,15E-05	6,48E-05	6,53E-05	6,3482E-05	1,85631E-06	2,924	6,12E-05	6,32E-05	6,63E-05	5,51E-05	6,55E-05	6,22726E-05	4,47288E-06	7,183
Q9Z1Z0	USO1_MOUSE	General vesicular transport factor p115	4,18E-05	5,53E-05	4,87E-05	4,82E-05	4,84886E-05	5,51485E-06	11,373	5,4E-05	5,11E-05	4,45E-05	5,45E-05	5,08E-05	5,09676E-05	3,96549E-06	7,780
Q9D0E1	HNRPM_MOUSE	Heterogeneous nuclear ribonucleoprotein M	0,000141	0,000165	0,000166	0,000147	0,000154797	1,28213E-05	8,283	0,000149	0,000148	0,000141	0,000161	0,000166	0,000153218	1,03335E-05	6,744
Q8C167	PPCEL_MOUSE	Prolyl endopeptidase-like	8,73E-05	8,82E-05	6,67E-05	7,12E-05	7,83551E-05	1,10132E-05	14,055	7,99E-05	6,96E-05	7,16E-05	8,55E-05	7,14E-05	7,56031E-05	6,81408E-06	9,013
Q8CA95	PDE10_MOUSE	cAMP and cAMP-inhibited cGMP 3',5'-cyclic phosphodiesterase 10A	0,000193	0,00014	0,000139	9,06E-05	0,000140588	4,17918E-05	29,726	0,000157	0,00016	0,000139	0,000203	0,000204	0,000172617	2,931E-05	16,980

P13707	GPDA_MOUSE	Glycerol-3-phosphate dehydrogenase [NAD(+)], cytoplasmic	0,000166	0,000156	0,000121	0,000165	0,000152039	2,107E-05	13,858	0,000167	0,000126	0,000179	0,000187	0,000144	0,000160627	2,53028E-05	15,753
Q99JI4	PSMD6_MOUSE	26S proteasome non-ATPase regulatory subunit 6	5,47E-05	6,61E-05	6,87E-05	6,34E-05	6,32384E-05	6,05534E-06	9,575	6,1E-05	5,73E-05	5,71E-05	5,43E-05	6,03E-05	5,80114E-05	2,71459E-06	4,679
Q99LF4	RTCB_MOUSE	tRNA-splicing ligase RtcB homolog	4,87E-05	4,95E-05	4,96E-05	5,31E-05	5,02317E-05	1,95424E-06	3,890	4,9E-05	4,81E-05	5,28E-05	5,46E-05	5,42E-05	5,17403E-05	3,02129E-06	5,839
Q80XI4	PI42B_MOUSE	Phosphatidylinositol 5-phosphate 4-kinase type-2 beta	9,75E-05	0,000115	0,000118	0,000112	0,0001106	9,08067E-06	8,210	0,00012	0,000102	0,000125	0,000133	0,000115	0,00011899	1,16698E-05	9,807
Q9D8W5	PSD12_MOUSE	26S proteasome non-ATPase regulatory subunit 12	4,4E-05	3,94E-05	5,17E-05	4,5E-05	4,5021E-05	5,10127E-06	11,331	4,43E-05	4,49E-05	5,08E-05	5,13E-05	4,94E-05	4,81566E-05	3,31142E-06	6,876
O55029	COPB2_MOUSE	Coatomer subunit beta'	2,94E-05	2,98E-05	2,64E-05	2,44E-05	2,75103E-05	2,55747E-06	9,296	2,24E-05	2,36E-05	2,33E-05	2,23E-05	2,22E-05	2,27651E-05	6,38288E-07	2,804
Q9QYB1	CLIC4_MOUSE	Chloride intracellular channel protein 4	0,000164	0,000141	0,000125	0,00014	0,0001425	1,59181E-05	11,171	0,000142	0,000157	0,000142	0,000133	0,000105	0,000135668	1,93683E-05	14,276
P51660	DHB4_MOUSE	Peroxisomal multifunctional enzyme type 2	9,51E-05	9,45E-05	9,42E-05	0,000103	9,67855E-05	4,38161E-06	4,527	9,48E-05	9,77E-05	0,000103	9,14E-05	8,84E-05	9,49807E-05	5,53931E-06	5,832
Q8R574	KPRB_MOUSE	Phosphoribosyl pyrophosphate synthase-associated protein 2	6,47E-05	7,46E-05	7,06E-05	7,78E-05	7,19362E-05	5,64114E-06	7,842	5,98E-05	5,67E-05	5,5E-05	6,38E-05	6,62E-05	6,03086E-05	4,68976E-06	7,776
P50580	PA2G4_MOUSE	Proliferation-associated protein 2G4	5,78E-05	6,17E-05	6,95E-05	6,63E-05	6,38102E-05	5,15647E-06	8,081	6,22E-05	6,17E-05	6,9E-05	7,06E-05	6E-05	6,47131E-05	4,76112E-06	7,357
Q8R570	SNP47_MOUSE	Synaptosomal-associated protein 47	9,61E-05	0,000105	8,74E-05	9,12E-05	9,49874E-05	7,66045E-06	8,065	0,000107	9,29E-05	0,0001	9,44E-05	9,87E-05	9,8754E-05	5,67951E-06	5,751
O08547	SC22B_MOUSE	Vesicle-trafficking protein SEC22b	0,000106	0,000109	0,000109	0,000111	0,000108727	2,04883E-06	1,884	0,000115	0,000117	0,000152	0,00014	0,000128	0,000130329	1,55368E-05	11,921
Q91YR1	TWF1_MOUSE	Twinfilin-1	2,14E-05	2,33E-05	2,01E-05	2,47E-05	2,23684E-05	2,04074E-06	9,123	2,39E-05	2,09E-05	2,06E-05	1,74E-05	1,83E-05	2,02151E-05	2,51679E-06	12,450
Q99LD4	CSN1_MOUSE	COP9 signalosome complex subunit 1	7,36E-05	7,65E-05	7,28E-05	7,25E-05	7,38527E-05	1,84493E-06	2,498	8,78E-05	7,99E-05	7,48E-05	8,3E-05	8,67E-05	8,24125E-05	5,29531E-06	6,425
Q8BWR2	PITH1_MOUSE	PITH domain-containing protein 1	5,87E-05	6,64E-05	5,78E-05	7,75E-05	6,51034E-05	9,09015E-06	13,963	5,2E-05	5,82E-05	6,64E-05	5,21E-05	4,78E-05	5,5322E-05	7,24235E-06	13,091
Q99L13	3HIDH_MOUSE	3-hydroxyisobutyrate dehydrogenase, mitochondrial	0,000237	0,000232	0,000201	0,000214	0,000221031	1,67466E-05	7,577	0,000229	0,000209	0,000234	0,000209	0,000197	0,00021548	1,56548E-05	7,265
P32067	LA_MOUSE	Lupus La protein homolog	4,82E-05	5,45E-05	6,19E-05	5,42E-05	5,47155E-05	5,58404E-06	10,206	4,22E-05	3,57E-05	5E-05	5,04E-05	4,53E-05	4,47145E-05	6,08981E-06	13,619
Q7M6Y3	PICAL_MOUSE	Phosphatidylinositol-binding clathrin assembly protein	2,6E-05	4,23E-06	2,66E-05	2,67E-05	2,08746E-05	1,11015E-05	53,182	2,49E-05	2,57E-05	2,79E-05	2,63E-05	2,18E-05	2,53051E-05	2,26483E-06	8,950
Q9WVE8	PACN2_MOUSE	Protein kinase C and casein kinase substrate in neurons protein 2	3,43E-05	4,67E-05	4,07E-05	3,92E-05	4,02307E-05	5,11804E-06	12,722	4,6E-05	3,7E-05	5,42E-05	5,55E-05	3,98E-05	4,65061E-05	8,31199E-06	17,873
O35668	HAP1_MOUSE	Huntingtin-associated protein 1	2,48E-05	7,22E-05	5,81E-05	6,95E-05	5,61286E-05	2,17714E-05	38,788	5,08E-05	5,91E-05	7,69E-05	7,25E-05	6,91E-05	6,56729E-05	1,06017E-05	16,143
Q8C163	EXOG_MOUSE	Nuclease EXOG, mitochondrial	2,6E-05	2,48E-05	2,76E-05	2,92E-05	2,69135E-05	1,92902E-06	7,167	2,76E-05	2,68E-05	2,79E-05	3,46E-05	2,56E-05	2,8484E-05	3,5192E-06	12,355
P62192	PRS4_MOUSE	26S protease regulatory subunit 4	9,21E-05	0,000119	0,000117	0,0001	0,000107067	1,31677E-05	12,299	0,000111	0,000102	0,000118	8,93E-05	0,000113	0,000106634	1,13818E-05	10,674
Q9DCM0	ETHE1_MOUSE	Persulfide dioxygenase ETHE1, mitochondrial	1,41E-05	1,04E-06	1,54E-05	1,36E-05	1,10342E-05	6,70455E-06	60,761	1,3E-05	1,46E-05	1,59E-05	1,28E-05	1,23E-05	1,37219E-05	1,51901E-06	11,070
Q9CX86	ROA0_MOUSE	Heterogeneous nuclear ribonucleoprotein A0	7,1E-05	0,000131	0,000132	0,000136	0,000117439	3,10031E-05	26,399	7,2E-05	0,000117	0,000125	0,000114	0,000113	0,000108145	2,07363E-05	19,175
P70670	NACAM_MOUSE	Nascent polypeptide-associated complex subunit alpha, muscle-specific form	7,79E-05	7,98E-05	8,03E-05	7,84E-05	7,9084E-05	1,13206E-06	1,431	7,74E-05	7,34E-05	7,49E-05	6,93E-05	7,32E-05	7,36439E-05	2,9617E-06	4,022
Q3U1J4	DDB1_MOUSE	DNA damage-binding protein 1	6,52E-05	7,67E-05	7E-05	7,08E-05	7,06658E-05	4,69346E-06	6,642	6,5E-05	6,23E-05	6,13E-05	6,07E-05	6,85E-05	6,35524E-05	3,24354E-06	5,104
Q9JME5	AP3B2_MOUSE	AP-3 complex subunit beta-2	8,63E-05	0,000111	0,000104	0,000106	0,000101719	1,06402E-05	10,460	0,00012	0,000107	9,94E-05	0,000103	0,000109	0,000107535	7,76155E-06	7,218
P33173	KIF1A_MOUSE	Kinesin-like protein KIF1A	6,64E-05	8,45E-05	8,22E-05	7,59E-05	7,72089E-05	8,0921E-06	10,481	8,44E-05	7,52E-05	7,34E-05	6,82E-05	6,62E-05	7,34857E-05	7,10477E-06	9,668
Q91ZA3	PCCA_MOUSE	Propionyl-CoA carboxylase alpha chain, mitochondrial	0,000135	0,000163	0,000136	0,000134	0,000142016	1,38048E-05	9,721	0,000128	0,000132	0,000123	0,00011	0,000137	0,000126132	1,02073E-05	8,093
Q6ZPE2	MTMR5_MOUSE	Myotubularin-related protein 5	8,75E-05	5,56E-05	5,61E-05	6,2E-05	6,52828E-05	1,50742E-05	23,091	6,54E-05	3,22E-05	6,01E-05	6,37E-05	5,76E-05	5,58311E-05	1,35415E-05	24,254
Q9EPL8	IPO7_MOUSE	Importin-7	3,45E-05	4,06E-05	4,84E-05	3,99E-05	4,08552E-05	5,73547E-06	14,039	3,63E-05	3,63E-05	3,65E-05	3,72E-05	4,04E-05	3,73183E-05	1,78456E-06	4,782
Q91ZJ5	UGPA_MOUSE	UTP--glucose-1-phosphate uridylyltransferase	0,000176	0,000194	0,000197	0,000186	0,00018824	9,46048E-06	5,026	0,000185	0,000164	0,000183	0,000182	0,000168	0,000176646	9,8066E-06	5,552
Q91XM9	DLG2_MOUSE	Disks large homolog 2	0,000175	0,000154	0,000164	0,000155	0,000162088	9,54718E-06	5,890	0,000144	0,000145	0,000143	0,000145	0,000174	0,000149977	1,33311E-05	8,889
Q8VD37	SGIP1_MOUSE	SH3-containing GRB2-like protein 3-interacting protein 1	0,000176	0,000189	0,000178	0,000188	0,000182732	6,85494E-06	3,751	0,000204	0,000167	0,000165	0,000175	0,000198	0,000181867	1,77847E-05	9,779
Q91VR5	DDX1_MOUSE	ATP-dependent RNA helicase DDX1	0,00022	0,000243	0,000239	0,000243	0,000236249	1,11241E-05	4,709	0,000243	0,00023	0,000236	0,000226	0,000249	0,000236697	9,52046E-06	4,022
Q60737	CSK21_MOUSE	Casein kinase II subunit alpha	4,07E-05	3,79E-05	3,98E-05	4,23E-05	4,01744E-05	1,85396E-06	4,615	4,13E-05	3,87E-05	3,8E-05	3,48E-05	4,38E-05	3,93217E-05	3,40052E-06	8,648
P12787	COX5A_MOUSE	Cytochrome c oxidase subunit 5A, mitochondrial	0,000121	0,000187	0,000169	0,000172	0,000162068	2,83665E-05	17,503	0,000118	0,000163	0,000176	0,000171	0,000169	0,000159392	2,33896E-05	14,674
P84075	HPCA_MOUSE	Neuron-specific calcium-binding protein hippocalcin	0,000121	0,000128	0,000127	0,000159	0,00013383	1,73476E-05	12,962	8,64E-05	8,66E-05	0,000109	0,000116	0,000115	0,000102379	1,4755E-05	14,412
Q9CYH2	F213A_MOUSE	Redox-regulatory protein FAM213A	0,000348	0,000358	0,000337	0,00034	0,000345786	9,425E-06	2,726	0,000297	0,000361	0,00032	0,000317	0,000306	0,000320327	2,46079E-05	7,682
Q9WTP7	KAD3_MOUSE	GTP:AMP phosphotransferase AK3, mitochondrial	0,000146	0,000135	0,000145	0,000143	0,000142217	4,97167E-06	3,496	0,000139	0,000149	0,00013	0,0001	8,33E-05	0,000120207	2,75446E-05	22,914
Q9JKK7	TMOD2_MOUSE	Tropomodulin-2	7,28E-05	7,29E-05	7,18E-05	7,13E-05	7,22096E-05	7,69238E-07	1,065	7,04E-05	6,74E-05	7,03E-05	7,16E-05	6,61E-05	6,91463E-05	2,3087E-06	3,339
O35954	PITM1_MOUSE	Membrane-associated phosphatidylinositol transfer protein 1	0,00016	0,000172	0,000172	0,00016	0,000165861	7,14211E-06	4,306	0,000187	0,000169	0,000166	0,000172	0,000173	0,000173231	7,99093E-06	4,613
Q8K406	LGI3_MOUSE	Leucine-rich repeat LGI family member 3	6,91E-05	8,78E-05	5,99E-05	6,51E-05	7,04643E-05	1,21194E-05	17,199	8,42E-05	8,05E-05	7,43E-05	7,57E-05	6,88E-05	7,67072E-05	5,92501E-06	7,724
Q8VCW8	ACSF2_MOUSE	Acyl-CoA synthetase family member 2, mitochondrial	6,14E-05	8,31E-05	6,77E-05	6,72E-05	6,9848E-05	9,28477E-06	13,293	6,02E-05	7,61E-05	7,31E-05	5,59E-05	7,61E-05	6,8277E-05	9,55499E-06	13,994
Q9Z0P4	PALM_MOUSE	Paralemmin-1	0,000315	0,000388	0,000411	0,000384	0,000374428	4,161E-05	11,113	0,000399	0,000344	0,00037	0,000386	0,000397	0,00037917	2,26314E-05	5,969
Q8R464	CADM4_MOUSE	Cell adhesion molecule 4	0,000256	0,00025	0,000223	0,000264	0,000248055	1,78102E-05	7,180	0,000241	0,000272	0,000295	0,000273	0,000253	0,000266764	2,08427E-05	7,813
O35295	PURB_MOUSE	Transcriptional activator protein Pur-beta	0,000103	0,00012	0,000123	0,000141	0,00012171	1,52207E-05	12,506	0,000115	0,000107	0,000118	0,000121	9,77E-05	0,000111649	9,3839E-06	8,405
P01942	HBA_MOUSE	Hemoglobin subunit alpha	0,000215	9,66E-05	0,000146	0,00017	0,000157004	4,93565E-05	31,436	0,000103	0,000103	8,59E-05	5,76E-05	7,18E-05	8,41442E-05	1,96951E-05	23,406
Q62443	NPTX1_MOUSE	Neuronal pentraxin-1	4,5E-05	4,21E-05	4,78E-05	5,11E-05	4,65127E-05	3,85058E-06	8,279	3,9E-05	3,18E-05	4,62E-05	3,62E-05	3,6E-05	3,78635E-05	5,30786E-06	14,018
Q9R0P3	ESTD_MOUSE	S-formylglutathione hydrolase	0,000147	0,000137	0,000122	0,000137	0,000135462	1,04531E-05	7,717	0,000136	0,000154	0,000133	0,000113	0,000124	0,00013183	1,49017E-05	11,304
O88741	GDAP1_MOUSE	Ganglioside-induced differentiation-associated protein 1	0,000133	0,000121	9,51E-05	0,000132	0,000120264	1,75789E-05	14,617	0,000135	0,000132	0,000121	0,000128	0,000122	0,000127758	5,93163E-06	4,643
P51859	HDGF_MOUSE	Hepatoma-derived growth factor	8,74E-05	8,57E-05	8,79E-05	8,89E-05	8,74906E-05	1,36048E-06	1,555	7,39E-05	5,14E-05	8,24E-05	8,48E-05	7,7E-05	7,38946E-05	1,32857E-05	17,979
Q8R5M8	CADM1_MOUSE	Cell adhesion molecule 1	0,000134	0,000145	0,000176	0,000151	0,000151455	1,75594E-05	11,594	0,000134	0,00012	0,000132	0,000129	0,000177	0,000138516	2,23826E-05	16,159
Q68FF6	GIT1_MOUSE	ARF GTPase-activating protein GIT1	2,04E-05	2,62E-05	2,11E-05	2,79E-05	2,38839E-05	3,70314E-06	15,505	2,93E-05	2,35E-05	2,5E-05	2,78E-05	3,13E-05	2,73658E-05	3,17181E-06	11,590
Q6PHN9	RAB35_MOUSE	Ras-related protein Rab-35	6,24E-05	6,34E-05	5,94E-05	7,2E-05	6,42588E-05	5,40517E-06	8,412	6,72E-05	8,28E-05	8,32E-05	7,86E-05	7,15E-05	7,66656E-05	7,06738E-06	9,218
Q921F2	TADBP_MOUSE	TAR DNA-binding protein 43	0,000113	9,16E-05	8,59E-05	0,000107	9,91986E-05	1,25176E-05	12,619	0,000114	0,000116	0,000106	0,000101	0,000104	0,00010831	6,58142E-06	6,076
Q791T5	MTCH1_MOUSE	Mitochondrial carrier homolog 1	8,48E-05	8,17E-05	8,51E-05	7,43E-05	8,14605E-05	5,03158E-06	6,177	5,78E-05	5,88E-05	6,91E-05	5,9E-05	6,19E-05	6,13408E-05	4,59782E-06	7,496
Q9D1E6	TBCB_MOUSE	Tubulin-folding cofactor B	3,33E-05	3,63E-05	3,6E-05	4,15E-05	3,67882E-05	3,44611E-06	9,367	3,22E-05	2,25E-05	3,49E-05	3,69E-05	3,89E-05	3,30837E-05	6,38894E-06	19,311
Q9CQ54	NDUC2_MOUSE	NADH dehydrogenase [ubiquinone] 1 subunit C2	6E-05	6,77E-05	7,88E-05	6,86E-05	6,87728E-05	7,7301E-06	11,240	5,42E-05	5,86E-05	6,41E-05	5,68E-05	6,39E-05	5,95152E-05	4,37879E-06	7,357
P29391	FRIL1_MOUSE	Ferritin light chain 1	3,25E-05	3,08E-05	3,04E-05	2,8E-05	3,04234E-05	1,87451E-06	6,161	2,37E-05	2,85E-05	3,34E-05	2,91E-05	3,43E-05	2,98185E-05	4,2498E-06	14,252
P21460	CYTC_MOUSE	Cystatin-C	9,1E-05	8,04E-05	0,000113	0,000102	9,66923E-05	1,41436E-05	14,627	9,72E-05	0,000124	0,000106	9,58E-05	9,56E-05	0,000103605	1,20346E-05	11,616
B9EJ86	OSBL8_MOUSE	Oxysterol-binding protein-related protein 8	3,38E-05	3,07E-05	3,6E-05	2,81E-05	3,21244E-05	3,47466E-06	10,816	3,33E-05	3,18E-05	2,82E-05	3,07E-05	4,23E-05	3,32808E-05	5,38176E-06	16,171
P63325	RS10_MOUSE	40S ribosomal protein S10	0,000179	0,000164	0,000184	0,000227	0,000188337	2,67613E-05	14,209	0,000163	0,000101	0,00019	0,000207	0,000175	0,000167052	4,02981E-05	24,123
Q9WUC3	LY6H_MOUSE	Lymphocyte antigen 6H	4,54E-05	4,72E-05	7,28E-05	7,25E-05	5,94746E-05	1,52221E-05	25,594	4,29E-05	8,8E-05	7,56E-05	6,23E-05	6,25E-05	6,62413E-05	1,68439E-05	25,428
Q9WTP6	KAD2_MOUSE	Adenylate kinase 2, mitochondrial	7E-05	6,95E-05	6,99E-05	7,01E-05	6,98836E-05	2,83086E-07	0,405	6,4E-05	7,1E-05	3,92E-05	7,15E-05	6,47E-05	6,20881E-05	1,32424E-05	21,328
Q61187	TS101_MOUSE	Tumor susceptibility gene 101 protein	2,56E-05	3,01E-05	2,4E-05	2,38E-05	2,58515E-05	2,95952E-06	11,448	2,46E-05	2,39E-05	2,36E-05	2,64E-05	2,6E-05	2,4898E-05	1,25181E-06	5,028
P47915	RL29_MOUSE	60S ribosomal protein L29	9,65E-06	1,03E-05	8,19E-06	1,07E-05	9,71933E-06	1,11119E-06	11,433	1,11E-05	1,12E-05	1,07E-05	9,81E-06	8,57E-06	1,02603E-05	1,08777E-06	10,602
Q9CPQ3	TOM22_MOUSE	Mitochondrial import receptor subunit TOM22 homolog	1,02E-05	9,22E-06	1,09E-05	9,71E-06	1,00083E-05	7,08993E-07	7,084	1,2E-05	1,1E-05	1,85E-05	1,38E-05	1,26E-05	1,35889E-05	2,92199E-06	21,503
O88531	PPT1_MOUSE	Palmitoyl-protein thioesterase 1	6,02E-05	5,53E-05	6,49E-05	5,61E-05	5,91442E-05	4,37664E-06	7,400	6,36E-05	6,49E-05	6,27E-05	5,86E-05	6,47E-05	6,2911E-05	2,57484E-06	4,093
Q99NB9	SF3B1_MOUSE	Splicing factor 3B subunit 1	2,44E-05	3,36E-05	3,23E-05	2,89E-05	2,98122E-05	4,13333E-06	13,865	3,65E-05	2,49E-05	2,58E-05	3,17E-05	3,53E-05	3,08422E-05	5,34601E-06	17,333
Q9DBR7	MYPT1_MOUSE	Protein phosphatase 1 regulatory subunit 12A	2,1E-05	3,11E-05	2,76E-05	2,86E-05	2,70859E-05	4,30521E-06	15,895	2,61E-05	2,47E-05	2,53E-05	2,53E-05	2,94E-05	2,61897E-05	1,88207E-06	7,186

G5E8K5	ANK3_MOUSE	Ankyrin-3	0,000113	0,000105	0,0001	0,000101	0,000104979	6,014E-06	5,729	0,000114	0,000107	0,000106	0,000101	0,000111	0,000107861	4,76103E-06	4,414
Q810U4	NRCAM_MOUSE	Neuronal cell adhesion molecule	0,000223	0,000246	0,000256	0,000188	0,00022832	2,99436E-05	13,115	0,000198	0,000242	0,000238	0,000224	0,000249	0,000230264	2,03545E-05	8,840
Q9D394	RUFY3_MOUSE	Protein RUFY3	0,000175	0,000175	0,000164	0,000157	0,000167671	8,88159E-06	5,297	0,000182	0,000171	0,000177	0,000172	0,000163	0,000172968	7,0002E-06	4,047
Q9CZD3	SYG_MOUSE	Glycine--tRNA ligase	0,000139	0,000168	0,00015	0,00014	0,000149348	1,33807E-05	8,959	0,000148	0,000149	0,000146	0,00015	0,000165	0,000151632	7,57404E-06	4,995
Q9ESN6	TRIM2_MOUSE	Tripartite motif-containing protein 2	9,52E-05	0,000114	0,000104	0,000107	0,000104967	7,92332E-06	7,548	0,00012	0,000109	0,000105	0,000114	0,000112	0,000111962	5,44033E-06	4,859
Q9CQN1	TRAP1_MOUSE	Heat shock protein 75 kDa, mitochondrial	3,75E-05	3,48E-05	3,25E-05	4,12E-05	3,65023E-05	3,73582E-06	10,234	3,63E-05	4,06E-05	3,52E-05	3,63E-05	3,82E-05	3,73211E-05	2,11612E-06	5,670
P49025	CTRO_MOUSE	Citron Rho-interacting kinase	1,97E-05	2,26E-05	2,1E-05	2,34E-05	2,16895E-05	1,64207E-06	7,571	2,63E-05	2,34E-05	2,13E-05	2,39E-05	2,38E-05	2,37531E-05	1,76156E-06	7,416
P35585	AP1M1_MOUSE	AP-1 complex subunit mu-1	0,000171	0,000184	0,00017	0,000174	0,000174513	6,53391E-06	3,744	0,000179	0,000158	0,00018	0,000165	0,000188	0,000174019	1,22238E-05	7,024
P21107	TPM3_MOUSE	Tropomyosin alpha-3 chain	0,000242	0,000254	0,000251	0,000239	0,000246438	7,11753E-06	2,888	0,000247	0,000185	0,000251	0,000238	0,000237	0,000231416	2,67076E-05	11,541
Q60598	SRC8_MOUSE	Src substrate cortactin	0,000184	0,00019	0,000193	0,000201	0,000191865	7,18263E-06	3,744	0,000165	0,000138	0,000154	0,000138	0,000184	0,000155823	1,93398E-05	12,411
Q8BKX1	BAIP2_MOUSE	Brain-specific angiogenesis inhibitor 1-associated protein 2	0,000146	0,000151	0,000207	0,000157	0,000165142	2,85313E-05	17,277	0,000166	0,000146	0,000171	0,000193	0,000214	0,000177868	2,59699E-05	14,601
Q8C419	GP158_MOUSE	Probable G-protein coupled receptor 158	2,53E-05	2,37E-05	2,64E-05	2,35E-05	2,4717E-05	1,39009E-06	5,624	2,22E-05	2,71E-05	2,45E-05	2,55E-05	2,55E-05	2,49679E-05	1,81327E-06	7,262
Q8VBW6	ULA1_MOUSE	NEDD8-activating enzyme E1 regulatory subunit	2,58E-05	2,81E-05	2,93E-05	2,88E-05	2,79989E-05	1,52499E-06	5,447	3,38E-05	2,5E-05	2,66E-05	2,31E-05	2,83E-05	2,73627E-05	4,09477E-06	14,965
P49615	CDK5_MOUSE	Cyclin-dependent-like kinase 5	0,00022	0,000197	0,00022	0,000236	0,000218334	1,61486E-05	7,396	0,00019	0,000204	0,000203	0,000172	0,000164	0,000186475	1,82984E-05	9,813
Q9Z1Z2	STRAP_MOUSE	Serine-threonine kinase receptor-associated protein	7,96E-05	7,18E-05	8,63E-05	7,78E-05	7,8864E-05	5,9631E-06	7,561	7,1E-05	6,3E-05	7,07E-05	6,77E-05	5,7E-05	6,58583E-05	5,92926E-06	9,003
O70325	GPX41_MOUSE	Phospholipid hydroperoxide glutathione peroxidase, mitochondrial	6,77E-05	6,4E-05	5,74E-05	6,43E-05	6,33467E-05	4,30673E-06	6,799	5,83E-05	6,21E-05	7,11E-05	5,89E-05	4,58E-05	5,9234E-05	9,09209E-06	15,349
Q61035	SYHC_MOUSE	Histidine--tRNA ligase, cytoplasmic	2,45E-05	2,61E-05	2,73E-05	2,73E-05	2,62759E-05	1,3373E-06	5,089	2,39E-05	2,34E-05	2,65E-05	2,37E-05	2,08E-05	2,36547E-05	2,01082E-06	8,501
O35864	CSN5_MOUSE	COP9 signalosome complex subunit 5	7,16E-05	6,75E-05	6,82E-05	7,37E-05	7,02417E-05	2,89916E-06	4,127	6,43E-05	6,27E-05	6,01E-05	5,27E-05	4,92E-05	5,77979E-05	6,57101E-06	11,369
P28867	KPCD_MOUSE	Protein kinase C delta type	0,000184	0,000132	9,88E-05	8,99E-05	0,000126036	4,23883E-05	33,632	0,000211	0,000157	0,000145	0,000152	0,000159	0,000164909	2,6514E-05	16,078
Q9JLM8	DCLK1_MOUSE	Serine/threonine-protein kinase DCLK1	7,54E-05	7,95E-05	9,87E-05	7,36E-05	8,17801E-05	1,15281E-05	14,097	0,000106	7,39E-05	8,11E-05	8,34E-05	8,46E-05	8,58792E-05	1,22168E-05	14,226
Q60625	ICAM5_MOUSE	Intercellular adhesion molecule 5	2,79E-05	4,9E-05	0,0001	7,18E-05	6,2218E-05	3,10486E-05	49,903	4,26E-05	2,68E-05	3,69E-05	4,07E-05	6,62E-05	4,26529E-05	1,45297E-05	34,065
Q7TMF3	NDUAC_MOUSE	NADH dehydrogenase [ubiquinone] 1 alpha subcomplex subunit 12	6,29E-05	6,47E-05	7,33E-05	7,07E-05	6,79195E-05	4,91437E-06	7,236	6,39E-05	6,91E-05	8,15E-05	7,62E-05	7,02E-05	7,21814E-05	6,78722E-06	9,403
P17156	HSP72_MOUSE	Heat shock-related 70 kDa protein 2	9,25E-05	0,000126	0,000138	0,000124	0,00012012	1,93539E-05	16,112	0,000101	0,000105	0,000107	0,000101	0,000115	0,000105741	5,85391E-06	5,536
Q61937	NPM_MOUSE	Nucleophosmin	6,22E-05	6,5E-05	6,5E-05	7,58E-05	6,70094E-05	6,0283E-06	8,996	6,26E-05	5,99E-05	6,85E-05	6,19E-05	5,28E-05	6,11479E-05	5,66816E-06	9,270
Q3UUI3	THEM4_MOUSE	Acyl-coenzyme A thioesterase THEM4	2,67E-05	2,35E-05	4E-07	3,37E-05	2,10823E-05	1,44249E-05	68,422	3,09E-05	3,2E-05	3,79E-05	4,29E-05	3,75E-05	3,62227E-05	4,89106E-06	13,503
P26516	PSMD7_MOUSE	26S proteasome non-ATPase regulatory subunit 7	4,76E-05	5,54E-05	5,46E-05	4,9E-05	5,16425E-05	3,90129E-06	7,554	5,32E-05	4,44E-05	5,33E-05	5,05E-05	5,33E-05	5,0953E-05	3,84775E-06	7,552
Q91WJ8	FUBP1_MOUSE	Far upstream element-binding protein 1	3,81E-05	4,5E-05	5,11E-05	5,58E-05	4,74922E-05	7,67634E-06	16,163	4,75E-05	3,75E-05	4,73E-05	4,53E-05	5,31E-05	4,61493E-05	5,62001E-06	12,178
Q6WVG3	KCD12_MOUSE	BTB/POZ domain-containing protein KCTD12	9,85E-05	0,000103	0,000107	0,000113	0,000105314	6,20689E-06	5,894	9,58E-05	8,7E-05	9,99E-05	9,76E-05	0,000116	9,92444E-05	1,05776E-05	10,658
Q8BGZ1	HPCL4_MOUSE	Hippocalcin-like protein 4	7,81E-06	9,12E-06	1,1E-05	1,11E-05	9,76261E-06	1,5882E-06	16,268	1,06E-05	1,28E-05	1,56E-05	1,38E-05	1,31E-05	1,31716E-05	1,80249E-06	13,685
O55013	TPPC3_MOUSE	Trafficking protein particle complex subunit 3	2,1E-05	1,72E-05	1,85E-05	1,87E-05	1,8872E-05	1,59624E-06	8,458	1,59E-05	1,96E-05	2,2E-05	1,84E-05	1,9E-05	1,89564E-05	2,20802E-06	11,648
Q9DBE8	ALG2_MOUSE	Alpha-1,3/1,6-mannosyltransferase ALG2	4,85E-05	4,86E-05	3,82E-05	5,43E-05	4,7395E-05	6,70465E-06	14,146	4,54E-05	4,37E-05	4,44E-05	4,64E-05	3,49E-05	4,29607E-05	4,58931E-06	10,683
Q9EQZ6	RPGF4_MOUSE	Rap guanine nucleotide exchange factor 4	0	0	0	0	0	0	#DIV/0!	0	0	0	0	0	0	0	#DIV/0!
Q9R1P3	PSB2_MOUSE	Proteasome subunit beta type-2	0,000276	0,000232	0,000194	0,000318	0,000255015	5,37336E-05	21,071	0,000202	0,000146	0,000257	0,000248	0,000224	0,000215365	4,43615E-05	20,598
Q9Z0Y1	DCTN3_MOUSE	Dynactin subunit 3	9,99E-05	0,000104	9,61E-05	0,000108	0,000102221	5,34359E-06	5,227	9,74E-05	0,000105	0,000107	0,000103	8,4E-05	9,93532E-05	9,31158E-06	9,372
Q8K021	SCAM1_MOUSE	Secretory carrier-associated membrane protein 1	3,93E-05	4,37E-05	3,57E-05	3,98E-05	3,96324E-05	3,29122E-06	8,304	3,91E-05	3,49E-05	3,65E-05	3,54E-05	3,2E-05	3,55685E-05	2,59606E-06	7,299
P35803	GPM6B_MOUSE	Neuronal membrane glycoprotein M6-b	0,000175	0,000154	0,000181	0,000154	0,000165611	1,41517E-05	8,545	0,000163	0,000177	0,000177	0,00016	0,000204	0,000175953	1,7432E-05	9,907
P28658	ATX10_MOUSE	Ataxin-10	3,41E-05	4,31E-05	3,93E-05	5,03E-05	4,1684E-05	6,84027E-06	16,410	3,58E-05	4,03E-05	4,31E-05	3,78E-05	3,57E-05	3,85547E-05	3,16886E-06	8,219
Q61029	LAP2B_MOUSE	Lamina-associated polypeptide 2, isoforms beta/delta/epsilon/gamma	3,33E-05	3,69E-05	3,25E-05	3,43E-05	3,42463E-05	1,95465E-06	5,708	3,17E-05	2,77E-05	3,03E-05	3,48E-05	3,14E-05	3,11928E-05	2,56939E-06	8,237
Q9D6K5	SYJ2B_MOUSE	Synaptojanin-2-binding protein	2,08E-05	2,18E-05	2,44E-05	2,27E-05	2,24322E-05	1,55034E-06	6,911	2,29E-05	2,24E-05	2,52E-05	2,17E-05	1,86E-05	2,21552E-05	2,37051E-06	10,700
Q99L43	CDS2_MOUSE	Phosphatidate cytidylyltransferase 2	9,72E-05	0,000105	3,31E-05	8,68E-05	8,04413E-05	3,24054E-05	40,284	0,000105	0,0001	3,26E-05	7,6E-05	9,28E-05	8,12564E-05	2,93107E-05	36,072
Q0VBF8	STUM_MOUSE	Protein stum homolog	2,21E-05	2,83E-05	3,88E-05	3,46E-05	3,09428E-05	7,28582E-06	23,546	2,99E-05	2,43E-05	3,09E-05	2,69E-05	3,53E-05	2,94625E-05	4,18092E-06	14,191
Q8CAE9	PDXL2_MOUSE	#N/A	2,26E-05	2,47E-05	2,49E-05	2,35E-05	2,39291E-05	1,0981E-06	4,589	2,16E-05	1,88E-05	1,82E-05	2,23E-05	2,13E-05	2,04495E-05	1,85192E-06	9,056
Q8K310	MATR3_MOUSE	Matrin-3	4,64E-05	6,35E-05	5,7E-05	6,51E-05	5,79997E-05	8,48121E-06	14,623	7,24E-05	6,39E-05	6,56E-05	5,61E-05	6,56E-05	6,47272E-05	5,81429E-06	8,983
Q61330	CNTN2_MOUSE	Contactin-2	0,0001	9,74E-05	8,81E-05	6,52E-05	8,77051E-05	1,58805E-05	18,107	9,82E-05	9,36E-05	9,62E-05	9,23E-05	6,73E-05	8,95156E-05	1,26376E-05	14,118
Q9CR16	PPID_MOUSE	Peptidyl-prolyl cis-trans isomerase D	5,6E-05	5,72E-05	5,82E-05	6,65E-05	5,94802E-05	4,76412E-06	8,010	6,15E-05	5,84E-05	6,25E-05	6,06E-05	5,63E-05	5,98555E-05	2,50072E-06	4,178
Q8K212	PACS1_MOUSE	Phosphofurin acidic cluster sorting protein 1	3,36E-05	6,46E-05	6,74E-05	6,65E-05	5,80321E-05	1,63383E-05	28,154	6,52E-05	5,75E-05	6,61E-05	5,22E-05	6,87E-05	6,19481E-05	6,83524E-06	11,034
Q9JLN9	MTOR_MOUSE	Serine/threonine-protein kinase mTOR	1,9E-05	2,21E-05	2,71E-05	2,35E-05	2,29396E-05	3,36884E-06	14,686	1,99E-05	1,24E-05	1,76E-05	1,8E-05	2,07E-05	1,77404E-05	3,25287E-06	18,336
Q61490	CD166_MOUSE	CD166 antigen	0,000165	0,000195	0,000228	0,000177	0,000191076	2,72337E-05	14,253	0,000175	0,000143	0,000182	0,000193	0,000228	0,00018421	3,08135E-05	16,727
Q91VN4	MIC25_MOUSE	MICOS complex subunit Mic25	4,17E-05	3,88E-05	4,16E-05	3,96E-05	4,04159E-05	1,4334E-06	3,547	3,86E-05	2,9E-05	4,16E-05	4,14E-05	3,99E-05	3,8103E-05	5,20904E-06	13,671
Q5EBJ4	ERMIN_MOUSE	Ermin	7,68E-05	7,1E-05	5,89E-05	6,57E-05	6,8097E-05	7,60872E-06	11,173	7,92E-05	6,67E-05	6,77E-05	6,4E-05	6,33E-05	6,81766E-05	6,43553E-06	9,440
Q9WUR9	KAD4_MOUSE	Adenylate kinase 4, mitochondrial	3,42E-05	3,19E-05	3,84E-05	4,04E-05	3,62034E-05	3,8882E-06	10,740	3,08E-05	3,52E-05	3,38E-05	2,8E-05	3,22E-05	3,20003E-05	2,80504E-06	8,766
Q922E4	PCY2_MOUSE	Ethanolamine-phosphate cytidylyltransferase	3,31E-05	3,88E-05	3,89E-05	3,97E-05	3,76242E-05	3,06972E-06	8,159	3,78E-05	3,74E-05	3,2E-05	3,33E-05	3,73E-05	3,55585E-05	2,69485E-06	7,579
Q8R3P0	ACY2_MOUSE	Aspartoacylase	0,000115	0,000103	0,000102	0,00011	0,000107444	6,26973E-06	5,835	0,000112	8,43E-05	0,000109	9,49E-05	7,97E-05	9,60167E-05	1,44269E-05	15,025
Q9CXY6	ILF2_MOUSE	Interleukin enhancer-binding factor 2	0,000115	0,000137	0,000138	0,000132	0,000130665	1,05838E-05	8,100	0,00013	0,000112	0,000119	0,00012	0,000123	0,000120876	6,39019E-06	5,287
Q9CQZ5	NDUA6_MOUSE	NADH dehydrogenase [ubiquinone] 1 alpha subcomplex subunit 6	4,41E-05	4,59E-05	4,53E-05	4,72E-05	4,55902E-05	1,30212E-06	2,856	4,18E-05	5,28E-05	5,52E-05	4,32E-05	4,36E-05	4,73208E-05	6,19644E-06	13,095
Q8VHH5	AGAP3_MOUSE	Arf-GAP with GTPase, ANK repeat and PH domain-containing protein 3	3,69E-05	3,84E-05	4,19E-05	4,12E-05	3,95983E-05	2,31006E-06	5,834	4,12E-05	3,61E-05	3,85E-05	3,95E-05	3,7E-05	3,84668E-05	2,03011E-06	5,278
Q99JP7	GGT7_MOUSE	Gamma-glutamyltransferase 7	1,37E-05	1,36E-05	1,37E-05	1,33E-05	1,35766E-05	1,9921E-07	1,467	1,38E-05	1,24E-05	1,4E-05	1,1E-05	1,47E-05	1,31699E-05	1,47748E-06	11,219
O08586	PTEN_MOUSE	Phosphatidylinositol 3,4,5-trisphosphate 3-phosphatase and dual-specificity protein p	5,83E-06	3,29E-05	3,44E-05	3,64E-05	2,73962E-05	1,44501E-05	52,745	3,74E-05	3,66E-05	3,15E-05	3,54E-05	2,94E-05	3,40636E-05	3,45991E-06	10,157
O70591	PFD2_MOUSE	Prefoldin subunit 2	2,53E-05	2,69E-05	3E-05	2,79E-05	2,75437E-05	1,96769E-06	7,144	2,65E-05	2,99E-05	3,11E-05	3,2E-05	2,89E-05	2,96907E-05	2,12878E-06	7,170
Q78ZA7	NP1L4_MOUSE	Nucleosome assembly protein 1-like 4	4,22E-05	4,29E-05	3,99E-05	4,84E-05	4,33151E-05	3,59809E-06	8,307	4,07E-05	4,14E-05	5,63E-05	4,85E-05	4,74E-05	4,68676E-05	6,32797E-06	13,502
O70194	EIF3D_MOUSE	Eukaryotic translation initiation factor 3 subunit D	7,12E-05	7,93E-05	7,63E-05	7,47E-05	7,53826E-05	3,37438E-06	4,476	7,25E-05	7,16E-05	6,93E-05	6,91E-05	7,03E-05	7,05688E-05	1,45518E-06	2,062
Q5DTY9	KCD16_MOUSE	BTB/POZ domain-containing protein KCTD16	2,69E-05	2,97E-05	3,29E-05	2,99E-05	2,98435E-05	2,42003E-06	8,109	2,3E-05	2,07E-05	2,35E-05	2,31E-05	2,11E-05	2,22688E-05	1,28394E-06	5,766
P17665	COX7C_MOUSE	Cytochrome c oxidase subunit 7C, mitochondrial	0,000175	0,000185	0,000184	0,000161	0,000176393	1,12803E-05	6,395	0,00016	0,000172	0,000171	0,000171	0,000156	0,000166049	7,70369E-06	4,639
Q9QXS1	PLEC_MOUSE	Plectin	0,000127	0,000127	0,000126	0,000127	0,000126473	4,84047E-07	0,383	0,000147	0,000129	0,000124	0,00013	0,000135	0,000133028	8,73122E-06	6,563
P70336	ROCK2_MOUSE	Rho-associated protein kinase 2	0,000103	0,000103	9,92E-05	9,89E-05	0,000101037	2,32453E-06	2,301	0,000117	0,000103	9,95E-05	9,89E-05	0,000113	0,000106316	8,10651E-06	7,625
Q71LX4	TLN2_MOUSE	Talin-2	5,44E-06	6,25E-06	5,82E-06	6,2E-06	5,9269E-06	3,79862E-07	6,409	8,52E-06	8,93E-06	6,96E-06	6,08E-06	6,21E-06	7,34062E-06	1,31602E-06	17,928
Q9QYS2	GRM3_MOUSE	Metabotropic glutamate receptor 3	3,05E-05	2,82E-05	3,62E-05	3,75E-05	3,31076E-05	4,46978E-06	13,501	3,96E-05	2,59E-05	3,71E-05	4,12E-05	4,5E-05	3,77694E-05	7,20988E-06	19,089
Q62318	TIF1B_MOUSE	Transcription intermediary factor 1-beta	5,55E-06	8,41E-06	9,07E-06	7,44E-06	7,61628E-06	1,5306E-06	20,096	5,45E-06	7,29E-06	7,23E-06	8,47E-06	1,01E-05	7,71389E-06	1,73156E-06	22,447
Q6ZWX6	IF2A_MOUSE	Eukaryotic translation initiation factor 2 subunit 1	6,89E-05	7,1E-05	5,84E-05	6,3E-05	6,53201E-05	5,74096E-06	8,789	7,43E-05	6,49E-05	6,26E-05	6,21E-05	5,33E-05	6,3419E-05	7,51365E-06	11,848
P28650	PURA1_MOUSE	Adenylosuccinate synthetase isozyme 1	7,09E-05	8,86E-05	7,21E-05	6,83E-05	7,49521E-05	9,23351E-06	12,319	8,31E-05	9,95E-05	8,34E-05	7,9E-05	7,7E-05	8,44035E-05	8,85179E-06	10,487
Q9QZ88	VPS29_MOUSE	Vacuolar protein sorting-associated protein 29	0,000112	0,000129	0,000142	0,000132	0,000128586	1,2363E-05	9,615	0,000112	0,000101	0,000131	0,000115	0,000109	0,000113412	1,11301E-05	9,814

Q6P069	SORCN_MOUSE	Sorcin	8,15E-05	8,83E-05	8,34E-05	8,71E-05	8,50761E-05	3,1774E-06	3,735	7,14E-05	6,4E-05	8,27E-05	6,86E-05	5,68E-05	6,86956E-05	9,59685E-06	13,970
O70172	PI42A_MOUSE	Phosphatidylinositol 5-phosphate 4-kinase type-2 alpha	7,51E-05	0,000108	8,06E-05	8,72E-05	8,76027E-05	1,41616E-05	16,166	9,37E-05	6,78E-05	9,54E-05	0,000117	0,0001	9,47989E-05	1,76746E-05	18,644
P31786	ACBP_MOUSE	Acyl-CoA-binding protein	7,91E-05	0,000113	0,000136	0,000127	0,000114001	2,5136E-05	22,049	6,67E-05	7,76E-05	0,000108	0,000115	0,000103	9,41779E-05	2,08791E-05	22,170
P61957	SUMO2_MOUSE	Small ubiquitin-related modifier 2	2,73E-05	2,76E-05	3,59E-05	3,27E-05	3,08722E-05	4,15458E-06	13,457	2,46E-05	2,89E-05	2,9E-05	2,57E-05	2,33E-05	2,63153E-05	2,537E-06	9,641
Q91ZX7	LRP1_MOUSE	Prolow-density lipoprotein receptor-related protein 1	6,03E-05	7,33E-05	6,57E-05	6,26E-05	6,54594E-05	5,65475E-06	8,639	6,66E-05	6,6E-05	6,14E-05	6,24E-05	6,66E-05	6,46023E-05	2,51081E-06	3,887
Q8C1A5	THOP1_MOUSE	Thimet oligopeptidase	4,56E-05	4,62E-05	4,71E-05	5,19E-05	4,76809E-05	2,87254E-06	6,025	5,53E-05	5,42E-05	4,98E-05	5,56E-05	5,27E-05	5,35203E-05	2,36235E-06	4,414
Q06138	CAB39_MOUSE	Calcium-binding protein 39	0,000119	3,94E-05	8,04E-05	0,000111	8,74172E-05	3,6051E-05	41,240	0,000109	0,000113	9,83E-05	0,000118	8,92E-05	0,000105454	1,16701E-05	11,067
Q64516	GLPK_MOUSE	Glycerol kinase	9,67E-05	8,64E-05	9,17E-05	8,33E-05	8,95175E-05	5,93288E-06	6,628	7,82E-05	7,01E-05	7,54E-05	8,04E-05	6,92E-05	7,46771E-05	4,90543E-06	6,569
P49442	INPP_MOUSE	Inositol polyphosphate 1-phosphatase	9,63E-05	9,24E-05	9,89E-05	0,000108	9,88302E-05	6,45032E-06	6,527	8,91E-05	7,83E-05	8,36E-05	9,79E-05	8,58E-05	8,69368E-05	7,27774E-06	8,371
Q9CZX8	RS19_MOUSE	40S ribosomal protein S19	1,03E-05	9,52E-06	1,23E-05	1,23E-05	1,11287E-05	1,42855E-06	12,837	1,41E-05	1,64E-05	1,38E-05	1,32E-05	1,27E-05	1,40521E-05	1,43744E-06	10,229
P63037	DNJA1_MOUSE	DnaJ homolog subfamily A member 1	6,41E-05	7,74E-05	6,88E-05	7,09E-05	7,03029E-05	5,5161E-06	7,846	6,3E-05	6,57E-05	6,01E-05	6,95E-05	9,63E-05	7,09176E-05	1,45891E-05	20,572
Q80VP1	EPN1_MOUSE	Epsin-1	6,72E-05	7,2E-05	7,41E-05	7,78E-05	7,27863E-05	4,4294E-06	6,085	7,3E-05	6,3E-05	7,17E-05	8,62E-05	8,88E-05	7,6549E-05	1,07633E-05	14,061
Q9QZX7	SRR_MOUSE	Serine racemase	1,31E-05	1,36E-05	1,58E-05	1,4E-05	1,41391E-05	1,19305E-06	8,438	1,35E-05	1,16E-05	1,31E-05	8,1E-07	1,78E-05	1,13479E-05	6,3254E-06	55,741
P27659	RL3_MOUSE	60S ribosomal protein L3	7,53E-05	8,94E-05	7,86E-05	8,78E-05	8,27808E-05	6,86772E-06	8,296	8,66E-05	8,53E-05	8,67E-05	8,68E-05	7,75E-05	8,45622E-05	4,00934E-06	4,741
Q9D7X3	DUS3_MOUSE	Dual specificity protein phosphatase 3	3,68E-05	4,52E-05	4,45E-05	4,63E-05	4,31974E-05	4,34792E-06	10,065	4,79E-05	4,04E-05	4,34E-05	3,92E-05	4,07E-05	4,23093E-05	3,46319E-06	8,185
Q9D1D4	TMEDA_MOUSE	Transmembrane emp24 domain-containing protein 10	6,52E-05	7E-05	6,66E-05	6,94E-05	6,78044E-05	2,28005E-06	3,363	5,86E-05	6,77E-05	6,94E-05	5,36E-05	6,2E-05	6,22577E-05	6,51215E-06	10,460
Q80WG5	LRC8A_MOUSE	Volume-regulated anion channel subunit LRRC8A	4,83E-05	5,66E-05	3,78E-05	5,08E-05	4,83754E-05	7,87721E-06	16,284	5,37E-05	4,95E-05	5,1E-05	5,54E-05	5,16E-05	5,22505E-05	2,32878E-06	4,457
Q99LG2	TNPO2_MOUSE	Transportin-2	2,39E-05	2,65E-05	2,48E-05	2,65E-05	2,54616E-05	1,28907E-06	5,063	2,42E-05	1,68E-05	2E-05	2,83E-05	2,19E-05	2,22307E-05	4,31909E-06	19,428
Q9DCT8	CRIP2_MOUSE	Cysteine-rich protein 2	4,25E-05	3,96E-05	3,85E-05	4,17E-05	4,05766E-05	1,87274E-06	4,615	4,58E-05	4,94E-05	4,04E-05	4,31E-05	5,46E-05	4,6669E-05	5,54042E-06	11,872
Q68FH0	PKP4_MOUSE	Plakophilin-4	2,46E-05	2,99E-05	3,18E-05	3,16E-05	2,94615E-05	3,37119E-06	11,443	3,2E-05	2,83E-05	2,71E-05	2,87E-05	2,83E-05	2,88664E-05	1,85735E-06	6,434
Q8BFZ3	ACTBL_MOUSE	Beta-actin-like protein 2	5,12E-05	5,26E-05	5,51E-05	6,35E-05	5,56127E-05	5,50941E-06	9,907	4,98E-05	4,52E-05	5,44E-05	0,000167	6,14E-05	7,55344E-05	5,14566E-05	68,123
Q4V9Z5	SE6L2_MOUSE	Seizure 6-like protein 2	2,39E-05	3,63E-05	3,68E-05	3,86E-05	3,38905E-05	6,75005E-06	19,917	3,77E-05	1,6E-05	3,5E-05	3,75E-05	3,09E-05	3,14136E-05	9,04906E-06	28,806
Q921G7	ETFD_MOUSE	Electron transfer flavoprotein-ubiquinone oxidoreductase, mitochondrial	0,000118	0,000117	0,000107	9,88E-05	0,000110172	9,12376E-06	8,281	8,94E-05	9,05E-05	0,000102	8,35E-05	9,21E-05	9,13982E-05	6,52408E-06	7,138
Q8BLJ3	PLCX3_MOUSE	PI-PLC X domain-containing protein 3	4,22E-05	6,45E-05	7,38E-05	7,14E-05	6,29972E-05	1,4403E-05	22,863	5,62E-05	5,53E-05	6,7E-05	5,85E-05	5,52E-05	5,84482E-05	4,93999E-06	8,452
Q924M7	MPI_MOUSE	Mannose-6-phosphate isomerase	5,85E-05	7,17E-05	6,68E-05	6,59E-05	6,57393E-05	5,43867E-06	8,273	6,5E-05	5,8E-05	5,89E-05	6,27E-05	5,6E-05	6,01346E-05	3,66926E-06	6,102
Q5SRX1	TM1L2_MOUSE	TOM1-like protein 2	0,000106	0,000106	0,000112	0,000104	0,000107224	3,45179E-06	3,219	9,86E-05	9,28E-05	0,000108	0,0001	9,63E-05	9,91615E-05	5,58703E-06	5,634
Q9Z1P6	NDUA7_MOUSE	NADH dehydrogenase [ubiquinone] 1 alpha subcomplex subunit 7	5,02E-05	5,85E-05	4,29E-05	5,34E-05	5,12366E-05	6,52268E-06	12,731	5,05E-05	5,91E-05	5,99E-05	5,4E-05	4,53E-05	5,37491E-05	6,09795E-06	11,345
Q8VHJ5	MARK1_MOUSE	Serine/threonine-protein kinase MARK1	1,49E-05	1,4E-05	1,64E-05	1,45E-05	1,49833E-05	1,04618E-06	6,982	1,81E-05	1,24E-05	1,63E-05	1,27E-05	1,61E-05	1,51218E-05	2,46183E-06	16,280
Q68FL6	SYMC_MOUSE	Methionine--tRNA ligase, cytoplasmic	1,51E-05	1,6E-05	1,45E-05	1,17E-05	1,43176E-05	1,84854E-06	12,911	1,59E-05	1,38E-05	1,54E-05	1,51E-05	1,3E-05	1,46587E-05	1,20465E-06	8,218
Q9CPX6	ATG3_MOUSE	Ubiquitin-like-conjugating enzyme ATG3	1,2E-05	1,48E-05	1,18E-05	1,56E-05	1,35566E-05	1,93604E-06	14,281	1,14E-05	1,24E-05	1,18E-05	1,48E-05	1,26E-05	1,25949E-05	1,31494E-06	10,440
P97823	LYPA1_MOUSE	Acyl-protein thioesterase 1	1,52E-05	1,65E-05	1,41E-05	1,23E-05	1,45225E-05	1,77404E-06	12,216	1,37E-05	1,64E-05	1,45E-05	1,14E-05	1,16E-05	1,35185E-05	2,07047E-06	15,316
P62737	ACTA_MOUSE	#N/A	1,59E-05	2,44E-05	2,6E-05	1,43E-05	2,01726E-05	5,8915E-06	29,205	1,97E-05	2,98E-05	2,35E-05	1,66E-05	1,52E-05	2,09418E-05	5,87873E-06	28,072
Q61301	CTNA2_MOUSE	Catenin alpha-2	8,64E-06	6,35E-06	6,47E-06	7,38E-06	7,20974E-06	1,05564E-06	14,642	9,05E-06	9,83E-06	8,7E-06	7,4E-06	7,25E-06	8,44631E-06	1,1017E-06	13,044
P11627	L1CAM_MOUSE	Neural cell adhesion molecule L1	0,000153	0,000165	0,000174	0,000154	0,000161454	1,0237E-05	6,340	0,000173	0,00017	0,000155	0,000143	0,000167	0,000161619	1,2592E-05	7,791
Q8R0S2	IQEC1_MOUSE	IQ motif and SEC7 domain-containing protein 1	8,42E-05	9,83E-05	9E-05	0,000107	9,49878E-05	1,01322E-05	10,667	0,000111	9,94E-05	9,83E-05	9,74E-05	0,000115	0,000104146	8,06099E-06	7,740
Q9R1P4	PSA1_MOUSE	Proteasome subunit alpha type-1	0,000153	0,00015	0,00014	0,000153	0,000149057	6,15278E-06	4,128	0,000152	0,000148	0,000151	0,000126	0,000138	0,000142987	1,0872E-05	7,603
Q00PI9	HNRL2_MOUSE	Heterogeneous nuclear ribonucleoprotein U-like protein 2	0,000141	0,00014	0,00013	0,000145	0,000138916	6,54642E-06	4,713	0,000164	0,000151	0,000145	0,000139	0,000162	0,000151991	1,06187E-05	6,986
Q3UHJ0	AAK1_MOUSE	AP2-associated protein kinase 1	6,55E-05	6,4E-05	6,13E-05	5,24E-05	6,08101E-05	5,83731E-06	9,599	6,73E-05	5,23E-05	5,83E-05	5,6E-05	6,01E-05	5,88189E-05	5,56443E-06	9,460
Q8BGT8	PHIPL_MOUSE	Phytanoyl-CoA hydroxylase-interacting protein-like	7,14E-05	7,25E-05	7,55E-05	7,7E-05	7,40968E-05	2,59446E-06	3,501	8,04E-05	7,72E-05	8,22E-05	7,39E-05	6,68E-05	7,60844E-05	6,11021E-06	8,031
P23506	PIMT_MOUSE	Protein-L-isoaspartate(D-aspartate) O-methyltransferase	3,47E-05	3,75E-05	3,55E-05	4,6E-05	3,84067E-05	5,20433E-06	13,551	4,35E-05	3,46E-06	4,51E-05	4,65E-05	3,66E-05	3,50425E-05	1,80658E-05	51,554
Q9EP69	SAC1_MOUSE	Phosphatidylinositide phosphatase SAC1	0,000108	0,000104	8,33E-05	8,85E-05	9,5988E-05	1,19778E-05	12,478	8,27E-05	9,62E-05	9,3E-05	9,15E-05	8,23E-05	8,91257E-05	6,31417E-06	7,085
P28474	ADHX_MOUSE	Alcohol dehydrogenase class-3	5,81E-05	5,5E-05	5,58E-05	6,02E-05	5,7272E-05	2,35081E-06	4,105	5,92E-05	5,18E-05	4,86E-05	4,68E-05	4,64E-05	5,0574E-05	5,28119E-06	10,443
Q9DB72	BTBDH_MOUSE	BTB/POZ domain-containing protein 17	9,21E-05	7,94E-05	6,79E-05	8,2E-05	8,03339E-05	9,9486E-06	12,384	0,000106	6,1E-05	8,58E-05	8,71E-05	7,58E-05	8,30686E-05	1,63365E-05	19,666
Q8BH04	PCKGM_MOUSE	Phosphoenolpyruvate carboxykinase [GTP], mitochondrial	3,83E-05	4,64E-05	4,31E-05	4,57E-05	4,33831E-05	3,69515E-06	8,517	4,03E-05	4,12E-05	4,15E-05	4,13E-05	3,98E-05	4,08065E-05	7,45417E-07	1,827
Q9CYR6	AGM1_MOUSE	Phosphoacetylglucosamine mutase	1,47E-05	1,98E-05	1,71E-05	1,98E-05	1,78833E-05	2,45954E-06	13,753	1,91E-05	1,84E-05	2,21E-05	2,28E-05	2,17E-05	2,08202E-05	1,96119E-06	9,420
Q9DCJ5	NDUA8_MOUSE	NADH dehydrogenase [ubiquinone] 1 alpha subcomplex subunit 8	0,000157	0,000114	0,000138	0,000162	0,000142681	2,19826E-05	15,407	0,000129	0,000162	0,000153	0,000129	0,000141	0,000142884	1,49627E-05	10,472
Q9CQI6	COTL1_MOUSE	Coactosin-like protein	0,000224	0,00031	0,000302	0,000306	0,000285572	4,13525E-05	14,481	0,000219	0,00024	0,000288	0,000269	0,000251	0,000253257	2,65635E-05	10,489
P62334	PRS10_MOUSE	26S protease regulatory subunit 10B	9,33E-05	0,000105	0,000109	0,000106	0,000103358	6,9206E-06	6,696	0,000117	0,00011	0,000116	0,000123	0,000115	0,000116084	4,46966E-06	3,850
Q9JLJ2	AL9A1_MOUSE	4-trimethylaminobutyraldehyde dehydrogenase	8,82E-05	8,87E-05	8,38E-05	8,52E-05	8,65154E-05	2,36183E-06	2,730	8,01E-05	9,45E-05	9,37E-05	8,83E-05	7,67E-05	8,66421E-05	7,98811E-06	9,220
Q9CQW1	YKT6_MOUSE	Synaptobrevin homolog YKT6	0,000121	0,00011	0,00012	0,000116	0,000116534	5,00055E-06	4,291	0,000125	0,000121	0,000116	8,13E-05	0,000103	0,000109217	1,766E-05	16,170
P24472	GSTA4_MOUSE	Glutathione S-transferase A4	0,000141	0,000153	0,000163	0,000172	0,000157285	1,36588E-05	8,684	0,000123	0,000161	0,000157	0,000128	0,000114	0,00013645	2,1353E-05	15,649
Q80UM3	NAA15_MOUSE	N-alpha-acetyltransferase 15, NatA auxiliary subunit	1,4E-05	1,89E-05	1,56E-05	1,76E-05	1,65388E-05	2,16583E-06	13,095	1,75E-05	1,59E-05	1,55E-05	1,85E-05	1,45E-05	1,63532E-05	1,58554E-06	9,696
O08795	GLU2B_MOUSE	Glucosidase 2 subunit beta	1,46E-05	1,58E-05	1,42E-05	1,54E-05	1,49819E-05	7,31247E-07	4,881	1,32E-05	1,89E-05	1,66E-05	1,43E-05	1,6E-05	1,58226E-05	2,20564E-06	13,940
P24529	TY3H_MOUSE	Tyrosine 3-monooxygenase	2,98E-05	2,13E-05	3,74E-05	2,39E-05	2,80917E-05	7,1315E-06	25,387	3,82E-05	2,11E-05	2,22E-05	3,13E-05	4,71E-05	3,19745E-05	1,09631E-05	34,287
P49722	PSA2_MOUSE	Proteasome subunit alpha type-2	3,12E-05	4,53E-05	4,75E-05	4,35E-05	4,18889E-05	7,33845E-06	17,519	4,58E-05	4,79E-05	5,31E-05	5,28E-05	4,24E-05	4,83919E-05	4,57091E-06	9,446
Q99KC8	VMA5A_MOUSE	von Willebrand factor A domain-containing protein 5A	6,51E-05	8,14E-05	6,59E-05	7,37E-05	7,15124E-05	7,64155E-06	10,686	6,22E-05	6,24E-05	7,25E-05	6,99E-05	6,82E-05	6,70368E-05	4,60144E-06	6,864
P63242	IF5A1_MOUSE	Eukaryotic translation initiation factor 5A-1	0,000117	0,000146	0,000143	0,00014	0,000136394	1,3332E-05	9,775	0,000132	0,000117	0,000149	0,000134	0,000127	0,000131591	1,18038E-05	8,970
Q8BGD9	IF4B_MOUSE	Eukaryotic translation initiation factor 4B	1,89E-06	2,65E-06	1,3E-06	1,79E-06	1,908E-06	5,55354E-07	29,107	1,8E-06	1,97E-06	1,36E-06	2,21E-06	1,4E-06	1,74868E-06	3,67723E-07	21,029
P35293	RAB18_MOUSE	Ras-related protein Rab-18	6,43E-05	7,69E-05	7,87E-05	7,84E-05	7,45856E-05	6,87466E-06	9,217	6,82E-05	6,44E-05	7,68E-05	7,18E-05	5,67E-05	6,759E-05	7,64211E-06	11,307
Q80TL0	PPM1E_MOUSE	Protein phosphatase 1E	5,51E-05	6,5E-05	6,36E-05	6,93E-05	6,32241E-05	5,93802E-06	9,392	5,86E-05	6,16E-05	6,31E-05	5,38E-05	6,24E-05	5,98882E-05	3,83319E-06	6,401
Q501J6	DDX17_MOUSE	Probable ATP-dependent RNA helicase DDX17	3,26E-05	3,41E-05	3,61E-05	3,41E-05	3,42069E-05	1,44417E-06	4,222	2,97E-05	2,85E-05	3,38E-05	2,84E-05	3,49E-05	3,10404E-05	3,08962E-06	9,954
O35593	PSDE_MOUSE	26S proteasome non-ATPase regulatory subunit 14	4,42E-05	5,91E-05	5,97E-05	5,63E-05	5,48443E-05	7,22541E-06	13,174	5,28E-05	5,38E-05	5,34E-05	4,91E-05	4,46E-05	5,07396E-05	3,91189E-06	7,710
P62082	RS7_MOUSE	40S ribosomal protein S7	2,88E-05	3,18E-05	3,48E-05	3,77E-05	3,32824E-05	3,85421E-06	11,580	2,86E-05	2,49E-05	3,16E-05	2,74E-05	2,89E-05	2,82621E-05	2,43919E-06	8,631
Q2NL51	GSK3A_MOUSE	Glycogen synthase kinase-3 alpha	0,000101	0,000104	0,00012	0,000104	0,000107472	8,74536E-06	8,137	0,000111	0,000101	0,000114	0,000109	0,000104	0,000107839	5,00984E-06	4,646
Q9D023	MPC2_MOUSE	Mitochondrial pyruvate carrier 2	6,18E-05	5,45E-05	5,62E-05	4,91E-05	5,54193E-05	5,25305E-06	9,479	5,29E-05	5,41E-05	5,26E-05	5,25E-05	4,35E-05	5,11158E-05	4,30016E-06	8,413
Q9CZY3	UB2V1_MOUSE	Ubiquitin-conjugating enzyme E2 variant 1	2,31E-05	2,47E-05	2,42E-05	2,37E-05	2,39279E-05	6,73781E-07	2,816	2,77E-05	2,88E-05	3,1E-05	2,71E-05	2,54E-05	2,79814E-05	2,08847E-06	7,464
Q61102	ABCB7_MOUSE	ATP-binding cassette sub-family B member 7, mitochondrial	4,07E-05	3,4E-05	3,39E-05	3,19E-05	3,51268E-05	3,8295E-06	10,902	3,84E-05	3,33E-05	3,09E-05	3,54E-05	3,3E-05	3,42187E-05	2,83378E-06	8,281
O35682	MYADM_MOUSE	Myeloid-associated differentiation marker	2,7E-05	2,21E-05	2,39E-05	2,04E-05	2,33786E-05	2,84223E-06	12,157	2,48E-05	2,24E-05	2,13E-05	1,84E-05	1,84E-05	2,10373E-05	2,72784E-06	12,967
P85094	ISC2A_MOUSE	Isochorismatase domain-containing protein 2A	9,1E-05	8,63E-05	9,74E-05	7,61E-05	8,76853E-05	8,94254E-06	10,198	7,65E-05	9,31E-05	7,9E-05	6,1E-05	7,53E-05	7,69796E-05	1,14287E-05	14,846
Q8BGY7	F210A_MOUSE	Protein FAM210A	4,75E-05	5,34E-05	4,42E-05	4,46E-05	4,74112E-05	4,21876E-06	8,898	4,14E-05	3,9E-05	4E-05	3,93E-05	1,05E-05	3,40229E-05	1,31937E-05	38,779
Q9CR51	VATG1_MOUSE	V-type proton ATPase subunit G 1	1,73E-05	2,35E-05	2,35E-05	2,17E-05	2,15059E-05	2,91253E-06	13,543	1,7E-05	2,21E-05	2,34E-05	1,92E-05	1,93E-05	2,02092E-05	2,53753E-06	12,556

Q9QUH0	GLRX1_MOUSE	Glutaredoxin-1	2,48E-05	3,48E-05	3,96E-05	3,3E-05	3,30636E-05	6,19443E-06	18,735	3,18E-05	3,08E-05	2,86E-05	3,57E-05	3,23E-05	3,18354E-05	2,58333E-06	8,115
Q8CGP0	H2B3B_MOUSE	Histone H2B type 3-B	3,76E-05	4,72E-05	4,52E-05	4,84E-05	4,46024E-05	4,86406E-06	10,905	4,2E-05	4,64E-05	5,1E-05	4,65E-05	4,22E-05	4,5611E-05	3,71354E-06	8,142
Q9JIG8	PRAF2_MOUSE	PRA1 family protein 2	8,67E-05	9,38E-05	9,04E-05	8,59E-05	8,92022E-05	3,6113E-06	4,048	8,16E-05	8,22E-05	8,2E-05	7,93E-05	8,1E-05	8,12143E-05	1,16807E-06	1,438
P70398	USP9X_MOUSE	Probable ubiquitin carboxyl-terminal hydrolase FAF-X	3,43E-05	4,26E-05	3,9E-05	3,97E-05	3,89133E-05	3,43887E-06	8,837	4,28E-05	4,14E-05	3,45E-05	3,6E-05	3,59E-05	3,81375E-05	3,71792E-06	9,749
Q99K51	PLST_MOUSE	Plastin-3	5,95E-05	5,36E-05	4,66E-05	5,53E-05	5,37447E-05	5,4026E-06	10,052	6,67E-05	5,53E-05	5,41E-05	4,81E-05	5,6E-05	5,60429E-05	6,74409E-06	12,034
Q6P5F9	XPO1_MOUSE	Exportin-1	4,71E-05	4,86E-05	4,45E-05	5,13E-05	4,78719E-05	2,82551E-06	5,902	4,14E-05	4,29E-05	4,57E-05	4,05E-05	5,16E-05	4,44352E-05	4,49837E-06	10,123
P08003	PDIA4_MOUSE	Protein disulfide-isomerase A4	3,68E-05	3,45E-05	3,11E-05	3,77E-05	3,50068E-05	2,9314E-06	8,374	1,03E-05	3,39E-05	3,51E-05	2,81E-05	3,77E-05	2,90213E-05	1,10132E-05	37,949
P14733	LMNB1_MOUSE	Lamin-B1	6,18E-05	6,18E-05	5,98E-05	6,53E-05	6,21644E-05	2,2987E-06	3,698	7,48E-05	7,17E-05	7,14E-05	7,79E-05	7,88E-05	7,49115E-05	3,4068E-06	4,548
Q80XN0	BDH_MOUSE	D-beta-hydroxybutyrate dehydrogenase, mitochondrial	9,82E-05	0,000114	0,000118	0,000141	0,000117827	1,76285E-05	14,961	0,000117	0,000123	0,000128	0,000133	9,53E-05	0,000119342	1,47637E-05	12,371
Q8BWT1	THIM_MOUSE	3-ketoacyl-CoA thiolase, mitochondrial	0,000113	0,000113	0,000122	0,000113	0,000115125	4,33504E-06	3,765	2,27E-05	9,96E-05	0,000115	9,17E-05	0,000117	8,9249E-05	3,8676E-05	43,335
Q8BH66	ATLA1_MOUSE	Atlastin-1	4,23E-05	4,53E-05	4,05E-05	3,71E-05	4,13069E-05	3,42243E-06	8,285	3,46E-05	3,68E-05	4,05E-05	3,68E-05	3,46E-05	3,6662E-05	2,39683E-06	6,538
E9Q6P5	TTC7B_MOUSE	Tetratricopeptide repeat protein 7B	5,65E-05	5,92E-05	5,38E-05	6,42E-05	5,8437E-05	4,40346E-06	7,535	5,76E-05	5,06E-05	4,93E-05	6,16E-05	5,71E-05	5,52325E-05	5,12833E-06	9,285
O88447	KLC1_MOUSE	Kinesin light chain 1	3,16E-05	3,19E-05	2,82E-05	3,38E-05	3,1379E-05	2,3432E-06	7,467	3,24E-05	2,96E-05	3,1E-05	3,08E-05	2,83E-05	3,04261E-05	1,55E-06	5,094
Q8BJY1	PSMD5_MOUSE	26S proteasome non-ATPase regulatory subunit 5	9,19E-05	0,000109	0,000102	9,87E-05	0,000100347	6,99473E-06	6,971	9,63E-05	9,76E-05	0,000105	0,000102	7,31E-05	9,48273E-05	1,26194E-05	13,308
Q8QZY1	EIF3L_MOUSE	Eukaryotic translation initiation factor 3 subunit L	2,97E-05	3,8E-05	3,47E-05	3,68E-05	3,47811E-05	3,68061E-06	10,582	2,78E-05	1,39E-05	2,75E-05	3,39E-05	2,3E-05	2,52037E-05	7,41226E-06	29,409
Q9QXB9	DRG2_MOUSE	Developmentally-regulated GTP-binding protein 2	9,12E-05	1E-04	9,7E-05	9,44E-05	9,56419E-05	3,75712E-06	3,928	9,03E-05	7,7E-05	8,28E-05	7,55E-05	7,24E-05	7,96139E-05	7,08251E-06	8,896
Q8CG76	ARK72_MOUSE	Aflatoxin B1 aldehyde reductase member 2	1,7E-05	1,71E-05	1,88E-05	1,9E-05	1,79487E-05	1,05266E-06	5,865	1,82E-05	2,25E-05	1,94E-05	1,7E-05	1,79E-05	1,89773E-05	2,12907E-06	11,219
Q8C8N2	SCAI_MOUSE	Protein SCAI	3,29E-05	4,14E-05	3,3E-05	4,01E-05	3,68489E-05	4,52518E-06	12,280	4,52E-05	3,48E-05	4,51E-05	4,57E-06	5E-05	3,59191E-05	1,83736E-05	51,153
P04925	PRIO_MOUSE	Major prion protein	9,5E-05	9,49E-05	8,76E-05	0,000108	9,63971E-05	8,4906E-06	8,808	0,000108	0,000108	0,000131	0,000123	0,000112	0,00011626	1,04192E-05	8,962
Q8K0D5	EFGM_MOUSE	Elongation factor G, mitochondrial	2,04E-05	2,23E-05	2,31E-05	1,89E-05	2,11555E-05	1,9234E-06	9,092	2,41E-05	2,37E-05	2,39E-05	2,3E-05	2,25E-05	2,34428E-05	6,58069E-07	2,807
Q8BG73	SH3L2_MOUSE	SH3 domain-binding glutamic acid-rich-like protein 2	2,55E-05	2,63E-05	3,26E-05	3,08E-05	2,88031E-05	3,45812E-06	12,006	2,66E-05	2,45E-05	2,72E-05	2,48E-05	2,26E-05	2,51386E-05	1,84384E-06	7,335
P99026	PSB4_MOUSE	Proteasome subunit beta type-4	3,95E-05	4,12E-05	4,87E-05	4,37E-05	4,32814E-05	3,98625E-06	9,210	5,78E-05	4,66E-05	6,98E-05	6,53E-05	6,05E-05	6,00155E-05	8,78453E-06	14,637
Q01768	NDKB_MOUSE	Nucleoside diphosphate kinase B	0,000135	0,000153	0,000153	0,000161	0,000150493	1,07189E-05	7,123	0,000132	0,000145	0,000166	0,000138	0,000131	0,000142413	1,44018E-05	10,113
Q8C0L0	TMX4_MOUSE	Thioredoxin-related transmembrane protein 4	9,83E-05	9,76E-05	0,000113	0,000108	0,000104262	7,57227E-06	7,263	9,86E-05	8,65E-05	9,68E-05	9,31E-05	9,54E-05	9,40606E-05	4,68384E-06	4,980
P61358	RL27_MOUSE	60S ribosomal protein L27	6,22E-05	8,58E-05	0,000113	9,34E-05	8,84847E-05	2,08231E-05	23,533	6,59E-05	6,71E-05	0,000106	7,2E-05	8,33E-05	7,89182E-05	1,67707E-05	21,251
P61022	CHP1_MOUSE	Calcineurin B homologous protein 1	5,98E-05	5,81E-05	5,99E-05	6,04E-05	5,9569E-05	9,81296E-07	1,647	6,08E-05	5,68E-05	6,46E-05	6,27E-05	4,79E-05	5,85744E-05	6,60796E-06	11,281
Q9D0L7	ARM10_MOUSE	Armadillo repeat-containing protein 10	4,89E-05	4,34E-05	4,5E-05	4,83E-05	4,64199E-05	2,65967E-06	5,730	3,02E-06	4,13E-05	6,14E-05	5,64E-05	5,95E-05	4,43284E-05	2,44053E-05	55,056
P62855	RS26_MOUSE	40S ribosomal protein S26	2,8E-05	7,82E-05	7,8E-05	6,91E-05	6,33264E-05	2,3919E-05	37,771	5,58E-05	6,42E-05	6,96E-05	7,24E-05	5,1E-05	6,25972E-05	9,0724E-06	14,493
Q6P542	ABCF1_MOUSE	ATP-binding cassette sub-family F member 1	1,56E-05	1,37E-05	1,34E-05	1,44E-05	1,42765E-05	9,91879E-07	6,948	1,5E-05	1,3E-05	1,25E-05	1,43E-05	1,32E-05	1,36079E-05	9,92935E-07	7,297
Q3THW5	H2AV_MOUSE	Histone H2A.V	0,000145	0,000157	0,000172	0,000158	0,000158068	1,09608E-05	6,934	0,000157	0,000167	0,000187	0,000148	0,000161	0,000163865	1,45285E-05	8,866
O08530	S1PR1_MOUSE	Sphingosine 1-phosphate receptor 1	2,64E-05	3,24E-05	3,24E-05	3,08E-05	3,04744E-05	2,81529E-06	9,238	3,02E-05	3,61E-05	2,93E-05	2,81E-05	3,01E-05	3,07684E-05	3,08673E-06	10,032
Q9DCP2	S38A3_MOUSE	Sodium-coupled neutral amino acid transporter 3	0,000118	9,35E-05	0,000104	9,96E-05	0,000103784	1,03507E-05	9,973	9,55E-05	0,000101	8,45E-05	7,67E-05	8,43E-05	8,83854E-05	9,69669E-06	10,971
O35683	NDUA1_MOUSE	NADH dehydrogenase [ubiquinone] 1 alpha subcomplex subunit 1	8,86E-05	0,000108	0,000103	0,000107	0,000101647	9,05206E-06	8,905	9,19E-05	9,09E-05	0,000114	0,000112	0,000104	0,000102543	1,07674E-05	10,500
Q8BP47	SYNC_MOUSE	Asparagine--tRNA ligase, cytoplasmic	0,000186	0,000239	0,000241	0,000257	0,000230757	3,10593E-05	13,460	0,000209	0,000156	0,000225	0,000222	0,000212	0,000204982	2,80508E-05	13,685
P36916	GNL1_MOUSE	Guanine nucleotide-binding protein-like 1	4,58E-05	4,79E-05	5,15E-05	4,84E-05	4,84065E-05	2,35623E-06	4,868	5,04E-05	4,83E-05	5,72E-05	5,59E-05	1,67E-05	4,5691E-05	1,66227E-05	36,381
Q9Z2D6	MECP2_MOUSE	Methyl-CpG-binding protein 2	9,88E-05	0,000102	9,45E-05	8,09E-05	9,41358E-05	9,42951E-06	10,017	9,76E-05	0,000102	0,000108	9,34E-05	9,18E-05	9,84875E-05	6,55402E-06	6,655
Q9D5V5	CUL5_MOUSE	Cullin-5	3,55E-05	4,18E-05	4,16E-05	4,14E-05	4,00854E-05	3,05893E-06	7,631	3,55E-05	3,26E-05	3,55E-05	3,46E-05	3,98E-05	3,55951E-05	2,62567E-06	7,376
Q8QZS1	HIBCH_MOUSE	3-hydroxyisobutyryl-CoA hydrolase, mitochondrial	4,51E-05	5,02E-05	4,56E-05	4,94E-05	4,75551E-05	2,57391E-06	5,412	5,27E-05	4,88E-05	5,08E-05	5,73E-05	5,16E-05	5,22401E-05	3,17133E-06	6,071
Q9WTX6	CUL1_MOUSE	Cullin-1	4,72E-05	5,74E-05	4,88E-05	5,19E-05	5,13177E-05	4,5304E-06	8,828	4,82E-05	1,89E-05	5,42E-05	5,12E-05	5,07E-05	4,46223E-05	1,45416E-05	32,588
P16332	MUTA_MOUSE	Methylmalonyl-CoA mutase, mitochondrial	2,07E-05	2,09E-05	1,74E-05	2,2E-05	2,02546E-05	1,95905E-06	9,672	1,96E-05	2,26E-05	2,17E-05	2,32E-05	2,17E-05	2,17546E-05	1,35332E-06	6,221
Q6NZL0	SOGA3_MOUSE	Protein SOGA3	2,34E-05	2,43E-05	2,05E-05	3,37E-06	1,78885E-05	9,81474E-06	54,866	2,74E-05	2,75E-05	2,45E-05	2,09E-05	2,13E-05	2,43449E-05	3,18478E-06	13,082
P62918	RL8_MOUSE	60S ribosomal protein L8	5,95E-05	6,47E-05	6,59E-05	8,22E-05	6,80763E-05	9,85465E-06	14,476	6,24E-05	6,71E-05	6,81E-05	5,27E-05	4,55E-05	5,91713E-05	9,76153E-06	16,497
O88543	CSN3_MOUSE	COP9 signalosome complex subunit 3	6,34E-05	7,68E-05	5,97E-05	7,86E-05	6,96253E-05	9,46173E-06	13,590	7,15E-05	6,42E-05	7,7E-05	7,23E-05	7,47E-05	7,19291E-05	4,85218E-06	6,746
Q9Z2H2	RGS6_MOUSE	Regulator of G-protein signaling 6	4,52E-05	4,48E-05	4,53E-05	3,95E-05	4,37276E-05	2,81233E-06	6,431	5,8E-05	5,08E-05	4,66E-05	4,9E-05	4,07E-05	4,90196E-05	6,32488E-06	12,903
P60843	IF4A1_MOUSE	Eukaryotic initiation factor 4A-I	4E-05	5,73E-05	5,92E-05	5,97E-05	5,40597E-05	9,43208E-06	17,448	5,89E-05	4,8E-05	5,68E-05	5,64E-05	6,52E-05	5,70768E-05	6,15531E-06	10,784
Q9CQI3	GMFB_MOUSE	Glia maturation factor beta	0,000122	0,000142	0,000139	0,000111	0,00012838	1,45856E-05	11,361	0,000117	0,000125	0,000138	0,000114	0,000104	0,000119646	1,26183E-05	10,546
P35980	RL18_MOUSE	60S ribosomal protein L18	6,96E-05	0,000125	0,000147	0,000145	0,000121639	3,60759E-05	29,658	2,95E-05	9,39E-05	0,000125	0,00012	0,000117	9,71085E-05	3,96266E-05	40,807
P70280	VAMP7_MOUSE	Vesicle-associated membrane protein 7	1,48E-05	1,49E-05	1,63E-05	1,75E-05	1,58634E-05	1,24585E-06	7,854	1,46E-05	1,15E-05	1,49E-05	1,8E-05	1,29E-05	1,43877E-05	2,43023E-06	16,891
P61750	ARF4_MOUSE	ADP-ribosylation factor 4	4,61E-05	5,58E-05	5,89E-05	2,62E-05	4,67352E-05	1,47588E-05	31,580	4,32E-05	5,48E-05	6,05E-05	5,06E-05	5,29E-05	5,24052E-05	6,34461E-06	12,107
Q9Z127	LAT1_MOUSE	Large neutral amino acids transporter small subunit 1	2,96E-05	3,39E-05	2,75E-05	1,51E-06	2,31238E-05	1,46546E-05	63,375	3,06E-05	3,07E-05	2,71E-05	2,7E-05	2E-05	2,70772E-05	4,34238E-06	16,037
Q9D379	HYEP_MOUSE	Epoxide hydrolase 1	3,85E-05	4,1E-05	3,81E-05	3,63E-05	3,84615E-05	1,96879E-06	5,119	3,37E-05	4,13E-05	4,08E-05	3,64E-05	3,59E-05	3,76313E-05	3,30008E-06	8,770
Q8VCN9	TBCC_MOUSE	Tubulin-specific chaperone C	1,38E-05	1,66E-05	1,62E-05	1,6E-05	1,56569E-05	1,24187E-06	7,932	1,67E-05	1,63E-05	1,52E-05	1,59E-05	1,27E-05	1,53514E-05	1,60677E-06	10,467
Q5HZI9	S2551_MOUSE	Solute carrier family 25 member 51	1,36E-05	1,5E-05	1,85E-05	1,74E-05	1,61176E-05	2,24183E-06	13,909	1,17E-05	1,46E-05	1,84E-05	1,79E-05	1,73E-05	1,60263E-05	2,8098E-06	17,532
Q5SYD0	MYO1D_MOUSE	Unconventional myosin-Id	4,43E-05	4,66E-05	3,48E-05	3,73E-05	4,07754E-05	5,60132E-06	13,737	4,09E-05	3,81E-05	4,39E-05	4,46E-05	3,93E-05	4,13668E-05	2,83043E-06	6,842
Q9JLZ3	AUHM_MOUSE	Methylglutaconyl-CoA hydratase, mitochondrial	0,000223	0,000192	0,000176	0,000218	0,00020226	2,21803E-05	10,966	0,0002	0,000209	0,000198	0,00018	0,000155	0,000188193	2,16744E-05	11,517
O54829	RGS7_MOUSE	Regulator of G-protein signaling 7	5,01E-05	5,85E-05	5,8E-05	5,35E-05	5,5009E-05	3,95726E-06	7,194	6,08E-05	5,28E-05	5,09E-05	5,46E-05	6,4E-06	4,51041E-05	2,19559E-05	48,678
Q9D855	QCR7_MOUSE	Cytochrome b-c1 complex subunit 7	6,02E-05	6,97E-05	6,26E-05	7,11E-05	6,59014E-05	5,32694E-06	8,083	4,49E-05	5,41E-05	5,66E-05	5,19E-05	4,32E-05	5,01496E-05	5,83712E-06	11,639
Q8C7X2	EMC1_MOUSE	ER membrane protein complex subunit 1	1,59E-05	1,66E-05	1,58E-05	1,67E-05	1,62544E-05	4,67031E-07	2,873	1,3E-05	1,37E-05	1,29E-05	1,44E-05	1,42E-05	1,36289E-05	6,67504E-07	4,898
P61255	RL26_MOUSE	60S ribosomal protein L26	5,94E-05	3,47E-05	5,01E-05	6,83E-05	5,31521E-05	1,43515E-05	27,001	5,13E-05	4,49E-05	5,5E-05	4,3E-05	2,99E-05	4,49997E-05	9,79386E-06	21,764
P23492	PNPH_MOUSE	Purine nucleoside phosphorylase	8,6E-05	7,24E-05	7,87E-05	9,04E-05	8,18809E-05	7,94247E-06	9,700	7,24E-05	7,29E-05	7,3E-05	6,05E-05	5,47E-05	6,6687E-05	8,53943E-06	12,805
P55264	ADK_MOUSE	Adenosine kinase	3,55E-05	3,09E-05	3,75E-05	3,89E-05	3,57111E-05	3,509E-06	9,826	3,57E-05	3,9E-05	3,64E-05	3,38E-05	3,04E-05	3,50641E-05	3,20851E-06	9,150
Q6IRU5	CLCB_MOUSE	Clathrin light chain B	0,00011	0,000118	0,000122	0,000124	0,000118301	6,33936E-06	5,359	0,000116	9,95E-05	0,000109	0,000109	9,03E-05	0,0001047	9,90166E-06	9,457
P00405	COX2_MOUSE	Cytochrome c oxidase subunit 2	9,82E-06	7,22E-06	7,64E-06	7,67E-06	8,0894E-06	1,17321E-06	14,503	8,57E-06	7,86E-06	8,57E-06	6,42E-06	4,75E-06	7,23598E-06	1,64037E-06	22,670
P62754	RS6_MOUSE	40S ribosomal protein S6	3,98E-05	3,97E-05	4,64E-05	4,86E-05	4,36418E-05	4,55089E-06	10,428	3,89E-05	4,46E-05	4,14E-05	3,6E-05	3,5E-05	3,91797E-05	3,94893E-06	10,079
P59325	IF5_MOUSE	Eukaryotic translation initiation factor 5	5,09E-05	5,42E-05	5,2E-05	4,87E-05	5,14254E-05	2,29057E-06	4,454	5,13E-05	3,71E-05	4,6E-05	4,77E-05	4,28E-05	4,49826E-05	5,34818E-06	11,889
Q91ZP9	NECA2_MOUSE	N-terminal EF-hand calcium-binding protein 2	3,65E-05	3,78E-05	3,6E-05	3,86E-05	3,72375E-05	1,20075E-06	3,225	3,64E-05	2,36E-05	3,25E-05	3,21E-05	3,57E-05	3,20773E-05	5,08659E-06	15,857
P67871	CSK2B_MOUSE	Casein kinase II subunit beta	4,39E-05	3,92E-05	4,37E-05	4,01E-05	4,17338E-05	2,39705E-06	5,744	4,68E-05	3,66E-05	3,66E-05	3,29E-05	2,48E-05	3,55326E-05	7,9355E-06	22,333
Q91Z61	DIRA1_MOUSE	GTP-binding protein Di-Ras1	3,02E-05	2,94E-05	2,72E-05	2,98E-05	2,91387E-05	1,35308E-06	4,644	3,12E-05	3,44E-05	3,09E-05	3,07E-05	2,6E-05	3,06512E-05	2,9943E-06	9,769
P84086	CPLX2_MOUSE	Complexin-2	2,93E-05	2,62E-05	2,79E-05	3,45E-05	2,94979E-05	3,57832E-06	12,131	2,65E-05	2,9E-05	3,34E-05	2,76E-05	3,22E-05	2,97239E-05	2,94776E-06	9,917
P61329	FGF12_MOUSE	Fibroblast growth factor 12	6,96E-05	6,68E-05	6,95E-05	6,96E-05	6,88996E-05	1,39362E-06	2,023	6,36E-05	5,76E-05	7E-05	6,43E-05	6,08E-05	6,32471E-05	4,60296E-06	7,278
Q9R1P1	PSB3_MOUSE	Proteasome subunit beta type-3	7,8E-05	7,77E-05	7,01E-05	7,7E-05	7,5666E-05	3,75157E-06	4,958	7,79E-05	9,55E-05	7,49E-05	7,23E-05	7,02E-05	7,81646E-05	1,00856E-05	12,903

Q8R0F8	FAHD1_MOUSE	Acylpyruvase FAHD1, mitochondrial	2,61E-05	2,77E-05	2,54E-05	2,4E-05	2,57918E-05	1,54925E-06	6,007	2,24E-05	2,46E-05	2,5E-05	2,34E-05	2,41E-05	2,38974E-05	1,03704E-06	4,340
P62900	RL31_MOUSE	60S ribosomal protein L31	8,36E-05	0,000103	0,0001	0,000103	9,74544E-05	9,35555E-06	9,600	8,73E-05	7,48E-05	8,33E-05	7,8E-05	7,05E-05	7,8764E-05	6,66498E-06	8,462
Q8R1I1	QCR9_MOUSE	Cytochrome b-c1 complex subunit 9	4,01E-05	4,47E-05	4,58E-05	3,61E-05	4,16892E-05	4,46754E-06	10,716	3,75E-05	3,95E-05	4,08E-05	4,58E-05	3,99E-05	4,07127E-05	3,08183E-06	7,570
Q8BSL7	ARF2_MOUSE	ADP-ribosylation factor 2	2,7E-05	3,36E-05	3E-05	2,89E-05	2,98929E-05	2,78644E-06	9,321	2,85E-05	1,3E-05	3,14E-05	2,89E-05	1,9E-05	2,41517E-05	7,82542E-06	32,401
Q8VDD5	MYH9_MOUSE	Myosin-9	3,72E-05	4,44E-05	3,83E-05	4,1E-05	4,02327E-05	3,22719E-06	8,021	3,48E-05	3,54E-05	3,55E-05	2,92E-05	3,71E-05	3,43877E-05	3,00861E-06	8,749
Q99KK7	DPP3_MOUSE	Dipeptidyl peptidase 3	8,52E-05	7,65E-05	8,44E-05	7,38E-05	7,99696E-05	5,66164E-06	7,080	7,57E-05	7,34E-05	4,06E-05	7,21E-05	6,98E-05	6,63176E-05	1,45581E-05	21,952
Q91WQ3	SYYC_MOUSE	Tyrosine--tRNA ligase, cytoplasmic	8,87E-05	9,14E-05	8,8E-05	8,98E-05	8,95033E-05	1,46504E-06	1,637	9,21E-05	8,76E-05	9,36E-05	8,67E-05	8,3E-05	8,86037E-05	4,31011E-06	4,864
Q9JHR7	IDE_MOUSE	Insulin-degrading enzyme	2,13E-05	1,62E-05	8,95E-06	1,58E-05	1,55813E-05	5,08154E-06	32,613	9,24E-06	1,15E-05	1,49E-05	1,21E-05	1,99E-05	1,35398E-05	4,0785E-06	30,122
Q8BRT1	CLAP2_MOUSE	CLIP-associating protein 2	0,000148	0,000147	0,000126	0,000136	0,000139343	1,01137E-05	7,258	0,000133	0,000134	0,000138	0,000129	0,000137	0,00013432	3,54275E-06	2,638
Q6R891	NEB2_MOUSE	Neurabin-2	3,68E-05	3,44E-05	3,81E-05	3,37E-05	3,57302E-05	2,07016E-06	5,794	3,49E-05	3,08E-05	3,52E-05	3,1E-05	3,89E-05	3,41593E-05	3,36596E-06	9,854
P50544	ACADV_MOUSE	Very long-chain specific acyl-CoA dehydrogenase, mitochondrial	2,6E-05	2,31E-05	2,57E-05	2,79E-05	2,56761E-05	1,94651E-06	7,581	2,18E-05	2,61E-05	2,7E-05	2,68E-05	2,4E-05	2,5143E-05	2,22397E-06	8,845
Q99NB1	ACS2L_MOUSE	Acetyl-coenzyme A synthetase 2-like, mitochondrial	1,49E-05	1,63E-05	1,5E-05	1,63E-05	1,56528E-05	7,85874E-07	5,021	1,43E-05	1,01E-05	1,49E-06	1,27E-05	1,28E-05	1,02721E-05	5,14384E-06	50,076
Q9WVJ2	PSD13_MOUSE	26S proteasome non-ATPase regulatory subunit 13	3,24E-05	2,61E-05	2,91E-05	2,74E-05	2,87327E-05	2,69496E-06	9,379	2,86E-05	2,56E-05	3,13E-05	2,35E-05	2,22E-05	2,62545E-05	3,73725E-06	14,235
Q9R1V7	ADA23_MOUSE	Disintegrin and metalloproteinase domain-containing protein 23	0,000106	0,000113	0,000101	9,82E-05	0,000104283	6,42318E-06	6,159	0,000115	0,000109	0,000108	0,000111	0,000106	0,000109672	3,56395E-06	3,250
O88487	DC1I2_MOUSE	Cytoplasmic dynein 1 intermediate chain 2	1,76E-05	1,79E-05	1,94E-05	1,76E-05	1,81452E-05	8,76352E-07	4,830	1,92E-05	1,66E-05	2,02E-05	1,32E-05	1,79E-05	1,74417E-05	2,72212E-06	15,607
Q99MN1	SYK_MOUSE	Lysine--tRNA ligase	3,33E-05	3,3E-05	3,31E-05	3,49E-05	3,35505E-05	8,8243E-07	2,630	3,9E-05	3,48E-05	3,79E-05	3,79E-05	4,22E-05	3,83735E-05	2,66182E-06	6,937
Q3TDK6	ROGDI_MOUSE	Protein rogdi homolog	6,49E-05	5,53E-05	5,85E-05	6,32E-05	6,04777E-05	4,3623E-06	7,213	5,54E-05	6,08E-05	5,22E-05	5,35E-05	4,83E-05	5,4026E-05	4,57904E-06	8,476
Q8R2R9	AP3M2_MOUSE	AP-3 complex subunit mu-2	2,57E-05	6,31E-05	7,96E-05	7,34E-05	6,04561E-05	2,41304E-05	39,914	6,88E-05	6,06E-05	5,49E-05	7,17E-05	6,98E-05	6,51689E-05	7,14643E-06	10,966
Q9CXS4	CENPV_MOUSE	Centromere protein V	5,51E-05	6,63E-05	7,04E-05	6,08E-05	6,31743E-05	6,63022E-06	10,495	5,78E-05	5,56E-05	6,44E-05	5,75E-05	6,05E-05	5,91487E-05	3,3874E-06	5,727
Q61024	ASNS_MOUSE	Asparagine synthetase [glutamine-hydrolyzing]	5,46E-05	3,63E-05	5,51E-05	6,11E-05	5,17613E-05	1,07535E-05	20,775	5,4E-05	4,48E-05	6,23E-05	6,31E-05	5,81E-05	5,64444E-05	7,46673E-06	13,228
Q91Z31	PTBP2_MOUSE	Polypyrimidine tract-binding protein 2	4,26E-05	3,81E-05	3,1E-05	5,06E-05	4,05584E-05	8,21228E-06	20,248	3,98E-05	2,95E-05	2,85E-05	3,7E-05	3,26E-05	3,34903E-05	4,84683E-06	14,472
Q9DBS2	TPRGL_MOUSE	Tumor protein p63-regulated gene 1-like protein	3,86E-05	3,97E-05	4,03E-05	4,59E-05	4,11221E-05	3,24289E-06	7,886	3,29E-05	3,29E-05	3,17E-05	3,26E-05	3,29E-05	3,25936E-05	5,06848E-07	1,555
P60521	GBRL2_MOUSE	Gamma-aminobutyric acid receptor-associated protein-like 2	4,22E-05	4,97E-05	5,35E-05	4,59E-05	4,78401E-05	4,86576E-06	10,171	4,28E-05	3,5E-05	4,71E-05	4,28E-05	4,01E-05	4,15515E-05	4,46115E-06	10,736
Q811I0	ATPF1_MOUSE	ATP synthase mitochondrial F1 complex assembly factor 1	2,46E-05	2,1E-05	2,32E-05	2,51E-05	2,34606E-05	1,86061E-06	7,931	2,61E-05	2,28E-06	2,54E-05	2,46E-05	2,05E-05	1,97785E-05	1,00194E-05	50,658
P61087	UBE2K_MOUSE	Ubiquitin-conjugating enzyme E2 K	0,000117	0,000122	0,000122	0,000129	0,000122324	4,62681E-06	3,782	0,000101	0,000109	0,000115	8,64E-05	8,3E-05	9,89311E-05	1,38754E-05	14,025
Q9JHU9	INO1_MOUSE	Inositol-3-phosphate synthase 1	4,43E-05	5,45E-05	6,4E-05	6,5E-05	5,69752E-05	9,6533E-06	16,943	4,21E-05	1,25E-06	4,99E-05	5,58E-05	5,39E-05	4,05897E-05	2,26113E-05	55,707
Q61151	2A5E_MOUSE	Serine/threonine-protein phosphatase 2A 56 kDa regulatory subunit epsilon isoform	7,46E-05	6,02E-05	6,39E-05	8,68E-05	7,13749E-05	1,19353E-05	16,722	7,33E-05	8,59E-05	6,78E-05	8,57E-05	6,76E-05	7,60514E-05	9,20471E-06	12,103
Q9ERI6	RDH14_MOUSE	Retinol dehydrogenase 14	2,21E-05	1,93E-05	2,02E-05	2,22E-05	2,09497E-05	1,44121E-06	6,879	2,01E-05	2,3E-05	1,65E-05	1,65E-05	1,87E-05	1,89732E-05	2,71472E-06	14,308
Q3THG9	AASD1_MOUSE	Alanyl-tRNA editing protein Aarsd1	3,07E-05	2,97E-05	2,43E-05	3,33E-05	2,95125E-05	3,76616E-06	12,761	3,02E-05	3,23E-05	2,95E-05	3,22E-05	2,55E-05	2,99174E-05	2,76765E-06	9,251
Q6P8X1	SNX6_MOUSE	Sorting nexin-6	6,76E-05	7,59E-05	6,74E-05	6,91E-05	6,9999E-05	4,01237E-06	5,732	6,74E-05	7,05E-05	7,42E-05	6,65E-05	7,14E-05	6,99873E-05	3,12212E-06	4,461
Q8BP92	RCN2_MOUSE	Reticulocalbin-2	3,77E-05	3,95E-05	4,26E-05	3,84E-05	3,95534E-05	2,17552E-06	5,500	3,28E-05	4,01E-05	3,57E-05	3,15E-05	3,46E-05	3,49146E-05	3,29436E-06	9,435
Q9CZM2	RL15_MOUSE	60S ribosomal protein L15	2,88E-05	4,57E-05	4,26E-05	5,2E-05	4,22675E-05	9,81981E-06	23,233	3,6E-05	3,91E-05	4,35E-05	3,15E-05	3,34E-05	3,66806E-05	4,74043E-06	12,924
P63323	RS12_MOUSE	40S ribosomal protein S12	6,03E-05	7,07E-05	7,52E-05	6,89E-05	6,87459E-05	6,25266E-06	9,095	5,97E-05	6,39E-05	6,86E-05	5,91E-05	5,37E-05	6,10034E-05	5,60578E-06	9,189
Q9JI46	NUDT3_MOUSE	Diphosphoinositol polyphosphate phosphohydrolase 1	2,52E-05	2,91E-05	4,35E-05	2,98E-05	3,18904E-05	7,98664E-06	25,044	2,17E-05	1,71E-05	2,33E-05	2,71E-05	2,29E-05	2,23961E-05	3,60142E-06	16,081
P62748	HPCL1_MOUSE	Hippocalcin-like protein 1	4,01E-05	5,64E-05	5,91E-05	6,12E-05	5,4215E-05	9,60493E-06	17,716	4,36E-05	4,84E-05	4,68E-05	4,62E-05	4,97E-05	4,69324E-05	2,29677E-06	4,894
Q11136	PEPD_MOUSE	Xaa-Pro dipeptidase	3,11E-05	2,79E-05	3,65E-05	4,12E-05	3,41608E-05	5,88809E-06	17,236	2,99E-05	2,07E-05	2,75E-05	2,57E-05	2,74E-05	2,62418E-05	3,44876E-06	13,142
Q9D0M1	KPRA_MOUSE	Phosphoribosyl pyrophosphate synthase-associated protein 1	3,25E-05	3E-05	3,16E-05	3,86E-05	3,31519E-05	3,74127E-06	11,285	2,98E-05	2,68E-05	2,78E-05	2,87E-05	2,6E-05	2,78082E-05	1,52066E-06	5,468
Q3TYX3	SMYD5_MOUSE	SET and MYND domain-containing protein 5	2,68E-05	2,66E-05	3,13E-05	2,78E-05	2,81153E-05	2,17315E-06	7,729	2,84E-05	2,8E-05	2,58E-05	2,7E-05	2,42E-05	2,66831E-05	1,73469E-06	6,501
Q8BHF7	PGPS1_MOUSE	CDP-diacylglycerol--glycerol-3-phosphate 3-phosphatidyltransferase, mitochondrial	2,17E-05	2,22E-05	1,85E-05	2,21E-05	2,11147E-05	1,72863E-06	8,187	2,15E-05	2,19E-05	2,56E-05	2,97E-05	2,5E-05	2,47485E-05	3,32167E-06	13,422
P54830	PTN5_MOUSE	Tyrosine-protein phosphatase non-receptor type 5	4,54E-05	4,51E-05	4,64E-05	3,43E-05	4,28209E-05	5,70314E-06	13,319	4,44E-05	4,3E-05	4,18E-05	5,83E-05	6,85E-05	5,12159E-05	1,17526E-05	22,947
Q9Z0L0	TPBG_MOUSE	Trophoblast glycoprotein	2,53E-05	2,31E-05	2,55E-05	2,04E-05	2,3572E-05	2,3827E-06	10,108	2,31E-05	1,83E-05	1,78E-05	2,83E-05	2,72E-05	2,2944E-05	4,88827E-06	21,305
P48678	LMNA_MOUSE	Prelamin-A/C	3,45E-05	3,46E-05	3,71E-05	3,41E-05	3,50628E-05	1,34768E-06	3,844	3,26E-05	3,51E-05	4,35E-05	3,6E-05	3,22E-05	3,58828E-05	4,52371E-06	12,607
Q5SSL4	ABR_MOUSE	Active breakpoint cluster region-related protein	0,000103	0,00013	0,000122	0,000131	0,000121447	1,31371E-05	10,817	0,000111	0,000112	0,000122	0,000119	0,000115	0,000115825	4,32712E-06	3,736
Q5FWK3	RHG01_MOUSE	Rho GTPase-activating protein 1	5,31E-05	4,33E-05	4,04E-05	4,33E-05	4,50242E-05	5,56518E-06	12,360	4,7E-05	4,45E-05	4,57E-05	5,12E-05	4,67E-05	4,70279E-05	2,55891E-06	5,441
Q60631	GRB2_MOUSE	Growth factor receptor-bound protein 2	1,9E-05	1,97E-05	1,72E-05	2,01E-05	1,89857E-05	1,30109E-06	6,853	2,2E-05	2,07E-05	2,21E-05	2,08E-05	2,01E-05	2,11483E-05	8,53035E-07	4,034
O35678	MGLL_MOUSE	Monoglyceride lipase	2,53E-05	3,19E-05	4,86E-06	3,26E-05	2,36723E-05	1,29578E-05	54,738	3,86E-05	3,54E-05	4,4E-05	3,6E-05	3,37E-05	3,75487E-05	3,99122E-06	10,629
Q9WUK2	IF4H_MOUSE	Eukaryotic translation initiation factor 4H	3,98E-05	4,25E-05	3,05E-05	2,92E-05	3,55063E-05	6,65848E-06	18,753	3,85E-05	4,69E-05	4,03E-05	4,23E-05	5,95E-05	4,54893E-05	8,42272E-06	18,516
Q9Z110	P5CS_MOUSE	Delta-1-pyrroline-5-carboxylate synthase	1,89E-05	2,2E-05	2,05E-05	1,9E-05	2,00962E-05	1,46454E-06	7,288	2,18E-05	2,28E-05	2,11E-05	1,96E-05	2,01E-05	2,10952E-05	1,26161E-06	5,981
Q8K274	KT3K_MOUSE	Ketosamine-3-kinase	4,55E-05	4,06E-05	3,96E-05	5,01E-05	4,39674E-05	4,83179E-06	10,989	3,66E-05	4,17E-05	4,07E-05	3,31E-05	3,19E-05	3,67931E-05	4,39464E-06	11,944
Q62446	FKBP3_MOUSE	Peptidyl-prolyl cis-trans isomerase FKBP3	1,29E-05	1,23E-05	1,13E-05	1,16E-05	1,20425E-05	6,98432E-07	5,800	1,56E-05	1,2E-05	1,42E-05	1,25E-05	1,02E-05	1,29159E-05	2,09093E-06	16,189
Q8C5H8	NAKD2_MOUSE	NAD kinase 2, mitochondrial	2,1E-05	2,31E-05	1,82E-05	2,11E-05	2,08427E-05	2,02074E-06	9,695	1,71E-05	1,64E-05	1,91E-05	1,67E-05	1,74E-05	1,73436E-05	1,04076E-06	6,001
Q8VE70	PDC10_MOUSE	Programmed cell death protein 10	3,3E-05	3,54E-05	3,62E-05	3,65E-05	3,52569E-05	1,6031E-06	4,547	3,45E-05	3,44E-05	3,5E-05	3,85E-05	3,31E-05	3,51054E-05	1,99688E-06	5,688
Q9QYA2	TOM40_MOUSE	Mitochondrial import receptor subunit TOM40 homolog	6,46E-05	6,91E-05	6,88E-05	5,95E-05	6,55111E-05	4,49049E-06	6,855	6,82E-05	6E-05	6,89E-05	5,99E-05	2,82E-05	5,70282E-05	1,67038E-05	29,290
Q8R127	SCPDL_MOUSE	Saccharopine dehydrogenase-like oxidoreductase	7,22E-06	9E-06	4,55E-06	5,64E-06	6,60096E-06	1,93833E-06	29,364	8,41E-06	8,52E-06	7,81E-06	8,6E-06	7,06E-06	8,07979E-06	6,51705E-07	8,066
Q8BWY3	ERF1_MOUSE	Eukaryotic peptide chain release factor subunit 1	3,58E-05	3,58E-05	4,77E-05	4,49E-05	4,10496E-05	6,17568E-06	15,044	4,58E-05	4,33E-05	5,25E-05	4,75E-05	5,18E-05	4,81743E-05	3,94617E-06	8,191
Q60865	CAPR1_MOUSE	Caprin-1	2,91E-05	1,01E-05	3,73E-05	2,52E-05	2,54088E-05	1,13966E-05	44,853	3,87E-05	3,39E-05	4,19E-05	4,75E-05	4,52E-05	4,14475E-05	5,36394E-06	12,942
Q9Z130	HNRDL_MOUSE	Heterogeneous nuclear ribonucleoprotein D-like	3,3E-05	4,19E-05	4,31E-05	4,77E-05	4,14414E-05	6,14025E-06	14,817	4,48E-05	3,29E-05	4,18E-05	4,43E-05	3,99E-05	4,07336E-05	4,80076E-06	11,786
P56395	CYB5_MOUSE	Cytochrome b5	1,26E-06	2,67E-05	2,87E-05	2,9E-05	2,14042E-05	1,347E-05	62,932	1,97E-05	3,12E-05	2,99E-05	2,39E-05	2,88E-05	2,66963E-05	4,75693E-06	17,819
P61971	NTF2_MOUSE	Nuclear transport factor 2	1,68E-05	1,92E-05	1,98E-05	1,83E-05	1,8524E-05	1,32244E-06	7,139	1,59E-05	2,03E-05	2,11E-05	1,75E-05	1,35E-05	1,76483E-05	3,13072E-06	17,739
O55026	ENTP2_MOUSE	Ectonucleoside triphosphate diphosphohydrolase 2	1,51E-05	1,76E-05	1,94E-05	1,99E-05	1,80226E-05	2,18254E-06	12,110	1,3E-05	1,15E-05	1,71E-05	1,8E-05	1,85E-05	1,56188E-05	3,15858E-06	20,223
Q8BGU5	CCNY_MOUSE	Cyclin-Y	3,08E-05	3,17E-05	3,43E-05	3,49E-05	3,29532E-05	1,9689E-06	5,975	2,83E-05	2,3E-05	3E-05	2,77E-05	2,67E-05	2,71532E-05	2,60158E-06	9,581
Q7TMS5	ABCG2_MOUSE	ATP-binding cassette sub-family G member 2	2,64E-05	1,01E-06	1,73E-05	1,94E-05	1,60338E-05	1,07475E-05	67,030	2,61E-05	2,12E-05	2,04E-05	1,89E-05	1,96E-05	2,12559E-05	2,84016E-06	13,362
P62305	RUXE_MOUSE	Small nuclear ribonucleoprotein E	2,99E-05	4,51E-05	5,16E-05	4,04E-05	4,17654E-05	9,16694E-06	21,949	3,39E-05	3,48E-05	4,58E-05	4,63E-05	4,81E-05	4,17783E-05	6,82567E-06	16,338
Q5SQX6	CYFP2_MOUSE	Cytoplasmic FMR1-interacting protein 2	0,000263	0,000248	0,000271	0,000247	0,000257024	1,15287E-05	4,485	0,000268	0,000263	0,000245	0,000237	0,000272	0,000256727	1,52694E-05	5,948
Q5DTL9	S4A10_MOUSE	Sodium-driven chloride bicarbonate exchanger	2,78E-05	2,78E-05	2,97E-05	2,84E-05	2,84101E-05	8,94707E-07	3,149	2,97E-05	2,34E-05	2,7E-05	2,38E-05	3,21E-05	2,71767E-05	3,73118E-06	13,729
Q99J99	THTM_MOUSE	3-mercaptopyruvate sulfurtransferase	3,93E-05	3,8E-05	3,94E-05	3,92E-05	3,89848E-05	6,40723E-07	1,644	4,07E-05	3,26E-05	3,22E-05	3,55E-05	3,14E-05	3,44898E-05	3,83266E-06	11,112
P99028	QCR6_MOUSE	Cytochrome b-c1 complex subunit 6, mitochondrial	5,86E-05	5,18E-05	6,46E-05	5,74E-05	5,81038E-05	5,25597E-06	9,046	2,64E-05	3,85E-05	3,27E-05	3,73E-05	4,72E-05	3,64018E-05	7,68206E-06	21,103
P53612	PGTB2_MOUSE	Geranylgeranyl transferase type-2 subunit beta	1,56E-05	1,49E-05	1,33E-05	1,83E-05	1,55233E-05	2,08355E-06	13,422	1,21E-05	1,28E-05	1,06E-05	1,02E-05	7,36E-06	1,06063E-05	2,10254E-06	19,823
Q3UHB8	CC177_MOUSE	Coiled-coil domain-containing protein 177	2,45E-05	2,57E-05	2,58E-05	2,38E-05	2,49219E-05	9,81282E-07	3,937	2,55E-05	2,23E-05	2,07E-05	1,92E-05	2,34E-05	2,22205E-05	2,42271E-06	10,903
P56375	ACYP2_MOUSE	Acylphosphatase-2	7,71E-06	9,36E-06	1,07E-05	7,65E-06	8,8476E-06	1,45415E-06	16,436	7,48E-06	7,57E-06	9,3E-06	7,6E-06	8,29E-06	8,04742E-06	7,71687E-07	9,589
Q9CR67	TMM33_MOUSE	Transmembrane protein 33	4,4E-05	5,29E-05	4,96E-05	4,68E-05	4,83362E-05	3,80888E-06	7,880	4,66E-05	4,57E-05	4,42E-05	4,18E-05	3,99E-05	4,36457E-05	2,75044E-06	6,302

Q9WTR5	CAD13_MOUSE	Cadherin-13	6,47E-05	7,22E-05	7,77E-05	6,96E-05	7,1055E-05	5,44731E-06	7,666	8,31E-05	6,81E-05	6,88E-05	7,15E-05	6,62E-05	7,15415E-05	6,71556E-06	9,387
P53986	MOT1_MOUSE	Monocarboxylate transporter 1	2,47E-05	2,47E-05	2,13E-05	2,17E-05	2,30912E-05	1,86145E-06	8,061	2,53E-05	2,67E-05	2,56E-05	2,04E-05	2,33E-05	2,42601E-05	2,48087E-06	10,226
Q3UJU9	RMD3_MOUSE	Regulator of microtubule dynamics protein 3	4,76E-05	4,93E-05	4,75E-05	4,13E-05	4,63998E-05	3,52599E-06	7,599	4,13E-05	4,44E-05	3,93E-05	4,25E-05	4,26E-05	4,20317E-05	1,88448E-06	4,483
P39054	DYN2_MOUSE	Dynamin-2	1,6E-05	1,93E-05	1,63E-05	1,91E-05	1,7667E-05	1,77212E-06	10,031	2,39E-05	2,08E-05	2,16E-05	2,1E-05	9,85E-07	1,76561E-05	9,3975E-06	53,225
Q8R2Y0	ABHD6_MOUSE	Monoacylglycerol lipase ABHD6	1,55E-05	1,29E-05	1,61E-05	1,49E-05	1,4825E-05	1,3812E-06	9,317	1,72E-05	1,36E-05	1,2E-05	1,28E-05	1,53E-05	1,41693E-05	2,07477E-06	14,643
P30416	FKBP4_MOUSE	Peptidyl-prolyl cis-trans isomerase FKBP4	4,23E-05	4,43E-05	4,01E-05	3,86E-05	4,13204E-05	2,48346E-06	6,010	4,08E-05	4,25E-05	4,03E-05	3,84E-05	3,34E-05	3,90615E-05	3,50058E-06	8,962
P46664	PURA2_MOUSE	Adenylosuccinate synthetase isozyme 2	6,65E-05	7,21E-05	7,43E-05	6,86E-05	7,03873E-05	3,50141E-06	4,974	6,11E-05	5,9E-05	6,33E-05	6,52E-05	6,53E-05	6,27823E-05	2,6892E-06	4,283
Q9CY64	BIEA_MOUSE	Biliverdin reductase A	6,77E-05	5,38E-05	5,05E-05	6,86E-05	6,01481E-05	9,37466E-06	15,586	6,43E-05	5,88E-05	7,02E-05	7,17E-05	5,94E-05	6,48982E-05	5,94878E-06	9,166
Q6P9K8	CSKI1_MOUSE	Caskin-1	6,33E-05	7,08E-05	6,58E-05	7,14E-05	6,78399E-05	3,91059E-06	5,764	7,67E-05	6,9E-05	6,56E-05	5,99E-05	6,97E-05	6,81606E-05	6,1236E-06	8,984
Q8R001	MARE2_MOUSE	Microtubule-associated protein RP/EB family member 2	7,75E-05	9,61E-05	7,67E-05	9,4E-05	8,60664E-05	1,04348E-05	12,124	0,000101	7,41E-05	8,55E-05	8,31E-05	7,71E-05	8,41083E-05	1,03097E-05	12,258
A2A432	CUL4B_MOUSE	Cullin-4B	6,52E-05	1,12E-06	5,93E-05	5,64E-05	4,55094E-05	2,98202E-05	65,525	5,34E-05	3,99E-05	4,88E-05	3,84E-05	5,18E-05	4,64687E-05	6,88223E-06	14,810
P35505	FAAA_MOUSE	Fumarylacetoacetase	0,000116	0,000109	9,6E-05	0,000106	0,000106762	8,14164E-06	7,626	0,000113	4,96E-05	0,000101	8,87E-05	9,26E-05	8,89252E-05	2,39055E-05	26,883
Q02357	ANK1_MOUSE	Ankyrin-1	2,16E-05	2,17E-05	1,83E-05	2,1E-05	2,06723E-05	1,59564E-06	7,719	1,92E-05	2,06E-05	1,74E-05	1,57E-05	1,82E-05	1,8211E-05	1,85205E-06	10,170
Q64674	SPEE_MOUSE	Spermidine synthase	1,07E-05	9,38E-06	7,89E-06	9,18E-06	9,29115E-06	1,15918E-06	12,476	1,14E-05	9,64E-06	9,85E-06	1,24E-05	9,65E-06	1,0596E-05	1,26184E-06	11,909
P70372	ELAV1_MOUSE	ELAV-like protein 1	7,75E-05	8,85E-05	9,15E-05	9,43E-05	8,79581E-05	7,34794E-06	8,354	8,4E-05	7,46E-05	7,85E-05	7,51E-05	7,57E-05	7,75688E-05	3,86885E-06	4,988
Q5XJY5	COPD_MOUSE	Coatomer subunit delta	5,32E-05	5,48E-05	5,78E-05	5,5E-05	5,52204E-05	1,92964E-06	3,494	5,86E-05	4,64E-05	5,35E-05	5,8E-05	5,21E-05	5,37189E-05	4,93357E-06	9,184
P20060	HEXB_MOUSE	Beta-hexosaminidase subunit beta	4,55E-05	4,45E-05	4,61E-05	4,55E-05	4,54148E-05	6,7079E-07	1,477	4,53E-05	4,77E-05	4,35E-05	4,5E-05	4,11E-05	4,451E-05	2,41166E-06	5,418
Q8K3E5	AHI1_MOUSE	Jouberin	1,37E-05	1,79E-05	1,62E-05	1,67E-05	1,61205E-05	1,75555E-06	10,890	1,23E-05	1,93E-05	1,76E-05	1,32E-05	1,42E-05	1,53167E-05	2,98853E-06	19,512
Q9CZ42	NNRD_MOUSE	ATP-dependent (S)-NAD(P)H-hydrate dehydratase	3,53E-05	5,27E-05	6,29E-05	5,94E-05	5,25785E-05	1,22543E-05	23,307	5,93E-05	5,34E-05	5,67E-05	5,75E-05	5,75E-05	5,68855E-05	2,13484E-06	3,753
Q9DC07	LNEBL_MOUSE	LIM zinc-binding domain-containing Nebulette	9,56E-06	1,26E-05	8,96E-06	1,15E-05	1,06549E-05	1,68413E-06	15,806	8,9E-06	1,19E-05	1,12E-05	8,92E-06	7,24E-06	9,62624E-06	1,87129E-06	19,439
P54822	PUR8_MOUSE	Adenylosuccinate lyase	4,77E-05	4,74E-05	4,81E-05	4,6E-05	4,72891E-05	8,72617E-07	1,845	4,63E-05	4,7E-05	5,02E-05	4,89E-05	4,29E-05	4,70746E-05	2,77176E-06	5,888
Q9CY58	PAIRB_MOUSE	Plasminogen activator inhibitor 1 RNA-binding protein	3,96E-06	4,14E-06	3,09E-06	4,45E-06	3,91023E-06	5,81924E-07	14,882	5,59E-06	4,51E-06	4,38E-06	4,21E-06	4,2E-06	4,57833E-06	5,80337E-07	12,676
Q8BX94	OSBL2_MOUSE	Oxysterol-binding protein-related protein 2	1,78E-05	4,24E-05	8,4E-05	2,12E-05	4,13296E-05	3,04277E-05	73,622	2,75E-05	1,84E-05	2,15E-05	3,3E-05	5,89E-05	3,18457E-05	1,61319E-05	50,657
P62317	SMD2_MOUSE	Small nuclear ribonucleoprotein Sm D2	3,3E-05	3,97E-05	4,61E-05	4,3E-05	4,04547E-05	5,62382E-06	13,902	3,59E-05	3,25E-05	3,82E-05	2,93E-05	3,13E-05	3,34437E-05	3,58361E-06	10,715
Q8R191	SNG3_MOUSE	Synaptogyrin-3	6,96E-05	5,72E-05	6,6E-05	4,15E-05	5,8588E-05	1,25213E-05	21,372	5,75E-05	7,14E-05	5,91E-05	4,75E-05	4,06E-05	5,52237E-05	1,17687E-05	21,311
Q7TN79	AKA7G_MOUSE	A-kinase anchor protein 7 isoform gamma	1,59E-05	2,02E-05	1,74E-05	1,25E-05	1,64862E-05	3,21067E-06	19,475	1,44E-05	1,68E-05	1,71E-05	1,28E-05	1,56E-05	1,53322E-05	1,76982E-06	11,543
P00397	COX1_MOUSE	Cytochrome c oxidase subunit 1	7,45E-05	5,91E-05	5,31E-05	4,88E-05	5,89011E-05	1,12405E-05	19,084	4,82E-05	3,18E-05	3,88E-05	3,81E-05	4,72E-05	4,08112E-05	6,85707E-06	16,802
Q8C5W0	CLMN_MOUSE	Calmin	2,15E-05	1,77E-05	1,49E-05	2,09E-05	1,87688E-05	3,07428E-06	16,380	2,05E-05	1,66E-05	1,8E-05	2,02E-05	1,67E-05	1,83791E-05	1,84718E-06	10,050
Q9CQ89	CUTA_MOUSE	Protein CutA	6,07E-05	6,57E-05	8,71E-05	6,9E-05	7,06333E-05	1,15055E-05	16,289	3,99E-05	4,28E-06	5,46E-05	5,21E-05	5,06E-05	4,02957E-05	2,08991E-05	51,864
P56379	68MP_MOUSE	#N/A	3,72E-05	4,3E-05	5,91E-05	3,66E-05	4,39819E-05	1,04816E-05	23,832	3,82E-05	4,93E-05	5,79E-05	5,63E-05	5,57E-05	5,14832E-05	8,11997E-06	15,772
Q9Z0H8	CLIP2_MOUSE	CAP-Gly domain-containing linker protein 2	1,01E-05	9,35E-06	1,11E-05	1,03E-05	1,02098E-05	7,05933E-07	6,914	1,05E-05	1,11E-05	1,06E-05	2,03E-06	8,77E-06	8,61091E-06	3,78599E-06	43,967
P55066	NCAN_MOUSE	Neurocan core protein	2,35E-05	2,27E-05	3,35E-05	3,5E-05	2,86651E-05	6,47711E-06	22,596	2,24E-05	2,02E-05	2E-05	1,57E-05	3,11E-05	2,18973E-05	5,69714E-06	26,018
P16054	KPCE_MOUSE	Protein kinase C epsilon type	0,000135	0,000115	0,000133	0,000142	0,000130938	1,14941E-05	8,778	0,000134	0,000102	0,000138	0,000129	0,000146	0,000129854	1,65713E-05	12,761
Q61704	ITIH3_MOUSE	Inter-alpha-trypsin inhibitor heavy chain H3	3,02E-05	3,44E-05	2,44E-05	2,66E-05	2,89017E-05	4,39912E-06	15,221	2,42E-05	2,87E-05	2,3E-05	2,73E-05	2,76E-05	2,61512E-05	2,41159E-06	9,222
Q9Z0P5	TWF2_MOUSE	Twinfilin-2	5,22E-05	5,83E-05	5,57E-05	5,65E-05	5,56505E-05	2,56985E-06	4,618	8,16E-06	6,22E-05	5,3E-05	5,34E-05	5,82E-05	4,69984E-05	2,20397E-05	46,895
Q99LI8	HGS_MOUSE	Hepatocyte growth factor-regulated tyrosine kinase substrate	6,81E-05	5,22E-05	5,33E-05	6,79E-05	6,03579E-05	8,78687E-06	14,558	6,44E-05	6,14E-05	5,18E-05	4,26E-05	6,66E-05	5,73518E-05	1,00202E-05	17,471
Q63912	OMGP_MOUSE	Oligodendrocyte-myelin glycoprotein	5,37E-05	5,22E-05	6,03E-05	6,47E-05	5,77409E-05	5,8391E-06	10,113	4,58E-05	3,55E-05	5,28E-05	5,33E-05	6,4E-05	5,02733E-05	1,05204E-05	20,926
Q6X893	CTL1_MOUSE	Choline transporter-like protein 1	6,78E-05	7,09E-05	6,23E-05	6,52E-05	6,65399E-05	3,66068E-06	5,501	6,79E-05	7,23E-05	7,79E-05	7,49E-05	6,93E-05	7,24494E-05	4,05146E-06	5,592
P61222	ABCE1_MOUSE	ATP-binding cassette sub-family E member 1	2,9E-05	3,72E-05	3,74E-05	3,63E-05	3,49734E-05	4,01171E-06	11,471	2,93E-05	3,13E-05	3,02E-05	3,42E-05	3,1E-05	3,1174E-05	1,84991E-06	5,934
Q8BXZ1	TMX3_MOUSE	Protein disulfide-isomerase TMX3	5,95E-05	6,28E-05	5,2E-05	5,89E-05	5,82882E-05	4,55862E-06	7,821	5,41E-05	3,35E-05	6,12E-05	5,62E-05	4,66E-05	5,03229E-05	1,0803E-05	21,467
Q03517	SCG2_MOUSE	Secretogranin-2	2,15E-05	2,61E-05	1,3E-06	2,4E-05	1,82218E-05	1,14343E-05	62,751	1,67E-05	1,96E-05	2,34E-05	2,34E-05	2,51E-05	2,1644E-05	3,39729E-06	15,696
Q8K221	ARFP2_MOUSE	Arfaptin-2	5,42E-05	5,29E-05	5,62E-05	5,37E-05	5,42532E-05	1,40331E-06	2,587	5,66E-05	4,05E-05	5,19E-05	4,57E-05	5,61E-05	5,0143E-05	6,94139E-06	13,843
Q6PGN3	DCLK2_MOUSE	Serine/threonine-protein kinase DCLK2	2,67E-05	3,07E-05	1,67E-05	2,52E-05	2,48112E-05	5,89364E-06	23,754	3,04E-05	2,8E-05	2,77E-05	2,57E-05	2,93E-05	2,82279E-05	1,78663E-06	6,329
Q8C854	MYEF2_MOUSE	Myelin expression factor 2	2,43E-05	3,18E-05	3,07E-05	2,93E-05	2,90008E-05	3,2875E-06	11,336	3,81E-05	2,61E-05	2,92E-05	3,82E-05	3,53E-05	3,33783E-05	5,47488E-06	16,403
F8VPU2	FARP1_MOUSE	FERM, RhoGEF and pleckstrin domain-containing protein 1	3,17E-05	3,52E-05	3,49E-05	3,09E-05	3,31834E-05	2,18642E-06	6,589	3,42E-05	3,64E-05	3,34E-05	2,89E-05	3,13E-05	3,28482E-05	2,86673E-06	8,727
Q8BTX9	HSDL1_MOUSE	Inactive hydroxysteroid dehydrogenase-like protein 1	3,39E-05	4,12E-05	3,8E-05	4,37E-05	3,92315E-05	4,22463E-06	10,768	3,79E-05	3,71E-05	4,02E-05	3,88E-05	2,76E-05	3,63134E-05	4,99972E-06	13,768
P14106	C1QB_MOUSE	Complement C1q subcomponent subunit B	1,27E-05	1,34E-05	1,26E-05	1,23E-05	1,27616E-05	4,34238E-07	3,403	1,02E-05	1,13E-05	1,2E-05	9,69E-06	1,19E-05	1,10186E-05	1,04466E-06	9,481
Q2M3X8	PHAR1_MOUSE	Phosphatase and actin regulator 1	7,86E-05	7,79E-05	8,82E-05	7,73E-05	8,05305E-05	5,14437E-06	6,388	4,59E-05	7,38E-05	7,92E-05	9,4E-05	9,27E-05	7,71275E-05	1,95051E-05	25,289
Q60870	REEP5_MOUSE	Receptor expression-enhancing protein 5	1,09E-05	1,02E-05	7,19E-06	1,08E-05	9,75332E-06	1,73643E-06	17,803	1,15E-05	1,52E-05	1,24E-05	9,39E-06	8,45E-06	1,13908E-05	2,65887E-06	23,342
Q69ZS6	SV2C_MOUSE	Synaptic vesicle glycoprotein 2C	3,36E-05	4,01E-05	2,75E-05	3,16E-05	3,32039E-05	5,25718E-06	15,833	3,97E-05	4,04E-05	3,9E-05	3,76E-05	3,42E-05	3,81889E-05	2,43507E-06	6,376
Q9CT10	RANB3_MOUSE	Ran-binding protein 3	6,05E-06	4,02E-06	4,76E-06	4,65E-06	4,8714E-06	8,54438E-07	17,540	5,39E-06	2,76E-06	5,06E-06	4,71E-06	4,17E-06	4,4154E-06	1,0291E-06	23,307
Q9ET22	DPP2_MOUSE	Dipeptidyl peptidase 2	1,9E-05	2,13E-05	1,51E-05	2,1E-05	1,90914E-05	2,87657E-06	15,067	1,84E-05	1,93E-05	1,65E-05	1,72E-05	1,93E-05	1,81555E-05	1,24699E-06	6,868
Q9Z239	PLM_MOUSE	Phospholemman	2,38E-05	2,81E-05	2,56E-05	1,79E-06	1,98139E-05	1,21404E-05	61,272	2,34E-05	3,68E-05	3,23E-05	2,31E-05	2,59E-05	2,82843E-05	6,01041E-06	21,250
Q9JKR6	HYOU1_MOUSE	Hypoxia up-regulated protein 1	4,28E-05	4,87E-05	4,96E-05	5,88E-05	4,9968E-05	6,60913E-06	13,227	4,7E-05	3,39E-05	4,76E-05	4,92E-05	5,3E-05	4,6127E-05	7,25014E-06	15,718
Q8BWT5	DIP2A_MOUSE	Disco-interacting protein 2 homolog A	2,11E-05	2,65E-05	2,32E-05	2,42E-05	2,37565E-05	2,23904E-06	9,425	3,27E-05	2,42E-05	2,13E-05	2,97E-05	2,26E-05	2,61004E-05	4,8693E-06	18,656
Q9CXW4	RL11_MOUSE	60S ribosomal protein L11	6,86E-05	7,83E-05	8,88E-05	7,44E-05	7,75267E-05	8,52132E-06	10,991	7,8E-05	6,95E-05	7,58E-05	6,73E-05	9,96E-06	6,01156E-05	2,83799E-05	47,209
P35762	CD81_MOUSE	CD81 antigen	7,24E-05	7,17E-05	0,0001	0,000105	8,74457E-05	1,7877E-05	20,444	1,12E-05	0,000107	7,87E-05	7,22E-05	0,000102	7,431E-05	3,8334E-05	51,587
O35382	EXOC4_MOUSE	Exocyst complex component 4	1,34E-05	1,49E-05	1,62E-05	1,48E-05	1,48261E-05	1,16392E-06	7,850	1,65E-05	1,09E-05	1,34E-05	1,32E-05	1,48E-05	1,37644E-05	2,08669E-06	15,160
Q8R010	AIMP2_MOUSE	Aminoacyl tRNA synthase complex-interacting multifunctional protein 2	3,27E-05	3,34E-05	3,39E-05	3,55E-05	3,38782E-05	1,18663E-06	3,503	3,04E-05	2,6E-05	2,6E-05	2,7E-05	2,71E-05	2,73081E-05	1,81553E-06	6,648
Q8VCT3	AMPB_MOUSE	Aminopeptidase B	1,16E-05	1,09E-05	1,02E-05	1,32E-05	1,14785E-05	1,30953E-06	11,409	1,39E-05	4,43E-07	1,14E-05	1,44E-05	1,21E-05	1,04437E-05	5,72232E-06	54,792
Q9EPK7	XPO7_MOUSE	Exportin-7	2,32E-05	2,07E-05	2,12E-05	2E-05	2,12833E-05	1,38805E-06	6,522	1,92E-05	1,88E-05	1,78E-05	1,77E-05	1,98E-05	1,86683E-05	8,94223E-07	4,790
Q9QUM9	PSA6_MOUSE	Proteasome subunit alpha type-6	2,84E-05	3,08E-05	3,49E-05	3,46E-05	3,21647E-05	3,14284E-06	9,771	3,02E-05	3,01E-05	2,58E-05	2,73E-05	2,25E-05	2,71659E-05	3,19006E-06	11,743
Q91YJ2	SNX4_MOUSE	Sorting nexin-4	2,04E-05	2,37E-05	2,35E-05	2,54E-05	2,32244E-05	2,07813E-06	8,948	1,98E-05	1,52E-05	2,05E-05	2,37E-05	2,33E-05	2,04949E-05	3,42096E-06	16,692
Q3V3R1	C1TM_MOUSE	Monofunctional C1-tetrahydrofolate synthase, mitochondrial	4,92E-05	5,71E-05	6,3E-05	5,24E-05	5,54082E-05	6,00479E-06	10,837	2,09E-06	5,1E-05	5,37E-05	5,32E-05	5,92E-05	4,38451E-05	2,35351E-05	53,678
P70290	EM55_MOUSE	55 kDa erythrocyte membrane protein	4,59E-05	4,41E-05	4,63E-05	4,33E-05	4,49128E-05	1,45634E-06	3,243	5,03E-05	4,65E-05	4,44E-05	4,51E-05	4,47E-05	4,61919E-05	2,41632E-06	5,231
Q8VEH3	ARL8A_MOUSE	ADP-ribosylation factor-like protein 8A	4,29E-05	4,22E-05	3,42E-05	3,94E-05	3,96958E-05	3,93552E-06	9,914	3,83E-05	4,41E-05	9,16E-06	5,04E-05	3,88E-05	3,61598E-05	1,58614E-05	43,865
P70297	STAM1_MOUSE	Signal transducing adapter molecule 1	2,51E-05	3,6E-05	2,75E-05	3,49E-05	3,08791E-05	5,38568E-06	17,441	3,76E-05	2,61E-05	2,93E-05	3,44E-05	3,23E-05	3,19327E-05	4,45807E-06	13,961
P60824	CIRBP_MOUSE	Cold-inducible RNA-binding protein	2,44E-05	2,3E-05	2,84E-05	2,3E-05	2,47016E-05	2,57356E-06	10,419	2,16E-05	2,25E-05	2,44E-05	2,39E-05	2,19E-05	2,28524E-05	1,26701E-06	5,544
Q62093	SRSF2_MOUSE	Serine/arginine-rich splicing factor 2	5,62E-05	5,88E-05	6,56E-05	5,53E-05	5,89683E-05	4,65917E-06	7,901	6,24E-05	5,8E-05	6,22E-05	5,98E-05	5,83E-05	6,0134E-05	2,10447E-06	3,500
P51880	FABP7_MOUSE	Fatty acid-binding protein, brain	1,81E-05	2,31E-05	1,97E-05	2,48E-05	2,14126E-05	3,05167E-06	14,252	2,09E-05	2,51E-05	2,57E-05	1,92E-05	1,97E-05	2,21264E-05	3,05681E-06	13,815
Q60972	RBBP4_MOUSE	Histone-binding protein RBBP4	3,87E-05	4,36E-05	4,5E-05	4,54E-05	4,3167E-05	3,10678E-06	7,197	3,85E-05	1,27E-06	4,44E-05	4,21E-05	4,12E-05	3,34906E-05	1,81318E-05	54,140

Q9Z0J4	NOS1_MOUSE	Nitric oxide synthase, brain	1,08E-05	1,44E-05	1,97E-05	1,65E-05	1,53419E-05	3,72469E-06	24,278	1,49E-05	1,5E-05	1,61E-05	1,45E-05	1,22E-05	1,45493E-05	1,40595E-06	9,663
Q64010	CRK_MOUSE	Adapter molecule crk	2,11E-06	4,12E-05	3,85E-05	5,6E-06	2,18513E-05	2,0856E-05	95,445	3,98E-05	3,2E-05	3,48E-05	4,28E-05	2,82E-06	3,04335E-05	1,60022E-05	52,581
Q9Z2Y8	PROSC_MOUSE	Proline synthase co-transcribed bacterial homolog protein	1,43E-05	1,2E-05	1,82E-05	1,92E-05	1,59224E-05	3,37972E-06	21,226	1,45E-05	1,59E-05	1,56E-05	2,91E-06	1,61E-06	1,00983E-05	7,18808E-06	71,181
P60122	RUVB1_MOUSE	RuvB-like 1	3,05E-05	3,37E-05	3,55E-05	3,22E-05	3,29548E-05	2,12534E-06	6,449	2,73E-05	2,76E-05	3,59E-05	3,06E-05	3,46E-05	3,12113E-05	3,95693E-06	12,678
Q9D9V3	ECHD1_MOUSE	Ethylmalonyl-CoA decarboxylase	6,68E-05	6,67E-05	6,01E-05	6,63E-05	6,49716E-05	3,2744E-06	5,040	7,06E-05	6,88E-05	6,76E-05	6,15E-05	5,65E-05	6,49876E-05	5,8706E-06	9,033
Q7TN29	SMAP2_MOUSE	Stromal membrane-associated protein 2	6,09E-05	7,19E-05	6,97E-05	5,81E-05	6,51562E-05	6,70607E-06	10,292	5,68E-05	5,91E-05	5,68E-05	6,13E-05	6,66E-05	6,01103E-05	4,07322E-06	6,776
P84084	ARF5_MOUSE	ADP-ribosylation factor 5	1,43E-05	2,26E-05	1,74E-05	2,1E-05	1,88313E-05	3,72788E-06	19,796	1,94E-05	2,61E-05	2,3E-05	4,93E-07	1,89E-05	1,76011E-05	9,99673E-06	56,796
Q99JW2	ACY1_MOUSE	Aminoacylase-1	2,74E-05	2,55E-05	2,56E-05	2,69E-05	2,63361E-05	9,3078E-07	3,534	2,42E-05	1,71E-05	1,98E-05	2,3E-05	2,06E-05	2,09306E-05	2,77739E-06	13,270
P62849	RS24_MOUSE	40S ribosomal protein S24	4,09E-05	5,8E-05	6,58E-05	5,21E-05	5,41663E-05	1,04944E-05	19,374	4,57E-05	5,62E-05	5,75E-05	5,61E-05	4,82E-05	5,27086E-05	5,38402E-06	10,215
O54988	SLK_MOUSE	STE20-like serine/threonine-protein kinase	3,38E-05	3,3E-05	3,34E-05	3,33E-05	3,33693E-05	3,13533E-07	0,940	3,25E-05	3,27E-05	3,1E-05	3,39E-05	3,78E-05	3,35724E-05	2,57535E-06	7,671
Q8BK64	AHSA1_MOUSE	Activator of 90 kDa heat shock protein ATPase homolog 1	0,000195	0,000224	0,000186	0,000199	0,000200883	1,61398E-05	8,034	0,000188	0,000191	0,000189	0,000175	0,000189	0,000186279	6,30343E-06	3,384
Q99KE1	MAOM_MOUSE	NAD-dependent malic enzyme, mitochondrial	7,67E-05	7,64E-05	7,72E-05	6,74E-05	7,44457E-05	4,72473E-06	6,347	8,72E-05	7,09E-05	6,93E-05	8,7E-05	8,32E-05	7,9517E-05	8,76591E-06	11,024
Q9JIF0	ANM1_MOUSE	Protein arginine N-methyltransferase 1	2,33E-05	4,43E-05	3,39E-05	4,95E-05	3,77643E-05	1,1628E-05	30,791	3,23E-06	2,59E-05	3,94E-05	4,68E-05	3,2E-05	2,94626E-05	1,66372E-05	56,469
P54775	PRS6B_MOUSE	26S protease regulatory subunit 6B	2,68E-05	2,67E-05	2,98E-05	2,74E-05	2,76692E-05	1,44055E-06	5,206	2,63E-05	2,35E-05	2,93E-05	2,63E-05	2,78E-05	2,66482E-05	2,13919E-06	8,027
Q3TEA8	HP1B3_MOUSE	Heterochromatin protein 1-binding protein 3	2,05E-05	2,62E-05	2,47E-05	2,44E-05	2,3962E-05	2,40932E-06	10,055	1,88E-05	2,41E-05	2,66E-05	1,64E-05	2,37E-05	2,19233E-05	4,19951E-06	19,155
Q925N0	SFXN5_MOUSE	Sideroflexin-5	4,83E-05	4,03E-05	2,84E-05	4,29E-05	3,99726E-05	8,43208E-06	21,095	3,36E-05	5,18E-05	3,34E-05	3,12E-05	1,85E-05	3,37165E-05	1,18852E-05	35,250
P62717	RL18A_MOUSE	60S ribosomal protein L18a	1,47E-05	1,7E-05	1,7E-05	1,63E-05	1,62347E-05	1,10154E-06	6,785	1,75E-05	1,59E-05	1,6E-05	1,4E-05	1,56E-05	1,58103E-05	1,2425E-06	7,859
Q9CQ60	6PGL_MOUSE	6-phosphogluconolactonase	5,78E-05	6,97E-05	7,45E-05	7,58E-05	6,94422E-05	8,17479E-06	11,772	5,89E-05	5,73E-05	6,97E-05	5,49E-05	6,68E-05	6,15211E-05	6,39876E-06	10,401
O09167	RL21_MOUSE	60S ribosomal protein L21	7,84E-05	8,48E-05	7,83E-05	8,13E-05	8,06704E-05	3,09202E-06	3,833	6,73E-05	6,49E-05	6,42E-05	6,78E-05	6,17E-05	6,51744E-05	2,45248E-06	3,763
Q8VDP4	CCAR2_MOUSE	Cell cycle and apoptosis regulator protein 2	2,01E-05	2,55E-05	2,38E-05	2,67E-05	2,40461E-05	2,87897E-06	11,973	2,47E-05	2,21E-05	2,45E-05	2,4E-05	2,51E-05	2,40624E-05	1,15481E-06	4,799
Q9CQ69	QCR8_MOUSE	Cytochrome b-c1 complex subunit 8	0,000237	0,000273	0,000277	0,000239	0,000256637	2,11884E-05	8,256	0,000198	0,000241	0,000252	0,000217	0,000222	0,000225864	2,10575E-05	9,323
Q8BMG7	RBGPR_MOUSE	Rab3 GTPase-activating protein non-catalytic subunit	6,48E-05	7,92E-05	7,42E-05	7,14E-05	7,2376E-05	6,03271E-06	8,335	7,31E-05	6,68E-05	7,58E-05	7,12E-05	7,14E-05	7,16717E-05	3,28563E-06	4,584
Q91W50	CSDE1_MOUSE	Cold shock domain-containing protein E1	4,57E-05	4,67E-05	3,96E-05	4,52E-05	4,43194E-05	3,18255E-06	7,181	4,45E-05	3,93E-05	4,38E-05	4,58E-05	4,87E-05	4,44285E-05	3,40607E-06	7,666
Q8CIE6	COPA_MOUSE	Coatomer subunit alpha	3,94E-05	4,08E-05	4,75E-05	4,38E-05	4,2895E-05	3,57584E-06	8,336	4,25E-05	4,33E-05	3,42E-05	1,81E-06	4,23E-05	3,28242E-05	1,77249E-05	53,999
O35685	NUDC_MOUSE	Nuclear migration protein nudC	2,82E-05	2,78E-05	2,56E-05	2,51E-05	2,66536E-05	1,5664E-06	5,877	2,79E-05	3,08E-05	3,02E-05	2,6E-05	2,38E-05	2,77239E-05	2,90644E-06	10,484
P61164	ACTZ_MOUSE	Alpha-centractin	0,000159	0,000169	0,000121	0,000158	0,000151705	2,10609E-05	13,883	0,000174	0,00017	0,00015	0,00015	0,000146	0,000157892	1,28899E-05	8,164
P62281	RS11_MOUSE	40S ribosomal protein S11	1,04E-05	9,71E-06	1,26E-05	1,1E-05	1,09292E-05	1,20918E-06	11,064	8,65E-06	1,15E-05	1,4E-05	1,01E-05	1,11E-05	1,10811E-05	1,95695E-06	17,660
Q9QZM0	UBQL2_MOUSE	Ubiquilin-2	4,47E-05	5,25E-05	5,27E-05	5,58E-05	5,14351E-05	4,74907E-06	9,233	4,25E-05	4,06E-05	4,01E-05	4,1E-05	5,03E-05	4,28908E-05	4,22118E-06	9,842
P97390	VPS45_MOUSE	Vacuolar protein sorting-associated protein 45	1,64E-05	2,12E-05	1,72E-05	1,85E-05	1,83437E-05	2,11477E-06	11,529	1,77E-05	1,74E-05	1,49E-05	1,6E-05	2,02E-05	1,72607E-05	2,01258E-06	11,660
Q14BI2	GRM2_MOUSE	Metabotropic glutamate receptor 2	2,25E-05	2,18E-05	2,7E-05	2,76E-05	2,47176E-05	3,00943E-06	12,175	3,14E-05	2,04E-05	2,17E-05	2,45E-05	3,28E-05	2,61685E-05	5,6604E-06	21,631
O09131	GSTO1_MOUSE	Glutathione S-transferase omega-1	1,68E-05	1,92E-05	1,72E-05	1,92E-05	1,8103E-05	1,28782E-06	7,114	1,71E-05	1,66E-05	1,85E-05	2,02E-05	1,86E-05	1,81938E-05	1,4157E-06	7,781
Q9Z0S1	BPNT1_MOUSE	3'(2'),5'-bisphosphate nucleotidase 1	2,04E-05	2,27E-05	2,52E-05	2,63E-05	2,36457E-05	2,6166E-06	11,066	2,41E-05	1,91E-05	2,41E-05	3,04E-05	2,37E-05	2,43054E-05	4,02489E-06	16,560
P61021	RAB5B_MOUSE	Ras-related protein Rab-5B	1,1E-05	1,12E-05	1,03E-05	1,02E-05	1,06652E-05	5,38963E-07	5,053	1,09E-05	1,29E-05	1,31E-05	1,07E-05	7,39E-06	1,09915E-05	2,29331E-06	20,864
Q80ZJ1	RAP2A_MOUSE	Ras-related protein Rap-2a	1,92E-05	1,9E-05	2,15E-05	2E-05	1,99266E-05	1,13886E-06	5,715	2,14E-05	1,18E-05	1,74E-05	1,5E-05	1,17E-05	1,54501E-05	4,06264E-06	26,295
P21995	EMB_MOUSE	Embigin	3,8E-05	4,29E-05	3,7E-05	4,17E-05	3,99325E-05	2,83319E-06	7,095	4,22E-05	4,06E-05	4,45E-05	3,95E-05	4,42E-05	4,22081E-05	2,16627E-06	5,132
Q8BK08	TMM11_MOUSE	Transmembrane protein 11, mitochondrial	3,28E-05	2,97E-05	3,04E-05	3,03E-05	3,08164E-05	1,35102E-06	4,384	2,63E-05	2,94E-05	2,89E-05	2,34E-05	3,01E-05	2,76272E-05	2,73715E-06	9,907
Q8VE62	PAIP1_MOUSE	Polyadenylate-binding protein-interacting protein 1	3,84E-05	4,29E-05	4,57E-05	4,53E-05	4,3068E-05	3,36752E-06	7,819	3,55E-05	3,61E-05	3,79E-05	3,35E-05	4,67E-05	3,79796E-05	5,14206E-06	13,539
P62069	UBP46_MOUSE	Ubiquitin carboxyl-terminal hydrolase 46	3,02E-05	2,77E-05	3,64E-05	2,99E-05	3,10585E-05	3,74547E-06	12,059	2,81E-05	2,35E-05	3,23E-05	2,56E-05	2,88E-05	2,76678E-05	3,33169E-06	12,042
P70188	KIFA3_MOUSE	Kinesin-associated protein 3	5,67E-05	5,6E-05	5,6E-05	4,99E-05	5,46284E-05	3,19866E-06	5,855	5,29E-05	4,9E-05	5,87E-05	4,2E-05	4,94E-05	5,03951E-05	6,09439E-06	12,093
Q8R1N4	NUDC3_MOUSE	NudC domain-containing protein 3	2,48E-05	1,77E-05	2,64E-05	2,48E-05	2,34588E-05	3,88756E-06	16,572	1,62E-05	1,23E-05	1,82E-05	1,59E-05	1,34E-05	1,52127E-05	2,3683E-06	15,568
Q8BHK1	NIPA1_MOUSE	#N/A	7,02E-06	6,84E-06	4,56E-06	5,8E-06	6,05564E-06	1,13041E-06	18,667	5,58E-06	6,9E-06	6,13E-06	4,39E-06	3,39E-06	5,27721E-06	1,39488E-06	26,432
Q04690	NF1_MOUSE	Neurofibromin	6,02E-05	8,67E-05	8,1E-05	8,52E-05	7,82832E-05	1,23066E-05	15,721	8,22E-05	6,75E-05	8,18E-05	8,54E-05	8,43E-05	8,02556E-05	7,26118E-06	9,048
A2ALS5	RPGP1_MOUSE	Rap1 GTPase-activating protein 1	6,86E-05	7,05E-05	7,2E-05	7,23E-05	7,08642E-05	1,70381E-06	2,404	7,19E-05	7,68E-05	2,58E-05	6,14E-05	7,08E-05	6,13383E-05	2,06186E-05	33,615
Q8CHP8	PGP_MOUSE	Glycerol-3-phosphate phosphatase	2,28E-05	2,07E-05	2,36E-05	2,97E-05	2,41914E-05	3,86753E-06	15,987	2,66E-05	2,4E-05	2,8E-05	2,92E-05	2,51E-05	2,65857E-05	2,09485E-06	7,880
P62751	RL23A_MOUSE	60S ribosomal protein L23a	6,42E-05	7,22E-05	6,22E-05	6,75E-05	6,64956E-05	4,36745E-06	6,568	4,22E-05	6,41E-05	6,36E-05	6,43E-05	6,52E-05	5,98969E-05	9,90577E-06	16,538
Q99MN9	PCCB_MOUSE	Propionyl-CoA carboxylase beta chain, mitochondrial	6,91E-07	1,5E-05	1,85E-05	1,73E-05	1,2856E-05	8,24157E-06	64,107	1,63E-05	1,45E-05	1,63E-05	1,55E-05	1,55E-05	1,56091E-05	7,15501E-07	4,584
Q9DCS3	MECR_MOUSE	Trans-2-enoyl-CoA reductase, mitochondrial	2,59E-05	3,06E-05	2,63E-05	2,95E-05	2,80824E-05	2,34491E-06	8,350	2,66E-05	2,39E-05	2,14E-05	1,68E-05	1,95E-05	2,16116E-05	3,80922E-06	17,626
Q80TT2	BAIP3_MOUSE	BAI1-associated protein 3	2,02E-05	2,36E-05	2,22E-05	2,32E-05	2,23187E-05	1,52145E-06	6,817	1,9E-05	2,36E-05	2,12E-05	1,95E-05	2,31E-05	2,12764E-05	2,03951E-06	9,586
Q91VF2	HNMT_MOUSE	Histamine N-methyltransferase	6,25E-05	5,33E-05	4,89E-05	4,85E-05	5,33076E-05	6,48781E-06	12,171	5,53E-05	5,65E-05	5,5E-05	4,33E-05	4E-05	5,00362E-05	7,72397E-06	15,437
P62746	RHOB_MOUSE	Rho-related GTP-binding protein RhoB	3,52E-05	2,98E-05	3,99E-05	4,06E-05	3,63847E-05	5,02954E-06	13,823	2,74E-05	3,67E-06	3,54E-05	2,94E-05	3,43E-05	2,60409E-05	1,29306E-05	49,655
Q3UVL4	VPS51_MOUSE	Vacuolar protein sorting-associated protein 51 homolog	2,71E-05	3,18E-05	2,95E-05	3,17E-05	3,00263E-05	2,19697E-06	7,317	2,95E-05	2,57E-05	2,97E-05	2,43E-05	3,58E-05	2,89751E-05	4,45069E-06	15,360
P62245	RS15A_MOUSE	40S ribosomal protein S15a	2,25E-05	2,66E-05	2,44E-05	2,35E-05	2,42451E-05	1,78048E-06	7,344	2,58E-05	2,47E-05	2,75E-05	2,25E-05	2,11E-05	2,43459E-05	2,5516E-06	10,481
Q9QZ23	NFU1_MOUSE	NFU1 iron-sulfur cluster scaffold homolog, mitochondrial	4,82E-05	2,74E-05	4,49E-05	4,76E-05	4,20075E-05	9,8654E-06	23,485	3,97E-05	2,87E-05	3,83E-05	3,81E-05	2,31E-05	3,35733E-05	7,30433E-06	21,756
P50114	S100B_MOUSE	Protein S100-B	3,83E-05	4,15E-05	5,59E-05	4,44E-05	4,50392E-05	7,64467E-06	16,973	3,02E-05	2,06E-05	3,7E-05	4,08E-05	3,07E-05	3,18456E-05	7,67341E-06	24,096
Q99KV1	DJB11_MOUSE	DnaJ homolog subfamily B member 11	3,43E-05	3,6E-05	3,71E-05	3,92E-05	3,66615E-05	2,07329E-06	5,655	3,78E-05	3,76E-05	3,38E-05	3,28E-05	3,38E-05	3,51565E-05	2,36054E-06	6,714
Q8R2V5	ADAP2_MOUSE	Arf-GAP with dual PH domain-containing protein 2	4,21E-05	4,04E-05	4,24E-05	3,64E-05	4,03248E-05	2,79795E-06	6,939	3,22E-05	2,04E-05	2,86E-05	2,26E-05	2,11E-05	2,49608E-05	5,16253E-06	20,683
Q2PFD7	PSD3_MOUSE	PH and SEC7 domain-containing protein 3	4,73E-05	3,82E-05	5,75E-05	4,82E-05	4,77995E-05	7,88093E-06	16,487	3,95E-05	3,96E-05	4,58E-05	4,38E-05	4,64E-05	4,30265E-05	3,28543E-06	7,636
Q9EQ06	DHB11_MOUSE	Estradiol 17-beta-dehydrogenase 11	4,61E-05	4,59E-05	4,01E-05	3,84E-05	4,26292E-05	3,94086E-06	9,244	4,55E-05	6,51E-05	3,92E-05	3,44E-05	3,13E-05	4,31041E-05	1,34166E-05	31,126
E9Q3L2	PI4KA_MOUSE	Phosphatidylinositol 4-kinase alpha	2,16E-05	5,05E-06	1,95E-05	1,94E-05	1,63832E-05	7,61992E-06	46,511	1,84E-05	1,93E-05	2,01E-05	1,7E-05	1,89E-05	1,8731E-05	1,1286E-06	6,025
Q61768	KINH_MOUSE	Kinesin-1 heavy chain	5,63E-05	5,27E-05	5,32E-05	4,24E-05	5,11536E-05	6,03995E-06	11,807	5,25E-05	5,82E-05	5,39E-05	6,08E-05	6,37E-05	5,78125E-05	4,68107E-06	8,097
Q9JHK4	PGTA_MOUSE	Geranylgeranyl transferase type-2 subunit alpha	2,84E-05	2,91E-05	2,79E-05	2,54E-05	2,77031E-05	1,58427E-06	5,719	2,5E-05	2,29E-05	5,81E-06	2,48E-05	1,98E-05	1,96898E-05	8,03478E-06	40,807
P70195	PSB7_MOUSE	Proteasome subunit beta type-7	2,25E-05	1,51E-05	2,28E-05	2,67E-05	2,17963E-05	4,86478E-06	22,319	1,9E-05	1,52E-05	1,34E-05	1,17E-05	9,14E-07	1,20514E-05	6,77655E-06	56,230
Q8BX70	VP13C_MOUSE	Vacuolar protein sorting-associated protein 13C	1,5E-05	2,37E-05	2E-05	1,79E-05	1,91325E-05	3,65999E-06	19,130	1,98E-05	1,97E-05	1,87E-05	1,86E-05	2,28E-05	1,99076E-05	1,71134E-06	8,596
Q91W89	MA2C1_MOUSE	Alpha-mannosidase 2C1	2,29E-05	2,36E-05	2,18E-05	2,41E-05	2,31205E-05	9,82381E-07	4,249	2,77E-05	2,25E-05	2,32E-05	2,16E-05	2,45E-05	2,39002E-05	2,38256E-06	9,969
Q9WVL0	MAAI_MOUSE	Maleylacetoacetate isomerase	3,97E-05	7,7E-06	3,86E-05	3,72E-05	3,07783E-05	1,54208E-05	50,103	2,75E-05	3,59E-05	3,3E-05	3,06E-05	2,1E-05	2,95951E-05	5,71063E-06	19,296
Q9DCT1	AKCL2_MOUSE	1,5-anhydro-D-fructose reductase	1,79E-05	1,94E-05	1,96E-05	9,75E-07	1,44606E-05	9,02277E-06	62,395	1,72E-05	2,08E-05	2,24E-05	2,12E-05	1,79E-05	1,99141E-05	2,24058E-06	11,251
O88456	CPNS1_MOUSE	Calpain small subunit 1	4,18E-05	4,4E-05	3,69E-05	4,81E-05	4,27108E-05	4,66815E-06	10,930	5E-05	5,43E-05	5,12E-05	4,87E-05	4,45E-05	4,97381E-05	3,59054E-06	7,219
Q8VHL1	SETD7_MOUSE	Histone-lysine N-methyltransferase SETD7	3,07E-05	2,46E-05	2,81E-05	2,69E-05	2,75821E-05	2,53971E-06	9,208	3,08E-05	2,49E-05	2,9E-05	2,85E-05	2,41E-05	2,74481E-05	2,83608E-06	10,332
Q8VEK0	CC50A_MOUSE	Cell cycle control protein 50A	4,55E-06	2,77E-05	3,22E-05	2,54E-05	2,24532E-05	1,22685E-05	54,640	1,87E-05	2,61E-05	2,47E-05	2,53E-05	2,87E-05	2,47161E-05	3,66806E-06	14,841
Q9D0M5	DYL2_MOUSE	Dynein light chain 2, cytoplasmic	1,71E-05	1,63E-05	1,97E-05	1,75E-05	1,76419E-05	1,44828E-06	8,209	1,17E-05	1,32E-05	1,3E-05	1,42E-05	1,21E-05	1,28544E-05	9,81931E-07	7,639
Q99K46	UBP11_MOUSE	Ubiquitin carboxyl-terminal hydrolase 11	2,38E-05	2,95E-05	3,31E-05	2,76E-05	2,85153E-05	3,87054E-06	13,574	2,81E-05	2,91E-05	2,73E-05	3,18E-05	3,59E-05	3,04408E-05	3,47086E-06	11,402

P43276	H15_MOUSE	Histone H1.5	5,56E-05	5,46E-05	6,42E-05	6,82E-05	6,06462E-05	6,60487E-06	10,891	5,89E-05	6,9E-05	6,62E-05	6,05E-05	7,08E-05	6,50549E-05	5,19518E-06	7,986
Q8BL65	ABLM2_MOUSE	Actin-binding LIM protein 2	3,3E-05	3,12E-05	3,79E-05	3,38E-05	3,39975E-05	2,83996E-06	8,353	3,26E-05	3,43E-05	3,27E-05	2,97E-05	3,15E-05	3,21534E-05	1,71225E-06	5,325
Q8QZR5	ALAT1_MOUSE	Alanine aminotransferase 1	2,45E-05	2,26E-05	2,45E-05	2,35E-05	2,37732E-05	9,21481E-07	3,876	2,27E-05	1,34E-05	2,18E-05	2,18E-05	1,96E-05	1,98624E-05	3,77596E-06	19,011
Q8CA71	SHSA4_MOUSE	Protein shisa-4	4,42E-05	3,96E-05	4,66E-05	4,6E-05	4,40996E-05	3,16883E-06	7,186	3,54E-05	3,82E-05	3,56E-05	3,35E-05	2,84E-05	3,42229E-05	3,66445E-06	10,708
Q7TPR4	ACTN1_MOUSE	Alpha-actinin-1	1,94E-05	2,43E-05	3,86E-05	2,91E-05	2,78588E-05	8,1603E-06	29,292	3,55E-05	2,76E-05	3,47E-05	3,41E-06	5,56E-05	3,13708E-05	1,87953E-05	59,913
Q6P5E4	UGGG1_MOUSE	UDP-glucose:glycoprotein glucosyltransferase 1	4,78E-06	5,68E-06	5,49E-06	4,13E-06	5,02133E-06	7,09692E-07	14,134	4,63E-06	5,12E-06	4,43E-06	4,75E-06	2,85E-06	4,35736E-06	8,77921E-07	20,148
Q80UG2	PLXA4_MOUSE	Plexin-A4	5,75E-06	1,07E-05	1,16E-05	1E-05	9,51194E-06	2,58341E-06	27,160	9,55E-06	8,51E-06	9,94E-06	9,6E-06	2,32E-07	7,56628E-06	4,13476E-06	54,647
O08759	UBE3A_MOUSE	Ubiquitin-protein ligase E3A	2,73E-05	4,17E-05	2,83E-05	4,33E-05	3,51312E-05	8,52036E-06	24,253	3,18E-05	3,89E-05	3,9E-05	3,8E-05	3,61E-05	3,6771E-05	3,00083E-06	8,161
P70206	PLXA1_MOUSE	Plexin-A1	1,55E-05	1,83E-05	1,77E-05	2,38E-05	1,88202E-05	3,51316E-06	18,667	2,01E-05	1,87E-05	1,74E-05	2,01E-05	2,06E-05	1,93919E-05	1,3255E-06	6,835
D3YVF0	AKAP5_MOUSE	A-kinase anchor protein 5	6,7E-06	4,52E-06	1,42E-05	9,15E-06	8,63539E-06	4,14476E-06	47,997	8,92E-06	8,03E-06	7,88E-06	8,42E-06	1,73E-05	1,01095E-05	4,03283E-06	39,892
Q8CHT0	AL4A1_MOUSE	Delta-1-pyrroline-5-carboxylate dehydrogenase, mitochondrial	3,65E-05	4,47E-05	4,79E-05	4,21E-05	4,28008E-05	4,83041E-06	11,286	1,56E-05	5,51E-05	4,75E-05	4,86E-05	4,43E-05	4,22366E-05	1,53811E-05	36,416
Q6GQT9	NOMO1_MOUSE	Nodal modulator 1	3,06E-05	3,24E-05	3,07E-05	3,36E-05	3,17913E-05	1,4624E-06	4,600	2,92E-05	2,48E-05	2,7E-05	3,64E-06	2,92E-05	2,27591E-05	1,08427E-05	47,641
Q60902	EP15R_MOUSE	Epidermal growth factor receptor substrate 15-like 1	1,41E-05	1,83E-05	2,19E-05	1,79E-05	1,8035E-05	3,19251E-06	17,702	2,02E-05	1,55E-05	1,82E-05	1,38E-05	1,72E-05	1,69787E-05	2,44706E-06	14,413
P47791	GSHR_MOUSE	Glutathione reductase, mitochondrial	4,95E-05	3,57E-05	3,76E-05	4,56E-05	4,21039E-05	6,54995E-06	15,557	3,06E-05	4,15E-05	4,6E-05	4E-05	4,4E-05	4,04373E-05	5,98371E-06	14,797
P28661	SEPT4_MOUSE	Septin-4	1,63E-05	1,37E-05	1,29E-05	1,5E-06	1,10704E-05	6,54197E-06	59,094	1,73E-05	1,18E-05	1,36E-05	1,14E-05	1,43E-05	1,36726E-05	2,37498E-06	17,370
Q8BNW9	KBTBB_MOUSE	Kelch repeat and BTB domain-containing protein 11	2,12E-05	2,13E-05	1,52E-05	2,16E-05	1,982E-05	3,10977E-06	15,690	1,46E-05	1,53E-05	1,78E-05	1,77E-05	2,46E-05	1,79832E-05	3,97486E-06	22,103
Q8BIW1	PRUNE_MOUSE	Protein prune homolog	1,76E-05	1,75E-05	2,07E-05	1,71E-05	1,82373E-05	1,65941E-06	9,099	1,46E-05	1,85E-05	1,96E-05	1,75E-05	2,05E-05	1,81387E-05	2,30341E-06	12,699
Q505F5	LRC47_MOUSE	Leucine-rich repeat-containing protein 47	5,3E-06	4,38E-06	3,92E-06	4,77E-06	4,59194E-06	5,84615E-07	12,731	4,62E-06	8,94E-07	4,66E-06	3,8E-06	4,4E-06	3,67596E-06	1,59227E-06	43,316
Q05BC3	EMAL1_MOUSE	Echinoderm microtubule-associated protein-like 1	1,67E-05	1,27E-05	1,3E-05	1,51E-05	1,43873E-05	1,85063E-06	12,863	2,22E-05	1,85E-05	1,9E-05	1,61E-05	1,7E-05	1,85416E-05	2,37693E-06	12,819
Q80WM4	HPLN4_MOUSE	Hyaluronan and proteoglycan link protein 4	2,29E-05	3,04E-05	2,59E-05	2,83E-05	2,68813E-05	3,24132E-06	12,058	2,37E-05	2,86E-05	2,83E-05	2,72E-05	2,46E-05	2,64811E-05	2,21146E-06	8,351
Q3V0K9	PLSI_MOUSE	Plastin-1	1,05E-05	1,09E-05	9,74E-06	7,58E-06	9,69481E-06	1,4956E-06	15,427	8,08E-06	6,64E-06	8,08E-06	6,76E-06	6,36E-06	7,18586E-06	8,2962E-07	11,545
A2RT62	FXL16_MOUSE	F-box/LRR-repeat protein 16	6,49E-05	5,84E-05	7,08E-05	6,29E-05	6,42429E-05	5,16585E-06	8,041	5,14E-05	4,87E-05	5,63E-05	5,94E-05	6,6E-05	5,63478E-05	6,79258E-06	12,055
Q8CBY8	DCTN4_MOUSE	Dynactin subunit 4	3,89E-05	4,43E-05	4,09E-05	3,9E-05	4,07883E-05	2,54267E-06	6,234	4,72E-05	4,26E-05	9,9E-06	3,72E-05	4,05E-05	3,54839E-05	1,47595E-05	41,595
Q56A07	SCN2B_MOUSE	Sodium channel subunit beta-2	8,19E-05	7,34E-05	8,25E-05	7,59E-05	7,84482E-05	4,47409E-06	5,703	7,03E-05	6,46E-05	6,89E-05	5,85E-05	6,12E-05	6,47193E-05	4,99133E-06	7,712
Q9JJU8	SH3L1_MOUSE	SH3 domain-binding glutamic acid-rich-like protein	1,5E-05	2,16E-05	2,32E-05	2,33E-05	2,07626E-05	3,89005E-06	18,736	1,41E-05	1,69E-05	2,01E-05	1,41E-05	1,67E-05	1,6403E-05	2,48435E-06	15,146
Q3UNZ8	QORL2_MOUSE	Quinone oxidoreductase-like protein 2	3,1E-05	3E-05	8,53E-06	3,12E-05	2,51844E-05	1,11131E-05	44,127	2,8E-05	3,21E-05	2,52E-05	2,74E-05	2,83E-05	2,8365E-05	2,50276E-06	8,823
P63330	PP2AA_MOUSE	Serine/threonine-protein phosphatase 2A catalytic subunit alpha isoform	0,000569	0,000567	0,000641	0,000683	0,000614993	5,68356E-05	9,242	0,000614	0,000588	0,000582	0,00058	0,000535	0,000579677	2,8682E-05	4,948
P62984	RL40_MOUSE	#N/A	0,001245	0,001498	0,001377	0,001283	0,001350617	0,000112929	8,361	0,001358	0,001226	0,001113	0,001199	0,001277	0,001234659	9,10429E-05	7,374
P37040	NCPR_MOUSE	NADPH--cytochrome P450 reductase	0,000107	0,000124	9,57E-05	0,000107	0,000108461	1,1509E-05	10,611	7,61E-05	0,000117	0,000121	0,000132	0,000128	0,000115015	2,24847E-05	19,549
Q9WUL7	ARL3_MOUSE	ADP-ribosylation factor-like protein 3	0,000108	0,000121	0,000116	9,36E-05	0,000109561	1,20296E-05	10,980	0,000125	0,000106	0,000109	0,000117	9,69E-05	0,000110894	1,07351E-05	9,680
Q99JR1	SFXN1_MOUSE	Sideroflexin-1	0,00022	0,000215	0,000222	0,000204	0,000215309	7,871E-06	3,656	0,000183	0,000201	0,00021	0,000202	0,000189	0,000196909	1,07155E-05	5,442
P34884	MIF_MOUSE	Macrophage migration inhibitory factor	0,000417	0,000544	0,000653	0,000565	0,000544729	9,72526E-05	17,853	0,00043	0,000597	0,000561	0,000589	0,000581	0,000551663	6,93982E-05	12,580
Q9CPQ8	ATP5L_MOUSE	ATP synthase subunit g, mitochondrial	5,9E-05	6,95E-05	7,92E-05	6,07E-05	6,70944E-05	9,30299E-06	13,866	4,91E-05	6,23E-05	7,07E-05	5,7E-05	6,11E-05	6,00452E-05	7,90774E-06	13,170
Q78IK2	USMG5_MOUSE	Up-regulated during skeletal muscle growth protein 5	0,000262	0,000289	0,000356	0,000287	0,000298385	4,00537E-05	13,423	0,000227	0,000264	0,000283	0,000252	0,000242	0,00025384	2,13322E-05	8,404
P27048	RSMB_MOUSE	#N/A	6,77E-05	7,77E-05	7,83E-05	7,78E-05	7,53841E-05	5,10023E-06	6,766	6,69E-05	6,39E-05	7,47E-05	6,61E-05	6,64E-05	6,75838E-05	4,12537E-06	6,104
Q8R1B4	EIF3C_MOUSE	Eukaryotic translation initiation factor 3 subunit C	3,52E-05	4,38E-05	3,8E-05	3,89E-05	3,89559E-05	3,56476E-06	9,151	4,27E-05	3,6E-05	4,09E-05	3,56E-05	3,76E-05	3,8574E-05	3,11771E-06	8,082
P84104	SRSF3_MOUSE	Serine/arginine-rich splicing factor 3	0,000124	0,000176	0,000176	0,000174	0,000162326	2,55465E-05	15,738	0,000149	0,000154	0,000181	0,000153	0,000169	0,000161099	1,32818E-05	8,244
P70663	SPRL1_MOUSE	SPARC-like protein 1	4,5E-05	4,3E-05	4,04E-05	4,09E-05	4,23202E-05	2,0958E-06	4,952	4,22E-05	3,92E-05	4,1E-05	3,94E-05	3,61E-05	3,95679E-05	2,30433E-06	5,824
Q91VZ6	SMAP1_MOUSE	Stromal membrane-associated protein 1	0,000218	0,000221	0,000219	0,000218	0,00021906	1,20447E-06	0,550	0,00023	0,000209	0,000209	0,000205	0,000224	0,000215418	1,11613E-05	5,181
Q91VR7	MLP3A_MOUSE	Microtubule-associated proteins 1A/1B light chain 3A	5,07E-05	6,17E-05	7,74E-05	6,51E-05	6,37103E-05	1,10148E-05	17,289	6,17E-05	6,37E-05	7,98E-05	8,45E-05	7,28E-05	7,25076E-05	9,91223E-06	13,671
Q61696	HS71A_MOUSE	Heat shock 70 kDa protein 1A	6,59E-05	9,51E-05	9,78E-05	3,55E-05	7,35812E-05	2,9231E-05	39,726	6,66E-05	6,35E-05	7,52E-05	7,02E-05	7,1E-05	6,92997E-05	4,4349E-06	6,400
Q9JL62	GLTP_MOUSE	Glycolipid transfer protein	0,000104	0,000117	7,49E-05	8,51E-05	9,52196E-05	1,87461E-05	19,687	9,41E-05	9,47E-05	9,71E-05	8,41E-05	6,26E-05	8,65003E-05	1,42745E-05	16,502
A2A690	TANC2_MOUSE	Protein TANC2	0,00043	0,000456	0,000604	0,000558	0,000511911	8,26682E-05	16,149	0,000389	0,000368	0,000421	0,000385	0,000371	0,000386717	2,11289E-05	5,464
Q9D3A9	TTYH1_MOUSE	Protein tweety homolog 1	5,41E-05	6,17E-05	5,9E-05	5,64E-05	5,77964E-05	3,28559E-06	5,685	5,81E-05	4,73E-05	5,47E-05	5,23E-05	6,08E-05	5,46362E-05	5,24012E-06	9,591
Q3TTY5	K22E_MOUSE	Keratin, type II cytoskeletal 2 epidermal	3,28E-05	4,85E-05	6,05E-05	4,69E-05	4,71686E-05	1,13242E-05	24,008	7,42E-05	0,000345	0,000117	9,06E-05	5,36E-05	0,000135989	0,000119102	87,583
Q922Q8	LRC59_MOUSE	Leucine-rich repeat-containing protein 59	7,86E-05	7,04E-05	6,51E-05	7,06E-05	7,11777E-05	5,56414E-06	7,817	7,51E-05	7,85E-05	6,27E-05	6,58E-05	6,23E-05	6,88787E-05	7,45027E-06	10,817
Q9DAS9	GBG12_MOUSE	Guanine nucleotide-binding protein G(I)/G(S)/G(O) subunit gamma-12	0,000113	0,000124	0,000122	2,79E-05	9,67356E-05	4,61426E-05	47,700	0,000125	9,81E-05	0,000149	0,000165	0,000137	0,000134684	2,51051E-05	18,640
Q6ZWV7	RL35_MOUSE	60S ribosomal protein L35	2,75E-05	4,27E-05	4,49E-05	4,49E-05	4,00138E-05	8,39891E-06	20,990	4,22E-05	4,97E-05	5,65E-05	4,51E-05	4,8E-05	4,82948E-05	5,3974E-06	11,176
Q8R1V4	TMED4_MOUSE	Transmembrane emp24 domain-containing protein 4	3,42E-05	3,44E-05	9,88E-06	2,46E-05	2,5759E-05	1,15378E-05	44,791	2,93E-05	4,2E-05	2,7E-05	2,55E-05	2,44E-05	2,96491E-05	7,14086E-06	24,085
Q922Q4	P5CR2_MOUSE	Pyrroline-5-carboxylate reductase 2	2,44E-05	2,3E-05	2,38E-05	2,54E-05	2,41321E-05	1,00967E-06	4,184	2,57E-05	2,37E-05	2,29E-05	2,46E-05	2,5E-05	2,44183E-05	1,10255E-06	4,515
P63087	PP1G_MOUSE	Serine/threonine-protein phosphatase PP1-gamma catalytic subunit	1,67E-05	3,52E-05	3,45E-05	3,27E-05	2,9756E-05	8,78481E-06	29,523	3,82E-05	3,1E-05	3,3E-05	3,63E-05	2,95E-05	3,36093E-05	3,58174E-06	10,657
P97494	GSH1_MOUSE	Glutamate--cysteine ligase catalytic subunit	7,44E-05	7,7E-05	8,18E-05	7,85E-05	7,79093E-05	3,10408E-06	3,984	7,1E-05	6,65E-05	7,45E-05	7,17E-05	8,17E-05	7,30738E-05	5,59383E-06	7,655
Q8K2C9	HACD3_MOUSE	Very-long-chain (3R)-3-hydroxyacyl-CoA dehydratase 3	0,000117	0,00013	0,000115	0,000117	0,000119949	6,81954E-06	5,685	0,000116	0,000113	7,63E-05	0,000108	0,000119	0,000106543	1,74067E-05	16,338
Q9D1G5	LRC57_MOUSE	Leucine-rich repeat-containing protein 57	4,44E-05	3,64E-05	4,3E-05	1,05E-05	3,35609E-05	1,57641E-05	46,972	3,55E-05	4,11E-05	3,91E-05	4,06E-05	3,17E-05	3,75891E-05	3,96944E-06	10,560
Q9CRA7	ATP5S_MOUSE	ATP synthase subunit s, mitochondrial	0,000235	0,000211	0,000199	0,000234	0,000219761	1,75901E-05	8,004	0,000192	0,0002	0,000235	0,000255	0,000203	0,000217196	2,69749E-05	12,420
Q9DCZ1	GMPR1_MOUSE	GMP reductase 1	4,1E-05	4,29E-05	3,61E-05	3,5E-05	3,87286E-05	3,8009E-06	9,814	3,49E-05	3,31E-05	3,49E-05	3,74E-05	3,01E-05	3,40716E-05	2,72128E-06	7,987
Q8BY89	CTL2_MOUSE	Choline transporter-like protein 2	2,85E-05	4,38E-05	4,08E-05	2,71E-05	3,50559E-05	8,49985E-06	24,247	3,54E-05	2,96E-05	4,39E-05	4,67E-05	4,42E-05	3,99474E-05	7,18986E-06	17,998
Q9WUA2	SYFB_MOUSE	Phenylalanine--tRNA ligase beta subunit	2,97E-05	3,47E-05	3,4E-05	3E-05	3,20627E-05	2,62473E-06	8,186	3,5E-05	3,36E-05	3,28E-05	2,73E-05	3,21E-05	3,21608E-05	2,9147E-06	9,063
Q641P0	ARP3B_MOUSE	Actin-related protein 3B	3,42E-05	4,33E-05	4,62E-05	3,97E-05	4,08492E-05	5,1697E-06	12,656	4,63E-05	3,8E-05	5,04E-05	4,8E-05	5,68E-05	4,79176E-05	6,83133E-06	14,256
Q9DBL7	COASY_MOUSE	Bifunctional coenzyme A synthase	3,54E-05	3,94E-05	3,29E-05	6,99E-06	2,86843E-05	1,47032E-05	51,259	3,87E-05	3,41E-05	3,26E-05	3,52E-05	3,35E-05	3,48287E-05	2,36607E-06	6,793
P17047	LAMP2_MOUSE	Lysosome-associated membrane glycoprotein 2	9,82E-07	4,09E-05	2,75E-05	4,13E-05	2,76638E-05	1,89142E-05	68,371	3,33E-05	2,58E-05	3,48E-05	3,23E-05	3,18E-05	3,15918E-05	3,42889E-06	10,854
O35345	IMA7_MOUSE	Importin subunit alpha-7	8,11E-05	6,16E-05	6,71E-05	7,4E-05	7,09478E-05	8,47726E-06	11,949	7,24E-05	9,51E-05	8E-05	8,46E-05	7,57E-05	8,15313E-05	8,85446E-06	10,860
P43277	H13_MOUSE	#N/A	5,97E-05	6,9E-05	7,41E-05	8,25E-05	7,13463E-05	9,5407E-06	13,372	6,19E-05	7,84E-05	7,25E-05	6,78E-05	7,98E-05	7,21011E-05	7,45271E-06	10,336
Q8CGC7	SYEP_MOUSE	Bifunctional glutamate/proline--tRNA ligase	4,86E-05	5,67E-05	5,1E-05	5,61E-05	5,31115E-05	3,95409E-06	7,445	4,9E-05	5,23E-05	4,49E-05	5,28E-05	5,6E-05	5,10278E-05	4,23272E-06	8,295
Q62189	SNRPA_MOUSE	U1 small nuclear ribonucleoprotein A	7,07E-05	5,3E-05	5,92E-05	5,85E-05	6,03464E-05	7,45112E-06	12,347	6,9E-05	6,21E-05	5,5E-05	4,51E-05	4,36E-05	5,49598E-05	1,08989E-05	19,831
P62267	RS23_MOUSE	40S ribosomal protein S23	8,01E-06	1,25E-05	9,55E-06	1,17E-05	1,0442E-05	2,04317E-06	19,567	1,05E-05	1,57E-05	1,34E-05	1,03E-05	1,05E-05	1,20888E-05	2,40425E-06	19,888
Q9JMH9	MY18A_MOUSE	Unconventional myosin-XVIIIa	5,12E-05	5,57E-05	5,42E-05	8,57E-07	4,0473E-05	2,64786E-05	65,423	5,94E-05	3,47E-05	5,73E-05	6,31E-05	5,78E-05	5,44583E-05	1,12519E-05	20,662
P49443	PPM1A_MOUSE	Protein phosphatase 1A	7,14E-05	7,84E-05	8,03E-05	7,28E-05	7,57331E-05	4,26567E-06	5,633	8,21E-05	7,24E-05	7,32E-05	7,06E-05	7,39E-05	7,44257E-05	4,46084E-06	5,994
P13020	GELS_MOUSE	Gelsolin	4,73E-05	4,61E-05	3,61E-05	3,96E-05	4,22934E-05	5,33137E-06	12,606	4,4E-05	4,66E-05	4,98E-05	4,27E-05	3,81E-05	4,42344E-05	4,39848E-06	9,944
Q60605	MYL6_MOUSE	Myosin light polypeptide 6	0,000102	0,000124	8,49E-05	0,000116	0,000106509	1,70223E-05	15,982	8,93E-05	0,000117	0,000125	0,000106	0,000104	0,000108082	1,35576E-05	12,544
P67984	RL22_MOUSE	60S ribosomal protein L22	5,81E-05	8,19E-05	8,56E-05	8,42E-05	7,7441E-05	1,29692E-05	16,747	6,04E-05	7,09E-05	7,5E-05	7,83E-05	6,83E-05	7,05991E-05	6,858E-06	9,714

P00158	CYB_MOUSE	Cytochrome b	7,73E-05	8,03E-05	8,86E-05	7,96E-05	8,14486E-05	4,93757E-06	6,062	6,02E-05	6,51E-05	6,37E-05	5,89E-05	7,14E-05	6,38683E-05	4,89454E-06	7,663
Q99MK8	ARBK1_MOUSE	Beta-adrenergic receptor kinase 1	6,26E-05	6,16E-05	6,63E-05	5,19E-05	6,05777E-05	6,12986E-06	10,119	6,56E-05	6,24E-05	5,32E-05	3,8E-05	5,32E-05	5,44919E-05	1,07314E-05	19,694
P48453	PP2BB_MOUSE	Serine/threonine-protein phosphatase 2B catalytic subunit beta isoform	9,34E-05	0,000113	0,000128	0,000116	0,00011261	1,44457E-05	12,828	9,95E-05	8,46E-05	9,54E-05	0,000108	8,96E-05	9,53391E-05	8,90779E-06	9,343
Q99PV0	PRP8_MOUSE	Pre-mRNA-processing-splicing factor 8	2,86E-05	2,9E-05	3,13E-05	8,35E-06	2,43296E-05	1,07211E-05	44,066	2,86E-05	9,27E-06	2,48E-05	2,52E-05	2,69E-05	2,29533E-05	7,79884E-06	33,977
Q9CZT8	RAB3B_MOUSE	Ras-related protein Rab-3B	0,000547	0,000398	0,000422	0,000496	0,000465656	6,84085E-05	14,691	0,000495	0,000502	0,000374	0,000447	0,000386	0,0004406	5,97009E-05	13,550
Q9WV85	NDK3_MOUSE	Nucleoside diphosphate kinase 3	1,64E-05	2,36E-05	2,19E-05	2,26E-05	2,10979E-05	3,2301E-06	15,310	1,93E-05	1,78E-06	2,16E-05	1,84E-05	2,01E-05	1,62444E-05	8,16903E-06	50,288
Q8C3Q5	SHSA7_MOUSE	Protein shisa-7	2,99E-05	3,23E-05	3,79E-05	3,49E-05	3,37551E-05	3,43841E-06	10,186	3,28E-05	2,61E-05	3,21E-05	3,31E-05	3,02E-05	3,08404E-05	2,90621E-06	9,423
O08788	DCTN1_MOUSE	Dynactin subunit 1	0,000103	0,000139	0,000126	0,000119	0,000121916	1,5013E-05	12,314	0,000126	0,000133	9,4E-05	0,000123	0,000129	0,000120977	1,55284E-05	12,836
P28271	ACOC_MOUSE	Cytoplasmic aconitate hydratase	2,44E-05	2,55E-05	2,43E-05	2,58E-05	2,49992E-05	7,55738E-07	3,023	2,35E-05	2,92E-05	2,5E-05	2,49E-05	2,86E-05	2,62363E-05	2,4913E-06	9,496
Q9D358	PPAC_MOUSE	Low molecular weight phosphotyrosine protein phosphatase	6,93E-05	8,59E-05	9,13E-05	7,55E-05	8,05105E-05	9,96278E-06	12,375	2,67E-06	6,16E-05	7,55E-05	6,79E-05	6,28E-05	5,40859E-05	2,92538E-05	54,088
A2AGT5	CKAP5_MOUSE	Cytoskeleton-associated protein 5	3,4E-05	4,47E-05	3,88E-05	4,72E-05	4,11834E-05	5,93494E-06	14,411	4,83E-05	3,3E-05	4,44E-05	4,67E-05	4,33E-05	4,31426E-05	6,00434E-06	13,917
P23116	EIF3A_MOUSE	Eukaryotic translation initiation factor 3 subunit A	3,39E-05	4,55E-05	3,56E-05	3,55E-05	3,76412E-05	5,29754E-06	14,074	5,06E-05	4,81E-05	4,12E-05	3,96E-05	4,22E-05	4,4344E-05	4,75483E-06	10,723
Q9QUR6	PPCE_MOUSE	Prolyl endopeptidase	4,93E-05	4,68E-05	3,21E-06	5,01E-05	3,73638E-05	2,28104E-05	61,049	4,93E-05	4,03E-05	3,4E-05	3,78E-05	4,02E-05	4,03244E-05	5,60795E-06	13,907
P61294	RAB6B_MOUSE	Ras-related protein Rab-6B	1,92E-05	1,88E-05	2,21E-05	1,93E-05	1,98546E-05	1,4899E-06	7,504	1,95E-05	2,58E-05	2,54E-05	2,05E-05	1,65E-05	2,15535E-05	3,96482E-06	18,395
P34022	RANG_MOUSE	Ran-specific GTPase-activating protein	0,000123	0,000116	0,00013	0,00013	0,000124729	6,44134E-06	5,164	0,000115	0,000108	0,000129	0,00012	0,000122	0,000118663	7,60697E-06	6,411
Q8BFP9	PDK1_MOUSE	[Pyruvate dehydrogenase (acetyl-transferring)] kinase isozyme 1, mitochondrial	4,95E-05	4,78E-05	4,76E-05	5,1E-05	4,8965E-05	1,61063E-06	3,289	4,37E-05	4,01E-05	4E-05	4,31E-05	3,94E-05	4,12642E-05	1,97898E-06	4,796
Q61595	KTN1_MOUSE	Kinectin	0,000503	0,000174	0,000148	0,0002	0,000256344	0,000165562	64,586	0,000196	0,000274	0,000176	0,000222	0,000205	0,000214627	3,71117E-05	17,291
Q9JIG7	CCD22_MOUSE	Coiled-coil domain-containing protein 22	2,47E-05	3,55E-05	3,89E-05	2,81E-05	3,17987E-05	6,50468E-06	20,456	1,38E-06	1,43E-05	2,64E-05	2,26E-05	2,94E-05	1,88257E-05	1,12655E-05	59,841
Q9EPR4	S23A2_MOUSE	Solute carrier family 23 member 2	2,08E-05	2,41E-05	2,03E-05	2,67E-05	2,29569E-05	3,03068E-06	13,202	2,03E-05	1,92E-05	1,85E-05	1,52E-05	2,22E-05	1,90764E-05	2,5993E-06	13,626
O88737	BSN_MOUSE	Protein bassoon	1,87E-05	2,2E-05	2,77E-05	1,72E-05	2,139E-05	4,67727E-06	21,867	2,44E-05	2,29E-05	1,59E-05	1,69E-05	2,34E-05	2,07079E-05	3,9579E-06	19,113
Q8BU30	SYIC_MOUSE	Isoleucine--tRNA ligase, cytoplasmic	3,83E-05	4,66E-05	4,47E-05	3,82E-05	4,19563E-05	4,31637E-06	10,288	4,5E-05	4,23E-05	5,95E-06	3,68E-05	3,62E-05	3,32438E-05	1,57008E-05	47,229
Q8CCK0	H2AW_MOUSE	Core histone macro-H2A.2	5,71E-05	6,07E-05	5,14E-05	5,96E-05	5,72062E-05	4,16633E-06	7,283	5,92E-05	5,26E-05	5,33E-05	5,55E-05	5,43E-05	5,4962E-05	2,59774E-06	4,726
Q61543	GSLG1_MOUSE	Golgi apparatus protein 1	6,97E-05	5,35E-05	8,07E-05	6,61E-05	6,75129E-05	1,12067E-05	16,599	5,61E-05	3,91E-05	5,21E-05	5,13E-05	6,95E-05	5,3606E-05	1,09094E-05	20,351
P12815	PDCD6_MOUSE	Programmed cell death protein 6	2,48E-05	2,69E-05	3,52E-05	2,94E-06	2,24571E-05	1,37583E-05	61,265	2,27E-05	2,8E-05	2,86E-05	2,88E-05	3,06E-05	2,7748E-05	2,99864E-06	10,807
Q99M87	DNJA3_MOUSE	DnaJ homolog subfamily A member 3, mitochondrial	2,68E-05	2,84E-05	2,81E-05	2,91E-05	2,81145E-05	9,53638E-07	3,392	2,77E-05	2,37E-05	2,45E-05	2,54E-05	2,82E-05	2,59069E-05	1,95617E-06	7,551
Q921H8	THIKA_MOUSE	3-ketoacyl-CoA thiolase A, peroxisomal	2,64E-05	3,23E-05	2,86E-05	2,79E-05	2,87843E-05	2,51827E-06	8,749	2,41E-05	2,57E-05	2,53E-05	2,38E-05	3,94E-05	2,7651E-05	6,59597E-06	23,854
Q0GNC1	INF2_MOUSE	Inverted formin-2	9,14E-06	1,33E-05	1,01E-05	1,12E-05	1,09455E-05	1,7958E-06	16,407	1,52E-05	1,24E-05	1,21E-05	1,43E-05	1,63E-05	1,40323E-05	1,80337E-06	12,852
Q9CY27	TECR_MOUSE	Very-long-chain enoyl-CoA reductase	7,51E-05	7,12E-05	5,94E-05	6,87E-05	6,85985E-05	6,66794E-06	9,720	6,38E-05	5,86E-05	5,94E-05	5,27E-05	6,1E-05	5,91027E-05	4,09734E-06	6,933
Q9JKD3	SCAM5_MOUSE	Secretory carrier-associated membrane protein 5	0,000285	0,000192	0,000233	0,000236	0,000236521	3,80377E-05	16,082	0,000167	0,000284	0,000177	0,000153	0,00027	0,000210352	6,17845E-05	29,372
Q07076	ANXA7_MOUSE	Annexin A7	8,22E-05	6,36E-05	7,57E-05	6,66E-05	7,20104E-05	8,52069E-06	11,833	7,17E-05	6,87E-05	7,07E-05	7,6E-05	6,79E-05	7,09899E-05	3,17541E-06	4,473
Q9Z1R2	BAG6_MOUSE	Large proline-rich protein BAG6	4,15E-05	4,75E-05	5E-05	4,72E-05	4,65449E-05	3,58069E-06	7,693	4,59E-05	4,24E-05	4,28E-05	3,85E-05	4,76E-05	4,34507E-05	3,48362E-06	8,017
Q9D880	TIM50_MOUSE	Mitochondrial import inner membrane translocase subunit TIM50	2,78E-05	3,75E-05	2,92E-05	2,34E-05	2,94937E-05	5,89829E-06	19,998	3,15E-05	4,09E-05	2,91E-05	2,06E-05	2,93E-05	3,03026E-05	7,26018E-06	23,959
P51855	GSHB_MOUSE	Glutathione synthetase	8,72E-05	7,14E-05	7,59E-05	8,58E-05	8,0091E-05	7,65586E-06	9,559	6,29E-05	7,93E-05	8,38E-05	8,49E-05	7,01E-05	7,62176E-05	9,43896E-06	12,384
Q80UJ7	RB3GP_MOUSE	Rab3 GTPase-activating protein catalytic subunit	5,11E-05	4,59E-05	4,82E-05	4,56E-05	4,77196E-05	2,55889E-06	5,362	4,48E-05	4,41E-05	3,61E-05	3,92E-05	4,48E-05	4,17915E-05	3,94131E-06	9,431
O08583	THOC4_MOUSE	THO complex subunit 4	1,88E-05	2,12E-05	2,35E-05	2,17E-05	2,1299E-05	1,90767E-06	8,957	2,57E-05	2,75E-05	2,46E-05	1,77E-05	2,46E-05	2,40016E-05	3,73916E-06	15,579
P56376	ACYP1_MOUSE	Acylphosphatase-1	5,72E-06	7,99E-06	8,95E-06	9,47E-06	8,02951E-06	1,65923E-06	20,664	7,39E-06	7,84E-06	9,61E-06	8,21E-06	8,22E-06	8,25302E-06	8,29583E-07	10,052
P52503	NDUS6_MOUSE	NADH dehydrogenase [ubiquinone] iron-sulfur protein 6, mitochondrial	2,53E-05	2,84E-05	2,18E-05	2,4E-05	2,48602E-05	2,74627E-06	11,047	5,89E-06	3,39E-05	3,19E-05	2,46E-05	1,99E-05	2,32472E-05	1,12146E-05	48,241
P46061	RAGP1_MOUSE	Ran GTPase-activating protein 1	7,31E-05	6,78E-05	6,54E-05	6,59E-05	6,8061E-05	3,49356E-06	5,133	7,08E-05	8,85E-05	5,83E-05	7,27E-05	7,41E-05	7,28798E-05	1,07816E-05	14,794
Q60829	PPR1B_MOUSE	Protein phosphatase 1 regulatory subunit 1B	6,92E-05	4,92E-05	6,56E-05	4,08E-05	5,62247E-05	1,34598E-05	23,939	5,59E-05	4,65E-05	4,86E-05	7,14E-06	9,92E-05	5,14829E-05	3,27728E-05	63,658
Q8BHZ0	FA49A_MOUSE	Protein FAM49A	2,47E-05	2,48E-05	3,41E-05	2,82E-05	2,7948E-05	4,37966E-06	15,671	2,9E-05	2,47E-05	2,16E-05	2,73E-05	2,84E-05	2,62099E-05	3,06302E-06	11,687
O54916	REPS1_MOUSE	RalBP1-associated Eps domain-containing protein 1	1,1E-05	1,14E-05	1,36E-05	1,34E-05	1,23609E-05	1,35662E-06	10,975	1,26E-05	1,38E-05	1,28E-05	1,17E-05	1,57E-05	1,3318E-05	1,52407E-06	11,444
Q9D7H3	RTCA_MOUSE	RNA 3'-terminal phosphate cyclase	2,45E-05	3,1E-05	2,61E-05	2,33E-05	2,62304E-05	3,37707E-06	12,875	2,22E-05	1,7E-06	2,53E-05	2,42E-05	2,28E-05	1,92353E-05	9,88164E-06	51,372
P58771	TPM1_MOUSE	Tropomyosin alpha-1 chain	9,34E-05	8,94E-05	9,29E-05	8,23E-05	8,95188E-05	5,09187E-06	5,688	9,35E-05	7,81E-05	9,25E-05	8,34E-05	8,9E-05	8,73261E-05	6,47757E-06	7,418
Q9ERK4	XPO2_MOUSE	Exportin-2	4,44E-05	4,78E-05	4,48E-05	4,81E-05	4,62583E-05	1,95387E-06	4,224	4,85E-05	4,95E-05	4,56E-05	4,59E-05	4,5E-05	4,68913E-05	1,98508E-06	4,233
P21619	LMNB2_MOUSE	Lamin-B2	2,06E-05	2,16E-05	2,37E-05	2,17E-05	2,19154E-05	1,27784E-06	5,831	1,93E-05	1,74E-05	2,06E-05	1,73E-05	2,14E-05	1,91752E-05	1,86255E-06	9,713
P32020	NLTP_MOUSE	Non-specific lipid-transfer protein	2,14E-05	2,47E-05	2,77E-05	2,58E-05	2,48987E-05	2,65497E-06	10,663	2,1E-05	2,4E-05	2,38E-05	2,4E-05	2,15E-05	2,28629E-05	1,50361E-06	6,577
Q9CQ62	DECR_MOUSE	2,4-dienoyl-CoA reductase, mitochondrial	2,99E-05	2,76E-05	2,88E-05	2,71E-05	2,8368E-05	1,25935E-06	4,439	2,63E-05	2,91E-05	3,2E-05	3,12E-05	2,24E-05	2,81939E-05	3,93354E-06	13,952
P35278	RAB5C_MOUSE	Ras-related protein Rab-5C	5,91E-06	7,36E-06	5,44E-06	6,53E-06	6,31256E-06	8,309E-07	13,163	6,71E-06	8,69E-06	6,96E-06	5,62E-06	1,1E-06	5,81685E-06	2,85609E-06	49,100
Q925I1	ATAD3_MOUSE	ATPase family AAA domain-containing protein 3	5,72E-05	6,21E-05	5,34E-05	2,82E-06	4,3899E-05	2,76179E-05	62,912	5,14E-05	5,51E-05	5,58E-05	4,87E-05	5,58E-05	5,33551E-05	3,18716E-06	5,973
P45878	FKBP2_MOUSE	Peptidyl-prolyl cis-trans isomerase FKBP2	5,01E-05	5,38E-05	5,82E-05	5,15E-05	5,34131E-05	3,5142E-06	6,579	5,68E-05	6,03E-05	5,96E-05	5,76E-05	6,51E-05	5,98693E-05	3,23479E-06	5,403
Q9EPN1	NBEA_MOUSE	Neurobeachin	8,85E-06	1,43E-05	1,29E-05	4,88E-07	9,13762E-06	6,21188E-06	67,981	1,41E-05	4,17E-06	1,27E-05	1,23E-05	1,72E-05	1,21115E-05	4,84296E-06	39,986
Q9WVK4	EHD1_MOUSE	EH domain-containing protein 1	2,11E-05	4,35E-05	4,66E-05	4,48E-05	3,90058E-05	1,20169E-05	30,808	3,53E-05	4,42E-05	4,7E-05	4,81E-05	4,05E-05	4,30062E-05	5,23407E-06	12,170
Q9DBT5	AMPD2_MOUSE	AMP deaminase 2	3,99E-05	4,83E-05	4,06E-05	4,4E-05	4,32186E-05	3,8597E-06	8,931	4,14E-05	3,89E-05	3,85E-05	3,47E-05	1,04E-06	3,09238E-05	1,68764E-05	54,574
Q9CQF9	PCYOX_MOUSE	Prenylcysteine oxidase	3,15E-05	3,41E-05	2,89E-05	3,81E-05	3,31509E-05	3,95962E-06	11,944	3,31E-05	3,41E-05	3,22E-05	3,43E-05	3,97E-05	3,46635E-05	2,92949E-06	8,451
Q9D4H1	EXOC2_MOUSE	Exocyst complex component 2	3,56E-05	3,53E-05	3,78E-05	3,49E-05	3,5897E-05	1,29331E-06	3,603	3,08E-05	2,63E-05	2,68E-05	3E-05	3,26E-05	2,93158E-05	2,66961E-06	9,106
Q80ZJ6	ZER1_MOUSE	Protein zer-1 homolog	2,36E-05	2,61E-05	2,22E-05	2,82E-05	2,50126E-05	2,69081E-06	10,758	2,44E-05	1,93E-05	2,53E-05	2,47E-05	2,64E-05	2,40116E-05	2,75366E-06	11,468
Q924N4	S12A6_MOUSE	Solute carrier family 12 member 6	2,93E-05	2,93E-05	2,85E-05	2,73E-05	2,86042E-05	9,18761E-07	3,212	2,72E-05	2,5E-05	3,07E-05	2,91E-05	2,93E-05	2,82545E-05	2,1964E-06	7,774
P23198	CBX3_MOUSE	Chromobox protein homolog 3	1,08E-05	1,66E-05	1,64E-05	1,38E-05	1,44088E-05	2,73918E-06	19,010	1,26E-06	1,35E-05	1,54E-05	1,07E-05	8,11E-06	9,78194E-06	5,50682E-06	56,296
Q8VE22	RT23_MOUSE	28S ribosomal protein S23, mitochondrial	2,12E-05	1,97E-05	2,38E-05	1,68E-05	2,03591E-05	2,92643E-06	14,374	1,35E-05	1,78E-05	2,12E-05	2,02E-05	1,73E-05	1,80038E-05	2,9804E-06	16,554
Q8VED9	LEGL_MOUSE	Galectin-related protein	8,5E-06	1,18E-05	1,9E-05	1,46E-05	1,3462E-05	4,45636E-06	33,103	1,08E-05	7,94E-06	1,11E-05	6,61E-06	9,99E-06	9,29943E-06	1,95213E-06	20,992
Q9EPR5	SORC2_MOUSE	VPS10 domain-containing receptor SorCS2	1,48E-05	2,11E-05	2,05E-05	2,13E-05	1,94353E-05	3,12089E-06	16,058	1,07E-05	1,94E-05	6,05E-07	2,23E-05	2,08E-05	1,47638E-05	9,1204E-06	61,775
F6SEU4	SYGP1_MOUSE	Ras/Rap GTPase-activating protein SynGAP	6,55E-05	6,82E-05	9,98E-05	9,19E-05	8,13799E-05	1,70865E-05	20,996	7,67E-05	6,23E-05	6,49E-05	5,15E-05	8,12E-05	6,73369E-05	1,18571E-05	17,609
P61202	CSN2_MOUSE	COP9 signalosome complex subunit 2	2,59E-05	2,09E-05	2,21E-05	2,49E-05	2,34817E-05	2,32791E-06	9,914	1,87E-05	2,66E-05	2,65E-05	2,13E-05	2,07E-05	2,27658E-05	3,58773E-06	15,759
P37804	TAGL_MOUSE	Transgelin	1,09E-05	1,58E-05	1,69E-05	1,16E-05	1,37743E-05	2,98775E-06	21,691	2,38E-06	2,75E-05	1,05E-05	1,05E-05	7,68E-06	1,17286E-05	9,44153E-06	80,500
Q920E5	FPPS_MOUSE	Farnesyl pyrophosphate synthase	6,03E-05	5,91E-05	5,5E-05	6,41E-05	5,9632E-05	3,76919E-06	6,321	6,28E-05	5,64E-05	5,18E-05	5,66E-05	6,64E-06	4,68508E-05	2,28185E-05	48,705
O35927	CTND2_MOUSE	Catenin delta-2	2,69E-05	2,66E-05	1,96E-05	2,37E-05	2,41828E-05	3,368E-06	13,927	2,92E-05	1,24E-05	2,42E-05	2,65E-05	2,47E-05	2,33831E-05	6,45991E-06	27,626
Q8CGA0	PPM1F_MOUSE	Protein phosphatase 1F	2,85E-05	3,97E-05	3,84E-05	4,1E-05	3,69132E-05	5,6847E-06	15,400	2,94E-05	3,38E-05	3,79E-05	4,08E-05	3,46E-05	3,52847E-05	4,31528E-06	12,230
Q91WK2	EIF3H_MOUSE	Eukaryotic translation initiation factor 3 subunit H	2,69E-05	2,63E-05	2,68E-05	2,66E-05	2,66469E-05	2,81922E-07	1,058	2,3E-05	3,76E-06	3,01E-05	2,96E-05	2,69E-05	2,26938E-05	1,09538E-05	48,268
