# Supplementary material for: Chronic treatment with D2-antagonist haloperidol leads to inhibitory/excitatory imbalance in striatal D1-neurons
Source: Transl Psychiatry. 2023 Oct 6;13:312. doi: 10.1038/s41398-023-02609-w (PMC10558446; doi:10.1038/s41398-023-02609-w)
Supplement: Supplementary file 3 — Supplementary Table S2 [file 41398_2023_2609_MOESM3_ESM.docx]

| **Sup.Table S2 – Experimental design and statistical Analysis.** | | | | |
| --- | --- | --- | --- | --- |
| Figure | Measurement | *n* | Mean ± SEM | Statistical test and *P* value |
| Fig.1D | Downregulated Proteins | VE = 4 mice  HA = 5 mice | SYN2 VE 1.00 ± 0.037; HA 0.890 ± 0.034  DBNL VE 1.00 ± 0.013; HA 0.918 ± 0.029  CALM VE 1.00 ± 0.153; HA 0.454 ± 0.016  PICK1 VE 1.00 ± 0.010; HA 0.827 ± 0.032  STXB1 VE 1.00 ± 0.007; HA 0.923 ± 0.006  KAPCA VE 1.00 ± 0.106; HA 0.902 ± 0.078 | Two-sided Welch’s unpaired t-test (confidence level 90%)  SYN2 **p*=0.1000  DBNL**p*=0.0619  CALM **p*=0.0533  PICK1 ****p*=0.0061  STXB1 ****p*=0.0003  KAPCA *****p*=0.0007 |
| Fig.1E | Upregulated Proteins | VE = 4 mice  HA = 5 mice | KAP3 VE 1.00 ± 0.040; HA 1.169 ± 0.063  AP2B1 VE 1.00 ± 0.016; HA 1.112 ± 0.044  GAD65 VE 1.00 ± 0.070; HA 1.240 ± 0.074  AP2A2 ± VE 1.00 ± 0.015; HA 1.121 ± 0.036  AP2A1 VE 1.00 ± 0.012; HA 1.071 ± 0.020  GLS VE 1.00 ± 0.019; HA 1.079 ± 0.016  KAPCB VE 1.00 ± 0.038; HA 1.191 ± 0.040  SYN1 VE 1.00 ± 0.007; HA 1.093 ± 0.021  EAA2 VE 1.00 ± 0.020; HA 1.096 ± 0.009  GABT VE 1.00 ± 0.016; HA 1.130 ± 0.023  HSP7C VE 1.00 ± 0.022; HA 1.161 ± 0.015  TSN7 VE 1.00 ± 0.016; HA 1.354 ± 0.040 | Two-sided Welch’s unpaired t-test (confidence level 90%)  KAP3 **p*=0.0854  AP2B1 **p*=0.0827  GAD65 **p*=0.0771  AP2A2 ***p*=0.0371  AP2A1 ***p*=0.343  GLS ***p*=0.0314  KAPCB ***p*=0.0193  SYN1 ***p*=0.0125  EAA2 ***p*=0.0186  GABT ****p*=0.0051  HSP7C ****p*=0.0023  TSN7 *****p*=0.0006 |
| Fig.2C | D2-MSNs Cumulative Probability sEPSC interevent interval | 20 events per cell | - | Kolmogorov-Smirnov test, *****p*<0.0001 |
| Fig.2C inset | D2-MSNs sEPSC frequency (Hz) | VE = 15/3 cells/mice  HA = 20/4 cells/mice | VE 2.015 ± 0.336  HA 3.690 ± 0.833 | Two-sided Welch’s unpaired t-test, **p*=0.0165 |
| Fig.2D | D2-MSNs Cumulative Probability sEPSC amplitude | 20 events per cell | - | Kolmogorov-Smirnov test, *****p*<0.0001 |
| Fig.2D inset | D2-MSNs sEPSC amplitude (pA) | VE = 15/3 cells/mice  HA = 20/4 cells/mice | VE 12.480 ± 0.130  HA 15.448 ± 0.833 | Two-sided Welch’s unpaired t-test, ***p*=0.0065 |
| Fig.2E | D2-MSNs sEPSC decay time (ms) | VE = 15/3 cells/mice  HA = 20/4 cells/mice | VE 6.315 ± 0.130  HA 6.524 ± 0.228 | Two-sided Welch’s unpaired t-test, *p*=0.4448 |
| Fig.2F | D2-MSNs sEPSC rise time (ms) | VE = 15/3 cells/mice  HA = 20/4 cells/mice | VE 1.389 ± 0.051  HA 1.322 ± 0.067 | Two-sided Welch’s unpaired t-test, *p*=0.4416 |
| Fig.2H | D1-MSNs Cumulative Probability sEPSC interevent interval | 25 events per cell | - | Kolmogorov-Smirnov test, ****p*=0.0004 |
| Fig.2H inset | D1-MSNs sEPSC frequency (Hz) | VE = 15/3 cells/mice  HA = 20/4 cells/mice | VE 2.178 ± 0.0269  HA 2.914 ± 0.367 | Two-sided Welch’s unpaired t-test, *p*=0.1258 |
| Fig.2I | D1-MSNs Cumulative Probability sEPSC amplitude | 25 events per cell | - | Kolmogorov-Smirnov test, *p*=0.2445 |
| Fig.2I inset | D1-MSNs sEPSC amplitude (pA) | VE = 15/3 cells/mice  HA = 20/4 cells/mice | VE 12.998 ± 0.556  HA 13.004 ± 0.346 | Two-sided Welch’s unpaired t-test, *p*=0.9930 |
| Fig.2J | D1-MSNs sEPSC decay time (ms) | VE = 15/3 cells/mice  HA = 20/4 cells/mice | VE 7.074 ± 0.115  HA 7.144 ± 0.315 | Two-sided Welch’s unpaired t-test, *p*=0.8405 |
| Fig.2K | D1-MSNs sEPSC rise time (ms) | VE = 15/3 cells/mice  HA = 20/4 cells/mice | VE 1.578 ± 0.059  HA 1.632 ± 0.059 | Two-sided Welch’s unpaired t-test, *p*=0.5357 |
| Fig.3C | D2-MSNs Cumulative Probability sIPSC interevent interval | 10 events per cell | - | Kolmogorov-Smirnov test, *p*=0.1203 |
| Fig.3C inset | D2-MSNs sIPSC frequency (Hz) | VE = 15/3 cells/mice  HA = 20/4 cells/mice | VE 0.803 ± 0.086  HA 1.649 ± 0.296 | Two-sided Welch’s unpaired t-test, **p*=0.0139 |
| Fig.3D | D2-MSNs Cumulative Probability sIPSC amplitude | 10 events per cell | - | Kolmogorov-Smirnov test, *****p*<0.0001 |
| Fig.3D inset | D2-MSNs sIPSC amplitude (pA) | VE = 15/3 cells/mice  HA = 20/4 cells/mice | VE 14.848 ± 0.868  HA 16.548 ± 0.539 | Two-sided Welch’s unpaired t-test, *p*=0.1201 |
| Fig.3E | D2-MSNs sIPSC decay time (ms) | VE = 15/3 cells/mice  HA = 20/4 cells/mice | VE 9.130 ± 0.101  HA 9.123 ± 0.106 | Two-sided Welch’s unpaired t-test, *p*=0.9638 |
| Fig.3F | D2-MSNs sIPSC rise time (ms) | VE = 15/3 cells/mice  HA = 20/4 cells/mice | VE 2.278 ± 0.119  HA 2.189 ± 0.107 | Two-sided Welch’s unpaired t-test, *p*=0.5946 |
| Fig.3H | D1-MSNs Cumulative Probability sIPSC interevent interval | 10 events per cell | - | Kolmogorov-Smirnov test, ****p*=0.0006 |
| Fig.3H inset | D1-MSNs sIPSC frequency (Hz) | VE = 15/3 cells/mice  HA = 20/4 cells/mice | VE 0.599 ± 0.073  HA 1.260 ± 0.175 | Two-sided Welch’s unpaired t-test, ***p*=0.0023 |
| Fig.3I | D1-MSNs Cumulative Probability sIPSC amplitude | 10 events per cell | - | Kolmogorov-Smirnov test, *p*=0.0641 |
| Fig.3I inset | D1-MSNs sIPSC amplitude (pA) | VE = 15/3 cells/mice  HA = 20/4 cells/mice | VE 15.904 ± 0.719  HA 15.318 ± 0.705 | Two-sided Welch’s unpaired t-test, *p*=0.5764 |
| Fig.3J | D1-MSNs sIPSC decay time (ms) | VE = 15/3 cells/mice  HA = 20/4 cells/mice | VE 8.442 ± 0.090  HA 8.771 ± 0.109 | Two-sided Welch’s unpaired t-test, **p*=0.0307 |
| Fig.3K | D1-MSNs sIPSC rise time (ms) | VE = 15/3 cells/mice  HA = 20/4 cells/mice | VE 2.131 ± 0.104  HA 2.402 ± 0.111 | Two-sided Welch’s unpaired t-test, *p*=0.0948 |
| Fig.4B left | D2-MSNs I/E ratio | VE = 15/3 cells/mice  HA = 20/4 cells/mice | VE 0.476 ± 0.052  HA 0.495 ± 0.066 | Two-sided Welch’s unpaired t-test, *p*=0.8227 |
| Fig.4B right | D2-MSNs log (I/E) | VE = 15/3 cells/mice  HA = 20/4 cells/mice | VE -0.363 ± 0.049  HA -0.404 ± 0.324 | Two-sided Welch’s unpaired t-test, *p*=0.6535 |
| Fig.4C left | D1-MSNs I/E ratio | VE = 15/3 cells/mice  HA = 20/4 cells/mice | VE 0.309 ± 0.029  HA 0.468 ± 0.056 | Two-sided Welch’s unpaired t-test, **p*=0.0205 |
| Fig.4C right | D1-MSNs log (I/E) | VE = 15/3 cells/mice  HA = 20/4 cells/mice | VE -0.549 ± 0.053  HA -0.386 ± 0.049 | Two-sided Welch’s unpaired t-test, **p*=0.0348 |

| Sup. Figure | Measurement | *n* | Mean ± SEM | Statistical test and *P* value |
| --- | --- | --- | --- | --- |
| Fig.S4 | Proteins involved in regulating neuronal excitability | VE = 4 mice  HA = 5 mice | SCN2B VE 1.000 ± 0.025; HA 0.825 ± 0.025  AT1A2 VE 1.000 ± 0.046; HA 1.267 ± 0.037  AT1A3 VE 1.000 ± 0.028; HA 1.156 ± 0.030  AT1A1 VE 1.000 ± 0.022; HA 1.112 ± 0.027  AT2B4 VE 1.000 ± 0.080; HA 1.271 ± 0.051 | Two-sided Welch’s unpaired t-test (confidence level 90%)  SCN2B ****p*=0.003634  AT1A2 ****p*=0.005017  AT1A3 ***p*=0.012969  AT1A1 ***p*=0.027873  AT2B4 ***p*=0.034637 |
| Fig.S5B | Resting Potential (mV) | VE = 15/3 cells/mice  HA = 19/4 cells/mice | VE -77.625 ± 1.683  HA -76.321 ± 1.545 | Two-sided Welch’s unpaired t-test, *p*=0.5841 |
| Fig.S5C | Rheobase Current (pA) | VE = 15/3 cells/mice  HA = 19/4 cells/mice | VE 130.667 ± 9.544  HA 173.158 ± 21.778 | Two-sided Welch’s unpaired t-test, *p*=0.0949 |
| Fig.S5D | Membrane Resistance (MΩ) | VE = 15/3 cells/mice  HA = 19/4 cells/mice | VE 194.143 ± 33.693  HA 123.589 ± 17.091 | Two-sided Welch’s unpaired t-test, *p*=0.0652 |
| Fig.S5E | AP Threshold (mV) | VE = 15/3 cells/mice  HA = 19/4 cells/mice | VE -43.250± 1.313  HA -41.698± 0.888 | Two-sided Welch’s unpaired t-test, *p*=0.3523 |
| Fig.S5F | Input Resistance (MΩ) | VE = 15/3 cells/mice  HA = 19/4 cells/mice | VE 81.404 ± 6.145  HA 89.351 ± 11.398 | Two-sided Welch’s unpaired t-test, *p*=0.5559 |
| Fig.S5G | Membrane Capacitance (pF) | VE = 15/3 cells/mice  HA = 19/4 cells/mice | VE 96.640 ± 7.203  HA 93.661 ± 5.991 | Two-sided Welch’s unpaired t-test, *p*=0.7601 |
| Fig.S5H | IV curve | VE = 15/3 cells/mice  HA = 19/4 cells/mice | - | two-way repeated-measures ANOVA, p>0.9999 |
| Fig.S5I | IF curve | VE = 15/3 cells/mice  HA = 19/4 cells/mice | - | two-way repeated-measures ANOVA, **p=0.0029 |
| Fig.S5J | Current-Voltage plot | VE = 15/3 cells/mice  HA = 19/4 cells/mice | - | two-way repeated-measures ANOVA, p=0.0096 |
| Fig.S5K | Mean current response | VE = 15/3 cells/mice  HA = 19/4 cells/mice | - | two-way repeated-measures ANOVA, p>0.9999 |
| Fig.S6B | Resting Potential (mV) | VE = 15/3 cells/mice  HA = 19/4 cells/mice | VE -74.614 ± 1.834  HA -77.006 ± 0.740 | Two-sided Welch’s unpaired t-test, *p*=0.2569 |
| Fig.S6C | Rheobase Current (pA) | VE = 15/3 cells/mice  HA = 19/4 cells/mice | VE 173.333 ± 25.866  HA 183.684 ± 9.303 | Two-sided Welch’s unpaired t-test, *p*=0.7201 |
| Fig.S6D | Membrane Resistance (MΩ) | VE = 15/3 cells/mice  HA = 19/4 cells/mice | VE 111.793 ± 16.628  HA 101.211 ± 7.512 | Two-sided Welch’s unpaired t-test, *p*=0.5811 |
| Fig.S6E | AP Threshold (mV) | VE = 15/3 cells/mice  HA = 19/4 cells/mice | VE -42.163± 0.708  HA -41.998± 0.804 | Two-sided Welch’s unpaired t-test, *p*=0.8816 |
| Fig.S6F | Input Resistance (MΩ) | VE = 15/3 cells/mice  HA = 19/4 cells/mice | VE 78.711 ± 7.986  HA 74.705 ± 3.513 | Two-sided Welch’s unpaired t-test, *p*=0.6619 |
| Fig.S6G | Membrane Capacitance (pF) | VE = 15/3 cells/mice  HA = 19/4 cells/mice | VE 98.391 ± 6.814  HA 107.057 ± 4.528 | Two-sided Welch’s unpaired t-test, *p*=0.3148 |
| Fig.S6H | IV curve | VE = 15/3 cells/mice  HA = 19/4 cells/mice | - | two-way repeated-measures ANOVA, p>0.9999 |
| Fig.S6I | IF curve | VE = 15/3 cells/mice  HA = 19/4 cells/mice | - | two-way repeated-measures ANOVA, p=0.7466 |
| Fig.S6J | Current-Voltage plot | VE = 15/3 cells/mice  HA = 19/4 cells/mice | - | two-way repeated-measures ANOVA, p>0.9999 |
| Fig.S6K | Mean current response | VE = 15/3 cells/mice  HA = 19/4 cells/mice | - | two-way repeated-measures ANOVA, p>0.9999 |
