# Supplementary material for: Chronic treatment with D2-antagonist haloperidol leads to inhibitory/excitatory imbalance in striatal D1-neurons
Source: Transl Psychiatry. 2023 Oct 6;13:312. doi: 10.1038/s41398-023-02609-w (PMC10558446; doi:10.1038/s41398-023-02609-w)
Supplement: Supplementary file 4 — Supplementary Table S3 [file 41398_2023_2609_MOESM4_ESM.pdf]

Sup.Table S3 – List of all proteins ordered by p value. All individual values are normalized to the control average.

Accession Name	Protein Name	Norm WT1	Norm WT2	Norm WT3	Norm WT4	Norm Halop1	Norm Halop2	Norm Halop3	Norm Halop4	Norm Halop5	AVG KO/WT	TTEST
P50518	VATE1	0,96300661	0,987426103	1,00705504	1,042512247	1,201769441	1,165018934	1,24215071	1,265320483	1,213212888	1,217494491	4,6E-05
O08585	CLCA	0,951474781	0,996663232	1,012556915	1,039305072	0,842344273	0,754415175	0,797747691	0,752325346	0,761471822	0,781660861	5,72E-05
O09167	RL21	0,971338625	1,05135608	0,970066406	1,007238889	0,833829703	0,804865003	0,795768854	0,840030656	0,765053465	0,807909536	6,59E-05
O08599	STXB1	0,999355262	1,006092662	1,016318909	0,978233167	0,93004267	0,939735231	0,902885252	0,929473578	0,91471422	0,92337019	0,000137
Q9WUA3	PFKAP	1,009451317	0,993902121	0,975692335	1,020954228	1,21440984	1,120737179	1,16342132	1,195810202	1,243529326	1,187581573	0,000156
Q9D8B4	NDUAB	1,047381912	1,047683713	1,025104286	0,879830089	0,762187875	0,696124751	0,697160484	0,756899535	0,710935287	0,724661586	0,000204
Q8BZ98	DYN3	0,979493708	0,999855805	0,985070402	1,035580085	1,139406633	1,199648892	1,240342277	1,187439018	1,295046474	1,212376659	0,000278
Q62283	TSN7	1,031346753	0,985365844	1,027622291	0,955665112	1,422611345	1,178700523	1,388878284	1,366344529	1,41146097	1,35359913	0,000292
Q8C7X2	EMC1	0,978345776	1,023999746	0,972096705	1,025557773	0,796799514	0,844686025	0,795628404	0,883480993	0,871774986	0,838473985	0,000295
Q63810	CANB1	0,955432392	0,989975114	1,129123256	0,925469238	0,760398593	0,685045694	0,746854887	0,68372054	0,654360087	0,70607596	0,000358
P14733	LMNB1	0,993529965	0,994343696	0,961595888	1,05053045	1,203308923	1,153569854	1,148416638	1,252370082	1,267610065	1,205055112	0,000378
Q8BFP9	PDK1	1,011240784	0,976154247	0,971148953	1,041456016	0,892475102	0,818398111	0,816881193	0,88035696	0,805537019	0,842729677	0,000415
Q791T5	MTCH1	1,040925767	1,002963545	1,044382906	0,911727782	0,709632164	0,722375239	0,84814858	0,724445764	0,760463805	0,75301311	0,000419
Q8R010	AIMP2	0,964717862	0,986345181	1,001535387	1,047401571	0,897683402	0,766362946	0,768476086	0,797965771	0,799837693	0,806065179	0,000441
Q8BKZ9	ODPX	1,061994625	0,983724167	0,996280811	0,958000398	0,877028142	0,789487074	0,830675536	0,865752168	0,815733439	0,835735272	0,000462
Q5DTY9	KCD16	0,902548655	0,994387023	1,100967497	1,002096825	0,771649173	0,692812151	0,786632215	0,773027043	0,706812951	0,746186707	0,000502
P05132	KAPCA	1,023517503	1,031117468	0,93300329	1,012361739	0,892062597	0,802875508	0,821325762	0,796580162	0,771904005	0,816949607	0,000541
Q9D0M5	DYL2	0,969148596	0,924765597	1,116205131	0,989880676	0,665392729	0,748448787	0,736513224	0,806843938	0,685963378	0,728632411	0,000583
Q9DBS2	TPRGL	0,939034516	0,964958619	0,9805371	1,115469766	0,800376962	0,79888284	0,771084975	0,793446385	0,799240675	0,792606367	0,000604
P0DN34	NDUB1	0,981306132	0,928181573	1,072172986	1,018339308	0,780280744	0,821034441	0,842814414	0,741922974	0,717603827	0,78073128	0,000645
Q8R366	IGSF8	0,956297907	1,05121164	0,978133093	1,014357361	1,105420158	1,176551211	1,179232451	1,136658352	1,110978872	1,141768209	0,000854
Q9CR62	M2OM	1,050235616	0,990164702	0,963218785	0,996380898	0,788487212	0,751863091	0,872156732	0,805148131	0,67555687	0,778642407	0,000873
P63017	HSP7C	0,97115708	1,071886181	0,961460445	0,995496294	1,139839595	1,131965423	1,138275259	1,170697311	1,223077854	1,160771088	0,0009
Q60676	PPP5	1,014578614	0,91824966	1,107917433	0,959254294	0,808604891	0,756372369	0,790920788	0,795695589	0,702562127	0,770831153	0,000958
P00158	CYB	0,94943081	0,985736904	1,087926312	0,976905975	0,738727883	0,799610725	0,782466754	0,72370105	0,876268651	0,784155013	0,001083
Q8R2V5	ADAP2	1,044894965	1,001507317	1,052126538	0,90147118	0,797955404	0,505934024	0,708426379	0,55985414	0,522792736	0,618992537	0,001108
Q61990	PCBP2	0,956958987	1,007542834	1,015884241	1,019613938	0,876303615	0,872168645	0,917739512	0,813960275	0,794884978	0,855011405	0,001386
Q61171	PRDX2	0,936296808	0,968383394	1,121196052	0,974123746	0,82945376	0,807780064	0,822026006	0,800967557	0,802135094	0,812472496	0,001404
Q8BH59	CMC1	1,025319284	1,031257326	0,987297277	0,956126113	0,906671315	0,860988094	0,899485552	0,904821676	0,927483614	0,89989005	0,001437
P26369	U2AF2	0,914400657	0,963115117	1,131554477	0,990929749	0,607483214	0,598645594	0,758644785	0,805274436	0,72554832	0,69911927	0,001875
Q9JIS5	SV2A	0,984423602	1,044534551	0,950317376	1,020724471	1,242309781	1,161527178	1,11733716	1,120005594	1,247212587	1,17767846	0,001979
P99028	QCR6	1,008211568	0,891709357	1,11227272	0,987806355	0,453688933	0,663468003	0,561954607	0,641688765	0,811685712	0,626497204	0,001983
Q9CQJ8	NDUB9	1,07924261	0,932259604	1,006107802	0,982389985	0,845064187	0,710070828	0,835349846	0,656547778	0,653506113	0,740107751	0,002098
Q99LC3	NDUAA	1,039100108	0,995413497	0,996537738	0,968948657	0,879051798	0,778643569	0,857208898	0,827396616	0,685494576	0,805559091	0,002116
Q9JIG8	PRAF2	0,97223514	1,051080213	1,013492699	0,963191948	0,915209142	0,921309656	0,919068281	0,88891429	0,907759771	0,910452228	0,002162
P14824	ANXA6	0,953978246	1,034656997	0,998897825	1,012466931	1,104087853	1,066845993	1,07066019	1,069686719	1,087368042	1,079729759	0,002209
Q7TMM9	TBB2A	0,564898587	1,536250097	0,719275434	1,179575882	2,846056875	2,329444864	1,727809217	2,320054148	2,316632254	2,307999472	0,002259

Q9CQH3	NDUB5	0,978769323	1,084081548	0,934528521	1,002620608	0,814662021	0,846012865	0,898187902	0,774373503	0,770552879	0,820757834	0,002354
Q9R0Q6	ARC1A	0,998742797	0,953463181	1,006078228	1,041715794	0,935017574	0,773220906	0,812115513	0,821633658	0,754500991	0,819297728	0,002403
Q9DB77	QCR2	1,084497403	0,964943596	0,998345838	0,952213163	0,833764841	0,897140665	0,884235986	0,848805503	0,864884907	0,86576638	0,002508
Q9D4H1	EXOC2	0,99228065	0,983602455	1,052523433	0,971593461	0,859163647	0,734008121	0,746440168	0,836776534	0,906933673	0,816664429	0,002856
Q9DCZ4	MIC26	0,9532767	0,963932384	1,062243891	1,020547026	0,80005657	0,742984457	0,915662978	0,853275999	0,756351677	0,813666336	0,003255
O70252	HMOX2	1,018753582	0,989470724	0,951286359	1,040489335	1,155518671	1,104035515	1,282625089	1,329049473	1,328960561	1,240037862	0,003429
Q56A07	SCN2B	1,044631285	0,936026328	1,051422981	0,967919406	0,896365717	0,823209949	0,878791224	0,746130767	0,780470934	0,824993718	0,003634
Q8CA71	SHSA4	1,002125219	0,897945921	1,056188322	1,043740538	0,803165124	0,866165755	0,807631838	0,759404589	0,643806281	0,776034718	0,003772
Q62083	PICK1	0,997312986	1,031242135	0,991522328	0,979922551	0,828674593	0,728238746	0,795127036	0,945470276	0,835209905	0,826544111	0,003957
Q9CQZ6	NDUB3	0,962483301	0,996393229	1,017494468	1,023629002	0,757905184	0,856272771	0,938020143	0,803774449	0,733749363	0,817944382	0,003978
Q9D855	QCR7	0,913070799	1,057155453	0,950331582	1,079442165	0,681815436	0,820890815	0,859393836	0,787376943	0,655421105	0,760979627	0,004163
Q9Z2W0	DNPEP	1,0619552	0,95079104	1,011751705	0,975502055	0,858486305	0,778161599	0,798873981	0,913510662	0,891886452	0,8481838	0,004188
P07724	ALBU	1,250755298	0,714581354	0,991152422	1,043510926	0,622690075	0,518622354	0,577560566	0,457979655	0,65140571	0,565651672	0,004326
Q922Q1	MARC2	0,958996298	0,997809141	1,068405182	0,974789379	0,76395923	0,923843512	0,881850265	0,785658127	0,786795446	0,828421316	0,004332
P32037	GTR3	1,052545423	1,068037503	0,931841837	0,947575236	1,21926401	1,112889031	1,142682922	1,12892357	1,16880461	1,154512829	0,00437
P43006	EAA2	1,067323387	0,96828761	0,969613481	0,994775522	1,092552185	1,115404886	1,06016233	1,101688893	1,109837504	1,09592916	0,004394
P10126	EF1A1	0,947031708	1,094901186	0,970439966	0,987627141	1,087493762	1,180104537	1,171440084	1,270823017	1,194852907	1,180942861	0,004433
Q62188	DPYL3	0,900496323	1,06551307	0,942081283	1,091909323	1,202865314	1,147894173	1,232506102	1,299168172	1,166422778	1,209771308	0,004441
Q64516	GLPK	1,080605503	0,965060703	1,024161182	0,930172611	0,8731924	0,78341786	0,842375391	0,898656838	0,773448076	0,834218113	0,004461
P63082	VATL	0,817944451	0,746873505	1,476506766	0,958675278	1,383070281	1,734127929	1,978950079	2,017669718	2,096905115	1,842144624	0,004691
Q91YT0	NDUV1	1,065082166	0,995023373	0,960258795	0,979635666	0,868462206	0,886241117	0,949115489	0,888405468	0,855293401	0,889503536	0,004715
Q9CQX2	CYB5B	0,971259862	1,069966775	1,024844029	0,933929335	0,824716468	0,890161159	0,915865274	0,818280616	0,864822086	0,862769121	0,004763
O55029	COPB2	1,070278041	1,082012782	0,96036205	0,887347128	0,812816206	0,85671611	0,848584205	0,811624987	0,807814038	0,827511109	0,004811
Q9QXB9	DRG2	0,9534395	1,04554315	1,014417992	0,986599358	0,944265185	0,805573692	0,865936895	0,789833703	0,756472969	0,832416489	0,004838
Q8BGH2	SAM50	1,053081672	0,935852082	1,015421504	0,995644742	0,851573187	0,860021294	0,942058538	0,873008351	0,899053977	0,885143069	0,004887
Q80UW2	FBX2	1,034556386	1,019075892	0,982810852	0,963556871	0,880309815	0,834698624	0,883540752	0,827344191	0,680438494	0,821266375	0,004995
P55088	AQP4	1,023302396	1,156571571	0,841927437	0,978198596	1,387266555	1,399528346	1,538236738	1,291080813	1,176002872	1,358423065	0,00501
Q6PIE5	AT1A2	1,064829953	1,083437752	0,848808271	1,002924023	1,293358731	1,413300379	1,251011123	1,191287175	1,187109297	1,267213341	0,005017
P62141	PP1B	0,974732939	0,997952771	1,015668774	1,011645516	1,066666162	1,115767996	1,170969321	1,049052154	1,106704482	1,101832023	0,005141
O35857	TIM44	1,041125328	0,89512984	1,127189007	0,936555825	0,804344315	0,804874011	0,796997745	0,744199945	0,608383523	0,751759908	0,005464
Q8R1N4	NUDC3	1,058874398	0,756181271	1,126909516	1,058034815	0,689744109	0,522812857	0,777665334	0,678929419	0,573265167	0,648483377	0,005528
Q61206	PA1B2	0,937924907	0,946331841	1,084680691	1,031062562	0,850590722	0,823830842	0,899934802	0,887980523	0,795435945	0,851554567	0,005846
P61922	GABT	1,013333436	1,047175354	0,964642167	0,974849043	1,084578718	1,154582039	1,093165261	1,221364526	1,097317473	1,130201603	0,006235
Q8BH95	ECHM	0,981444905	0,992524021	0,91622357	1,109807505	1,104552581	1,179407641	1,288231219	1,366596425	1,245840209	1,236925615	0,006541
Q60930	VDAC2	1,033982981	1,006661979	0,947955049	1,011399991	0,867034342	0,896665943	0,914740745	0,798485563	0,731348513	0,841655021	0,006758
Q9D0K2	SCOT1	0,948716245	0,973738333	1,030881371	1,046664051	1,076802998	1,091380291	1,154853904	1,233735428	1,147356489	1,140825822	0,007111
Q02053	UBA1	0,939358351	1,058047324	0,999020587	1,003573738	1,150211679	1,109330059	1,075648541	1,076430665	1,181071039	1,118538397	0,007314
P62631	EF1A2	0,918845039	1,104691426	0,925905422	1,050558113	1,162886373	1,171510238	1,155108538	1,154810217	1,125740518	1,154011177	0,007536
Q9DC69	NDUA9	1,033225815	0,949074774	0,947960752	1,069738658	0,825698426	0,863914909	0,845121408	0,729956846	0,604074759	0,77375327	0,00757
P48962	ADT1	1,133593882	0,957066755	0,946928858	0,962410505	0,817618725	0,850989086	0,883764804	0,829634244	0,744271065	0,825255585	0,007625

P46664	PURA2	0,94514762	1,024574963	1,056081283	0,974196134	0,868658407	0,838548555	0,89959746	0,925794186	0,927171882	0,891954098	0,007652
Q9CZY3	UB2V1	0,966721694	1,033027164	1,009625114	0,990626028	1,155757456	1,202929664	1,29595954	1,131003364	1,061369734	1,169403952	0,007795
Q99JW2	ACY1	1,038765079	0,967552276	0,972636868	1,021045777	0,917637358	0,649307389	0,752208684	0,873683289	0,780904683	0,79474828	0,007798
Q8BGU5	CCNY	0,936172654	0,963018875	1,040738393	1,060070078	0,857873676	0,699144564	0,911723511	0,84133197	0,809895987	0,823993942	0,007891
O55143	AT2A2	1,066520986	1,005641225	1,000267899	0,92756989	1,29207102	1,168887097	1,084857025	1,142754337	1,293886192	1,196491134	0,007898
Q8VEK3	HNRPU	0,892317591	1,121887104	0,985047612	1,000747693	1,228065886	1,25882185	1,277197046	1,280028034	1,614545647	1,331731693	0,008159
Q9DC70	NDUS7	0,985407578	1,01618463	0,999520389	0,998887403	0,867099595	0,911793983	0,937360667	0,874748478	0,753865411	0,868973627	0,008404
Q921G7	ETFD	1,073285787	1,060249984	0,969685518	0,896778711	0,811381535	0,821710329	0,921364162	0,757677617	0,83585412	0,829597553	0,008589
P50516	VATA	0,950945133	1,021873859	1,002879401	1,024301608	1,093402324	1,038487214	1,077740697	1,083313376	1,143117999	1,087212322	0,008733
Q01853	TERA	0,970566449	1,016391586	1,014156772	0,998885193	1,123488901	1,08218043	1,067181478	1,076142029	1,209370704	1,111672708	0,008972
P10639	THIO	0,898803149	1,010654077	1,134624273	0,955918501	0,790625363	0,887569786	0,819618196	0,809701105	0,706122057	0,802727301	0,009067
P35279	RAB6A	0,92669092	0,933099217	1,065249506	1,074960357	1,232319888	1,174806216	1,432572928	1,55704172	1,216906374	1,322729425	0,009238
O35129	PHB2	1,036006317	0,967943504	0,982325718	1,013724461	0,936091191	0,9208467	0,95884887	0,906343606	0,854665954	0,915359264	0,009592
O54774	AP3D1	0,743587287	1,025370218	1,076303044	1,154739451	1,368540272	1,206853918	1,247900565	1,393041543	1,46113614	1,335494488	0,009607
P62900	RL31	0,857585051	1,061210985	1,027918422	1,053285542	0,895799439	0,76710643	0,854472422	0,799981793	0,723712354	0,808214488	0,009817
P53612	PGTB2	1,007916654	0,958031328	0,856336849	1,177715169	0,779192998	0,823767775	0,681492002	0,657787328	0,474016542	0,683251329	0,009997
Q60598	SRC8	0,958939986	0,988219934	1,004243304	1,048596775	0,86140998	0,718922131	0,804023753	0,719657265	0,956728681	0,812148362	0,010012
P99026	PSB4	0,912976814	0,952639631	1,124646667	1,009736888	1,336143462	1,076743056	1,612630411	1,509009536	1,398646294	1,386634552	0,010043
O35864	CSN5	1,01912054	0,960552743	0,971547412	1,048779306	0,915584259	0,893006922	0,855622596	0,750120697	0,699886093	0,822844114	0,010149
Q9D6F9	TBB4A	1,052505595	1,041780621	0,948838887	0,956874898	1,196774335	1,072050335	1,169730138	1,173735073	1,069138527	1,136285682	0,010202
P62204	CALM	0,71588963	0,790687804	1,502077818	0,991344748	0,48573376	0,447335006	0,474066835	0,387460463	0,474758632	0,453870939	0,01026
P59999	ARPC4	0,86717292	1,00899309	1,075009849	1,048824141	0,817271871	0,714930432	0,893798035	0,836201505	0,834820874	0,819404543	0,01074
Q9EPK7	XPO7	1,091488715	0,972784774	0,996003241	0,939723269	0,901656523	0,885252644	0,836733349	0,832377921	0,929655917	0,877135271	0,010809
P70670	NACAM	0,984520614	1,008868637	1,015023062	0,991587688	0,979199857	0,927850487	0,946965644	0,876275671	0,925760415	0,931210415	0,010855
O88935	SYN1	0,979277764	1,018726338	1,007050248	0,994945649	1,141501487	1,023873727	1,081664589	1,071620715	1,146072937	1,092946691	0,010909
Q99MN1	SYK	0,993705105	0,982113333	0,985413236	1,038768326	1,16379835	1,037732956	1,128167628	1,130763023	1,258314135	1,143755218	0,010919
Q91ZZ3	SYUB	0,93982665	0,994804905	1,054756796	1,010611649	0,854451319	0,965323186	0,900624326	0,819307474	0,856271559	0,879195573	0,011071
Q8BLQ9	CADM2	1,047295505	1,01363308	1,000110454	0,938960962	1,149241225	1,07840875	1,047314891	1,119360957	1,083753687	1,095615902	0,011562
Q8C5H8	NAKD2	1,006303918	1,109665859	0,873428102	1,010602122	0,819755212	0,786814708	0,914867042	0,802164836	0,83699572	0,832119504	0,011616
Q8R574	KPRB	0,899741041	1,036544219	0,981701008	1,082013733	0,831822818	0,788042597	0,765015343	0,886593218	0,920337028	0,838362201	0,011663
Q62420	SH3G2	1,097827973	0,955477925	0,938473602	1,0082205	1,195898162	1,070177837	1,171954142	1,139376715	1,106356891	1,136752749	0,011804
P50396	GDIA	0,922081105	1,065600901	0,989914276	1,022403719	1,105898541	1,054517811	1,191673705	1,245218717	1,215417976	1,16254535	0,012032
Q8C1A5	THOP1	0,955556571	0,968046294	0,988329385	1,08806775	1,15997125	1,135754161	1,044557951	1,166015066	1,106051817	1,122470049	0,012139
P62334	PRS10	0,902204271	1,018251057	1,053719275	1,025825398	1,127944492	1,064539379	1,125511547	1,185546419	1,112050961	1,12311856	0,012143
P20108	PRDX3	0,843625502	0,858548229	1,242244738	1,055581532	0,738799065	0,700334277	0,731325811	0,751723343	0,664825848	0,717401669	0,012358
O55023	IMPA1	0,938283561	0,896306828	1,10030248	1,065107132	0,862866715	0,840855996	0,878623852	0,811724075	0,723722584	0,823558644	0,012705
Q9Z0X1	AIFM1	1,111438547	1,01026467	0,938031921	0,940264862	0,929842742	0,811375106	0,876168469	0,764088024	0,761149367	0,828524741	0,012735
Q8BMF4	ODP2	1,021837223	1,047326568	0,945896641	0,984939568	0,93579761	0,848034682	0,931172269	0,896393042	0,935021261	0,909283773	0,012812
Q6PIC6	AT1A3	1,047442386	1,015961224	0,906583313	1,030013077	1,153965851	1,284806088	1,119934065	1,094347467	1,12756107	1,156122908	0,012969
A2A690	TANC2	0,839512991	0,890370853	1,179669593	1,090446564	0,760339554	0,718967383	0,822175841	0,751780078	0,723927433	0,755438058	0,012987

Q8CAE9	PDXL2	0,944152101	1,032003694	1,042663373	0,981180833	0,904252372	0,78452632	0,759697164	0,933827046	0,890640016	0,854588584	0,013192
P12960	CNTN1	0,9737065	1,029872167	0,983471105	1,012950227	1,111401759	1,092383635	1,061581259	1,014712273	1,114182885	1,078852362	0,013276
Q9Z1Z2	STRAP	1,009088496	0,910274212	1,094017948	0,986619345	0,900107712	0,79821552	0,89624181	0,85861062	0,72226107	0,835087347	0,013829
Q99LY9	NDUS5	0,997067113	0,958265701	1,01478928	1,029877906	0,818164017	0,90922164	0,974744998	0,78549402	0,784710169	0,854466969	0,014134
Q6P069	SORCN	0,957477958	1,038010463	0,980791142	1,023720437	0,838798717	0,752611168	0,972360355	0,80648265	0,667051416	0,807460861	0,01434
P47754	CAZA2	0,951754986	0,978571089	1,003229806	1,066444118	0,958450378	0,896786127	0,909690324	0,816615068	0,862034027	0,888715185	0,014496
Q3V0K9	PLSI	1,084300363	1,12900937	1,004913919	0,781776348	0,833070666	0,685297275	0,833876473	0,69772225	0,65607176	0,741207685	0,014721
P97797	SHPS1	1,025205484	0,947352345	1,088508386	0,938933784	0,82854398	0,852410534	0,750593592	0,775321926	0,960671642	0,833508335	0,014808
Q61205	PA1B3	0,880641031	0,926583248	1,01900526	1,173770461	0,769544412	0,861078743	0,826673483	0,809882658	0,723622308	0,798160321	0,014845
Q9CZ13	QCR1	1,048758097	0,997018413	0,973333048	0,980890442	0,907787519	0,895289476	0,975607905	0,936738556	0,937580963	0,930600884	0,015183
P61087	UBE2K	0,959548242	0,993570615	0,995913964	1,05096718	0,828756975	0,89383211	0,936549226	0,706379469	0,678306773	0,808764911	0,015189
P47857	PFKAM	1,033810364	1,038909846	0,993615064	0,933664725	1,12741852	1,050006938	1,095483447	1,053602828	1,121632263	1,089628799	0,015756
P67778	PHB	1,062381525	0,985855402	0,983458746	0,968304327	0,906849886	0,955057648	0,933208956	0,842796854	0,825878383	0,892758346	0,016201
P39053	DYN1	0,905026286	1,06624099	0,994685464	1,03404726	1,187969647	1,05854399	1,106181816	1,151522932	1,24559918	1,149963513	0,016361
Q61316	HSP74	0,963432325	1,057242489	0,9919571	0,987368086	1,103853971	1,064490296	1,080372679	1,035584172	1,147575444	1,086375312	0,017034
P14106	C1QB	0,993185865	1,04801382	0,991083875	0,96771644	0,796748139	0,886649475	0,940018034	0,759084164	0,934596283	0,863419219	0,017425
Q9DCN2	NB5R3	0,896807695	0,96862821	1,093047285	1,04151681	0,801556318	0,905014696	0,890486534	0,712267756	0,655166789	0,792898419	0,017446
P97427	DPYL1	0,932414089	1,042442845	1,003819049	1,021324017	1,10235649	1,025666233	1,125750134	1,199597298	1,191131978	1,128900427	0,017584
P99024	TBB5	1,050592748	0,916964265	0,965236673	1,067206314	1,047746349	1,180025473	1,156481566	1,316074341	1,187277736	1,177521093	0,01788
P04925	PRIO	0,985728151	0,984658394	0,908985088	1,120628368	1,116380956	1,117385105	1,362487655	1,272311997	1,161717695	1,206056682	0,018027
Q9QVP9	FAK2	0,777924125	1,090079205	1,055070877	1,076925793	0,738795001	0,586377335	0,772196772	0,819421988	0,828726402	0,7491035	0,018403
P01942	HBA	1,37016108	0,615321758	0,931848561	1,082668601	0,655185043	0,653339635	0,547207642	0,366806182	0,457145374	0,535936775	0,018506
Q99LD4	CSN1	0,996182087	1,036306456	0,986174893	0,981336565	1,188189745	1,081229784	1,012379436	1,123886331	1,173831019	1,115903263	0,018535
Q9CZX8	RS19	0,928678982	0,855150262	1,107339088	1,108831668	1,264387371	1,477223736	1,244011812	1,18443666	1,143379292	1,262687774	0,018854
Q99LR1	ABD12	1,023777464	0,993714993	1,013654045	0,968853498	0,897761265	0,904666471	1,004104266	0,877412559	0,900800772	0,916949067	0,019163
Q9WTX5	SKP1	0,905937159	0,952034563	1,081171496	1,060856782	0,865217981	0,861380547	0,929262091	0,795032428	0,719882216	0,834155052	0,019272
Q62433	NDRG1	1,102948012	1,094094135	0,869980789	0,932977064	1,273086156	1,074716761	1,253428522	1,319404908	1,158501211	1,215827512	0,019397
Q60631	GRB2	0,998841012	1,037336231	0,904324759	1,059497999	1,157806485	1,090026227	1,163696666	1,097195835	1,060810179	1,113907078	0,019451
Q9R0Q7	TEBP	0,96107025	0,977282423	0,979299671	1,082347656	0,901969779	0,715504005	0,950795706	0,748330234	0,658316125	0,79498317	0,019645
P15532	NDKA	0,893490315	1,037359595	1,093807359	0,975342732	0,870224682	0,842778779	0,917439592	0,876328621	0,749159085	0,851186152	0,019651
P05201	AATC	1,029779164	0,886436064	1,01903004	1,064754732	0,916306109	0,790581091	0,879679779	0,915986711	0,84135416	0,86878157	0,020016
P00397	COX1	1,265429552	1,003443233	0,901926846	0,829200369	0,818096628	0,539082994	0,658859238	0,647483836	0,800863633	0,692877266	0,020056
P68181	KAPCB	0,995596228	0,895095032	1,110203946	0,999104794	1,307455778	1,145707225	1,097302397	1,11324884	1,28984789	1,190712426	0,02029
Q9QUP5	HPLN1	0,841477135	1,047367559	1,058637489	1,052517818	0,90824425	0,894372048	0,780932184	0,772715358	0,783623203	0,827977408	0,020365
Q8BJH1	ZC21A	0,908507889	1,024224626	1,123080985	0,944186501	1,101631993	1,134262543	1,154139887	1,110582026	1,223951838	1,144913657	0,020437
Q80WJ7	LYRIC	0,883915226	1,113580366	0,937872042	1,064632366	0,826411677	0,763886693	0,855932172	0,765908788	0,924746626	0,827377191	0,020783
Q61838	PZP	1,383695262	0,8179837	0,852357742	0,945963296	0,797587882	0,572995619	0,616033802	0,488323618	0,618899084	0,618768001	0,020784
Q9Z2I0	LETM1	0,978188908	1,04037356	1,049416135	0,932021397	0,935899734	0,930591239	0,916498682	0,899375149	0,937820199	0,924037001	0,020944
Q8BGZ1	HPCL4	0,800401081	0,934205405	1,130408412	1,134985103	1,083915108	1,306016387	1,593406479	1,416065843	1,346521492	1,349185062	0,020957
P08228	SODC	0,783052282	0,81136058	1,236519924	1,169067214	0,639256088	0,643605902	0,786614728	0,662903956	0,664912313	0,679458597	0,021015

Q9QYF9	NDRG3	1,010736111	0,974489547	1,007253288	1,007521054	0,958837879	0,932165084	0,993619949	0,957008458	0,922365762	0,952799426	0,021018
Q921I1	TRFE	1,200299	0,978056773	0,854536688	0,967107539	0,848195394	0,819629817	0,79377731	0,685750901	0,802682672	0,790007219	0,021026
Q9D710	TMX2	0,993522964	0,992720816	0,998883899	1,014872322	0,912914168	0,991977114	0,867998829	0,874039902	0,785467565	0,886479516	0,021039
Q8VDP6	CDIPT	0,932573193	0,956332887	1,157256944	0,953836976	1,110102178	1,129340671	1,17325889	1,244511117	1,135682121	1,158578995	0,021101
Q9DCS3	MECR	0,92087154	1,089798299	0,937501901	1,05182826	0,946933219	0,849305263	0,761491077	0,596489008	0,693676228	0,769578959	0,021223
P84075	HPCA	0,905341857	0,957001639	0,946102023	1,191554481	0,645732411	0,646802373	0,811468173	0,864579248	0,8563864	0,764993721	0,021548
Q9D0M1	KPRA	0,97920312	0,905756653	0,951984307	1,16305592	0,899349047	0,807635309	0,839621943	0,864492827	0,782962842	0,838812393	0,021583
Q80TZ3	AUXI	0,975398447	0,978414588	1,017245779	1,028941186	1,106632318	1,065305772	1,027211671	1,053576664	1,152711101	1,081087505	0,021959
O35136	NCAM2	0,937745908	1,011590004	1,033675334	1,016988754	1,171041891	1,129233797	1,094147986	1,013037915	1,100082257	1,101508769	0,022639
O70194	EIF3D	0,944504996	1,051976328	1,012338819	0,991179857	0,961430088	0,950315798	0,91965494	0,917089275	0,932222403	0,936142501	0,02273
Q9EQF6	DPYL5	0,960971613	1,080169279	0,906363942	1,052495166	1,178234225	1,12733123	1,245535417	1,247619531	1,034818924	1,166707865	0,022901
Q6PHN9	RAB35	0,970541981	0,98611095	0,923627354	1,119719716	1,045996589	1,288466961	1,294533978	1,223507912	1,112867187	1,193074525	0,023443
P18242	CATD	0,962093041	0,988055983	1,029339692	1,020511285	0,728997313	0,878652505	0,999324485	0,803666701	0,829477729	0,848023746	0,023487
P51881	ADT2	1,106923332	0,937780593	0,977590801	0,977705274	0,909569041	0,928583909	0,911751772	0,844783842	0,810359977	0,881009708	0,023793
P62827	RAN	0,930302654	0,984158278	1,048007609	1,037531459	0,905795708	0,824195468	0,915717938	0,899284712	0,960286849	0,901056135	0,023965
P24288	BCAT1	0,94616751	0,955631375	1,070711885	1,027489229	0,907944216	0,936561152	0,921924997	0,901576061	0,808825419	0,895366369	0,024075
P45878	FKBP2	0,938605267	1,00756607	1,08900613	0,964822532	1,063510825	1,128102002	1,11628048	1,078202767	1,218271092	1,120873433	0,024108
Q9QZM0	UBQL2	0,868850896	1,020704522	1,024829458	1,085615124	0,825626029	0,788876649	0,780545307	0,796845819	0,977515418	0,833881844	0,024372
Q9R0P9	UCHL1	0,917534421	0,908712766	1,048825016	1,124927797	0,783259898	0,724121198	0,962694679	0,800388711	0,809750679	0,816043033	0,024403
Q05BC3	EMAL1	1,157372462	0,884882067	0,905642293	1,052103178	1,54608096	1,285245531	1,318169529	1,115797564	1,1784434	1,288747397	0,024418
Q99JR1	SFXN1	1,019658182	0,998245643	1,032637659	0,949458516	0,851136523	0,932802398	0,974251374	0,937933267	0,8765773	0,914540173	0,024436
Q4VAE3	TMM65	1,001091721	1,049440025	1,063906639	0,885561616	0,836061675	0,827901564	0,82531185	0,779373269	0,460968126	0,745923297	0,024469
Q9JII6	AK1A1	0,915703144	0,919191653	1,119075611	1,046029592	0,911219336	0,753147523	0,893194981	0,845348493	0,718472949	0,824276656	0,024522
P08113	ENPL	0,97701001	1,007222986	1,0090052	1,006761804	0,913102493	0,92664795	0,975342368	0,870908623	0,976671042	0,932534495	0,02458
Q91V92	ACLY	0,928830198	1,06058182	0,984043085	1,026544897	1,212769351	1,064627076	1,064694865	1,128246205	1,261009157	1,146269331	0,024631
P02088	HBB1	1,412300037	0,697448865	0,89150046	0,998750638	0,739622439	0,645135636	0,579721957	0,46201447	0,545992355	0,594497371	0,025032
Q60865	CAPR1	1,145016518	0,396396721	1,46700868	0,991578082	1,522475779	1,335189768	1,650366114	1,869179489	1,77890575	1,63122338	0,025935
Q8VDD5	MYH9	0,924055554	1,104136663	0,952043539	1,019764245	0,864189394	0,879628267	0,881492324	0,72649605	0,9218031	0,854721827	0,026263
P62983	RS27A	0,936162162	1,072406616	1,005652007	0,985779215	0,980024951	0,901216075	0,890110842	0,885859876	0,897860842	0,911014517	0,026328
Q60996	2A5G	0,945375857	0,976251214	0,987279641	1,091093289	1,140934482	1,149816869	1,137577498	1,09688131	1,019687995	1,108979631	0,026681
D3Z7P3	GLSK	0,955402786	0,972956979	1,052116652	1,019523582	1,047426987	1,031375082	1,076225995	1,124896577	1,113165731	1,078618074	0,02696
Q8VDN2	AT1A1	1,016445218	0,977569294	1,062172499	0,943812989	1,146323342	1,051171253	1,074857097	1,074878233	1,212103058	1,111866597	0,027873
P70372	ELAV1	0,881480883	1,005674491	1,040763368	1,072081259	0,954533823	0,84845923	0,892020788	0,853867873	0,860532538	0,88188285	0,02846
O35643	AP1B1	0,98676354	1,00471116	1,015434008	0,993091292	1,12434328	0,98336469	1,09692343	1,172978296	1,176603765	1,110842692	0,028557
Q9CPU0	LGUL	0,986103864	0,870466575	0,987104878	1,156324683	0,79336225	0,780328873	0,946771268	0,831799794	0,697988002	0,810050037	0,028691
P18760	COF1	0,952045097	1,034091191	0,952252891	1,061610821	1,060899788	1,108576953	1,035272026	1,134822825	1,096197334	1,087153785	0,028894
P23492	PNPH	1,050350716	0,884335612	0,961309846	1,104003826	0,884353478	0,889780695	0,890960517	0,738856273	0,668241892	0,814438571	0,02923
Q99JI6	RAP1B	0,93278354	0,985753161	0,973569648	1,10789365	1,036662496	1,157553123	1,376276679	1,45162634	1,219693391	1,248362406	0,029284
Q791V5	MTCH2	1,042467515	0,984200726	0,973993156	0,999338603	0,91669373	0,953830045	0,96984193	0,875752782	0,815569594	0,906337616	0,029306
Q9CXZ1	NDUS4	1,041964567	0,982057168	0,937598072	1,038380193	0,801940422	0,856315351	1,008855924	0,785408533	0,698744452	0,830252936	0,029334

P49615	CDK5	1,008594843	0,901634503	1,008605135	1,08116552	0,868360049	0,934742839	0,931008425	0,786843585	0,74945286	0,854081552	0,029426
O08547	SC22B	0,975900877	0,998095688	1,004552492	1,021450942	1,06151424	1,07279953	1,39677777	1,28438021	1,177934403	1,19868123	0,029587
Q62443	NPTX1	0,967962077	0,905141418	1,028203367	1,098693138	0,839442811	0,684701189	0,992695985	0,778874207	0,774520331	0,814046904	0,029723
Q3U1J4	DDB1	0,922947279	1,084922056	0,990864369	1,001266297	0,920373932	0,880931164	0,866951375	0,858741775	0,969685993	0,899336848	0,030747
Q99P72	RTN4	1,044532128	1,025920629	0,978415007	0,951132236	1,218965369	1,073380785	1,077815817	1,05402439	1,093479955	1,103533263	0,030858
P13595	NCAM1	0,909773664	1,084660789	0,942525377	1,06304017	1,055801304	1,110798276	1,111073803	1,152161362	1,139278749	1,113822699	0,031599
Q8BTG7	NDRG4	0,927907697	1,057957596	1,017925495	0,996209212	1,17556568	0,991098167	1,361402507	1,187522096	1,267834868	1,196684664	0,031612
Q8R164	BPHL	0,95914469	0,976815316	1,037200721	1,026839272	1,021749476	1,083054375	1,078583111	1,052390963	1,043634502	1,055882486	0,032707
Q9CY27	TECR	1,095045411	1,037518324	0,866201125	1,001235141	0,930160817	0,854022968	0,866503125	0,768138792	0,889046565	0,861574453	0,033196
Q9D0I9	SYRC	0,98480924	1,083622317	0,963447365	0,968121078	0,90723248	0,896764679	0,930167463	0,827532446	0,966626678	0,905664749	0,034125
P16858	G3P	0,962965479	1,045915666	0,971509505	1,019609349	1,08437592	1,060121774	1,073727164	1,049048208	1,01746461	1,056947535	0,03434
Q6Q477	AT2B4	0,780903516	0,986762343	1,231941565	1,000392577	1,203675457	1,14896592	1,225524061	1,298776862	1,477407768	1,270870014	0,034637
P06151	LDHA	1,013254293	0,925257986	1,011688642	1,049799078	0,976427614	0,92310254	0,881132528	0,831987082	0,762232187	0,87497639	0,034711
Q64521	GPDM	0,863381095	1,080793582	1,010933985	1,044891339	0,859758415	0,859737803	0,914694225	0,892209812	0,902029315	0,885685914	0,034877
Q60771	CLD11	1,020304495	1,152587749	0,935989752	0,891118004	1,222087735	1,531134452	1,585409615	1,152636659	1,111851807	1,320624053	0,035152
P28738	KIF5C	0,916919192	1,096407838	0,960757547	1,025915424	1,186710379	1,040920929	1,066246694	1,153468633	1,186918559	1,126853039	0,036035
Q80UJ7	RB3GP	1,07174297	0,962775031	1,010108395	0,955373604	0,938457019	0,924459736	0,756860611	0,821152926	0,937928689	0,875771796	0,036183
Q9CQ89	CUTA	0,85970381	0,929827955	1,233384414	0,97708382	0,564994287	0,060597282	0,772660053	0,737948188	0,716260751	0,570492112	0,036262
P50114	S100B	0,851345039	0,922019717	1,240925627	0,985709617	0,669509373	0,457947191	0,820619156	0,904882412	0,682366492	0,707064925	0,037158
Q9D8E6	RL4	0,943085472	1,020830883	0,997825223	1,038258422	0,927134663	0,88098847	0,985340434	0,887805305	0,951045014	0,926462777	0,037222
O88456	CPNS1	0,978713556	1,031068399	0,864015425	1,12620262	1,17120846	1,271268397	1,198175585	1,140429531	1,041574409	1,164531276	0,03739
P49442	INPP	0,974785176	0,935218134	1,000856242	1,089140448	0,901136777	0,792354182	0,845526408	0,990656287	0,868619098	0,87965855	0,037747
Q0GNC1	INF2	0,83477556	1,21645094	0,92294748	1,025826021	1,384884969	1,132333989	1,100925499	1,30421892	1,487706233	1,282013922	0,03776
P27048	RSMB	0,898629474	1,031302167	1,038325147	1,031743213	0,887064572	0,847697115	0,990615338	0,876187335	0,881068725	0,896526617	0,038376
Q62425	NDUA4	0,946190406	1,035412954	1,055435734	0,962960906	0,882405974	0,918409254	0,944837439	0,9742833	0,863966362	0,916780466	0,038508
Q9CZC8	SCRN1	1,004522088	0,870100473	1,095450801	1,029926638	0,880060105	0,848414428	0,930926402	0,898687852	0,879727577	0,887563273	0,038838
Q11136	PEPD	0,909354545	0,816508928	1,06754561	1,206590917	0,874737706	0,605025032	0,806444152	0,753251852	0,801463968	0,768184542	0,038845
P32067	LA	0,881682733	0,996392825	1,130967265	0,990957176	0,770989063	0,652518671	0,914728398	0,92054942	0,827305425	0,817218195	0,038881
Q8K0D5	EFGM	0,962547922	1,051885538	1,094270034	0,891296505	1,139956628	1,119283	1,129432888	1,086207065	1,065719332	1,108119783	0,039897
Q9JKK7	TMOD2	1,007887665	1,009871114	0,994519758	0,987721464	0,974526505	0,93359083	0,972922819	0,991871771	0,914978407	0,957578066	0,040162
Q5SRX1	TM1L2	0,987965322	0,991084899	1,046980127	0,973969653	0,91913938	0,865288504	1,00479508	0,937120375	0,897680281	0,924804724	0,04044
P17426	AP2A1	0,965852564	1,015516031	1,027703429	0,990927976	1,059096444	1,034624906	1,049985181	1,051853484	1,16040467	1,071192937	0,040445
P11499	HS90B	0,953572271	1,105152777	0,952367801	0,988907151	1,098160191	1,111021974	1,05776572	1,052963512	1,132611793	1,090504638	0,040814
P17427	AP2A2	0,965659744	0,972579426	1,031504521	1,030256309	1,09542471	1,046588192	1,067218485	1,121390008	1,273874338	1,120899146	0,040816
Q04447	KCRB	0,923501102	0,828364851	1,258684253	0,989449794	0,864340077	0,709118725	0,716658999	0,824837972	0,783698018	0,779730758	0,040841
Q8VEM8	MPCP	1,138852058	0,980543413	0,964139317	0,916465211	0,890465237	0,933303757	0,897318296	0,863851449	0,837785702	0,884544888	0,040848
Q8C0L0	TMX4	0,942647905	0,936205748	1,084309687	1,03683666	0,945387881	0,829427456	0,927975169	0,893013736	0,914956098	0,902152068	0,041204
Q99020	ROAA	0,924321157	0,992724171	1,060108369	1,022846303	0,940008921	0,654356989	0,862027778	0,827738717	0,925864986	0,841999478	0,041277
P23927	CRYAB	0,934084881	1,084102423	0,828262963	1,153549732	1,210264782	1,293307343	1,375994014	1,194368851	1,045597308	1,22390646	0,041299
P21619	LMNB2	0,940573941	0,987843963	1,080278179	0,991303917	0,878595187	0,792841847	0,94015674	0,787282826	0,975941885	0,874963697	0,041339

P62838	UB2D2	0,890480476	1,095527039	1,098778616	0,915213868	0,844166184	0,890453039	0,918292375	0,865635891	0,817567066	0,867222911	0,041565
P14685	PSMD3	0,978301843	0,958501139	1,011618869	1,051578148	1,096029386	1,013978618	1,210242031	1,144558276	1,061525993	1,105266861	0,041821
P58389	PTPA	0,958220396	1,031235217	0,971715458	1,03882893	0,996240916	0,915037582	0,921105271	0,884482377	0,811247007	0,905622631	0,043558
Q6P1F6	2ABA	0,968211237	0,999575368	1,06854729	0,963666104	1,053176594	1,004364346	1,137776832	1,176471699	1,179079403	1,110173775	0,043913
Q3UGR5	HDHD2	1,077755117	0,875839006	0,985977914	1,060427963	0,925714756	0,632595887	0,894991018	0,887368661	0,723884091	0,812910882	0,044147
Q9EPJ9	ARFG1	1,042789356	0,922520479	1,032078402	1,002611763	0,996933529	0,861012586	0,854358039	0,913146786	0,914840818	0,908058352	0,044219
O88343	S4A4	0,890533455	1,190128432	0,938331158	0,981006955	1,245551458	1,138007193	1,189404789	1,081938978	1,14773734	1,160527952	0,044611
P70168	IMB1	0,996892663	1,059342248	0,998511373	0,945253716	1,068768954	1,033856521	1,05513955	1,047838249	1,122927455	1,065706146	0,044612
Q91VM9	IPYR2	0,996854827	0,997106716	0,995551182	1,010487275	0,952407564	0,560354298	0,915112732	0,814851221	0,811072867	0,810759736	0,044975
P22892	AP1G1	0,961220107	1,02635819	0,991209033	1,02121267	0,97652765	0,911177941	0,927451955	0,88740882	0,988422236	0,93819772	0,046071
O70133	DHX9	0,94439966	1,121000024	1,038633784	0,895966532	0,816032781	0,732207176	0,752285698	0,949764483	0,93426631	0,83691129	0,046294
Q8BP92	RCN2	0,952546585	0,9993924	1,077268491	0,970792524	0,829526106	1,012985685	0,901369133	0,79599798	0,873722724	0,882720326	0,046742
P70195	PSB7	1,034509975	0,692489722	1,045871388	1,227128914	0,869459061	0,698355193	0,61614233	0,53866064	0,041944617	0,552912369	0,046889
Q8BH66	ATLA1	1,02436014	1,096344495	0,981083222	0,898212143	0,83795114	0,890982688	0,97968586	0,891503331	0,837639187	0,887552441	0,047256
Q9JI46	NUDT3	0,789433205	0,914016432	1,363284919	0,933265445	0,679168475	0,535235128	0,729916917	0,84913417	0,717957682	0,702282475	0,047398
Q3UJU9	RMD3	1,025951979	1,062088935	1,02276254	0,889196546	0,889852463	0,957661136	0,84751716	0,916818044	0,917454501	0,905860661	0,047426
P62082	RS7	0,864368117	0,956541631	1,045330801	1,133759451	0,858878849	0,747284969	0,949205979	0,823179961	0,867246176	0,849159187	0,047832
P63028	TCTP	0,998685207	1,000229177	0,998892809	1,002192807	0,967729669	0,987432948	0,982018688	0,936548849	0,896008597	0,95394775	0,048039
P17751	TPIS	0,882751791	0,851369476	1,103790909	1,162087824	0,728117321	0,874559526	0,869990973	0,82960754	0,803667141	0,8211885	0,048248
Q8VDM4	PSMD2	0,879259576	1,026979798	1,059589169	1,034171456	1,075581766	1,041211422	1,110733293	1,1221289	1,169274229	1,103785922	0,048387
Q8QZS1	HIBCH	0,94893495	1,054856811	0,958327554	1,037880685	1,108506334	1,025851418	1,069143376	1,204996699	1,084092144	1,098517994	0,048636
Q9D5V5	CUL5	0,885655736	1,042324821	1,038292747	1,033726696	0,884911873	0,813221817	0,886435397	0,862815698	0,992517611	0,887980479	0,049308
Q9CRB9	MIC19	0,94340385	0,958928993	1,114556451	0,983110706	0,930684698	0,868924569	0,971444547	0,776595566	0,75426303	0,860382482	0,049519
Q7TQI3	OTUB1	0,936105927	1,033488138	1,011748918	1,018657017	0,988206266	0,906942336	0,937247813	0,869451355	0,783883741	0,897146302	0,049559
Q6IRU5	CLCB	0,927305948	0,993758195	1,030093961	1,048841895	0,97677018	0,840859088	0,925382934	0,919055323	0,763093597	0,885032224	0,04968
Q9CPQ3	TOM22	1,02179713	0,921434907	1,086678006	0,970089956	1,194934148	1,102489476	1,847436166	1,38092124	1,263086049	1,357773416	0,049978
P03888	NU1M	1,209704556	0,99801048	0,948731595	0,843553369	0,929356467	0,851321916	0,727165485	0,741573597	0,810141091	0,811911711	0,050126
Q922D8	C1TC	0,940353527	1,130422684	0,954280474	0,974943315	1,091102597	1,206653039	1,086001183	1,046708012	1,193691901	1,124831346	0,050239
Q9QUM9	PSA6	0,882515333	0,956778825	1,085756236	1,074949606	0,93751653	0,934912738	0,801001091	0,848569322	0,700936015	0,844587139	0,051028
Q9JLN9	MTOR	0,829901466	0,96156062	1,183470893	1,025067021	0,86833255	0,540346354	0,768239303	0,786420122	0,90343181	0,773354028	0,051356
P61329	FGF12	1,010665596	0,969673419	1,00926428	1,010396706	0,922655694	0,835301162	1,0156697	0,93314916	0,883029374	0,917961018	0,051657
Q8QZY1	EIF3L	0,853015738	1,09341774	0,996542087	1,057024434	0,798732895	0,399285069	0,789271766	0,974040366	0,661850066	0,724636032	0,051783
P97807	FUMH	1,041302825	0,989822468	0,967049829	1,001824879	0,933270264	0,940765637	1,001673043	0,956366633	0,936232763	0,953661668	0,051798
P12658	CALB1	0,946566572	0,982455077	0,98855365	1,082424701	0,815495603	0,889969863	0,931908595	0,996265289	0,858728813	0,898473632	0,051917
Q61234	SNTA1	0,984973426	0,96705738	0,987870296	1,060098898	0,995131165	0,924210585	0,92925603	0,937424347	0,859946204	0,929193666	0,052238
P28474	ADHX	1,014330182	0,961006073	0,97344504	1,051218704	1,033802	0,905015111	0,849167512	0,816518247	0,810736227	0,883047819	0,052327
Q99KJ8	DCTN2	0,962658958	1,129921897	0,854768726	1,052650419	1,20434199	1,089018998	1,12227886	1,122541725	1,11979551	1,131595417	0,052489
Q8K274	KT3K	1,034759263	0,923857607	0,901593959	1,13978917	0,832695171	0,948746584	0,925162955	0,752597152	0,724930993	0,836826571	0,052497
P63163	RSMN	0,888922337	0,988522351	1,040770208	1,081785104	0,911667422	0,834249747	0,973898005	0,896530253	0,849843283	0,893237742	0,053668
Q62348	TSN	0,856842109	0,928556123	1,056033767	1,158568001	0,847863831	0,799562145	0,927307884	0,882154482	0,757507147	0,842879098	0,0539

Q00493	CBPE	0,902993086	1,042787543	1,001398813	1,052820558	0,889993147	0,816781211	0,95614139	0,931999418	0,932501415	0,905483316	0,054534
Q8BGB7	ENOPH	0,949159057	0,948925301	1,101075237	1,000840405	0,953093705	0,904329267	0,927779566	0,839462581	0,719726066	0,868878237	0,054592
P09411	PGK1	0,994734844	0,939261727	1,017775107	1,048228322	0,916621101	0,846784547	0,952534844	0,988553078	0,908042365	0,922507187	0,054684
Q8BGQ7	SYAC	0,943368301	1,078905274	0,983991353	0,993735071	1,041097843	1,026580647	1,110061327	1,077555836	1,128708716	1,076800874	0,054896
Q9DCP2	S38A3	1,135639827	0,901136366	1,003881409	0,959342398	0,920280656	0,972326019	0,813984895	0,738943466	0,812616983	0,851630404	0,055029
O35678	MGLL	1,070836794	1,345502704	0,205391376	1,378269126	1,630574708	1,496041926	1,857492893	1,521208169	1,425618219	1,586187183	0,055191
P10630	IF4A2	0,934231044	1,002144646	1,07971526	0,983909049	0,826202068	0,90344859	0,972329695	0,96094365	0,860987453	0,904782291	0,055322
Q99J99	THTM	1,008712035	0,975728355	1,011264317	1,004295294	1,045094791	0,837204718	0,824924044	0,911491026	0,804779178	0,884698751	0,055893
P62317	SMD2	0,815149972	0,982162512	1,139729399	1,062958117	0,887531437	0,804524928	0,94424685	0,724348817	0,77282225	0,826694856	0,056095
Q9CQ54	NDUC2	0,8720636	0,984443163	1,145897547	0,997595691	0,787553228	0,852403036	0,932247469	0,826225425	0,928514294	0,86538869	0,056452
Q6X893	CTL1	1,018968243	1,06482115	0,935655669	0,980554938	1,020934259	1,0860458	1,170115967	1,12559283	1,041366702	1,088811112	0,057876
P05202	AATM	1,038357548	1,004890081	0,978798056	0,977954315	1,022043631	0,930800692	0,92372087	0,895329341	0,874387904	0,929256488	0,058286
P05064	ALDOA	0,976878108	1,064102121	0,946647473	1,012372298	1,09644077	1,028047895	1,04658246	1,070970195	1,049227119	1,058253688	0,058879
Q7TQD2	TPPP	1,104149666	1,042746585	0,849875665	1,003228084	1,153726488	1,216131739	1,119435872	1,17652798	1,018812029	1,136926822	0,059186
Q8BG51	MIRO1	0,956307466	1,102079518	0,959427062	0,982185954	0,897028422	0,800622136	0,926837439	0,967795003	0,917268985	0,901910397	0,059895
Q9WTT4	VATG2	0,824066749	0,978432138	1,049401404	1,148099709	1,208753063	1,121815427	1,353437956	1,168133796	1,063213398	1,183070728	0,059996
P61082	UBC12	0,90532134	1,038894992	1,041021019	1,014762649	0,811340977	0,919023919	1,010128621	0,863779598	0,754516905	0,871758004	0,060529
Q922R8	PDIA6	0,957176039	1,069675497	0,999797773	0,973350691	0,913628784	0,920688349	0,960325344	0,969927086	0,954049456	0,943723804	0,060944
Q61035	SYHC	0,930944043	0,992348457	1,039209644	1,037497855	0,908379486	0,891591263	1,007028744	0,902946462	0,791275444	0,90024428	0,061191
P59325	IF5	0,989502231	1,053128966	1,01116704	0,946201763	0,997675595	0,722089005	0,894097735	0,926869587	0,832843092	0,874715003	0,061207
Q3UUI3	THEM4	1,267008318	1,116369225	0,01896691	1,597655546	1,463774713	1,516439651	1,799476672	2,034097007	1,776985002	1,718154609	0,061373
P48453	PP2BB	0,829570001	1,001783659	1,139537147	1,029109193	0,883353539	0,751277565	0,847198852	0,955979225	0,795338571	0,846629551	0,062085
P12970	RL7A	0,903392199	0,996520753	1,080470719	1,019616329	0,946104617	0,951563152	0,938275124	0,836005364	0,828278884	0,900045428	0,062391
P63213	GBG2	0,828546511	1,052993259	1,075207689	1,043252542	1,050666085	1,145088902	1,146187368	1,145668791	1,11712664	1,120947557	0,062635
Q60931	VDAC3	1,056693388	0,946481376	1,016329398	0,980495838	0,88162449	1,012292031	0,93029587	0,785896036	0,750692969	0,872160279	0,06275
Q9CPU4	MGST3	1,053416778	0,959247733	1,121004265	0,866331224	0,965493209	0,805895432	0,86698631	0,890944577	0,834062646	0,872676435	0,063439
Q8R0F8	FAHD1	1,011911351	1,074293824	0,983797229	0,929997596	0,867667872	0,954602919	0,969202294	0,907916592	0,933353894	0,926548714	0,063442
Q9QY76	VAPB	1,003334551	1,063860569	0,972136992	0,960667888	1,148840751	1,134360148	1,123003501	1,056912939	0,982871794	1,089197827	0,063443
Q9DBF1	AL7A1	1,003184653	1,03412285	0,98529993	0,977392567	1,048176038	1,200042269	1,080908648	1,039391556	1,034556434	1,080614989	0,064479
P06745	G6PI	1,043407064	0,982544298	0,932948588	1,04110005	1,009446663	1,053472364	1,099186437	1,151914581	1,074074069	1,077618823	0,065009
P24527	LKHA4	0,984865017	1,043938333	1,039747321	0,931449329	1,00974211	1,070763424	1,062282288	1,072631066	1,088893327	1,060862443	0,065219
Q99N28	CADM3	0,94996547	1,009707469	1,058887116	0,981439945	1,107235898	1,095622638	1,07994339	0,979713532	1,114535819	1,075410255	0,065225
Q91XV3	BASP1	0,928931192	1,126078169	0,909546846	1,035443793	1,017986867	1,339663959	1,035900459	1,402129144	1,319890523	1,223114191	0,06536
Q9DB05	SNAA	1,034735466	0,996754644	0,957174476	1,011335414	0,957568988	0,95449011	0,959899011	0,851733642	0,760567419	0,896851834	0,06548
Q8K3J1	NDUS8	1,053410423	1,003169336	0,940885587	1,002534654	0,836150192	0,798882366	1,004284009	0,965042149	0,866123511	0,894096445	0,065737
Q9CQ69	QCR8	0,924420104	1,064754058	1,077818778	0,933007061	0,770967021	0,940112909	0,980015456	0,846220871	0,863146852	0,880092622	0,066354
Q8BW75	AOFB	1,073695674	1,092979027	1,007330794	0,825994506	1,085977135	1,072204081	1,189989616	1,1170873	1,193708778	1,131793382	0,066832
Q9JJK2	LANC2	0,990389559	0,973042683	1,061756627	0,974811131	1,011770766	0,878249759	0,941179761	0,863601053	0,926717226	0,924303713	0,067101
P14131	RS16	0,895604944	1,048647726	1,028644903	1,027102427	0,851603695	0,771163353	1,033532305	0,880313903	0,829421932	0,873207038	0,067147
Q9QUI0	RHOA	0,860839396	0,964862221	1,089084053	1,085214331	0,978040992	1,165467363	1,366909664	1,233928126	1,183684127	1,185606054	0,067268

Q78IK2	USMG5	0,878720722	0,968010502	1,191915349	0,961353427	0,761932388	0,884765967	0,949732358	0,846140351	0,811000181	0,850714249	0,067894
Q9R0X4	ACOT9	0,996758076	0,981125647	1,031060015	0,991056261	0,966417526	0,930066925	0,961361568	1,005102479	0,904916091	0,953572918	0,067996
Q9CR67	TMM33	0,911083304	1,094958201	1,026019828	0,967938667	0,963597734	0,946148846	0,913541548	0,865173852	0,826346977	0,902961791	0,068242
Q9DCZ1	GMPR1	1,057931035	1,107294261	0,931130036	0,903644667	0,900836921	0,85428372	0,900130432	0,966920522	0,776591492	0,879752617	0,068582
P62137	PP1A	0,949694942	0,947676301	1,085304102	1,017324654	1,027057577	0,872150806	0,835681271	0,862172165	0,890133914	0,897439147	0,068612
Q3TDK6	ROGDI	1,073105848	0,914804168	0,967752206	1,044337778	0,915820164	1,004602921	0,863769618	0,884423933	0,797987955	0,893320918	0,069302
Q99PT1	GDIR1	1,020926616	0,974457424	0,983583007	1,021032953	1,054579239	1,109791868	1,138363502	1,062003715	0,981286778	1,069205021	0,069363
Q00PI9	HNRL2	1,013396992	1,008983103	0,933349492	1,044270412	1,178256047	1,084656649	1,046735155	0,998062364	1,162903624	1,094122768	0,069424
Q62418	DBNL	0,981658314	0,969243618	1,028622473	1,020475595	0,955083924	0,822256873	0,86180972	0,961375293	0,988351747	0,917775511	0,070047
Q91WC3	ACSL6	1,243934649	0,940428956	0,952962875	0,862673521	1,176134792	1,19614295	1,134568547	1,210726505	1,100638226	1,163642204	0,071339
Q8BWY3	ERF1	0,871506859	0,872434016	1,162189424	1,093869701	1,115300229	1,053665791	1,279604194	1,157900596	1,261343242	1,17356281	0,072371
Q8BK08	TMM11	1,06414432	0,96512132	0,987218031	0,983516329	0,853616969	0,955075165	0,937599791	0,760917949	0,975336809	0,896509336	0,07249
Q80ZJ1	RAP2A	0,962236674	0,954320325	1,079172912	1,004270089	1,07162944	0,59288594	0,870995878	0,754608128	0,586631978	0,775350273	0,072618
P51174	ACADL	1,032838785	1,052442226	0,902020256	1,012698733	1,020594565	1,049753695	1,152326279	1,108421744	1,083748443	1,082968945	0,072744
P56959	FUS	0,901776859	1,015110105	1,079668086	1,003444951	0,896620554	0,79485808	0,894757631	0,864481798	1,010955691	0,892334751	0,073085
Q02357	ANK1	1,045427063	1,050526547	0,886244824	1,017801566	0,928989866	0,996222804	0,842750073	0,758292038	0,87844687	0,88094033	0,073823
P48722	HS74L	0,909788656	1,090201538	0,978120184	1,021889622	1,139104152	1,09693195	0,998811302	1,094808827	1,125309124	1,090993071	0,074132
Q9DCT2	NDUS3	1,09207309	0,966968523	0,962683357	0,97827503	0,945172045	0,947079911	0,974383421	0,885606934	0,828481326	0,916144728	0,075897
Q9DCT8	CRIP2	1,047744858	0,975398009	0,94819325	1,028663883	1,129769545	1,217277356	0,996678352	1,061365907	1,345643548	1,150146942	0,075953
Q99M71	EPDR1	0,996298786	0,900238161	1,000519053	1,102944001	0,897725665	1,015797373	0,869149441	0,793980686	0,709783543	0,857287342	0,076087
Q8K021	SCAM1	0,990764109	1,103431134	0,900609482	1,005195275	0,986699079	0,879434298	0,920854228	0,89403578	0,806271618	0,897459001	0,076228
P70296	PEBP1	0,70226278	0,851323003	1,457587036	0,988827181	0,754354106	0,57405638	0,784724069	0,643819678	0,686759409	0,688742728	0,076293
Q3UHB8	CC177	0,982072991	1,030292503	1,034655559	0,952978946	1,023118195	0,895203142	0,829547071	0,771492873	0,938657888	0,891603834	0,076654
Q9EPR4	S23A2	0,90397393	1,048754468	0,88297771	1,164293891	0,883816021	0,838131089	0,804585871	0,660567292	0,967726504	0,830965355	0,07703
Q60932	VDAC1	1,016638017	0,974125694	0,994250644	1,014985645	0,925120344	1,038030043	0,919247687	0,832625181	0,830026386	0,909009928	0,078125
Q61081	CDC37	0,900630697	1,029514177	1,028302065	1,041553061	1,054226829	0,998192523	1,185739977	1,142842728	1,107699542	1,09774032	0,078144
P60766	CDC42	0,976001005	1,06742297	0,941554223	1,015021802	0,901398759	0,9696996	0,954166066	0,961174736	0,844522074	0,926192247	0,078236
Q71LX4	TLN2	0,917431624	1,054859119	0,981357903	1,046351355	1,437436459	1,506844003	1,174584353	1,025882055	1,047881219	1,238525618	0,078924
Q8BG73	SH3L2	0,884716078	0,913116551	1,132458126	1,069709245	0,922981582	0,85123002	0,945200431	0,861280886	0,783168379	0,87277226	0,078967
Q9CPQ1	COX6C	0,919454142	0,993962133	1,091055405	0,99552832	0,89790693	0,870891685	0,980950167	0,960877889	0,811523444	0,904430023	0,07906
Q9JJU8	SH3L1	0,724615894	1,039024899	1,115204613	1,121154595	0,681300142	0,815896262	0,969243294	0,678532145	0,805168096	0,790027988	0,079078
Q6ZWN5	RS9	0,901739412	0,988941225	1,049005906	1,060313457	0,875119937	0,9049616	0,987840341	0,944307403	0,820958222	0,906637501	0,079167
Q8BFR5	EFTU	1,014519213	1,024381665	0,999221357	0,961877765	0,998738599	0,958609175	0,97295715	0,953724084	0,960339305	0,968873662	0,079171
Q99M87	DNJA3	0,954763588	1,010448643	0,998805548	1,03598222	0,983785819	0,844463084	0,871297989	0,9042893	1,003561786	0,921479596	0,079504
Q99NB1	ACS2L	0,952409443	1,042712468	0,960839881	1,044038207	0,914875377	0,642860928	0,095037401	0,810772377	0,817693207	0,656247858	0,080119
P48320	DCE2	0,936166653	1,212964807	0,82991722	1,02095132	0,996390629	1,150665477	1,390606357	1,452872861	1,210344935	1,240176052	0,080364
P61957	SUMO2	0,885507323	0,892779777	1,161586351	1,060126549	0,797645023	0,93553806	0,938881881	0,833835757	0,75607333	0,85239481	0,08057
P54071	IDHP	0,931417725	1,087163225	0,964667767	1,016751283	0,879537043	0,724176536	0,997514695	0,817477037	0,948183135	0,873377689	0,081381
Q9JIG7	CCD22	0,777956126	1,115742681	1,222239187	0,884062006	0,043540071	0,450404332	0,830455749	0,712287131	0,923446778	0,592026812	0,081774
P68040	RACK1	0,95684045	0,995661865	1,011631448	1,035866237	0,973643862	0,966948564	0,941620311	0,810632238	0,732833372	0,885135669	0,081823

Q8K2B3	SDHA	1,031222258	1,006734381	0,954325758	1,007717603	1,022838491	1,077111794	1,09763401	1,021333048	1,019711768	1,047725823	0,08224
P48774	GSTM5	0,937767532	0,934416928	1,01138859	1,11642695	1,087837947	1,031455537	1,370116751	1,274201342	1,077337182	1,168189752	0,082485
P17183	ENOG	0,828235771	0,860931688	1,188329877	1,122502664	0,788024696	0,666152258	0,80572597	0,932826057	0,855206826	0,809587161	0,08261
Q9D2G2	ODO2	1,073397154	0,975005825	0,96423134	0,987365681	0,932610521	0,946158958	0,96585724	0,979721005	0,882309071	0,941331359	0,08301
P60521	GBRL2	0,88302665	1,039746195	1,118580815	0,95864634	0,894507633	0,730864395	0,985042224	0,894192231	0,838149366	0,86855117	0,083029
P51859	HDGF	0,998705556	0,979484015	1,005205318	1,016605111	0,844990646	0,587529039	0,941761003	0,969010467	0,879712354	0,844600702	0,084341
Q8BP67	RL24	0,710930224	1,051808458	1,102416944	1,134844375	1,077662232	1,071695672	1,341038948	1,286853682	1,319775955	1,219405298	0,084971
P23116	EIF3A	0,901648519	1,208893462	0,945819079	0,943638941	1,344853043	1,277830778	1,095446888	1,052056023	1,120173245	1,178071996	0,085551
Q9CYR6	AGM1	0,823724466	1,109168455	0,957712536	1,109394542	1,066711091	1,02755249	1,238162994	1,272714473	1,216009899	1,164230189	0,085576
Q8QZR5	ALAT1	1,031869415	0,949139757	1,028529392	0,990461436	0,954329097	0,564743957	0,916605661	0,917792191	0,82401508	0,835497197	0,085836
Q91V12	BACH	1,001176373	1,027798302	0,9639972	1,007028125	1,03564422	0,892105944	0,925744541	0,934625746	0,886698004	0,934963691	0,086071
Q6ZWV3	RL10	0,841433595	0,933308465	1,073013101	1,15224484	1,083426283	1,050093444	1,391452384	1,230454507	1,162648956	1,183615115	0,086278
Q91ZA3	PCCA	0,950302424	1,145563394	0,958895882	0,9452383	0,904506289	0,927521267	0,868487861	0,775751324	0,964478571	0,888149062	0,086549
P25444	RS2	0,978816786	0,977776898	0,99225921	1,051147105	0,922029284	0,984983064	0,980068626	0,817712619	0,749284252	0,890815569	0,086821
Q9CPR4	RL17	0,944341811	1,06780408	0,994970069	0,992884041	0,908313111	0,95566219	1,01493888	0,848640521	0,839266496	0,91336424	0,087322
P17879	HS71B	0,747341615	1,160678316	1,105743196	0,986236873	0,81006107	0,747839548	0,893143659	0,815681193	0,880435143	0,829432122	0,087589
P60487	PLPP	0,956972274	0,9150228	1,080400072	1,047604854	0,965668089	0,902663663	0,904480621	0,934386319	0,93275414	0,927990566	0,087591
Q8BG05	ROA3	0,935592131	0,976132097	1,032318082	1,05595769	0,985154006	0,821195837	0,982004617	0,908240086	0,880598077	0,915438525	0,087999
Q8BHF7	PGPS1	1,025534566	1,051580796	0,878311745	1,044572894	1,019841962	1,034892635	1,213664191	1,406797594	1,185303673	1,172100011	0,08991
Q91ZP9	NECA2	0,98053966	1,0158579	0,966568582	1,037033857	0,977957597	0,634724376	0,873549306	0,861911393	0,958976094	0,861423753	0,090814
P63323	RS12	0,876435221	1,028287603	1,093519721	1,001757455	0,868600983	0,929372997	0,998475289	0,859681876	0,780740634	0,887374356	0,090953
Q9ESM3	HPLN2	1,247470724	1,172642837	0,788657357	0,791229081	0,86598692	0,860985853	0,776314989	0,685193073	0,653343716	0,76836491	0,091035
Q61029	LAP2B	0,97113822	1,078878301	0,947904388	1,00207909	0,927076977	0,809012805	0,883629976	1,016135502	0,918328446	0,910836741	0,091216
Q9CRB8	MTFP1	0,97009904	1,17996972	0,839907798	1,010023442	0,907161716	0,91820837	0,825482758	0,885431554	0,652893185	0,837835517	0,091333
Q60597	ODO1	1,093523787	0,953692192	0,956890494	0,995893526	0,982713928	0,926295168	0,879956108	0,892030522	0,96356321	0,928911787	0,091861
P60710	ACTB	0,768519243	1,08121545	1,048201998	1,10206331	1,155064044	1,102925015	1,162751918	1,110741442	1,149192495	1,136134983	0,092324
Q9DBG3	AP2B1	1,035962721	0,949576732	0,995021629	1,019438919	1,128274595	0,954531299	1,061469397	1,185364726	1,232826233	1,11249325	0,092332
Q922B2	SYDC	0,956683343	1,035125797	0,997299971	1,010890888	0,968964568	0,921058924	0,978284998	0,992810754	0,931591481	0,958542145	0,092336
Q9Z2H5	E41L1	0,919899042	1,01771663	1,036277401	1,026106927	1,024858136	1,06153605	1,081624548	1,045437996	1,204444874	1,083580321	0,092868
P56391	CX6B1	0,825345531	0,946129914	1,234339239	0,994185316	0,623012061	0,94067673	0,797965119	0,76340444	0,911134837	0,807238637	0,092949
Q9JHK4	PGTA	1,025570335	1,049778384	1,006147888	0,918503394	0,902741264	0,828397172	0,209635216	0,89683666	0,716109915	0,710744045	0,093716
Q9QZ88	VPS29	0,870216616	1,002104397	1,10080501	1,026873977	0,867448273	0,783119948	1,017964562	0,895588245	0,845822814	0,881988768	0,093859
P21279	GNAQ	0,927010862	1,04545772	1,026150135	1,001381283	0,967212384	0,942220012	0,941112832	0,961154609	0,959951871	0,954330342	0,093914
Q9CYH2	F213A	1,005481185	1,035910331	0,974177602	0,984430882	0,859877197	1,045072777	0,925407749	0,915476425	0,886037136	0,926374257	0,094024
Q8CGF6	WDR47	0,996817594	1,029352283	1,042628621	0,931201501	0,943880003	0,915086213	1,009316652	0,899625553	0,936603041	0,940902292	0,094137
Q9WUK2	IF4H	1,12103684	1,198109266	0,858832812	0,822021083	1,08332502	1,32053817	1,135413831	1,191517869	1,67501438	1,281161854	0,095111
Q8BGY7	F210A	1,001235263	1,125356084	0,933150779	0,940257873	0,873429648	0,821831744	0,843421406	0,828304475	0,22108288	0,717614031	0,095127
Q9ER58	TICN2	1,045126403	1,024669106	0,871080448	1,059124043	0,946442109	0,653103985	0,946214555	0,866489542	0,891520898	0,860754218	0,095809
Q9JKB1	UCHL3	1,020693545	0,945252339	0,992059485	1,041994631	1,120717895	0,971249823	1,089215108	1,119447728	1,050301052	1,070186321	0,096273
Q68FD5	CLH1	0,965662534	1,009186619	1,029734928	0,995415919	1,155972253	1,016931678	0,991729576	1,066887266	1,133387124	1,072981579	0,09639

A2RT62	FXL16	1,010099428	0,908300349	1,102272615	0,979327609	0,799748064	0,758465604	0,876319967	0,924033233	1,026956102	0,877104594	0,097149
Q64332	SYN2	0,883416983	0,999172143	1,087453946	1,029956928	0,891843311	0,799744401	0,939290164	0,814886624	1,004814592	0,890115819	0,097905
P51863	VA0D1	0,911478445	1,01343516	1,027129772	1,047956623	1,013963818	0,896155115	0,909358732	0,939853518	0,881570105	0,928180258	0,098418
Q69ZS6	SV2C	1,012055269	1,208079391	0,828558873	0,951306468	1,195153454	1,216313143	1,175013598	1,133228386	1,030962313	1,150134179	0,098624
P61222	ABCE1	0,829106242	1,06394948	1,068649424	1,038294853	0,837602368	0,893551788	0,862783752	0,977631251	0,885246016	0,891363035	0,098688
P31324	KAP3	1,041295259	0,914665255	1,109993042	0,934046445	1,258703133	1,022056408	1,055518893	1,112696421	1,39607564	1,169010099	0,098757
Q8VED9	LEGL	0,6314374	0,874463792	1,413008932	1,081089876	0,804332317	0,590093819	0,826846471	0,490875832	0,741816595	0,690793007	0,099502
P29341	PABP1	0,896442446	1,081376807	1,016514865	1,005665882	1,061773958	1,101793969	1,050114927	1,062824172	1,057498199	1,066801045	0,09959
Q9WUR9	KAD4	0,943980861	0,879898826	1,060385	1,115735314	0,849425253	0,972583217	0,934945736	0,772902263	0,889665693	0,883904432	0,10049
Q8BK64	AHSA1	0,972026166	1,112921699	0,924181353	0,990870782	0,934066956	0,952463557	0,938582285	0,872537886	0,938844995	0,927299136	0,102421
Q61543	GSLG1	1,032741492	0,792367864	1,195113572	0,979777072	0,830536834	0,57889225	0,771827134	0,759885409	1,028910634	0,794010452	0,102435
Q9DCX2	ATP5H	1,038349262	0,971474289	0,975530164	1,014646285	0,934348249	0,994531637	0,992101768	0,915380062	0,95039783	0,957351909	0,102546
Q501J6	DDX17	0,952595459	0,996039252	1,055390766	0,995974523	0,866952864	0,832454568	0,987834685	0,829319044	1,020593894	0,907431011	0,103158
Q61768	KINH	1,100489678	1,030761843	1,039652194	0,829096285	1,026202418	1,137807944	1,052983783	1,188525275	1,245346992	1,130173282	0,10357
Q924M7	MPI	0,890390979	1,090786979	1,016470652	1,00235139	0,989349264	0,882123289	0,895526597	0,954365915	0,852356836	0,91474438	0,106585
P70663	SPRL1	1,062270566	1,01676613	0,95434488	0,966618424	0,997651077	0,925257364	0,96799413	0,930984978	0,852936976	0,934964905	0,106713
P70441	NHRF1	0,966727173	0,988877556	1,057141717	0,987253554	1,004299955	0,854252755	0,954257937	0,87875543	0,971017142	0,932516644	0,107351
O88844	IDHC	0,917969877	0,935251641	1,052325514	1,094452968	0,869388413	0,749530285	0,979925324	0,90926837	0,946608069	0,890944092	0,107677
Q6ZPJ3	UBE2O	0,918977236	1,031492312	1,008502333	1,041028119	1,122463004	0,935306713	1,100580515	1,24662183	1,187396976	1,118473808	0,108074
P09528	FRIH	1,012214776	1,081380233	0,943949604	0,962455387	0,844255719	0,896115015	1,068380354	0,846782255	0,791569033	0,889420475	0,110022
O35098	DPYL4	0,855258222	1,117277927	1,017648318	1,009815533	1,119100507	1,009246438	1,165809722	1,182904594	1,068408116	1,109093875	0,110531
O35345	IMA7	1,143792522	0,868155744	0,94554908	1,042502654	1,01996647	1,339907256	1,126907538	1,19263955	1,066446389	1,14917344	0,112466
Q99LX0	PARK7	0,948501219	0,995740883	0,94066647	1,115091428	1,04710448	1,044510107	1,223747247	1,157622378	1,032984314	1,101193705	0,112626
O08709	PRDX6	0,884386341	0,996252536	1,004378366	1,114982757	1,076555358	1,147088246	1,131620228	1,055572792	1,028907055	1,087948736	0,11306
P07901	HS90A	0,964934628	1,091109679	0,949875697	0,994079997	1,057261267	1,065714505	1,058689904	1,009696594	1,102268979	1,05872625	0,113705
Q8BWR2	PITH1	0,902290338	1,019580031	0,88822749	1,189902141	0,799308181	0,894687638	1,020213373	0,801030266	0,733536294	0,84975515	0,114339
Q6ZWV7	RL35	0,687457545	1,068315144	1,121395467	1,122831845	1,055428903	1,243218617	1,411603327	1,126012416	1,19849483	1,206951619	0,114389
Q80X50	UBP2L	0,983623177	1,052750496	0,938767985	1,024858342	1,094190571	0,781503021	0,843791539	0,814304917	0,86671198	0,880100406	0,114403
P49025	CTRO	0,910128638	1,041516762	0,968384671	1,079969928	1,21070683	1,080099504	0,982368188	1,103562901	1,098973615	1,095142208	0,115304
Q61885	MOG	1,065986194	0,972815133	0,923421615	1,037777058	0,978057154	1,36320277	1,34458733	1,199457511	0,993463258	1,175753605	0,115496
Q9QYJ0	DNJA2	0,990054257	1,026934157	0,993341122	0,989670464	0,909288634	0,927382515	0,976740548	0,928898193	1,027542943	0,953970567	0,115876
P11404	FABPH	0,613360004	0,766962231	1,596967222	1,022710543	0,69078065	0,495643298	0,73032017	0,644066073	0,690062103	0,650174459	0,116612
Q91ZJ5	UGPA	0,933557068	1,0305525	1,04609907	0,989791362	0,984594966	0,870440836	0,974406558	0,968076906	0,89450855	0,938405563	0,116699
P40124	CAP1	0,944251468	1,027240582	1,054886734	0,973621216	1,05192757	0,984687512	1,04954386	1,169831923	1,213104295	1,093819032	0,118209
Q9CQQ7	AT5F1	0,994255287	1,017780218	0,943133423	1,044831072	0,88111083	0,96561207	1,004350064	0,96527706	0,884483356	0,940166676	0,118313
Q9D0F9	PGM1	0,787581927	0,933634049	1,136223289	1,142560736	0,757774536	0,763898731	0,945330031	0,893752786	0,871816465	0,84651451	0,118406
Q9CQC7	NDUB4	1,034919153	1,050135321	1,023865154	0,891080373	0,784549745	0,997581105	0,990607313	0,835143952	0,88012926	0,897602275	0,119252
P28663	SNAB	0,891645957	0,934817116	1,09300836	1,080528567	0,838090392	0,859860415	0,99000172	0,944287551	0,882196749	0,902887365	0,120299
Q8C8R3	ANK2	0,90970898	1,037307437	1,028337713	1,024645871	1,066229247	1,042053902	1,035195906	1,030636053	1,155800237	1,065983069	0,121287
Q3UM45	PP1R7	0,99679176	0,976305793	1,037612742	0,989289705	1,04433559	0,9381038	1,134722933	1,175281189	1,169968685	1,092482439	0,1216

P68037	UB2L3	0,946978158	1,084723045	0,96047337	1,007825428	0,970579689	0,899400733	0,95547129	0,978662455	0,83703428	0,928229689	0,121636
Q64133	AOFA	1,040056925	1,042339345	0,988020932	0,929582799	0,840059349	0,93699432	1,013431972	0,963482907	0,874408136	0,925675337	0,121662
Q99KK7	DPP3	1,065294008	0,956680732	1,054798339	0,92322692	0,947157278	0,917673494	0,507161011	0,900981025	0,87345414	0,82928539	0,123132
P62301	RS13	0,856639906	1,038467311	1,036925944	1,067966839	0,874552801	1,006138394	0,876261832	0,884903831	0,912297729	0,910830917	0,123191
Q03265	ATPA	1,056505057	1,014040265	0,966177978	0,9632767	0,950219423	0,967439625	0,99007272	0,95366838	0,952945449	0,96286912	0,123224
Q61699	HS105	0,913812158	1,06497647	1,042173164	0,979038208	1,063696256	1,147139812	1,04740684	0,993737781	1,21328873	1,093053884	0,123869
Q99JI4	PSMD6	0,865669804	1,044978417	1,086190866	1,003160913	0,965076203	0,906183458	0,903348852	0,858531207	0,95357802	0,917343548	0,124386
Q9CWJ9	PUR9	0,96355898	0,92386625	1,034014773	1,078559997	0,634189451	0,845808475	0,915530763	1,001068559	0,929584132	0,865236276	0,125447
Q9CR26	VTA1	1,032391905	1,033243333	0,976877513	0,957487249	1,193315367	0,953423733	1,110925907	1,067312443	1,084190357	1,081833561	0,125728
O70589	CSKP	1,010031272	0,938475796	1,019884777	1,031608155	1,152971603	1,082002484	1,022266972	1,002078105	1,04694047	1,061251927	0,126012
P00920	CAH2	1,117900553	0,930386725	0,899080104	1,052632618	0,952856566	0,766689888	0,994387708	0,910404963	0,723064735	0,869480772	0,126149
Q3TC72	FAHD2	0,941545905	0,981188642	1,099254852	0,978010602	0,984236917	0,966138847	0,879143848	0,944199814	0,901943936	0,935132672	0,126715
Q9CXY6	ILF2	0,881971021	1,051711059	1,054121241	1,01219668	0,995639213	0,860338087	0,912582835	0,917983935	0,938870682	0,92508295	0,127681
P40237	CD82	1,124608715	1,024632841	0,886712572	0,964045872	1,017624877	1,17202403	1,139312332	1,146824718	1,022273918	1,099611975	0,128617
Q9CY58	PAIRB	1,012632742	1,058282234	0,790801014	1,13828401	1,429568346	1,154031711	1,120602851	1,077185177	1,0729091	1,170859437	0,130224
Q80TL4	PHF24	0,993299552	0,910749176	1,16452135	0,931429922	0,875252955	0,763056241	0,927560683	0,890800182	0,983981267	0,888130266	0,130427
P62984	RL40	0,921523077	1,109046231	1,019759764	0,949670928	1,005490253	0,907633781	0,824267172	0,88752332	0,945807003	0,914144306	0,130742
Q9D358	PPAC	0,860888019	1,067248631	1,134627687	0,937235664	0,033215792	0,765317311	0,937395157	0,843075744	0,779929558	0,671786712	0,131287
Q641P0	ARP3B	0,836805343	1,061219993	1,129946885	0,972027779	1,134408782	0,930067466	1,234909948	1,174794666	1,391004395	1,173037051	0,131657
Q61301	CTNA2	1,197772852	0,881287033	0,897291819	1,023648296	1,254617189	1,363523017	1,20675467	1,026942768	1,005730879	1,171513704	0,132283
Q9JLB0	MPP6	1,068028696	1,028311586	0,891395786	1,012263932	1,119556096	1,087653066	1,045315855	1,094987706	1,001044592	1,069711463	0,132758
Q8VE62	PAIP1	0,89088213	0,996134036	1,060793392	1,052190442	0,825301522	0,838666976	0,881028104	0,778996057	1,085264494	0,88185143	0,133422
Q6PER3	MARE3	1,013084487	0,991583286	0,932790154	1,062542073	0,995683668	1,01868979	0,881568424	0,852251636	0,846254445	0,918889592	0,133977
Q8VIJ6	SFPQ	0,869262817	1,043460122	1,129320457	0,957956604	1,121388694	1,005153184	1,100269252	1,092319182	1,169349324	1,097695927	0,134187
P28867	KPCD	1,45638426	1,046474976	0,783581448	0,713559316	1,677238626	1,241782521	1,154162259	1,20428112	1,264697226	1,308432351	0,134309
Q9D898	ARP5L	0,939530792	0,949134173	1,093180037	1,018154999	1,010902692	0,902492458	0,935053399	0,899484208	0,923603732	0,934307298	0,134583
P46638	RB11B	0,965458641	0,991156196	0,99447144	1,048913722	0,901152064	0,961857878	1,034345404	0,914961969	0,806674377	0,923798338	0,13512
P70349	HINT1	0,502983001	0,77057383	1,893010099	0,83343307	0,494317832	0,47676328	0,653254333	0,523127513	0,56915527	0,543323646	0,136184
P24472	GSTA4	0,893641569	0,972179376	1,039376587	1,094802468	0,77928426	1,023506909	0,999800892	0,812890226	0,722183025	0,867533062	0,136217
P62242	RS8	0,874157622	1,007984653	1,050039164	1,067818562	0,87573509	0,944018632	1,014899892	0,857718036	0,882951645	0,915064659	0,136493
Q9D819	IPYR	0,953955771	0,947997854	1,014756647	1,083289727	1,00576043	0,920524348	0,973723173	0,899488455	0,836480639	0,927195409	0,137671
P57780	ACTN4	0,963299439	0,987592994	1,150626049	0,898481518	0,938955647	0,876022019	0,948917503	0,821260729	0,95180859	0,907392898	0,137991
P45591	COF2	0,973339805	0,961096069	0,997975482	1,067588643	0,943369287	0,75726813	0,90590738	1,055540295	0,819082091	0,896233437	0,137997
P55066	NCAN	0,818800234	0,792193311	1,167196803	1,221809653	0,782967045	0,705341998	0,69935229	0,54700056	1,084834593	0,763899297	0,138985
P63044	VAMP2	0,918426271	1,06400711	1,030051949	0,98751467	1,109639217	1,004418201	1,041124728	1,269396425	1,067993506	1,098514415	0,139102
Q99MR8	MCCA	0,945142865	1,138673011	0,977305729	0,938878396	0,921032007	0,979390597	0,943598226	0,856193653	0,905328329	0,921108563	0,139531
P62748	HPCL1	0,740004319	1,039993621	1,090466069	1,129535991	0,804663903	0,892147704	0,862434339	0,852677649	0,916431762	0,865671071	0,140006
P50247	SAHH	0,973877598	0,999223735	1,003344262	1,023554405	0,982140244	0,86502542	0,999186094	0,961803952	0,960683932	0,953767928	0,141416
P17182	ENOA	0,942458204	0,939923409	1,051503573	1,066114813	0,907397449	0,879303278	0,908421954	0,98624786	0,991792484	0,934632605	0,14259
P62774	MTPN	0,77478552	0,993223102	1,223956228	1,008035149	0,753824981	0,835845451	0,839513586	0,85932933	0,971020284	0,851906726	0,142864

Q9DBJ1	PGAM1	0,923365377	0,815057183	1,092420487	1,169156953	0,809539956	0,881592489	0,906154679	0,932303174	0,85787658	0,877493376	0,143579
Q9D6J6	NDUV2	1,080535015	1,04749721	0,896672293	0,975295482	0,876343324	0,959308533	0,974798416	0,941009468	0,910516752	0,932395299	0,143791
O70251	EF1B	0,924649959	0,998280527	1,034425678	1,042643835	0,986692988	0,927969514	1,014356822	0,848605571	0,818634565	0,919251892	0,143906
P08752	GNAI2	1,008012635	1,031977018	1,009215385	0,950794963	0,951051132	1,008433724	0,986149474	0,935595233	0,93095188	0,962436289	0,143986
Q9JKC6	CEND	1,121924762	1,171354129	0,732510997	0,974210112	1,121034245	1,37446381	1,203755346	1,187463181	1,0118217	1,179707656	0,144883
P17665	COX7C	0,994420391	1,049744373	1,044204087	0,91163115	0,904905669	0,974441067	0,9717225	0,972138389	0,883592862	0,941360097	0,145103
Q9JM76	ARPC3	0,571261889	1,078244285	1,064896123	1,285597703	1,073781434	1,135337052	1,246365925	1,486995838	1,346910615	1,257878173	0,145264
P49722	PSA2	0,743732128	1,082265838	1,134401311	1,039600724	1,094471707	1,142342933	1,26729881	1,259495876	1,012611255	1,155244116	0,145398
Q9CQ60	6PGL	0,83252767	1,004166292	1,072278272	1,091027766	0,848355123	0,824707108	1,003904268	0,79061326	0,962084962	0,885932944	0,145664
Q6GQT9	NOMO1	0,960976818	1,017667818	0,964390223	1,05696514	0,918463441	0,780557496	0,848387992	0,114390081	0,917655113	0,715890824	0,146774
Q9ERD7	TBB3	1,029173599	1,017035404	0,968418714	0,985372283	1,044343289	0,937186282	1,094195409	1,190517581	1,249673532	1,103183218	0,147687
O88531	PPT1	1,01867362	0,935843455	1,096662537	0,948820389	1,076085062	1,097856895	1,060090061	0,990610615	1,093800049	1,063688537	0,149021
O89112	LANC1	1,036310768	1,020526746	1,002341365	0,940821121	0,958845788	0,871209954	1,016802441	0,76335156	0,957687054	0,91357936	0,149234
Q91Z31	PTBP2	1,050020793	0,939073035	0,763873877	1,247032294	0,982382949	0,727959457	0,703329697	0,912373475	0,802599779	0,825729071	0,149365
Q8R127	SCPDL	1,093722446	1,36325242	0,689263068	0,853762066	1,273739127	1,29080443	1,183277584	1,303496548	1,068839796	1,224031497	0,149368
Q9D0M3	CY1	1,025410631	1,01943848	0,980776387	0,974374502	0,977790247	0,98002555	1,006511513	0,943446961	0,956228435	0,972800541	0,149841
Q61548	AP180	0,91961476	0,984288021	1,050469456	1,045627763	1,114543773	0,981031172	1,117723015	1,013304547	1,129383832	1,071197268	0,15044
P12367	KAP2	0,954964134	1,046183412	1,059649017	0,939203436	1,098937729	0,97134795	1,14896657	1,092509795	1,040202222	1,070392853	0,150711
P35438	NMDZ1	0,947088013	0,952581376	1,11091786	0,989412751	1,078808875	0,802321693	0,730824764	0,807510623	0,949965818	0,873886355	0,15105
Q9R1V7	ADA23	1,014101601	1,080382774	0,964104948	0,941410677	1,103163114	1,046988549	1,032884749	1,062701297	1,012632574	1,051674057	0,151752
Q9D1D4	TMEDA	0,961973346	1,032387951	0,981826384	1,02381232	0,864721205	0,99812087	1,024049456	0,790026097	0,91405869	0,918195264	0,152001
P17156	HSP72	0,770234577	1,047983324	1,146853869	1,03492823	0,841169455	0,872875405	0,892353185	0,837648763	0,95743117	0,880295596	0,154264
Q64674	SPEE	1,153590047	1,00965357	0,848775237	0,987981147	1,230308694	1,03748116	1,060276254	1,335939249	1,038188788	1,140438829	0,154561
O35381	AN32A	1,010292208	0,920533207	1,087540814	0,981633771	1,061470351	1,097764393	1,075919071	1,102511824	0,978015941	1,063136316	0,156263
Q9DAS9	GBG12	1,167158221	1,284246741	1,260074131	0,288520907	1,296134931	1,01427079	1,535338598	1,702730163	1,412962641	1,392287424	0,156818
O08553	DPYL2	0,969085875	1,066309626	0,972477759	0,99212674	1,00505214	1,026565298	1,016803632	1,088164122	1,093563596	1,046029757	0,156985
Q99JY0	ECHB	1,013040363	1,00951009	0,962499787	1,01494976	0,855785837	1,03190178	1,011748075	0,88652893	0,85923331	0,929039586	0,158011
P62754	RS6	0,913049115	0,90980372	1,063720385	1,11342678	0,890621081	1,022601204	0,948658665	0,825818119	0,801077381	0,89775529	0,158755
Q91WS0	CISD1	0,903865536	1,018236706	1,056053435	1,021844322	0,876282253	1,124557812	1,330739478	1,216621153	1,159560509	1,141552241	0,159191
Q9ESN6	TRIM2	0,90667111	1,089534394	0,988491735	1,015302762	1,139097043	1,042466871	1,000240735	1,08868302	1,062672709	1,066632076	0,159237
Q9CPW0	CNTP2	0,981068319	1,11819655	0,972571553	0,928163578	0,939336858	0,945165987	0,918027063	0,917195492	0,97910339	0,939765758	0,159645
Q99LB6	MAT2B	1,070247082	0,970815733	1,009337838	0,949599346	1,04798407	0,90953299	0,940572032	0,90840071	0,842227269	0,929743414	0,159737
Q02248	CTNB1	0,977486594	1,030535131	0,996038814	0,995939461	1,090316295	1,074946189	0,982680617	1,005851971	1,041785918	1,039116198	0,160766
Q9D6M3	GHC1	0,995143158	0,860379649	1,089766857	1,054710336	0,937472738	0,914217145	0,999723809	0,853117686	0,883944356	0,917695147	0,161504
Q9WUM5	SUCA	1,016910538	0,978127642	0,971682441	1,033279379	1,034874783	1,058112517	1,048695549	0,985552263	1,024216396	1,030290301	0,162145
P28650	PURA1	0,945499842	1,182015228	0,96172857	0,91075636	1,108501144	1,327261081	1,112709929	1,054338628	1,027686102	1,126099377	0,16215
Q9WTP7	KAD3	1,024376938	0,949217295	1,021702299	1,004703468	0,973978014	1,050771178	0,910654099	0,704834383	0,585938079	0,845235151	0,163451
O70591	PFD2	0,919287114	0,977786894	1,090229618	1,012696374	0,963170442	1,084158829	1,128716816	1,163564628	1,050139386	1,07795002	0,164433
Q8JZU2	TXTP	1,017272059	0,941829307	0,993939297	1,046959338	0,851334229	0,964762565	1,016711489	0,979554403	0,841779192	0,930828376	0,165637
Q8BH04	PCKGM	0,882408358	1,070510568	0,993074005	1,054007069	0,928697442	0,948781286	0,956972755	0,951972312	0,916622712	0,940609302	0,165934

O54734	OST48	0,998063739	0,987181074	0,994944379	1,019810808	1,020887528	0,796456332	1,013712591	0,905062434	0,895080751	0,926239927	0,166285
P26645	MARCS	0,833767695	0,988203904	1,17384467	1,004183731	0,814689061	0,872099301	0,96267532	0,840636758	0,9656661	0,891153308	0,166818
P16388	KCNA1	1,527126009	0,244937294	1,143786351	1,084150346	1,480597996	1,497135462	1,351983896	1,375423842	1,187856204	1,37859948	0,166892
Q9Z2H2	RGS6	1,034605962	1,025218491	1,036368041	0,903807505	1,327374989	1,16059849	1,065358586	1,121646387	0,930130825	1,121021855	0,167512
P27773	PDIA3	0,985379535	1,076284223	0,942105672	0,99623057	0,948130556	0,940829627	0,999543323	0,936216548	0,961498401	0,957243691	0,167614
G5E829	AT2B1	1,085249232	0,889278976	1,053333275	0,972138517	1,439111626	1,005033936	0,95213805	1,14298073	1,31702826	1,17125852	0,167658
Q7TMK9	HNRPQ	1,013027564	1,032708244	0,867195403	1,08706879	1,124455905	1,002006721	1,117498502	1,019164246	1,145818855	1,081788846	0,167724
P97494	GSH1	0,954342549	0,988020214	1,049711898	1,00792534	0,911443496	0,853853345	0,955868153	0,920080975	1,048427864	0,937934767	0,168306
P29758	OAT	0,957472538	0,977610224	1,040940632	1,023976606	0,953661172	0,900739611	0,987311229	1,014526539	0,918404151	0,954928541	0,168965
P85094	ISC2A	1,037568321	0,983931455	1,110290938	0,868209286	0,872765667	1,06138478	0,901106031	0,695241281	0,859038406	0,877907233	0,170052
Q91XM9	DLG2	1,077923218	0,953145935	1,013258584	0,955672263	0,889481392	0,891913016	0,880214595	0,892656726	1,072139388	0,925281023	0,1717
Q68FF6	GIT1	0,854744629	1,096303266	0,882089736	1,166862369	1,227212759	0,981860404	1,046307107	1,164000664	1,309539334	1,145784054	0,171752
Q9QXS6	DREB	0,877714236	0,954682649	1,134637924	1,032965191	0,932916093	0,766042176	0,889202208	0,925687713	0,994496753	0,901668989	0,171859
P23198	CBX3	0,747235364	1,154740494	1,137839571	0,960184572	0,087295782	0,939082502	1,065430683	0,739755444	0,562873863	0,678887655	0,171892
Q8R0S2	IQEC1	0,886280236	1,035297943	0,947337158	1,131084663	1,165721028	1,046284885	1,035186905	1,025640597	1,209255218	1,096417727	0,17336
Q99PJ0	NTRI	0,920983703	0,930291758	1,157650338	0,991074201	0,963190996	0,88686708	0,863881565	0,890203412	0,982860855	0,917400782	0,176436
Q61937	NPM	0,927662544	0,970586992	0,970237006	1,131513458	0,934741971	0,893357784	1,022951565	0,923344054	0,788234686	0,912526012	0,177295
P62746	RHOB	0,966328441	0,818649612	1,097884737	1,11713721	0,754321023	0,100984378	0,971797538	0,808544564	0,942896433	0,715708787	0,178567
P67871	CSK2B	1,051199357	0,939951572	1,047030898	0,961818173	1,121666664	0,875874584	0,877190244	0,788540139	0,593791285	0,851412583	0,179623
Q61735	CD47	1,360002493	0,86567823	0,928265502	0,846053775	1,098352703	1,263037995	1,086657518	1,205162184	1,188057077	1,168253496	0,180241
Q9Z2Y8	PROSC	0,89667322	0,753012403	1,142550588	1,207763788	0,908939962	0,99602219	0,982084715	0,182732557	0,101316495	0,634219184	0,182455
Q9QZ23	NFU1	1,147013193	0,65135011	1,069487438	1,13214926	0,945025407	0,683492204	0,91170417	0,906089212	0,549793829	0,799220965	0,182492
Q9QXS1	PLEC	1,001021201	1,003915676	0,994758274	1,000304848	1,161506179	1,021469951	0,978222747	1,030541895	1,067390582	1,051826271	0,182746
O70443	GNAZ	0,932527392	1,000159002	1,074665023	0,992648583	1,08662907	0,997361371	1,047833394	1,025402162	1,075958043	1,046636808	0,183343
Q9Z218	DPP6	0,891108861	1,033676499	1,05952142	1,01569322	0,985259809	1,0723363	1,091202896	1,035756229	1,202484491	1,077407945	0,183825
O89053	COR1A	0,95175131	0,990293537	1,077063778	0,980891375	1,01993749	0,886266177	0,906977332	0,932572447	0,984554831	0,946061655	0,185089
Q8VBY2	KKCC1	0,957841598	1,022398067	0,93011265	1,089647685	0,550429214	0,055571496	0,912173247	0,980267974	0,989669648	0,697622316	0,185644
Q3TTY5	K22E	0,695658052	1,027605592	1,2819565	0,994779856	1,57260912	7,314832263	2,470185674	1,920326251	1,137211296	2,883032921	0,186163
Q8QZT1	THIL	0,956596702	1,026374443	0,991425349	1,025603506	1,053164582	0,999660332	1,016349645	1,013603214	1,075485804	1,031652715	0,186726
Q8R3P0	ACY2	1,069965034	0,959343893	0,945110189	1,025580884	1,044370377	0,784999067	1,013600887	0,883592295	0,741641604	0,893640846	0,187148
P32020	NLTP	0,857848422	0,993077833	1,111575945	1,0374978	0,841640727	0,965170599	0,954773338	0,965588743	0,864010003	0,918236682	0,187313
F6SEU4	SYGP1	0,805321088	0,838349786	1,226809305	1,129519821	0,94294801	0,765617676	0,797020743	0,633383849	0,998224611	0,827438978	0,18754
Q8VDQ8	SIR2	1,115504659	1,014994015	0,800270115	1,069231211	1,155036683	1,242975716	1,217820873	1,127520068	0,912614467	1,131193562	0,189215
P68404	KPCB	1,104446794	1,009986351	0,915910116	0,969656739	1,173403083	0,891623994	1,069243729	1,184220972	1,297538514	1,123206058	0,190112
Q9Z2W9	GRIA3	0,804405351	0,872059324	1,362435016	0,961100309	0,971507141	0,755648614	0,566643353	0,809115652	0,915069455	0,803596843	0,191161
O54984	ASNA	0,951339198	1,024006222	1,048729895	0,975924684	1,009820747	0,905346206	0,971372507	0,948119286	0,967546852	0,96044112	0,19229
O35954	PITM1	0,962084751	1,03950966	1,034999922	0,963405667	1,125043278	1,018679459	0,999089676	1,036347217	1,043012236	1,044434373	0,193527
Q8K2C9	HACD3	0,979072126	1,084415905	0,960587956	0,975924013	0,969342732	0,944925674	0,636086141	0,898906556	0,991921034	0,888236428	0,193529
P62069	UBP46	0,970863265	0,893454607	1,173252025	0,962430103	0,904072038	0,75799449	1,0398402	0,823335785	0,928886862	0,890825875	0,193587
Q9Z140	CPNE6	0,556343567	1,040252029	1,135766353	1,26763805	0,694579812	0,433007567	0,94446169	0,838053283	0,867573535	0,755535178	0,194355

Q920I9	WDR7	0,959353934	0,99440047	1,035292816	1,01095278	1,044248954	0,887567207	0,90049124	0,935648353	0,979267644	0,94944468	0,194774
Q9CQI6	COTL1	0,783539007	1,086425841	1,057060072	1,07297508	0,766168834	0,839854234	1,007725056	0,942888893	0,877566506	0,886840705	0,196015
Q99LG2	TNPO2	0,940299011	1,041397877	0,975654711	1,042648402	0,951885028	0,661132073	0,784618683	1,109564673	0,858347407	0,873109573	0,196291
Q05920	PYC	1,014557044	1,022495413	0,970022123	0,99292542	1,097701799	1,112059836	1,04757301	1,034451661	0,950478217	1,048452905	0,196624
P51410	RL9	0,855030348	1,016614178	0,994239697	1,134115777	0,911288742	0,885922807	1,031873582	0,908974252	0,773429769	0,90229783	0,197122
P35293	RAB18	0,862689446	1,030791346	1,055632377	1,050886831	0,91493417	0,863040057	1,030086648	0,963238834	0,759732946	0,906206531	0,197442
P62962	PROF1	0,827495646	1,075672273	1,076518376	1,020313705	0,905602709	0,906821175	0,979769286	0,924134722	0,895442735	0,922354125	0,197667
Q8R464	CADM4	1,032621852	1,006614173	0,897956876	1,062807099	0,971464232	1,097565911	1,190239427	1,099021975	1,018827933	1,075423895	0,197759
P60824	CIRBP	0,987293312	0,932209564	1,151080427	0,929416697	0,873255839	0,910096926	0,988696197	0,969020831	0,884634822	0,925140923	0,197917
Q80TJ1	CAPS1	0,938093039	0,967602567	1,05555459	1,038749803	1,137709314	0,971622203	1,023692488	1,046272656	1,131852629	1,062229858	0,199167
Q921F2	TADBP	1,135124822	0,923114077	0,86622986	1,075531241	1,152584775	1,170696804	1,069543365	1,020441093	1,045972155	1,091847638	0,199481
Q8K310	MATR3	0,800398426	1,094851637	0,982287765	1,122462172	1,247793009	1,101847893	1,131561901	0,967192409	1,131563937	1,11599183	0,199634
Q8C522	ENDD1	1,049313871	1,065367236	0,960046903	0,92527199	0,96165137	0,894583879	1,000804602	0,98268741	0,839628821	0,935871216	0,199821
O88342	WDR1	0,925815782	1,059264482	0,931727116	1,08319262	0,976505272	0,895837477	0,975264854	0,925382193	0,942244324	0,943046824	0,200738
P35505	FAAA	1,08264129	1,022413911	0,899532487	0,995412312	1,060210716	0,464423939	0,942189776	0,830893476	0,866941122	0,832931806	0,201031
Q9DBT5	AMPD2	0,923234404	1,118485785	0,939612976	1,018666835	0,958778606	0,90035999	0,890804846	0,803590595	0,024067473	0,715520302	0,201593
O08539	BIN1	1,040460607	1,051566179	0,924254429	0,983718785	1,083472224	0,938372658	1,084071175	1,147910631	1,074895645	1,065744467	0,202499
Q62465	VAT1	0,917823019	1,030116959	1,02145085	1,030609172	0,798478853	1,013790546	0,855096768	0,967495653	0,99151341	0,925275046	0,203099
Q7TN29	SMAP2	0,935159383	1,104062906	1,069766801	0,891010909	0,871849821	0,906883548	0,871700346	0,940210677	1,022143465	0,922557571	0,203455
Q6NZL0	SOGA3	1,307918582	1,358382521	1,145315762	0,188383136	1,533414749	1,539666753	1,369686859	1,170562811	1,1913065	1,360927535	0,203463
Q8K0S0	PHYIP	0,802935805	1,001752089	1,174001686	1,02131042	0,936226347	0,837559748	0,874598413	0,809139008	0,999619849	0,891428673	0,203561
P23242	CXA1	1,045346345	0,937852875	0,912095029	1,104705751	0,941667276	0,810309973	1,095365829	0,856060155	0,717977456	0,884276138	0,204427
Q8C854	MYEF2	0,838193963	1,095013788	1,056970353	1,009821896	1,314503852	0,899158543	1,007386024	1,316527013	1,217149173	1,150944921	0,204552
Q9Z1G4	VPP1	0,931186591	1,012005915	1,037135738	1,019671756	1,111188524	1,012871226	0,997369276	1,011940941	1,146265657	1,055927125	0,206287
Q91YJ2	SNX4	0,877502149	1,02027717	1,01000682	1,092213861	0,854057065	0,653092246	0,88267058	1,020883897	1,001674353	0,882475628	0,206415
P14148	RL7	0,856438797	1,004389674	1,09070622	1,048465309	0,877713189	0,930507062	1,030815731	0,885301909	0,892233844	0,923314347	0,206949
Q9R1P0	PSA4	0,966490681	1,012956754	0,965995619	1,054556946	0,911102922	0,419467942	0,91582266	1,078721961	0,79237543	0,823498183	0,207252
O35658	C1QBP	1,023618849	0,974310613	0,978686498	1,023384041	0,99885019	1,003411289	0,994416738	0,870740439	0,830715735	0,939626878	0,207526
Q61598	GDIB	0,989576638	1,024755022	0,999762067	0,985906273	1,009664189	0,943489961	1,116973541	1,103585314	1,093365422	1,053415685	0,207995
Q9QXV0	PCSK1	0,7896073	0,941125801	1,145192234	1,124074665	0,676065354	0,771312455	1,034188884	0,950912563	0,855214565	0,857538764	0,208489
P63321	RALA	1,032502857	0,962474917	1,001191778	1,003830447	1,063449298	1,074870607	1,069402741	0,998578577	0,976219459	1,036504136	0,209098
Q8BSL7	ARF2	0,903478107	1,125415624	1,002926293	0,968179976	0,95272279	0,435472429	1,050912707	0,966536431	0,634056262	0,807940124	0,209297
Q91WD5	NDUS2	1,040988458	0,999606454	0,995351503	0,964053585	0,931304141	0,850124766	0,995832029	0,969888209	1,009357122	0,951301253	0,20961
Q8C3Q5	SHSA7	0,886191097	0,95639637	1,122782111	1,034630422	0,971289911	0,771835691	0,951751853	0,979723423	0,893656762	0,913651528	0,209638
Q91YR1	TWF1	0,956717684	1,040369827	0,897862937	1,105049552	1,06657598	0,933566153	0,921468639	0,778785625	0,818265839	0,903732447	0,209811
Q61481	PDE1A	0,933328039	0,966482339	1,100377928	0,999811694	0,930167611	0,777362154	0,985011606	0,972892488	0,96388166	0,925863104	0,209943
Q9Z239	PLM	1,202810692	1,416667991	1,290118603	0,090402713	1,17910882	1,855328197	1,628841216	1,165363595	1,30884431	1,427497228	0,210264
Q60854	SPB6	0,961338857	1,077812058	0,94044062	1,020408465	1,024834041	1,030379595	1,099719525	1,000292493	1,081364793	1,04731809	0,211528
P21995	EMB	0,952594171	1,074909807	0,927662836	1,044833186	1,056725376	1,017761509	1,114694481	0,98983783	1,10591216	1,056986271	0,212695
Q8CGY8	OGT1	0,926570806	0,976806821	1,071686797	1,024935576	0,980304801	0,828376317	0,960328299	0,918279964	1,006476257	0,938753128	0,21289

Q8BG39	SV2B	0,99328751	0,854788327	1,136279064	1,015645099	1,257444856	0,992330656	1,044390463	1,022686775	1,255046042	1,114379758	0,212965
Q9CPX8	QCR10	0,902176982	1,139828434	1,076169132	0,881825451	0,959751086	0,843891852	0,851995981	1,042212242	0,761354679	0,891841168	0,213157
P23818	GRIA1	0,457239054	1,155324883	1,07305276	1,314383303	0,75707558	0,669514302	0,71950059	0,032333589	1,080171577	0,651719128	0,213169
Q8BXZ1	TMX3	1,020563596	1,077599579	0,891390796	1,010446028	0,927935779	0,573895728	1,050509659	0,964978889	0,799415048	0,863347021	0,214299
Q9JMA1	UBP14	0,878483252	1,172878381	0,923247535	1,025390832	0,94922197	1,033535606	1,190030304	1,346993309	1,13691701	1,13133964	0,214342
P63101	1433Z	1,074536165	0,955309757	0,971903476	0,998250601	1,07124231	1,134195859	1,012577515	1,011455856	1,015146502	1,048923609	0,214479
P06801	MAOX	1,019140202	1,05151657	0,916138032	1,013205196	1,035637281	0,999514793	1,120381847	1,082061503	1,010297266	1,049578538	0,214682
Q9CQW2	ARL8B	0,976522843	1,109423214	0,85144167	1,062612273	0,822677517	0,69393882	1,073291809	0,949064253	0,865162759	0,880827032	0,214842
P16332	MUTA	1,024092498	1,031681266	0,860475982	1,083750253	0,969160302	1,113914703	1,070324894	1,146434095	1,070461233	1,074059045	0,21509
Q8R016	BLMH	1,016268053	1,00874452	0,993480588	0,981506839	1,058577741	0,952308176	1,060852808	1,065112946	1,032471552	1,033864645	0,217057
Q9JM14	NT5C	0,831965415	0,912045883	1,075162629	1,180826073	0,906706616	0,879782022	0,943451064	0,897034257	0,897258513	0,904846494	0,217111
Q8CA95	PDE10	1,372078473	0,9952104	0,988376882	0,644334244	1,118095141	1,136474728	0,989347709	1,4474875	1,44771061	1,227823138	0,217147
Q9D023	MPC2	1,116012763	0,983180752	1,014794226	0,886012259	0,954250238	0,976209854	0,948305244	0,947870127	0,785093449	0,922345782	0,217302
P62918	RL8	0,873391629	0,949721169	0,968728987	1,208158214	0,916262907	0,985698274	1,000903353	0,774012908	0,669073137	0,869190116	0,217709
Q9R0Y5	KAD1	0,982886616	0,99536137	1,02654761	0,995204404	1,003481925	0,993600923	0,995742942	0,942484386	0,891212654	0,965304566	0,218288
Q9JHU9	INO1	0,778311109	0,957153574	1,123660591	1,140874725	0,738802527	0,021895288	0,87655565	0,979135809	0,945660155	0,712409886	0,221994
Q8CAY6	THIC	0,702618088	1,02113476	1,167983046	1,108264106	0,974052187	0,780156678	1,03747788	0,55513729	0,763018287	0,821968464	0,222287
Q9D7H3	RTCA	0,933446331	1,181880837	0,995094304	0,889578528	0,845045419	0,064626031	0,965697046	0,921815805	0,869419196	0,733320699	0,222547
P47757	CAPZB	0,889297643	0,973970268	1,003725865	1,133006224	0,993936944	1,04660692	1,11292128	1,109608685	1,078040539	1,068222874	0,222664
P35564	CALX	0,977148727	1,065119931	0,965252386	0,992478957	1,011697891	1,07605904	1,048427432	0,978806271	1,080888958	1,039175918	0,226993
P61161	ARP2	0,831425455	1,020379981	1,051635592	1,096558972	1,059462984	0,961656097	1,125290555	1,117701973	1,167847903	1,086391902	0,227115
Q61644	PACN1	0,953320655	1,03072435	1,033399732	0,982555263	1,060988561	1,013796131	1,044048611	0,979775585	1,06839843	1,033401464	0,22714
Q6P5F9	XPO1	0,984194484	1,015722279	0,929494145	1,070589092	0,864213737	0,897062012	0,955189103	0,845672758	1,078918535	0,928211229	0,22727
Q9DCT1	AKCL2	1,236325722	1,341184751	1,355038893	0,067450634	1,191860084	1,438691126	1,5513694	1,466488472	1,237233756	1,377128568	0,227413
Q9EPL8	IPO7	0,843973411	0,994851426	1,184906117	0,976269045	0,887346519	0,888077142	0,892365267	0,909444597	0,989909611	0,913428627	0,227883
Q8C0P5	COR2A	1,378163167	0,870140621	0,834915795	0,916780418	0,956889242	0,828266583	0,768417134	0,912015231	0,737860045	0,840689647	0,230207
Q9WVE8	PACN2	0,852309887	1,161055298	1,012186964	0,974447852	1,142976011	0,919181883	1,347411213	1,38031034	0,990042627	1,155984415	0,230355
Q8BWT1	THIM	0,979804537	0,983077131	1,056444173	0,98067416	0,197440846	0,865455483	1,000166409	0,796156691	1,016943149	0,775232516	0,230514
P16330	CN37	1,107190537	1,069513411	0,822701804	1,000594248	1,118026084	1,131558262	1,17979052	1,15188067	0,919501184	1,100151344	0,231075
Q9R1V6	ADA22	1,028481767	1,025420269	1,099095798	0,847002166	0,936229561	0,900791205	0,927667731	0,809963928	1,024196641	0,919769813	0,231526
Q62059	CSPG2	0,984704008	1,080910068	0,958313701	0,976072222	0,867857358	0,680393714	0,847653758	0,938022465	1,117360262	0,890257511	0,231815
P62259	1433E	1,036247878	0,954274604	0,939864269	1,069613249	1,087103249	1,204029829	1,121213648	1,09327775	0,902760447	1,081676985	0,23191
Q9CQ92	FIS1	0,890747987	1,14337256	1,025339173	0,94054028	1,038540978	1,608523401	1,14517227	0,989903387	1,101484232	1,176724854	0,232044
Q9Z1R2	BAG6	0,891798312	1,020866351	1,073649972	1,013685365	0,985830524	0,911578638	0,919886854	0,828044228	1,022277743	0,933523597	0,232092
Q8BP47	SYNC	0,805251671	1,037149472	1,042578704	1,115020152	0,904529478	0,677533045	0,976541663	0,963630705	0,919287405	0,888304459	0,23223
Q9ERI6	RDH14	1,054306307	0,921745873	0,962790188	1,061157632	0,960351453	1,097487879	0,788915972	0,789165971	0,892340758	0,905652407	0,233301
O08583	THOC4	0,884447311	0,995918291	1,101894684	1,017739714	1,205334834	1,290968483	1,154093694	0,829104743	1,154959961	1,126892343	0,23354
Q61016	GBG7	1,002616697	0,819798742	1,387159845	0,790424716	1,030888233	1,138297336	1,040224455	1,307613007	1,537763974	1,210957401	0,234596
P54830	PTN5	1,061215054	1,053815332	1,083828419	0,801141194	1,0370675	1,00398173	0,976716292	1,362145346	1,600338961	1,196049966	0,235212
P31786	ACBP	0,694050723	0,992372498	1,195626353	1,117950427	0,584972287	0,680430382	0,951124606	1,006518962	0,907510602	0,826111368	0,236052

P45952	ACADM	0,998562616	0,962520018	1,021230603	1,017686763	0,976012301	1,025163422	0,987495468	0,902219398	0,934766026	0,965131323	0,236302
O88569	ROA2	0,954055694	0,990320026	0,994218064	1,061406216	1,015530883	0,90906608	0,979235575	0,951953702	0,964193766	0,963996001	0,237011
Q9DCH4	EIF3F	0,92897551	0,98972536	1,079150639	1,002148491	1,002323644	0,843539614	0,922986814	0,945898036	1,005622229	0,944074067	0,237441
P11983	TCPA	0,925247636	1,056414802	0,957094066	1,061243496	1,03349804	0,98684792	1,075462316	1,15765343	1,033921153	1,057476572	0,237745
Q6PFR5	TRA2A	0,947734373	0,93718212	1,089431364	1,025652143	0,906552377	0,484450269	0,967275692	1,047684458	0,854420528	0,852076665	0,238493
O55026	ENTP2	0,838511226	0,977014426	1,078570578	1,105903769	0,720047529	0,639265076	0,948143226	1,000919803	1,024745118	0,86662415	0,238751
P63216	GBG3	0,795422965	0,912539213	1,342350886	0,949686936	0,757772797	0,067709871	0,827017975	0,810142851	1,109540567	0,714436812	0,239424
P28652	KCC2B	0,927437145	1,10215508	0,973829756	0,996578019	1,070693856	0,969162155	1,019017935	1,093414413	1,188240696	1,068105811	0,239578
Q7TSJ2	MAP6	1,107314566	1,04340728	0,901649445	0,947628709	1,034408412	0,836434704	0,956048905	0,901746282	0,920374244	0,929802509	0,242489
Q9DBP5	KCY	0,972987967	0,964959298	1,016851931	1,045200805	1,014774012	1,000684873	1,063872157	1,064677381	1,004060934	1,029613871	0,243145
Q91X97	NCALD	1,198545963	0,887377284	1,013483132	0,900593621	1,135440313	0,718367241	1,00996251	0,675853487	0,632924634	0,834509637	0,243597
P34022	RANG	0,986139014	0,932785132	1,03908261	1,041993245	0,923572668	0,867904337	1,03231032	0,958494968	0,974537587	0,951363976	0,245279
Q62277	SYPH	0,887773204	0,862732767	1,127395987	1,122098043	0,787677832	0,919796076	0,884589394	0,94743191	0,989579287	0,9058149	0,245593
P19096	FAS	0,940301837	1,064172836	0,984580866	1,010944462	1,108312593	1,13810853	1,018741877	0,99318619	0,999882396	1,051646317	0,246167
Q9D5T0	ATAD1	0,963535932	1,119771109	0,846145367	1,070547592	0,924394284	0,898868487	0,846712991	1,07492724	0,731590228	0,895298646	0,246446
P42932	TCPQ	0,924244275	1,093082122	0,969054163	1,01361944	1,046335715	0,97822896	1,071222644	1,127939832	1,040833781	1,052912186	0,246841
Q60625	ICAM5	0,448358711	0,78691433	1,611465995	1,153260964	0,684444433	0,430980081	0,593046633	0,654456114	1,064774581	0,685540368	0,24724
Q63912	OMGP	0,929314268	0,904777954	1,045184772	1,120723006	0,793553956	0,614093286	0,915219494	0,922453471	1,108036539	0,870671349	0,247504
Q91VR7	MLP3A	0,795106374	0,96779521	1,214842643	1,022255774	0,9680735	1,000213864	1,253077938	1,326956231	1,142092952	1,138082897	0,247676
Q91V61	SFXN3	1,053691743	0,923261474	1,11044821	0,912598572	0,979065853	0,897545964	0,881699713	0,905757637	1,011133446	0,935040523	0,24857
Q9DCS9	NDUBA	1,038508687	0,971342046	0,966128902	1,024020366	0,936123174	0,976477045	1,037867748	0,951631856	0,918986268	0,964217218	0,248918
O35682	MYADM	1,156943351	0,94670385	1,023997071	0,872355728	1,058776801	0,958076381	0,90949936	0,786141876	0,786769487	0,899852781	0,249197
P03930	ATP8	0,75368758	1,062303752	1,245445231	0,938563437	0,817168999	0,786630447	0,958289062	0,995320919	0,789946824	0,86947125	0,249686
Q3TXS7	PSMD1	0,526332809	1,388553874	1,325276486	0,759836831	1,35550945	1,130832946	1,364852765	1,182160128	1,18161065	1,242993188	0,250935
P70188	KIFA3	1,037752072	1,025366412	1,02422933	0,912652186	0,968103627	0,897146226	1,074301947	0,768585816	0,904404298	0,922508383	0,252495
P70336	ROCK2	1,021168374	1,018580736	0,981638414	0,978612476	1,155442578	1,02304604	0,985120444	0,979095966	1,118504602	1,052241926	0,252779
Q6PGN3	DCLK2	1,075778418	1,235632902	0,671781548	1,016807132	1,22432417	1,130144209	1,117280732	1,03407917	1,182711781	1,137708012	0,252858
Q8BMS1	ECHA	1,02501808	1,073492857	0,871475642	1,030013421	1,002490177	1,203928735	1,114510318	1,019922329	1,02152764	1,07247584	0,253331
Q9CQD1	RAB5A	0,961202514	0,966031895	1,082502004	0,990263587	0,948632253	1,02338107	1,013730721	0,858892352	0,860832602	0,9410938	0,253442
Q2PFD7	PSD3	0,989916443	0,799100649	1,202527702	1,008455206	0,826903623	0,829186791	0,957703951	0,916715708	0,970217704	0,900145555	0,254017
P97447	FHL1	0,956735861	0,864345117	1,024870698	1,154048324	0,855589362	0,972681204	0,965189543	0,936746867	0,904506662	0,926942727	0,254416
P63094	GNAS2	1,001517593	1,037884538	0,869361401	1,091236468	0,920560837	0,793508414	1,0301921	1,030025903	0,618481676	0,878553786	0,255197
P27546	MAP4	1,104124312	1,036690018	0,902889355	0,956296315	1,00380354	0,936787793	1,000436382	0,805490251	0,901468228	0,929597239	0,255591
P11798	KCC2A	0,84321134	0,969379134	1,161621372	1,025788155	0,887632364	0,687983168	0,95444862	0,956021434	0,990527433	0,895322604	0,256377
P47963	RL13	0,848636627	1,003077791	1,01911223	1,129173352	0,945357259	0,925151179	1,01256938	0,880159865	0,880513381	0,928750213	0,257501
Q922J6	TSN2	1,080798724	0,990424229	0,79723607	1,131540977	1,031392744	1,536462367	1,321021507	1,080723097	0,909837099	1,175887363	0,257573
P52196	THTR	1,013765694	1,004010856	0,963168131	1,019055318	1,01382547	0,984110293	0,996567887	0,907513647	0,744729644	0,929349388	0,257656
P48758	CBR1	0,964683948	0,979764297	1,004076778	1,051474976	1,013273283	1,047314917	1,130088255	0,989073067	1,01923225	1,039796354	0,257726
Q6PDL0	DC1L2	1,105394605	1,010551392	0,945791927	0,938262076	1,054066521	0,896051059	0,941037697	0,943061689	0,849263502	0,936696093	0,257888
Q9CPQ8	ATP5L	0,879255648	1,036113238	1,180538512	0,904092602	0,731384848	0,928435757	1,054475919	0,850151831	0,910231455	0,894935962	0,257936

Q9DD18	DTD1	0,990821269	0,944984001	1,015468842	1,048725888	0,974000528	1,04048388	0,983337539	0,885374577	0,816698795	0,939979064	0,258439
P46097	SYT2	0,990023669	1,116228712	0,915935413	0,977812205	0,9819252	0,981641211	1,039556419	0,703299544	0,788575431	0,898999561	0,258912
P48725	PCNT	0,947794872	1,008660116	1,019208721	1,024336291	0,96421912	0,943203546	0,99273182	1,010767686	0,962119435	0,974608321	0,259033
P60879	SNP25	1,106395764	0,978704477	0,926291244	0,988608514	1,108733763	0,971063552	1,091751327	1,087968897	1,01558455	1,055020418	0,259359
P62751	RL23A	0,964780994	1,085228492	0,935076895	1,014913619	0,634749135	0,963909077	0,956767098	0,967377475	0,98102613	0,900765783	0,259404
O35295	PURB	0,849665012	0,982780319	1,01312426	1,154430409	0,942589197	0,877350825	0,972187222	0,991425024	0,803127284	0,91733591	0,259767
P63330	PP2AA	0,925132522	0,922439988	1,04168319	1,1107443	0,99832626	0,956370522	0,946000191	0,943017812	0,869158759	0,942574709	0,261074
Q6P5E4	UGGG1	0,952567644	1,132139885	1,092976521	0,822315949	0,922822938	1,020461856	0,881441901	0,945778583	0,568344393	0,867769934	0,261329
Q9QZ06	TOLIP	0,933764182	1,000538193	0,998326048	1,067371576	1,002309202	0,950462856	0,978474397	0,981721995	0,870532325	0,956700155	0,261732
P04370	MBP	1,01566364	1,099579885	0,828550051	1,056206425	1,110716011	1,092185099	1,124601858	1,134558768	0,941320265	1,0806764	0,261904
O35526	STX1A	1,077953763	0,870906613	1,128921608	0,922218015	1,295211763	1,050708367	0,993988544	1,058842433	1,087637159	1,097277653	0,262185
Q9R1P3	PSB2	1,080484271	0,90956696	0,761573894	1,248374875	0,792473373	0,571899668	1,006092269	0,974063835	0,878069808	0,84451979	0,26333
P56379	68MP	0,846834619	0,977647486	1,34370915	0,831808745	0,868393008	1,121260888	1,317456785	1,28013364	1,265528913	1,170554647	0,263897
Q8BL65	ABLM2	0,97126792	0,917828932	1,115478743	0,995424405	0,95743865	1,009671432	0,961782899	0,872830257	0,927064642	0,945757576	0,264289
Q8R3V5	SHLB2	0,937126515	1,036014109	1,072001132	0,954858244	0,967189276	0,872155671	0,992158329	0,881504098	1,020481085	0,946697692	0,265717
P14206	RSSA	0,915890155	1,039050719	1,045912221	0,999146905	0,978362579	0,895624797	1,00173863	0,949289518	0,973898937	0,959782892	0,266242
P19536	COX5B	0,946981231	0,885892105	1,095353713	1,071772952	1,002814647	1,051907538	1,225518924	1,097475846	1,007478986	1,077039188	0,267138
P68510	1433F	0,974207919	0,990985248	1,006043821	1,028763012	1,023829971	0,983494547	1,008326252	0,887662654	0,79273262	0,939209209	0,26753
Q8BVE3	VATH	0,940652518	1,016507986	1,038520845	1,004318651	0,939481326	0,927693447	0,981136519	0,978783445	1,017120405	0,968843028	0,268057
Q61704	ITIH3	1,045664238	1,191010625	0,843415316	0,919909821	0,836357161	0,992158152	0,797384351	0,94340411	0,954852944	0,904831344	0,268085
P08226	APOE	1,101818688	1,008362497	0,953623597	0,936195218	0,975735023	0,926157836	0,993445181	0,961333372	0,927291511	0,956792585	0,269427
Q8BYI9	TENR	0,939311915	1,037997449	1,019706069	1,002984566	1,121575424	1,000206046	0,992212996	1,040292785	1,037938939	1,038445238	0,27015
Q8CHP8	PGP	0,941262574	0,855545129	0,975666448	1,227525849	1,100697542	0,993333334	1,159475609	1,205029653	1,036335671	1,098974362	0,270926
Q9CXS4	CENPV	0,872977074	1,049846269	1,114241191	0,962935465	0,915624699	0,88037	1,018773917	0,909595346	0,957028856	0,936278564	0,27255
Q9JHI5	IVD	0,959034413	1,039836814	0,979928521	1,021200251	0,899672074	1,017192436	1,023245557	0,955322871	0,895514255	0,958189438	0,272711
O70161	PI51C	1,018961708	1,03415228	0,89848325	1,048402762	1,145807662	0,972298995	0,980615584	1,052369362	1,210931793	1,072404679	0,272885
Q8VE22	RT23	1,042739591	0,96645788	1,167142162	0,823660367	0,665373286	0,873364911	1,040014382	0,994454298	0,848348699	0,884311115	0,273858
Q9DBE8	ALG2	1,023174538	1,026348076	0,805565494	1,144911893	0,957110701	0,922345922	0,937413082	0,978011392	0,737316559	0,906439531	0,275974
Q80XI4	PI42B	0,881703263	1,036280899	1,068839766	1,013176072	1,080575295	0,92198275	1,131098872	1,204574012	1,041032681	1,075852722	0,278168
Q9QZQ8	H2AY	0,912386548	1,041456229	0,953921848	1,092235375	0,975436889	0,865263532	1,044758448	0,919472589	0,896462468	0,940278785	0,278258
Q9CXW4	RL11	0,884491585	1,009737853	1,145656225	0,960114337	1,006216951	0,896744477	0,977788742	0,86786012	0,128478829	0,775417824	0,27995
Q810U3	NFASC	0,99381787	1,060987902	0,985647768	0,959546461	1,06884352	1,072513719	1,016111874	0,981697858	1,019892675	1,031811929	0,280831
Q9D8N0	EF1G	0,949556613	1,047044006	1,027106771	0,976292609	0,973859468	0,923366449	1,024103249	0,971416235	0,947202915	0,967989663	0,280837
Q4KMM3	OXR1	0,848846445	1,063324942	0,975635705	1,112192907	1,211030587	0,991026278	1,026254926	1,029403274	1,146796631	1,080902339	0,282219
Q9WTP6	KAD2	1,001166104	0,994222261	1,000918023	1,003693612	0,915821683	1,016680079	0,561258227	1,022516043	0,92597437	0,88845008	0,283815
Q8BJU0	SGTA	0,95786202	0,975029692	1,117360094	0,949748194	1,014731142	0,984230922	0,948609507	0,933647908	0,834462923	0,943136481	0,284448
P42208	SEPT2	1,046541435	0,96754842	0,973524074	1,012386071	1,025283317	0,945479441	1,078883053	1,236240299	1,042509479	1,065679118	0,284467
P35486	ODPA	1,014720568	0,980273943	0,996078301	1,008927188	0,996675545	0,910831566	1,022233363	0,994770462	0,930239536	0,970950095	0,285794
Q80U28	MADD	0,924711534	1,037007743	0,966623845	1,071656878	1,091639117	0,797065879	0,684478051	0,9322364	0,99386316	0,899856521	0,285925
Q61425	HCDH	1,151579991	0,95712367	0,863520369	1,027775969	1,076089424	1,296259616	1,004403444	1,114184717	0,985072745	1,095201989	0,286096

Q8R1Q8	DC1L1	1,060893819	1,108551587	1,087066268	0,743488326	1,179166878	1,033043095	1,115738286	1,070867566	1,066868795	1,093136924	0,286929
Q8K221	ARFP2	0,999281616	0,975707563	1,035997302	0,989013519	1,042769423	0,746133826	0,955731894	0,842574707	1,033999331	0,924241836	0,287844
P08249	MDHM	0,987343822	0,959575076	0,979220813	1,07386029	0,918492565	1,081876444	0,918970329	0,901350424	0,927828761	0,949703704	0,288887
P62264	RS14	0,868512951	1,042387321	1,111391417	0,977708311	0,912011673	0,934822709	1,053330372	0,778711067	0,936641157	0,923103396	0,290104
P19157	GSTP1	0,97868995	0,966040634	1,006938039	1,048331378	1,008478708	1,063778403	1,166872648	1,111091923	0,930292056	1,056102748	0,290405
P10637	TAU	1,002064017	1,031826941	0,951691581	1,01441746	1,038175528	0,977201374	0,934566497	0,860925816	0,98013637	0,958201117	0,290781
P17710	HXK1	0,958786277	1,053003085	0,984888476	1,003322161	1,008259821	1,034511831	1,028154581	0,987248607	1,096940845	1,031023137	0,292313
P63318	KPCG	0,959377124	0,942429838	1,054718144	1,043474894	0,692808147	0,873750099	1,019403302	0,956193515	1,032543835	0,91493978	0,293654
P14094	AT1B1	1,011990288	0,998484437	0,995845432	0,993679843	0,91189385	1,017583173	0,977279777	0,983685384	0,994234288	0,976935294	0,293985
Q9CZM2	RL15	0,680948128	1,080935542	1,006739028	1,231377303	0,851432649	0,924169234	1,02817688	0,744299215	0,791023096	0,867820215	0,295072
O08529	CAN2	0,949794587	1,10063144	0,913233063	1,036340909	0,699737061	0,979805042	0,962181928	0,996312149	0,9511196	0,917831156	0,295394
P35802	GPM6A	0,911851547	0,895008908	1,158528179	1,034611366	0,9929654	1,041182204	0,979362606	0,702852459	0,740832936	0,891439121	0,296102
P01831	THY1	0,991433465	0,94665173	1,046896403	1,015018403	1,031338684	0,976983299	0,996713202	0,909770629	0,870981357	0,957157434	0,296859
P43276	H15	0,917391717	0,900314562	1,057934294	1,124359427	0,971493852	1,13732669	1,091185865	0,996797826	1,166672729	1,072695392	0,297623
Q9QYS2	GRM3	0,921305877	0,851768733	1,093865145	1,133060245	1,195607134	0,782971772	1,121429644	1,245888818	1,358130732	1,14080562	0,298316
Q9D8W5	PSD12	0,976927117	0,874090981	1,148765449	1,000216453	0,98440502	0,997656649	1,128671608	1,139783909	1,097722322	1,069647902	0,299675
O55022	PGRC1	0,828700313	0,972017383	1,105754101	1,093528203	0,770803529	0,922256008	1,04294502	0,936335688	0,907112623	0,915890574	0,299814
Q8CHH9	SEPT8	1,046824486	1,046560105	0,910769604	0,995845806	1,112875456	0,975955627	1,037668228	1,094926143	1,006345317	1,045554154	0,29983
Q9EP69	SAC1	1,126349009	1,084172691	0,86731449	0,92216381	0,861124287	1,00246845	0,968501426	0,953256806	0,857193164	0,928508826	0,301911
Q7TQF7	AMPH	0,929491602	0,991169849	1,088916433	0,990422116	1,042812987	0,885379748	0,897173435	0,843813556	1,029476408	0,939731227	0,302858
Q9WVJ2	PSD13	1,126068113	0,909607412	1,011244478	0,953079997	0,996928224	0,890308212	1,090626102	0,816950981	0,773933213	0,913749346	0,304008
P19246	NFH	1,246573891	1,127523352	0,727277172	0,898625585	1,261531337	1,362815231	1,123658903	1,12093706	0,88770393	1,151329292	0,30417
Q9WV85	NDK3	0,775881123	1,117516126	1,036010503	1,070592248	0,916856601	0,084367932	1,022279587	0,871843794	0,954420452	0,769953673	0,304291
P18872	GNAO	0,941484912	0,941696495	1,068464282	1,048354311	1,023754413	1,025060638	1,071640462	1,049851626	1,009107777	1,035882983	0,304322
Q8CIE6	COPA	0,91897358	0,952264834	1,107475698	1,021285888	0,990876981	1,008320102	0,797791115	0,042201606	0,986930067	0,765223974	0,306204
Q3TYX3	SMYD5	0,952456875	0,946684618	1,112615112	0,988243395	1,01179029	0,996284973	0,91727207	0,959964052	0,859994958	0,949061268	0,306322
P58281	OPA1	0,954088294	1,017791652	1,0387951	0,989324954	1,022605547	1,003952631	1,015903276	1,012156691	1,227017296	1,056327088	0,306923
Q9Z110	P5CS	0,938501754	1,095451299	1,018120844	0,947926102	1,084838122	1,13398481	1,04969204	0,977735769	1,002297155	1,049709579	0,307224
P03995	GFAP	0,950430432	1,210816101	0,785127365	1,053626102	0,892421912	0,833555618	1,07193537	0,910705947	0,743365358	0,890396841	0,30733
Q6ZQ38	CAND1	0,913551585	1,017427879	1,038818544	1,030201993	1,101128614	1,119933529	0,995405598	0,948583508	1,084606628	1,049931575	0,308502
O35593	PSDE	0,806509746	1,076943808	1,089249768	1,027296678	0,962843648	0,981399795	0,972791445	0,896074149	0,812676219	0,925157051	0,308982
Q99KC8	VMA5A	0,909759023	1,137762987	0,921450461	1,031027529	0,869704447	0,872362727	1,014389241	0,976781118	0,953837956	0,937415098	0,309757
P70280	VAMP7	0,934105355	0,940708721	1,02510635	1,100079574	0,920862105	0,726925376	0,941715061	1,133039044	0,812340594	0,906976436	0,30999
P45376	ALDR	0,618738693	0,969256781	1,316907532	1,095096994	0,951005234	0,774499531	0,851741463	0,871868282	0,826635171	0,855149936	0,310062
Q920E5	FPPS	1,011599955	0,991463842	0,921743037	1,075193165	1,053312856	0,946562336	0,868465982	0,948669893	0,111313985	0,78566501	0,31039
P62267	RS23	0,767277103	1,197011831	0,914784703	1,120926363	1,003834332	1,507126281	1,281276546	0,990896808	1,00541862	1,157710518	0,312676
P30416	FKBP4	1,023550457	1,071475086	0,970635254	0,934339203	0,986614882	1,02739179	0,976046224	0,929383454	0,807227924	0,945332855	0,314188
P35235	PTN11	0,984883074	1,116371993	0,906753647	0,991991286	1,053006006	1,046015904	1,024850459	1,081392063	1,013368938	1,043726674	0,314339
P00493	HPRT	0,883366681	1,020267538	1,014204491	1,082161289	0,944837864	0,984270537	1,148352906	1,271521059	1,062912679	1,082379009	0,314725
Q505F5	LRC47	1,153755142	0,95383547	0,853829398	1,038579989	1,006146138	0,19475927	1,01471652	0,828112755	0,95889359	0,800525655	0,315486

P21107	TPM3	0,983162013	1,030171321	1,018028741	0,968637926	1,001277835	0,750221426	1,018668575	0,964778444	0,960269781	0,939043212	0,315615
P62821	RAB1A	1,333905786	1,037536361	0,379015044	1,249542809	1,228406774	1,531628514	1,098638536	1,183907241	1,094269847	1,227370183	0,315972
Q9JMH9	MY18A	1,264070634	1,376713215	1,338052715	0,021163435	1,466976411	0,858410857	1,415876828	1,558588707	1,427882349	1,34554703	0,316055
Q922E4	PCY2	0,878767141	1,030615446	1,034591031	1,056026381	1,004540164	0,99482699	0,851575593	0,884140954	0,990398747	0,94509649	0,317555
P56375	ACYP2	0,871241293	1,058072453	1,20649948	0,864186773	0,845645858	0,855156883	1,051013061	0,85857137	0,93741296	0,909560027	0,320799
Q06138	CAB39	1,359205116	0,450590325	0,920092074	1,270112485	1,246178126	1,289214978	1,124040199	1,352294321	1,019909354	1,206327396	0,321325
P63038	CH60	1,010910615	1,034197604	0,928904204	1,025987577	0,960387226	1,007019349	0,964871894	0,949877555	0,988386087	0,974108422	0,321546
Q8BH44	COR2B	1,162966831	1,040244336	0,850810437	0,945978395	0,966589432	0,770346575	0,900917198	1,021179701	0,938149761	0,919436533	0,321809
Q8BU30	SYIC	0,913787027	1,110496351	1,064717492	0,91099913	1,072238671	1,00816796	0,141842516	0,876927646	0,862547477	0,792344854	0,322425
Q80WG5	LRC8A	0,998260228	1,170135575	0,78086114	1,050743057	1,110992034	1,023782067	1,053839462	1,145435042	1,066482304	1,080106182	0,324283
Q5PR73	DIRA2	1,070216495	0,944849148	0,981489358	1,003444999	1,26308664	0,99650457	1,035400648	1,105541443	0,949687901	1,070044241	0,324418
Q80Y17	L2GL1	0,880922982	1,188510274	0,93304657	0,997520173	1,088940167	0,98961286	1,248093594	1,038829049	1,045280777	1,082151289	0,324713
Q7TMS5	ABCG2	1,64829191	0,062963689	1,081521929	1,207222472	1,6282356	1,321943089	1,272304071	1,180640917	1,225338454	1,325692426	0,325087
Q60870	REEP5	1,11251552	1,045765035	0,736728932	1,104990514	1,177759841	1,559380021	1,273065809	0,962408886	0,866854	1,167893712	0,325556
P84309	ADCY5	1,020423204	0,926937105	1,268631657	0,784008034	1,20585632	0,917954527	0,828709879	1,424957454	1,668520263	1,209199689	0,326492
Q9CQ75	NDUA2	0,888460323	1,026531852	1,176922269	0,908085556	0,801123819	0,95597232	1,052386708	0,874982296	0,91961419	0,920815867	0,327085
Q7TMF3	NDUAC	0,926306864	0,95274476	1,079554192	1,041394185	0,940960317	1,017969719	1,199882699	1,121442649	1,033490502	1,062749177	0,328997
P14869	RLA0	0,908550415	1,072145937	0,976586663	1,042716985	1,049612841	1,007441577	0,895609361	0,923042071	0,848833437	0,944907857	0,32929
Q8BRT1	CLAP2	1,063100498	1,052290433	0,907443024	0,977166045	0,957113066	0,959177174	0,993242599	0,927716596	0,982530119	0,963955911	0,329338
O70318	E41L2	0,940048929	1,095261547	0,997767955	0,966921569	0,919179602	1,147196636	1,062238693	1,047785527	1,097728581	1,054825808	0,329853
Q8C0M9	ASGL1	0,95984023	0,998421055	1,028522408	1,013216307	0,917429241	1,06179368	1,028702074	0,901036501	0,82331902	0,946456103	0,330209
Q9D3A9	TTYH1	0,935330709	1,067401857	1,020612296	0,976655138	1,005820513	0,818211235	0,945907369	0,904570531	1,052104577	0,945322845	0,330708
P08003	PDIA4	1,050229287	0,984471092	0,888655223	1,076644398	0,295576986	0,968007519	1,001328743	0,802885869	1,077301066	0,829020037	0,331032
Q8BFZ9	ERLN2	0,971333315	1,031599266	0,989625785	1,007441634	0,937805723	0,957140502	1,008073718	0,91547767	1,036558961	0,971011315	0,331924
Q60972	RBBP4	0,895467173	1,009997709	1,042572843	1,051962275	0,892658655	0,029464748	1,028042484	0,974624572	0,954400154	0,775838123	0,332483
Q9D1E6	TBCB	0,90507116	0,987698208	0,978075446	1,129155186	0,87518712	0,612946069	0,948673096	1,002130626	1,057573748	0,899302131	0,334666
Q99KE1	MAOM	1,030762176	1,02675676	1,037449007	0,905032057	1,171081553	0,952508338	0,930563324	1,168607481	1,117846091	1,068121358	0,335619
P84244	H33	0,804171603	1,105520628	0,96154258	1,12876519	0,747710734	0,91294217	1,123048216	0,895247817	0,820094993	0,899808786	0,337084
Q6NXK7	DPP10	0,945449869	1,005095416	1,127975627	0,921479088	1,118508237	1,081285359	0,961060001	1,019234645	1,085297578	1,053077164	0,337089
P02535	K1C10	0,949387896	1,079047421	1,060878603	0,910686081	1,232413833	5,439364002	1,325072103	1,06488153	0,953517687	2,003049831	0,338981
Q80TL0	PPM1E	0,871428476	1,027404878	1,005505506	1,095661139	0,926490639	0,974994652	0,997258426	0,850260061	0,987183996	0,947237555	0,339191
P67984	RL22	0,750542265	1,05740579	1,105361236	1,08669071	0,779942923	0,916090595	0,969099126	1,010847208	0,882273696	0,91165071	0,339359
P26883	FKB1A	0,575317262	0,914352259	1,512836365	0,997494114	0,623882485	0,682879349	0,938900974	0,81659608	0,97802427	0,808056632	0,339839
Q91VR2	ATPG	1,033638122	0,940444344	0,973915737	1,052001797	0,963320703	1,009522364	1,023924021	0,932006999	0,879366901	0,961628198	0,340346
Q8K406	LGI3	0,980396787	1,245353354	0,850514909	0,92373495	1,194891416	1,143008546	1,054700952	1,074343762	0,976035651	1,088596065	0,34104
P10649	GSTM1	0,930497178	1,026667421	0,978355785	1,064479617	0,960236639	1,265295442	1,167735509	1,041334283	0,944724747	1,075865324	0,342198
P61255	RL26	1,118148155	0,653562348	0,942861166	1,285428331	0,980789196	0,845622016	1,035165585	0,809262997	0,562259521	0,846619863	0,343455
Q9CZ30	OLA1	0,891457182	1,07081057	0,947098633	1,090633615	0,977253553	0,785841192	0,906694205	1,035453023	0,969413178	0,93493103	0,343859
Q8BKC5	IPO5	0,953201231	1,014341679	1,027791608	1,004665482	0,929917331	0,931907966	0,959550703	0,960029164	1,061867081	0,968654449	0,344673
O08586	PTEN	0,212763912	1,2016273	1,255783129	1,32982566	1,366260119	1,334477462	1,151113653	1,293115906	1,07187672	1,243368772	0,34493

Q922S4	PDE2A	0,956110925	1,091546189	1,127683092	0,824659794	0,929113106	1,055455234	1,029136463	1,155559989	1,31730914	1,097314786	0,345226
Q6WVG3	KCD12	0,935545787	0,976697987	1,013277737	1,074478489	0,909422654	0,82574017	0,948342483	0,926642339	1,101681959	0,942365921	0,346657
O55131	SEPT7	1,10835666	0,969216263	0,971940955	0,950486121	1,09565906	0,964032621	1,062264447	1,089934573	1,005332823	1,043444705	0,347273
Q00612	G6PD1	1,02544096	1,066294349	0,924424421	0,98384027	0,957399909	0,932597231	0,970562128	1,056770482	0,851641864	0,953794323	0,347793
Q8R0A7	K0513	0,864530731	1,02321454	1,265064388	0,847190341	0,822714673	0,941915075	0,961941571	0,891047401	0,931994507	0,909922645	0,34785
Q9D394	RUFY3	1,045990656	1,040964959	0,977060719	0,935983666	1,0849038	1,017890682	1,05552378	1,025801121	0,973833948	1,031590666	0,348656
Q6P542	ABCF1	1,094878546	0,958036464	0,939498332	1,007586658	1,047417307	0,91208121	0,878719405	1,0026008	0,9250302	0,953169785	0,348727
Q64433	CH10	0,787766933	1,101434856	1,124903903	0,985894307	0,763098299	0,957499755	0,962856062	1,001309601	0,900966661	0,917146075	0,348793
Q91VM5	RMXL1	0,902732506	0,95691525	1,093657078	1,046695166	0,964667566	0,885635722	0,988102333	0,912552766	1,015396119	0,953270901	0,348864
P32921	SYWC	0,966632414	1,117929327	0,970303492	0,945134768	1,022009538	1,078196312	1,047798744	0,985090814	1,065514733	1,039722028	0,349489
Q99MK8	ARBK1	1,032880987	1,016198071	1,09414146	0,856779482	1,083102839	1,030059529	0,877928336	0,627680453	0,878913483	0,899536928	0,349535
Q99KV1	DJB11	0,935006557	0,983224948	1,011339011	1,070429485	1,030019471	1,026649908	0,921377669	0,893922462	0,922776033	0,958949109	0,350303
P20029	GRP78	0,959935398	1,059753191	1,011791308	0,968520103	1,031076225	1,023721226	1,024303973	0,967618552	1,151454111	1,039634817	0,351727
Q8CCK0	H2AW	0,998783638	1,061050727	0,897992921	1,042172714	1,034491743	0,918799903	0,931905418	0,969683466	0,948967644	0,960769635	0,352687
P35979	RL12	0,857684549	1,009082382	1,058902894	1,074330175	0,959912395	1,151325735	1,093824348	0,984036705	1,113233422	1,060466521	0,352834
Q9JM63	KCJ10	1,019767635	1,112549188	0,860247339	1,007435838	1,11596525	1,667279359	0,966316603	1,007712199	1,013433356	1,154141353	0,35325
Q78ZA7	NP1L4	0,97329316	0,99019629	0,920212171	1,116298378	0,938885773	0,956908835	1,300286327	1,120090871	1,093901502	1,082014662	0,353699
P63005	LIS1	1,044226388	0,988028161	1,016720201	0,95102525	1,044866987	0,918483465	0,959076511	0,971458781	0,96153488	0,971084125	0,354354
Q6ZPE2	MTMR5	1,33987275	0,851166166	0,859540305	0,949420779	1,002523543	0,493647695	0,921291535	0,975828375	0,882798856	0,855218001	0,354721
E9Q6P5	TTC7B	0,966913243	1,013785337	0,921436197	1,097865223	0,984822363	0,866044317	0,844113364	1,053437995	0,977395256	0,945162659	0,355688
Q9R1P4	PSA1	1,026904629	1,007906621	0,939470233	1,025718518	1,018378883	0,991765969	1,012497166	0,845554063	0,928193091	0,959277834	0,355772
P62823	RAB3C	1,793473954	0,494618986	1,036552067	0,675354993	0,628078375	0,616083034	0,691586499	1,009777822	0,750322869	0,73916972	0,358282
P62270	RS18	0,837355187	1,030313729	1,101769598	1,030561486	0,851517717	0,936573294	1,067790388	0,930940759	0,896730155	0,936710462	0,359236
O54916	REPS1	0,890078019	0,921390124	1,101381853	1,087150004	1,015965893	1,113629274	1,036049898	0,94982862	1,27166202	1,077427141	0,359372
Q7TNG5	EMAL2	1,062324487	1,115845319	0,906828452	0,915001742	1,046256664	1,050067307	0,853075174	0,848067589	0,850851644	0,929663676	0,359471
P63085	MK01	0,744539719	0,994925628	1,23164875	1,028885903	0,969496879	0,881787825	0,870842289	0,861976142	0,966044864	0,9100296	0,359715
Q8BUV3	GEPH	0,991479253	1,029683581	0,977071363	1,001765802	1,063423715	0,952982985	1,055100652	1,046029591	1,005567003	1,024620789	0,360932
Q9CQV8	1433B	1,116882925	0,887954536	0,88694713	1,108215409	0,968664388	1,057796089	0,992392636	0,84586616	0,643489287	0,901641712	0,360994
Q9QYA2	TOM40	0,986077912	1,055423787	1,049848844	0,908649456	1,040952435	0,915292374	1,052203524	0,914151972	0,429964782	0,870513018	0,361864
Q9CPP6	NDUA5	0,792722388	1,059446288	1,00896683	1,138864494	0,81759449	0,955329967	0,970245099	0,930929347	0,972320106	0,929283802	0,364009
P47911	RL6	0,903828402	1,039033424	1,004861006	1,052277168	1,018108803	0,928351164	0,958654511	0,918964131	0,997887821	0,964393286	0,364053
Q9WU78	PDC6I	0,966488995	1,069433629	0,947044208	1,017033168	1,02318458	0,920516724	0,964920702	0,94865428	0,990866462	0,969628549	0,36409
Q9EQH3	VPS35	0,930510694	1,022407916	1,01894032	1,028141071	0,965384446	0,880253047	0,974604964	0,924571134	1,061874511	0,96133762	0,364319
Q9R0P5	DEST	0,915731763	1,073508741	0,972316701	1,038442794	0,943203449	1,032148118	1,241622961	1,1359689	0,98118886	1,066826458	0,364579
Q9EPR5	SORC2	0,760592698	1,086120483	1,055730577	1,097556243	0,548405008	0,99972587	0,03114328	1,14909976	1,069810243	0,759636832	0,365078
Q9Z0E0	NCDN	0,847302885	1,05835053	1,051956463	1,042390122	0,952463466	0,851033951	0,953544888	0,890848409	1,060569409	0,941692025	0,365169
Q8VEJ9	VPS4A	0,998241794	1,008440811	1,033874087	0,959443308	1,007384359	0,905810203	0,959420329	1,062320958	0,854615319	0,957910234	0,366766
Q9CQI3	GMFB	0,947624117	1,106442065	1,080644516	0,865289303	0,914672123	0,971075834	1,075575649	0,884689747	0,813833797	0,93196943	0,366853
Q8C0E2	VP26B	0,998871633	1,030791053	0,953373607	1,016963708	0,801782876	0,739176773	1,07418944	0,933506634	1,071531662	0,924037477	0,36793
Q61361	PGCB	0,989640077	0,864037724	1,123562899	1,0227593	0,969180158	0,954599171	0,92751926	0,927327458	0,98710058	0,953145325	0,369392

P35980	RL18	0,572174747	1,028394153	1,211335617	1,188095483	0,242653497	0,771978921	1,025939421	0,990025785	0,961080341	0,798335593	0,369674
Q9JLZ3	AUHM	1,102626484	0,948849811	0,870329728	1,078193977	0,987420438	1,034358517	0,978748075	0,887842834	0,763887419	0,930451457	0,370046
P35803	GPM6B	1,054071797	0,927336401	1,091543616	0,927048186	0,982803411	1,067050012	1,068962244	0,963365435	1,230058713	1,062447963	0,370399
Q9JME5	AP3B2	0,84849642	1,087332667	1,022433551	1,041737362	1,17687696	1,056049866	0,977089112	1,007893865	1,067978041	1,057177569	0,37281
P28271	ACOC	0,977124153	1,020169422	0,971287682	1,031418743	0,941795599	1,166749612	0,998381731	0,996419252	1,144084885	1,049486216	0,375118
P60202	MYPR	1,122561046	1,082958919	0,772416502	1,022063533	1,142021903	1,256150202	1,288982922	1,016952281	0,843442293	1,10950992	0,375499
Q3V3R1	C1TM	0,887997078	1,029939945	1,137016547	0,94504643	0,037769126	0,920456563	0,969869057	0,959869545	1,068583877	0,791309634	0,375618
Q8K3H0	DP13A	0,906087944	0,994288711	1,007361004	1,092262342	0,993351517	0,823396209	0,958624418	0,956423764	1,025532085	0,951465599	0,376699
Q8R5M8	CADM1	0,885117824	0,958734785	1,159415979	0,996731411	0,887505886	0,793008413	0,870633182	0,850566995	1,171129951	0,914568886	0,377102
Q01065	PDE1B	1,095944379	0,929710409	1,102280332	0,87206488	1,096791669	0,826330641	0,977304597	1,280722298	1,452677225	1,126765286	0,379699
Q8BY89	CTL2	0,811968585	1,24964806	1,165139278	0,773244077	1,010146694	0,843728538	1,253224224	1,331148818	1,259433928	1,13953644	0,379729
P62881	GBB5	1,018328723	0,952389119	1,021327271	1,007954887	1,09216105	1,009628676	1,022082938	1,085417702	0,947158444	1,031289762	0,38049
Q9R1Q9	VAS1	0,986467979	0,92783582	1,061512247	1,024183954	0,843364239	0,831837685	1,01737439	1,095997823	0,916944116	0,941103651	0,380631
Q9DBL7	COASY	1,235785704	1,371938162	1,148708898	0,243567237	1,349298132	1,188857773	1,137067952	1,228259359	1,167558563	1,214208356	0,380635
Q62167	DDX3X	0,868986036	0,998673966	1,019772957	1,112567041	0,943525567	0,917138544	1,008881218	0,891102532	1,000379064	0,952205385	0,381762
Q9CR95	NECP1	0,877690975	1,102770001	1,011256664	1,00828236	1,040982431	0,933798987	0,913708158	0,896514748	0,980761063	0,953153077	0,382523
Q9CY64	BIEA	1,125599715	0,893853354	0,839202282	1,141344649	1,069684892	0,97839606	1,16720802	1,192026358	0,987550403	1,078973147	0,382987
Q01768	NDKB	0,900235741	1,016410806	1,014081387	1,069272067	0,87446418	0,961327745	1,104557661	0,918628947	0,872581147	0,946311936	0,383365
Q99KB8	GLO2	0,999582725	0,897343481	1,00372303	1,099350764	0,974477777	1,104460033	0,979722864	0,825855668	0,73728728	0,924360724	0,383836
Q8BTX9	HSDL1	0,864972256	1,051290082	0,96969295	1,114044712	0,965624313	0,944698263	1,024116212	0,98980231	0,703853985	0,925619017	0,383883
Q91Z61	DIRA1	1,035623022	1,01011437	0,932122595	1,022140014	1,071000326	1,180445636	1,06004876	1,055102365	0,892925155	1,051904448	0,384494
P70290	EM55	1,02287172	0,982811947	1,031214285	0,963102048	1,119073967	1,035932169	0,988206328	1,003076876	0,996111082	1,028480084	0,385528
Q9D051	ODPB	1,02519841	0,995024268	0,966278483	1,01349884	1,030620784	0,997171092	1,047350596	1,056946595	0,968528911	1,020123596	0,386142
P26638	SYSC	0,990305382	1,045386783	1,0006346	0,963673235	1,064718816	0,828993788	0,996530669	0,949852797	0,946925807	0,957404375	0,387041
Q640R3	HECAM	1,069336935	1,068929083	0,876617931	0,985116051	0,975818987	1,307205935	1,093063535	1,016598834	0,97879255	1,074295968	0,388846
P28658	ATX10	0,817553746	1,033276678	0,941797661	1,207371915	0,858952145	0,967356191	1,034688224	0,906313649	0,857326155	0,924927273	0,388873
P27601	GNA13	1,015141936	0,967246197	1,030308519	0,987303348	0,970243639	0,945715251	0,987935101	1,045461305	0,93369599	0,976610257	0,388907
Q9ESJ4	SPN90	0,895831618	1,062332634	0,955840898	1,085994851	1,149384459	0,847168085	1,029723132	1,171798751	1,173865763	1,074388038	0,390406
P68254	1433T	1,219371208	0,754225804	1,092856365	0,933546623	0,942343805	1,063533293	0,881029793	0,872408526	0,765794388	0,905021961	0,392623
Q5SSL4	ABR	0,845326657	1,068181877	1,005894545	1,080596922	0,917087885	0,923763114	1,000783952	0,978192554	0,948751189	0,953715739	0,392744
P62874	GBB1	0,73741826	0,899405065	1,20084507	1,162331605	0,763369709	1,126297592	0,847206982	0,805233445	0,908237047	0,890068955	0,393048
A2ALS5	RPGP1	0,968115142	0,99497337	1,016098926	1,020812562	1,013985948	1,083301446	0,364325492	0,866722529	0,999538068	0,865574697	0,393685
Q811D0	DLG1	0,978253762	1,020378366	1,005755243	0,995612629	1,028708017	0,985356062	0,983547833	0,891153867	0,989598248	0,975672805	0,39567
O88741	GDAP1	1,102405274	1,009018509	0,790468376	1,098107841	1,124974839	1,095175979	1,01021166	1,063724239	1,017507205	1,062318784	0,395746
Q9WUB3	PYGM	1,019723178	1,084937923	0,876337725	1,019001173	1,091348919	1,197243856	1,101732371	0,97240004	0,936000835	1,059745204	0,395782
P63087	PP1G	0,560178252	1,181637009	1,158374274	1,099810465	1,282381544	1,042834516	1,109976396	1,219285165	0,993008227	1,12949717	0,396202
O35382	EXOC4	0,901879622	1,00291381	1,094082546	1,001124022	1,11585368	0,735146672	0,906139991	0,888261911	0,996545235	0,928389498	0,396299
P63325	RS10	0,950218759	0,872330505	0,974600876	1,20284986	0,863283612	0,538194853	1,006356147	1,099072635	0,927996434	0,886980736	0,396556
Q8BHK1	NIPA1	1,15920576	1,129362187	0,753683519	0,957748534	0,921095238	1,138983106	1,011854428	0,725725237	0,55960986	0,871453574	0,397624
P84091	AP2M1	0,964289482	0,982766212	1,055865118	0,997079188	1,070819971	0,920134861	1,056272214	1,055764	1,061198688	1,032837947	0,398204

Q8BIZ1	ANS1B	0,804562676	0,821179486	1,190717658	1,183540179	1,008063143	0,597126937	0,8452651	0,903694379	1,056169331	0,882063778	0,399956
Q9JIA1	LGI1	0,980658489	0,991457238	1,018275486	1,009608787	0,914300633	0,925608543	0,96965913	0,994322232	1,059372094	0,972652526	0,400001
P08553	NFM	1,227034805	1,121318739	0,714927363	0,936719093	1,202836428	1,34878257	1,121775546	1,057795277	0,867554285	1,119748821	0,400406
Q9CPX6	ATG3	0,887921289	1,09332558	0,868418454	1,150334677	0,842233059	0,914358665	0,869111027	1,091182276	0,928415983	0,929060202	0,402991
Q8BHN3	GANAB	1,000648088	0,965662898	0,997649836	1,036039177	1,055684705	0,923503576	0,979344918	0,939408951	0,973957243	0,974379879	0,403707
P14873	MAP1B	0,963847285	1,04625172	1,003508628	0,986392366	1,05673529	1,050921174	1,000601365	0,974139409	1,020849114	1,02064927	0,404444
P31938	MP2K1	0,871970368	0,998982455	1,09003848	1,039008697	0,910225215	0,907674606	0,995729354	0,974830205	1,003027674	0,958297411	0,405721
Q7M6Y3	PICAL	1,2440894	0,202607763	1,275143261	1,278159576	1,190623405	1,233026067	1,334387145	1,260923169	1,042249132	1,212241783	0,405755
Q925N0	SFXN5	1,209034187	1,009403054	0,70932318	1,072239579	0,841794152	1,295975595	0,836678482	0,780053589	0,462948655	0,843490095	0,405814
Q9DCM0	ETHE1	1,277999782	0,094192964	1,393728195	1,23407906	1,182094	1,325823301	1,443344859	1,156064054	1,110554846	1,243576212	0,406483
Q8BWT5	DIP2A	0,889034408	1,115924684	0,976487178	1,01855373	1,374932169	1,018882213	0,897696018	1,250834483	0,950971608	1,098663298	0,407091
O54829	RGS7	0,910597453	1,062681801	1,053650371	0,973070376	1,105464547	0,960475216	0,925005432	0,992483509	0,116274934	0,819940728	0,408565
Q9WTX6	CUL1	0,918899614	1,1193245	0,950600347	1,011175539	0,938532824	0,368129427	1,055683035	0,997523465	0,98778616	0,869530983	0,409763
Q99KI0	ACON	1,064243374	1,035349357	0,943787527	0,956619742	1,063609495	1,074430634	0,991709061	0,97101427	1,052225357	1,030597763	0,409865
Q8CG76	ARK72	0,946140014	0,952685795	1,045056636	1,056117555	1,014081115	1,250911176	1,080455928	0,944679889	0,9963949	1,057304601	0,410196
Q99K85	SERC	1,043591767	0,917086885	0,993462016	1,045859332	1,039629607	0,84570693	1,028565391	0,995192267	0,84521272	0,950861383	0,410426
P00405	COX2	1,214260202	0,893105274	0,94486939	0,947765134	1,059197925	0,972258294	1,05925291	0,79399868	0,587801217	0,894501805	0,412011
Q91VD9	NDUS1	1,079637467	1,010691972	0,971986718	0,937683844	0,960011042	0,997641287	0,956387789	0,954887438	1,003215381	0,974428588	0,412616
O35668	HAP1	0,44163701	1,285691161	1,034355817	1,238316012	0,904984472	1,052436018	1,370161271	1,292211089	1,230417553	1,170042081	0,413074
Q8CHC4	SYNJ1	0,931014479	1,079101805	0,977376868	1,012506848	1,060019758	0,912772133	0,903126283	0,965599611	0,977656316	0,96383482	0,417574
Q9DCL9	PUR6	1,075132686	1,050111759	0,923057454	0,951698101	1,000395529	0,919641882	0,979723645	1,028480862	0,878588844	0,961366152	0,418087
Q99LF4	RTCB	0,970154934	0,984756545	0,987895285	1,057193236	0,97531712	0,956915101	1,051867961	1,087229875	1,078842504	1,030034512	0,418717
Q9QZX7	SRR	0,927461597	0,960760365	1,120351624	0,991426413	0,953904356	0,817888997	0,926780184	0,057304319	1,257069051	0,802589381	0,418877
P62812	GBRA1	0,973955742	1,005896508	0,806036282	1,214111469	1,206683346	1,283347926	1,042042904	1,130833603	0,830709495	1,098723455	0,419352
O35609	SCAM3	0,885180716	1,147453286	0,992718134	0,974647864	0,950707796	0,939139697	0,952086889	0,867225599	1,044188815	0,950669759	0,41949
Q3TEA8	HP1B3	0,856950533	1,092042515	1,032351675	1,018655277	0,782673604	1,007468302	1,110697122	0,684737352	0,98903812	0,9149229	0,419652
Q91ZU6	DYST	0,769604111	1,032434691	1,131410475	1,066550723	1,048510576	0,816657053	0,964208817	0,924358518	0,893876824	0,929522357	0,41987
P63328	PP2BA	1,003955611	1,001776484	0,984883177	1,009384728	1,058661218	0,871815783	1,01634002	1,1323052	1,179117708	1,051647986	0,420702
Q9DC07	LNEBL	0,897073878	1,178507933	0,840481044	1,083937144	0,839927134	1,113612917	1,047053163	0,83723473	0,679463476	0,903458284	0,420851
P31648	SC6A1	0,877901096	1,054502743	1,058382123	1,009214038	0,973380627	1,043859259	1,002357139	0,747671513	0,937693468	0,940992401	0,421029
Q91VN4	MIC25	1,030642242	0,960878082	1,02942071	0,979058967	0,955146539	0,718387435	1,02813054	1,023963049	0,988232506	0,942772014	0,422523
Q8R570	SNP47	1,011776903	1,107209426	0,920501744	0,960511928	1,130026985	0,978273959	1,056836405	0,994156769	1,0389771	1,039654244	0,42316
P12815	PDCD6	1,106524636	1,196171438	1,566418226	0,1308857	1,009038107	1,24769978	1,275411804	1,284191828	1,36167748	1,2356038	0,423851
P63040	CPLX1	0,829106302	0,898420676	1,082028761	1,19044426	0,904810358	1,435634374	1,222285953	1,119531075	0,896169349	1,115686222	0,424036
P46061	RAGP1	1,07355212	0,996501897	0,961208672	0,968737311	1,040416417	1,300914696	0,855911297	1,068196302	1,088567469	1,070801236	0,424163
P20357	MTAP2	0,794966049	0,993932938	1,199883511	1,011217502	1,028905188	0,848329134	0,919650427	0,858823811	0,991804076	0,929502527	0,424669
Q80TT2	BAIP3	0,905235562	1,057072193	0,996560897	1,041131348	0,852527457	1,056426615	0,948696186	0,87509422	1,033764842	0,953301864	0,425277
Q8BPN8	DMXL2	0,965266686	0,923402503	1,075282132	1,036048679	1,077190399	0,836183612	0,714936297	0,979400929	1,043742802	0,930290808	0,426612
Q99JY8	PLPP3	0,946659267	1,012026863	1,068015185	0,973298685	0,957374129	1,146977488	0,870865063	0,878254284	0,875887291	0,945871651	0,426637
Q9JIF0	ANM1	0,617526514	1,174066388	0,896687396	1,311719702	0,085532351	0,685920645	1,043474896	1,239809908	0,84611481	0,780170522	0,427712

Q9CWZ7	SNAG	0,94839324	1,002510429	0,994083101	1,05501323	1,019339682	0,949924728	1,034942126	0,936058488	0,925884798	0,973229965	0,429123
Q62189	SNRPA	1,172023823	0,878344748	0,980962712	0,968668717	1,143794366	1,02925353	0,910742376	0,747356198	0,722541972	0,910737688	0,429354
P57759	ERP29	0,9330338	0,980440952	1,050366501	1,036158747	0,955233103	0,83228708	1,054429882	0,989447449	0,970179047	0,960315312	0,430005
P61982	1433G	0,980444395	1,035656917	0,991379962	0,992518727	0,915949618	1,069389834	1,015131791	0,939391439	0,902502786	0,968473094	0,430557
P28661	SEPT4	1,468807623	1,234016688	1,161479108	0,135696581	1,565265249	1,063653734	1,225271915	1,029242264	1,291879131	1,235062458	0,431108
Q9DCD0	6PGD	0,935229995	1,029563658	1,000532516	1,034673831	1,005299217	0,88491069	1,121725448	1,202313845	1,051715088	1,053192858	0,433688
P56395	CYB5	0,058671264	1,247743636	1,339532573	1,354052526	0,922286788	1,455322557	1,395943166	1,117203281	1,345486618	1,247248482	0,434771
O70325	GPX41	1,06842681	1,01067571	0,905943082	1,014954398	0,919786346	0,980448876	1,122715235	0,929173643	0,723258792	0,935076578	0,436431
Q9Z0H8	CLIP2	0,989693156	0,915945263	1,084050597	1,010310984	1,029384705	1,091686878	1,038123097	0,198703998	0,859102795	0,843400295	0,438097
Q9JKR6	HYOU1	0,856132398	0,974256444	0,993319692	1,176291466	0,941144735	0,677668308	0,951658454	0,983640234	1,061549026	0,923132151	0,439244
Q9Z2D6	MECP2	1,049617657	1,087836555	1,003652548	0,858893241	1,036405693	1,079165121	1,147718004	0,992428095	0,975421966	1,046227776	0,439475
Q9QXY6	EHD3	0,684917622	1,193345533	1,11323033	1,008506515	1,179618911	0,940509227	1,09062173	1,103393969	1,12686797	1,088202361	0,441028
Q80UG2	PLXA4	0,604943019	1,121198357	1,217661386	1,056197238	1,004074967	0,894732589	1,044818966	1,009249542	0,024378557	0,795450924	0,441303
P54775	PRS6B	0,967697023	0,964779498	1,075999807	0,991523672	0,951013854	0,850345645	1,05875245	0,95054657	1,004843278	0,963100359	0,442965
Q923T9	KCC2G	1,14640322	1,000124388	0,906555981	0,946916411	0,963611681	0,996288234	0,990629227	0,962894293	0,873857143	0,957456116	0,443249
Q6R891	NEB2	1,030415724	0,961750932	1,065770979	0,942062365	0,975669451	0,861012428	0,985957271	0,8688708	1,088664616	0,956034913	0,443364
P10922	H10	0,80210862	1,106460485	0,967803592	1,123627303	0,83124242	1,007889024	1,197261654	1,30609456	1,117253623	1,091948256	0,443498
Q9EPN1	NBEA	0,968917742	1,565154203	1,412469943	0,053458112	1,544156169	0,456308112	1,389036757	1,350459062	1,887322141	1,325456448	0,444489
P62897	CYC	0,877324552	1,044403787	1,036806913	1,041464747	0,975168169	1,067007498	1,111758897	1,032112417	0,996853047	1,036580006	0,446766
G5E8K5	ANK3	1,080361005	1,00092867	0,952921028	0,965789297	1,086010343	1,01765282	1,011722933	0,966727966	1,055153043	1,027453421	0,447064
P61294	RAB6B	0,968753323	0,947263037	1,111306475	0,972677165	0,983799339	1,298581564	1,278642197	1,03388706	0,832924838	1,085567	0,448095
Q9Z2Q6	SEPT5	1,081727385	0,922204093	1,028102556	0,967965966	1,076057261	0,917474826	0,959952338	0,934554574	0,9326217	0,96413214	0,449082
P62331	ARF6	0,844372385	1,0489848	1,139349094	0,967293721	0,951088654	0,688803445	1,070303665	1,03281183	0,879602478	0,924522014	0,449856
Q8BL97	SRSF7	0,824945193	1,017650167	1,065073351	1,09233129	0,931735996	0,902305291	0,967050902	0,919245654	1,041162685	0,952300106	0,45212
Q9JL62	GLTP	1,096174902	1,223768594	0,78630709	0,893749415	0,988091916	0,994170369	1,019574902	0,883296419	0,657012433	0,908429208	0,452501
Q8C163	EXOG	0,966824945	0,921513786	1,024870701	1,086790567	1,024765884	0,995470377	1,036491547	1,284597108	0,950438758	1,058352735	0,452717
P21460	CYTC	0,940794866	0,831942398	1,171145391	1,056117345	1,005704539	1,280606776	1,092306703	0,990385851	0,988455391	1,071491852	0,453335
Q9CQW1	YKT6	1,035237679	0,941505612	1,02880791	0,994448799	1,071222015	1,040357818	0,992294285	0,697468583	0,884729128	0,937214366	0,453534
Q62446	FKBP3	1,070841145	1,022091591	0,941995047	0,965072216	1,296148686	0,99662267	1,183276874	1,041140968	0,84543711	1,072525262	0,454754
P62806	H4	0,825339081	1,094331158	1,060021839	1,020307923	0,845268276	0,918764757	1,065518357	0,946270323	0,96202261	0,947568865	0,456454
P60122	RUVB1	0,924766321	1,022554896	1,075945847	0,976732936	0,827161343	0,839015948	1,090511374	0,928338478	1,050456591	0,947096747	0,456651
Q9Z1Z0	USO1	0,861445524	1,139879894	1,003933838	0,994740744	1,113004481	1,053313238	0,918500147	1,124002827	1,04680067	1,051124272	0,456821
Q99K46	UBP11	0,834483564	1,03609453	1,160102333	0,969319573	0,98654608	1,020450914	0,957496734	1,115729177	1,257417446	1,06752807	0,457112
Q80T41	GABR2	1,074743443	1,016743233	0,989628279	0,918885044	1,105578668	1,097695869	0,876712137	0,780454625	0,805866479	0,933261555	0,45748
P97390	VPS45	0,896178326	1,157858739	0,936143434	1,009819501	0,96576432	0,951229613	0,813418904	0,871244474	1,103137567	0,940958975	0,458261
Q9CZ42	NNRD	0,67177006	1,002624165	1,196044421	1,129561353	1,126895725	1,016433335	1,07899754	1,094019979	1,093238172	1,08191695	0,458401
P61750	ARF4	0,986683005	1,193513372	1,260130038	0,559673586	0,92368901	1,173543869	1,295588978	1,082934289	1,130847436	1,121320716	0,458975
Q8VHH5	AGAP3	0,932697698	0,970738051	1,057268634	1,039295618	1,041233202	0,912041792	0,972656519	0,99757227	0,933627048	0,971426166	0,459371
P63001	RAC1	0,87466205	0,842465179	1,204856787	1,078015984	0,856357179	1,239583411	1,064970976	1,021788732	1,259738785	1,088487817	0,460325
P53986	MOT1	1,067869833	1,071140187	0,921376348	0,939613633	1,097616108	1,154825284	1,108990458	0,883631954	1,008052103	1,050623181	0,461417

Q8BJY1	PSMD5	0,916317407	1,083817718	1,016492018	0,983372857	0,960142848	0,972527442	1,042664516	1,021257313	0,728390024	0,944996429	0,462287
P47708	RP3A	1,113039713	1,016989567	0,921976637	0,947994082	1,267803596	1,140867544	1,018098263	0,924738647	0,961183583	1,062538327	0,463864
Q61024	ASNS	1,055077753	0,700472994	1,063686685	1,180762568	1,04235265	0,865308862	1,203681077	1,218431911	1,12259528	1,090473956	0,464438
Q925I1	ATAD3	1,303283101	1,41518535	1,217361793	0,064169756	1,170135391	1,255366171	1,270945325	1,109383708	1,27119688	1,215405495	0,464915
Q9Z0P5	TWF2	0,937586033	1,047897631	1,000107139	1,014409197	0,146620306	1,118570396	0,953068396	0,959093397	1,045287229	0,844527945	0,466353
Q63844	MK03	1,074124634	1,216374536	0,54671285	1,16278798	1,181573844	1,001498293	1,105174058	1,150986096	1,097313976	1,107309253	0,467112
P97823	LYPA1	1,04692189	1,135292015	0,970784022	0,847002072	0,941426784	1,127639826	0,998366945	0,785040723	0,801827261	0,930860308	0,467598
B9EKR1	PRPTZ	0,916073434	1,032333186	1,011399604	1,040193776	1,03890943	1,004153726	0,982336683	0,884123123	0,939193382	0,969743269	0,468319
P61358	RL27	0,703322263	0,969226966	1,272269013	1,055181757	0,745092723	0,758467704	1,201420491	0,813583836	0,940861161	0,891885183	0,468677
Q9CZ44	NSF1C	0,930920462	1,015290716	1,018184079	1,035604743	1,053875439	0,975612427	0,931516545	0,948700373	0,969694299	0,975879817	0,468837
Q64105	SPRE	0,945903551	1,047848611	0,993011322	1,013236516	1,031957637	1,090633993	1,03435564	1,004829863	0,956627113	1,023680849	0,469999
Q8BFZ3	ACTBL	0,921184469	0,94523602	0,991588805	1,141990706	0,895111004	0,811896186	0,977819566	3,002088778	1,104196233	1,358222353	0,471947
Q99MN9	PCCB	0,05372232	1,163445312	1,437953651	1,344878717	1,266440368	1,131089104	1,264681411	1,202335278	1,206181201	1,214145472	0,473832
Q8CAA7	PGM2L	0,907616154	1,066745264	1,024094702	1,00154388	1,060786931	0,935288656	1,00535658	0,90631979	0,925699004	0,966690192	0,474558
Q8CGC7	SYEP	0,915219391	1,067769552	0,960014814	1,056996243	0,923306387	0,98549422	0,845302591	0,99452319	1,055206734	0,960766624	0,47502
Q8BNW9	KBTBB	1,070763524	1,074156813	0,764988482	1,090091181	0,735030061	0,769511272	0,898890635	0,891399047	1,241802963	0,907326796	0,475208
Q60668	HNRPD	0,90713617	0,942538978	0,957519652	1,1928052	0,993241246	0,836408516	1,14705394	1,208101396	1,184024093	1,073765838	0,476033
B2RSH2	GNAI1	0,947042808	0,980596726	1,083612811	0,988747654	1,06744941	0,983705362	1,044912285	1,027424971	0,993565864	1,023411578	0,478068
Q8CAQ8	MIC60	1,011172817	1,021885165	1,027395788	0,93954623	0,970479941	1,041124442	1,032774193	0,976671245	1,099763974	1,024162759	0,478721
Q9R111	GUAD	0,88479197	0,871841566	1,280358247	0,963008218	0,844026444	0,754382732	0,925285865	0,987962475	1,091413792	0,920614262	0,480852
Q9JKD3	SCAM5	1,206384353	0,813714634	0,983157251	0,996743762	0,707544131	1,201958072	0,749896516	0,646017788	1,141367158	0,889356733	0,485054
O88447	KLC1	1,007393727	1,017137083	0,898070015	1,077399175	1,033105078	0,94391564	0,988769563	0,980459191	0,90191015	0,969631924	0,485702
P33173	KIF1A	0,85959774	1,09399111	1,064009989	0,982401161	1,092899129	0,973980958	0,950515448	0,883575647	0,857918701	0,951777976	0,485811
P47915	RL29	0,992761123	1,061697117	0,842635023	1,102906737	1,139336062	1,150572728	1,097295314	1,009241016	0,881864677	1,055661959	0,48647
P53810	PIPNA	0,915721609	0,999805043	0,918196018	1,16627733	1,066535514	0,876764556	0,999807047	0,972673218	0,821008693	0,947357806	0,486728
Q8BVQ5	PPME1	0,996951429	0,975962831	1,008158259	1,018927481	1,045967027	0,827362232	0,946751434	1,029077101	0,98604804	0,967041167	0,486857
Q5FWK3	RHG01	1,180004769	0,961140736	0,898112552	0,960741942	1,044928986	0,987496143	1,015629868	1,138149576	1,036309651	1,044502845	0,492478
O08795	GLU2B	0,972086	1,054592163	0,947728167	1,02559367	0,882199698	1,264678695	1,110239827	0,95659139	1,066868028	1,056115528	0,493423
P46660	AINX	1,261884258	1,101375408	0,774394134	0,8623462	1,192534654	1,150319355	1,068489696	1,069563399	0,919938749	1,080169171	0,494121
P20060	HEXB	1,002640499	0,980191351	1,015924272	1,001243877	0,99646824	1,050146858	0,958002254	0,989962518	0,905805916	0,980077157	0,495274
P06837	NEUM	0,977090538	1,145462921	0,896026567	0,981419974	1,00039098	1,0249883	1,058178506	0,980540142	1,133674772	1,03955454	0,4963
P23819	GRIA2	0,981488141	0,326077666	1,335528697	1,356905496	1,091570213	1,007347997	1,11083141	1,18366244	1,414520425	1,161586497	0,496635
P61089	UBE2N	0,888019043	1,069762721	1,023248926	1,01896931	0,94773239	1,032039759	1,026747381	0,900402993	0,929988983	0,967382301	0,497175
Q811I0	ATPF1	1,047879895	0,893574668	0,987220892	1,071324545	1,110564374	0,097289477	1,084246862	1,049806707	0,873355108	0,843052506	0,497455
Q9CR51	VATG1	0,806063293	1,095019842	1,091237272	1,007679593	0,792387101	1,027662289	1,088759094	0,892745563	0,896959067	0,939702623	0,497911
Q60634	FLOT2	0,928857719	0,98998039	0,989714185	1,091447706	1,074554591	0,961307259	0,950610575	1,073438889	1,108686014	1,033719466	0,49805
P30275	KCRU	1,025389726	0,998678166	0,947411661	1,028520447	1,076718589	0,927336788	1,060959259	1,083380118	0,987476504	1,027174252	0,49826
P35700	PRDX1	1,002691725	0,999995511	0,974539666	1,022773099	1,002417008	1,103590608	1,137866413	1,058982398	0,881407866	1,036852858	0,499481
Q9CQA3	SDHB	1,054577474	0,971930929	0,948509918	1,024981679	0,950626843	1,0430852	1,022545195	0,970352263	0,868350968	0,970992094	0,499719
Q91WK2	EIF3H	1,010556998	0,98572426	1,004471217	0,999247525	0,863684268	0,141060517	1,129815233	1,112372238	1,011309233	0,851648298	0,499788

Q9D1A2	CNDP2	0,987741393	1,010816258	1,029603989	0,97183836	1,026060452	0,963713458	1,048043812	1,063603473	0,984258383	1,017135916	0,500772
Q9CT10	RANB3	1,242878397	0,824490665	0,9770795	0,955551437	1,105758946	0,566969089	1,038087538	0,966155477	0,85499842	0,906393894	0,50099
Q9CRB6	TPPP3	0,926633767	1,128021008	0,920937224	1,024408	1,083112711	1,129528392	1,061987187	1,099001706	0,867371524	1,048200304	0,501328
Q6PDM2	SRSF1	0,926141726	1,02433385	1,01836552	1,031158904	0,986472489	0,945492197	1,077153662	0,957911271	0,885178603	0,970441645	0,501343
Q8CGK3	LONM	0,945346177	1,050730702	1,011151306	0,992771816	0,9959316	0,876193696	0,977423086	0,996313248	1,029192919	0,97501091	0,501368
P61979	HNRPK	0,87051611	1,045180002	1,045739648	1,03856424	0,96841967	0,852989936	1,029211029	0,968149959	1,001001949	0,963954508	0,502079
P14231	AT1B2	1,06931812	0,976875954	0,935021082	1,018784844	0,891368501	1,005412835	1,036686715	1,027077906	0,873200816	0,966749355	0,502219
P53994	RAB2A	0,866673413	1,020439964	1,130501908	0,982384715	0,84400211	0,941510362	0,964553739	1,071007269	0,957464756	0,955707647	0,504224
Q8BHZ0	FA49A	0,884403327	0,888373885	1,218572108	1,00865068	1,038843129	0,885158788	0,772061427	0,977699608	1,01528663	0,937809916	0,504727
Q64010	CRK	0,096462626	1,883284544	1,763835485	0,256417345	1,82130204	1,463180546	1,591861252	1,958385115	0,129031528	1,392752096	0,505725
Q8CBY8	DCTN4	0,954716592	1,087074543	1,00310781	0,955101055	1,157740762	1,04450874	0,242669424	0,911310458	0,993535703	0,869953017	0,50595
Q8BKX1	BAIP2	0,881695483	0,913692609	1,25587696	0,948734948	1,007566417	0,884304788	1,032888059	1,16685282	1,293673746	1,077057166	0,506478
P31650	S6A11	1,081480138	1,079483249	0,799733059	1,039303554	1,040907646	1,40624423	1,138530488	0,94248082	0,88820733	1,083274103	0,507908
Q9Z1G3	VATC1	1,042237262	1,017586314	0,886220058	1,053956366	1,189561788	0,948138981	1,182501448	1,096827055	0,864766082	1,056359071	0,508193
P80315	TCPD	1,040400593	1,058497909	1,059850399	0,841251099	1,030328824	1,006660295	1,092486719	0,986979616	1,06204469	1,035700029	0,509249
O08917	FLOT1	1,05537345	0,998155281	0,922651906	1,023819363	1,01916876	0,780001768	1,030911733	0,983466735	0,986201171	0,959950033	0,509974
E9Q3L2	PI4KA	1,316302239	0,308302831	1,190659342	1,184735588	1,120644256	1,176041563	1,225397395	1,040606824	1,153832188	1,143304445	0,511491
Q7TN79	AKA7G	0,962312604	1,223434023	1,057438762	0,756814611	0,873433379	1,016100714	1,036969928	0,775944755	0,947572873	0,93000433	0,51216
P56135	ATPK	0,863102591	0,962744466	1,24674651	0,927406433	0,789663204	0,93315848	1,028127435	0,959218468	0,987563207	0,939546159	0,512275
P84096	RHOG	1,103224346	0,960546846	0,937443105	0,998785703	1,01761519	1,083052453	1,226126695	0,999311005	0,907693804	1,046759829	0,51417
Q9D3D9	ATPD	1,005685919	0,991786995	0,987794959	1,014732126	0,827536844	1,07058786	1,14672399	0,855044761	0,840744388	0,948127569	0,516374
P55012	S12A2	1,093378835	1,035959243	0,802403801	1,068258121	0,892403044	0,916956578	1,169441145	0,841759644	0,877367359	0,939585554	0,518259
Q3ULJ0	GPD1L	0,927406476	0,998556248	1,061348769	1,012688507	1,06950131	0,919222613	1,119698411	1,029984075	1,014138114	1,030508905	0,518431
Q9WVK4	EHD1	0,540529332	1,115858526	1,195973494	1,147638648	0,904132373	1,132110859	1,204065214	1,234237436	1,03825178	1,102559533	0,520014
Q8JZN5	ACAD9	1,073155243	1,032991034	0,906934351	0,986919373	1,098792993	0,938374995	0,947324003	0,809610541	0,997436774	0,958307861	0,520737
Q91VF2	HNMT	1,172169677	0,999661949	0,918010698	0,910157675	1,037808107	1,059831838	1,031188449	0,813142062	0,751181616	0,938630414	0,521073
P43274	H14	0,933605057	1,051960484	0,998874068	1,015560391	0,85571212	0,841214146	1,083057021	0,939149304	1,071585157	0,95814355	0,523137
Q9JHR7	IDE	1,368478118	1,042583978	0,574232945	1,014704959	0,593028313	0,740889016	0,959444821	0,776587946	1,274920295	0,868974078	0,52374
P20444	KPCA	0,638690233	1,135190116	1,058557788	1,167561862	0,800531854	0,660493639	0,996908226	0,882070357	1,170979804	0,902196776	0,523871
Q3TXX4	VGLU1	1,001868987	0,908089931	1,089175217	1,000865866	1,125090514	0,976069747	0,87557927	0,81849463	0,978034958	0,954653824	0,524322
Q8BLJ3	PLCX3	0,670213681	1,02404525	1,172091327	1,133649742	0,891871034	0,878203108	1,062871897	0,929191636	0,876818066	0,927791148	0,525145
Q61151	2A5E	1,04515733	0,843933967	0,895233256	1,215675448	1,027349265	1,203758636	0,949254362	1,200722715	0,946518877	1,065520771	0,526553
O88544	CSN4	0,940434809	1,099872136	0,958009973	1,001683082	0,837385521	0,962023227	0,97544695	1,034741063	1,022500874	0,966419527	0,528282
Q9ET22	DPP2	0,994974924	1,115586592	0,78881827	1,100620213	0,966245043	1,011799157	0,865230007	0,902268982	1,00935331	0,9509793	0,528866
P51855	GSHB	1,088524782	0,891832998	0,947843528	1,071798691	0,78575153	0,990237938	1,046732302	1,059897929	0,875572628	0,951638465	0,529039
Q61792	LASP1	1,092324126	0,998382576	0,839182599	1,070110699	1,070756843	1,044289604	1,010275645	0,883600224	0,665904133	0,93496529	0,529516
Q9CQF9	PCYOX	0,949056886	1,029490431	0,870897252	1,150555431	0,998963334	1,027502699	0,970627562	1,033872175	1,197174682	1,045628091	0,529524
Q9WUM4	COR1C	0,963090321	1,067020644	1,009930751	0,959958284	0,999215929	0,955414694	1,047730649	1,06145252	1,038648535	1,020492465	0,53032
O70172	PI42A	0,857789083	1,227331125	0,919536535	0,995343257	1,069136805	0,77370884	1,08937813	1,334400251	1,144105514	1,082145908	0,530572
Q61187	TS101	0,988408457	1,165322317	0,927358038	0,918911188	0,951117705	0,923139352	0,914239186	1,022541986	1,004549709	0,963117588	0,530854

Q9CZW5	TOM70	1,029155304	0,975091074	1,040679025	0,955074598	1,000906615	0,934087874	0,991279993	1,069063877	1,162847511	1,031637174	0,530955
P26443	DHE3	0,994594003	1,032333184	0,959227388	1,013845425	1,033452297	0,882475423	1,140417276	1,129968457	0,996321572	1,036527005	0,531053
Q3THW5	H2AV	0,917679139	0,994513328	1,087280417	1,000527116	0,991783243	1,053995881	1,181814943	0,936060116	1,019709669	1,03667277	0,531151
O35685	NUDC	1,056232513	1,044280247	0,959414728	0,940072512	1,045977559	1,153762055	1,132738661	0,976469853	0,8918462	1,040158866	0,531513
Q8BI08	MAL2	1,075107339	0,938962396	1,044769601	0,941160664	1,050550462	0,989750627	0,925085709	0,944177895	0,960484972	0,974009933	0,532694
Q9D154	ILEUA	1,156920909	1,057010762	0,851444387	0,934623942	1,213013558	1,091102892	1,042506005	1,039556746	0,88715431	1,054666702	0,534672
Q6PB66	LPPRC	1,053054887	1,095813195	0,805643083	1,045488836	1,174557765	1,094966849	1,063709942	0,943583024	0,967538939	1,048871304	0,535859
Q80VP1	EPN1	0,923142904	0,989566441	1,018305116	1,06898554	1,002833975	0,865687302	0,985244972	1,184077739	1,220635151	1,051695828	0,536801
Q9R1T4	SEPT6	1,119298678	0,910921686	0,95548553	1,014294105	1,140635459	1,059300416	1,004744931	0,995134994	0,970207177	1,034004595	0,537485
Q9CYT6	CAP2	0,995677698	1,006395593	1,013903671	0,984023039	1,0432468	0,889230991	0,961168273	0,996708272	1,011318455	0,980334558	0,538131
P53026	RL10A	0,927287649	0,937013332	1,032005815	1,103693204	1,028830673	1,200462723	1,165102446	0,910268295	0,939716888	1,048876205	0,538876
Q8K183	PDXK	0,895691395	1,05465241	1,00128006	1,048376135	0,864311503	0,973046555	1,044348854	0,963502984	1,003711531	0,969784286	0,539235
Q03517	SCG2	1,179857737	1,430227733	0,071358612	1,318555918	0,918867702	1,075377091	1,28212335	1,286727088	1,375932072	1,187805461	0,539711
Q91VZ6	SMAP1	0,996609651	1,008200439	0,998033863	0,997156047	1,050818376	0,953903611	0,952614791	0,93507327	1,024454442	0,983372898	0,542084
Q5DTL9	S4A10	0,977757755	0,977658203	1,044513616	1,000070426	1,043704853	0,823612474	0,95023663	0,837217826	1,128144442	0,956583245	0,543584
P61264	STX1B	1,103886367	0,931360728	0,88557484	1,079178065	1,060069406	1,039352862	1,034654562	1,102940204	0,940497219	1,035502851	0,547348
Q3UNZ8	QORL2	1,231139056	1,1928746	0,33874941	1,237236934	1,144965558	1,273490387	0,999500952	1,088754759	1,124752937	1,126292919	0,548245
P35762	CD81	0,827663794	0,820439223	1,148340833	1,203556149	0,127760428	1,228629875	0,899984749	0,82538057	1,167166752	0,849784475	0,550818
Q8R1V4	TMED4	1,327641062	1,335368292	0,383658459	0,953332187	1,137758925	1,630246588	1,048661272	0,991288899	0,947124942	1,151016125	0,552025
Q9WUC3	LY6H	0,763432451	0,793797062	1,223864225	1,218906262	0,721517162	1,4796441	1,270606075	1,046933539	1,050173268	1,113774829	0,552492
Q8R5C5	ACTY	1,001924755	1,075701561	1,014243162	0,908130522	1,079453251	1,019774582	0,967327619	1,003987668	1,045139816	1,023136587	0,553676
Q3THE2	ML12B	1,064961819	0,979099369	1,036441935	0,919496877	1,046040097	1,057669598	0,998891388	0,789320062	0,912213607	0,96082695	0,555621
Q62442	VAMP1	0,794046551	1,209606159	1,0192004	0,977146889	0,936038019	0,993955911	1,087704283	0,90524442	0,789371578	0,942462842	0,557583
Q99PU5	ACBG1	1,026154538	1,065400888	0,880839728	1,027604846	0,99558556	1,177133798	1,00458563	1,015731167	0,974080606	1,033423352	0,56102
P24529	TY3H	1,05919306	0,759514179	1,331033802	0,850258959	1,358504751	0,752828618	0,788851971	1,114597952	1,676316115	1,138219881	0,56206
Q91YQ5	RPN1	0,972766058	0,944178138	1,022664494	1,06039131	0,986914536	1,040717113	1,051277823	0,963320853	1,051245953	1,018695256	0,562302
Q91W89	MA2C1	0,992488499	1,02208092	0,94389946	1,041531121	1,198927037	0,973534828	1,002292616	0,934212241	1,059639506	1,033721245	0,562556
Q8BX94	OSBL2	0,430973042	1,025423034	2,03136475	0,512239174	0,664963406	0,445256033	0,519476399	0,797666632	1,425281141	0,770528722	0,564066
Q9Z2I9	SUCB1	0,967391185	1,003488621	0,976512686	1,052607509	0,990442162	0,92036608	1,068863189	1,093173151	1,044315059	1,023431928	0,565367
Q8BGT8	PHIPL	0,963477728	0,978525219	1,018969127	1,039027925	1,08524824	1,041293255	1,109651514	0,996884844	0,901043324	1,026824236	0,566113
P13020	GELS	1,118323369	1,091042221	0,853694944	0,936939466	1,040736976	1,101382548	1,178111912	1,00945465	0,899783119	1,045893841	0,567255
Q9Z1P6	NDUA7	0,980122577	1,141450818	0,837168352	1,041258253	0,985043126	1,153022506	1,169302462	1,053899888	0,883921355	1,049037867	0,569887
O55100	SNG1	1,013773593	0,93323577	1,055806821	0,997183816	1,09702319	0,960009189	0,911064959	0,931353403	0,972393284	0,974368805	0,570668
Q05816	FABP5	0,893786608	1,071243435	0,987173728	1,047796229	0,947679848	0,915459535	1,09264156	1,006636028	0,872184954	0,966920385	0,570965
O55125	NIPS1	0,986984048	1,000714083	0,922393819	1,08990805	0,968243602	0,631030462	1,063034275	1,028054603	1,027696567	0,943611902	0,572365
Q9Z2U1	PSA5	0,94913604	1,01341591	1,013048292	1,024399759	0,953414461	0,919006135	1,078382305	1,001139108	0,943628627	0,979114127	0,572881
P56564	EAA1	0,897084441	0,999466722	1,113100999	0,990347838	0,798662536	1,042608769	0,948712798	0,925558405	1,082460134	0,959600528	0,572977
Q9CZT8	RAB3B	1,174169824	0,853844333	0,906441719	1,065544123	1,062589537	1,078553373	0,802598332	0,958927774	0,828293667	0,946192537	0,575338
P60843	IF4A1	0,739804207	1,060689669	1,095374067	1,104132058	1,089823075	0,888186683	1,051439795	1,043978088	1,205623436	1,055810215	0,579001
Q9Z127	LAT1	1,279715683	1,466584119	1,188312186	0,065388012	1,322200635	1,328669364	1,171691102	1,166747986	0,865521184	1,170966054	0,579321

P54227	STMN1	0,990314383	0,911538847	1,031838841	1,066307929	1,002178112	0,967066806	1,003107633	1,016063682	0,904687103	0,978620667	0,583128
P16546	SPTN1	0,93666246	1,016053863	1,046948132	1,000335545	1,077468502	1,013562782	0,959726136	0,966154077	1,088839574	1,021150214	0,583604
P63242	IF5A1	0,855960433	1,068174498	1,051904467	1,023960602	0,966895885	0,855075903	1,093244593	0,978851631	0,929888687	0,96479134	0,584226
O55126	NIPS2	1,009921321	1,007473908	0,975135378	1,007469394	0,977741884	0,854419987	0,999386552	1,022452692	1,038316488	0,97846352	0,586201
P48036	ANXA5	1,081133514	1,03401526	0,871156584	1,013694642	1,035259816	0,955142842	1,06866428	0,941403329	0,820195762	0,964133206	0,587202
Q5XJY5	COPD	0,963482368	0,992904759	1,047578305	0,996034567	1,060924875	0,840905324	0,969130903	1,049577491	0,943502756	0,97280827	0,587485
Q9QYB1	CLIC4	1,148647712	0,989427596	0,8776032	0,984321492	0,994756955	1,103726998	0,994482095	0,931743355	0,735595474	0,952060975	0,588642
P08551	NFL	1,264323968	1,108765773	0,702205213	0,924705046	1,206961515	1,147901551	1,086848577	1,08022921	0,83944416	1,072277003	0,589595
Q60902	EP15R	0,782069598	1,012471381	1,215089657	0,990369364	1,117938127	0,857539529	1,011706529	0,767134265	0,952821451	0,94142798	0,590226
Q9CWS0	DDAH1	1,047967679	0,96866431	0,962375183	1,020992827	1,144045034	1,093026748	0,952075524	1,022986118	0,928111818	1,028049049	0,59096
Q9WV60	GSK3B	1,005912522	0,956247673	1,092648856	0,945190948	0,954559671	0,839046386	1,078532017	0,972112881	1,006908268	0,970231845	0,59341
Q8BJI1	S6A17	0,881158813	1,031760811	1,081429804	1,005650571	1,028022296	0,881633063	0,936254129	0,878328454	1,102319511	0,965311491	0,593617
Q9D1G5	LRC57	1,322009092	1,084605525	1,280628073	0,31275731	1,056361789	1,224768827	1,164834797	1,209809843	0,944346642	1,12002438	0,593768
O35344	IMA4	0,913472321	1,051603575	1,032095282	1,002828822	1,031912529	0,942580866	1,004430232	0,997594593	0,925639887	0,980431621	0,594357
Q61595	KTN1	1,960613337	0,680268385	0,577035281	0,782082997	0,764255568	1,069612037	0,688511795	0,866145709	0,797772149	0,837259452	0,595871
E9PUL5	PRRT2	1,212433972	1,044419464	0,836922963	0,906223601	1,119396633	1,020614796	0,899107839	0,874992334	0,821652648	0,94715285	0,595894
Q99K51	PLST	1,107716967	0,997462376	0,866249578	1,028571079	1,241905725	1,02951831	1,005739612	0,895185913	1,041452387	1,042760389	0,598034
Q62108	DLG4	0,927197909	0,872209983	1,178825785	1,021766323	1,108566772	0,936917524	1,008396959	0,98461364	1,172668716	1,042232722	0,598116
P58771	TPM1	1,042917333	0,99911066	1,03806347	0,919908537	1,044813362	0,872879457	1,033562294	0,932059131	0,994212091	0,975505267	0,598236
Q80ZJ6	ZER1	0,941719521	1,044683108	0,885898663	1,127698708	0,975362089	0,771145809	1,010572523	0,986206218	1,056612228	0,959979773	0,60123
P61164	ACTZ	1,050535426	1,113066049	0,79757812	1,038820405	1,14624068	1,118431654	0,986909515	0,991567553	0,960768124	1,040783505	0,601834
Q9Z0Y1	DCTN3	0,977242	1,021432768	0,940580398	1,060744833	0,952545322	1,030872219	1,045950957	1,008490024	0,821845026	0,971940709	0,603347
P13707	GPDA	1,092298058	1,024248775	0,797300981	1,086152185	1,095632322	0,829829139	1,177623339	1,232858395	0,946485489	1,056485737	0,604058
Q9CQZ5	NDUA6	0,96635513	1,006062729	0,992599929	1,034982212	0,917531975	1,159204848	1,210202264	0,946571539	0,956287824	1,03795969	0,604724
Q9ES97	RTN3	0,991700073	1,021336432	1,00096296	0,986000536	1,123102291	1,043157893	0,976375461	0,971943746	0,977116867	1,018339252	0,605779
Q9CX86	ROA0	0,604909751	1,114852265	1,123919595	1,156318388	0,6132559	0,994294748	1,064320409	0,966508976	0,965947253	0,920865457	0,605796
Q8R2Y0	ABHD6	1,04326083	0,869371236	1,084232737	1,003135197	1,158290913	0,915759219	0,810547462	0,86201938	1,032264237	0,955776242	0,606036
Q99JP7	GGT7	1,008976122	1,000330801	1,011500521	0,979192557	1,016713778	0,910188121	1,029986421	0,810787284	1,08253977	0,970043075	0,606474
P24369	PPIB	0,950253493	1,08308516	1,085085037	0,881576311	1,008676373	1,052079145	1,085680625	1,003854387	0,982139204	1,026485947	0,606762
D3YVF0	AKAP5	0,776235874	0,523574909	1,64073298	1,059456237	1,033490828	0,930288936	0,912194819	0,975627251	2,00190308	1,170700983	0,606971
O08756	HCD2	1,105048739	0,97895429	0,954120188	0,961876783	0,946977253	1,084034675	0,947219707	0,978706209	0,922933303	0,975974229	0,608266
P62717	RL18A	0,902541717	1,044279247	1,048250931	1,004928105	1,078683227	0,980892892	0,985582143	0,864010381	0,960145434	0,973862815	0,609752
Q9JKV1	ADRM1	0,972031951	1,163523658	0,832780239	1,031664153	1,001637981	1,11074276	1,120659619	0,714425667	0,741166591	0,937726524	0,610634
P60904	DNJC5	1,096045533	1,099839681	0,855962505	0,948152281	1,0148729	1,087168975	0,949728013	0,82461256	0,931349805	0,96154645	0,61072
P51660	DHB4	0,982610817	0,97672996	0,973011455	1,067647768	0,97951607	1,009154442	1,060727894	0,94413274	0,913232457	0,981352721	0,612455
P37040	NCPR	0,986491346	1,1404036	0,882550321	0,990554733	0,701997474	1,080568223	1,119936529	1,217498963	1,182146126	1,060429463	0,615451
Q8K377	LRRT1	1,084366177	0,948241854	0,848607547	1,118784422	1,514321939	1,135292761	0,762566557	0,99387127	0,988905428	1,078991591	0,617186
Q3UHJ0	AAK1	1,077238585	1,051783429	1,008539997	0,862437989	1,10698288	0,860584333	0,959198296	0,921491704	0,988014067	0,967254256	0,617555
P40142	TKT	0,978810044	1,091023872	0,91882141	1,011344674	1,00909088	0,988621262	1,067113854	1,067047216	0,969614188	1,02029748	0,617839
P70175	DLG3	0,921778592	0,96473246	1,084036172	1,029452776	1,040502485	0,874629502	0,89428011	0,915908192	1,114821049	0,968028267	0,619585

Q8BGD9	IF4B	0,989933711	1,387324703	0,683161555	0,93958003	0,94552642	1,032118422	0,71400188	1,159532955	0,731311787	0,916498293	0,619791
P61971	NTF2	0,905486048	1,034047712	1,071280136	0,989186104	0,85932324	1,094108143	1,139611169	0,94261805	0,727971898	0,9527265	0,620407
P42669	PURA	0,93651178	1,101201394	0,92326002	1,039026807	1,008063037	0,981510928	0,981831816	0,995589508	0,929420615	0,979283181	0,623119
Q99L13	3HIDH	1,072118756	1,051206883	0,90839139	0,968282971	1,03582527	0,944579641	1,060077574	0,944070886	0,889877819	0,974886238	0,623772
Q9JLM8	DCLK1	0,921844403	0,971881586	1,20654396	0,899730052	1,301372562	0,903455915	0,991275767	1,019558713	1,034957552	1,050124102	0,624166
Q922Q8	LRC59	1,104441925	0,988558665	0,914969193	0,992030216	1,055738952	1,102283019	0,880644952	0,924339652	0,875492655	0,967699846	0,625084
Q8C166	CPNE1	1,004815687	0,876351703	1,061615999	1,057216611	0,957898157	0,709965846	1,038370991	1,149744681	0,91143218	0,953482371	0,625448
Q62093	SRSF2	0,953536841	0,99721655	1,112228068	0,93701854	1,058013085	0,983336161	1,055568735	1,013519788	0,988401533	1,01976786	0,628978
Q9QXL2	KI21A	0,972949503	0,966290064	1,005012338	1,055748094	1,231750029	1,061616977	0,888019669	0,903861546	1,104601924	1,037970029	0,629222
P61027	RAB10	0,752340362	0,956764499	1,436886048	0,854009091	0,837242913	0,780506101	1,016992007	1,128025488	0,854183895	0,923390081	0,629376
P28660	NCKP1	0,943013998	1,0040164	1,035114332	1,017855269	1,059653501	0,887348393	0,883576564	0,906723735	1,116282444	0,970716927	0,629866
Q9JLV5	CUL3	0,982068513	0,968454516	1,02123332	1,028243652	0,964469136	0,995873473	1,045175366	0,868144621	1,031085963	0,980949712	0,63123
E9Q8I9	FRY	1,000469619	1,003250085	1,024353639	0,971926658	1,06826653	0,866791408	0,835790954	0,8028593	1,204996813	0,955741001	0,632367
P63141	KCNA2	0,956201038	1,050063395	1,039368465	0,954367102	1,011992369	1,086686175	0,911242996	0,941703106	0,942986578	0,978922245	0,634669
Q8K212	PACS1	0,578821725	1,112624902	1,161876221	1,146677151	1,123697665	0,991374046	1,139154505	0,9000587	1,183119213	1,067480826	0,638143
O88487	DC1I2	0,971398565	0,988032779	1,071340876	0,96922778	1,060483911	0,916535301	1,11461554	0,728752872	0,985750678	0,96122766	0,638481
Q8CGA0	PPM1F	0,773231979	1,075433294	1,039718381	1,111616345	0,795446451	0,916571974	1,025952873	1,104574544	0,936872199	0,955883608	0,638752
Q9Z2U0	PSA7	0,974476672	0,954153852	0,985595768	1,085773707	1,077561685	1,085772034	0,998105136	0,963128659	0,971638938	1,01924129	0,639259
P27659	RL3	0,910128836	1,079826162	0,949574458	1,060470544	1,045584761	1,030756913	1,047201013	1,048241182	0,935816148	1,021520003	0,639269
A2AGT5	CKAP5	0,825334178	1,086249832	0,943095909	1,145320081	1,172071275	0,800741661	1,079066481	1,13471716	1,051273414	1,047573998	0,639896
P48318	DCE1	0,972279197	1,121909504	0,912317078	0,993494221	0,952519254	0,940934727	0,948959713	1,062318893	0,982877515	0,97752202	0,642224
Q3UHB1	NT5D3	1,072497393	1,207478236	0,528271831	1,19175254	0,966070247	0,84900843	1,010619949	1,012050197	0,80200347	0,927950458	0,643322
Q61879	MYH10	0,934954516	1,002734627	1,128291068	0,934019788	1,137711108	1,038665614	0,936521783	0,89553471	1,167361538	1,03515895	0,643549
Q9ERK4	XPO2	0,959761342	1,033431695	0,967401987	1,039404976	1,048365389	1,070048741	0,985505833	0,992654199	0,971850381	1,013684908	0,646813
O88543	CSN3	0,910583216	1,10236833	0,857796715	1,129251739	1,026534269	0,92148848	1,105782161	1,038334464	1,073304717	1,033088818	0,647842
Q9R0K7	AT2B2	1,079517313	0,966185704	1,025857039	0,928439944	1,19285067	1,078355086	0,937635779	0,913107044	1,03114647	1,03061901	0,64843
Q8K3E5	AHI1	0,850910993	1,109957395	1,003837048	1,035294563	0,764479706	1,194942191	1,093161346	0,816041673	0,882044268	0,950133837	0,650754
Q99LI8	HGS	1,127619849	0,865353898	0,882808729	1,124217524	1,066849089	1,01767913	0,857479889	0,705357106	1,103613689	0,950195781	0,651865
Q60864	STIP1	0,959388437	1,072811599	0,959632344	1,00816762	0,989063256	0,941249117	1,046230181	1,044034761	1,061015073	1,016318477	0,652081
P62702	RS4X	0,974902898	0,893053226	0,989289166	1,14275471	1,034877531	0,857713709	1,098150339	0,951872836	0,898880928	0,968299069	0,653557
Q8CDN6	TXNL1	0,915223571	1,057267117	0,982919747	1,044589564	1,026066766	0,993198817	1,105480227	0,977217073	0,98913599	1,018219775	0,654
Q9R1P1	PSB3	1,030454559	1,02635535	0,926103531	1,017086561	1,029779755	1,2615267	0,989919265	0,955943631	0,92793965	1,0330218	0,656036
Q8C167	PPCEL	1,114128051	1,125825151	0,851557552	0,908489245	1,020276251	0,887670079	0,914362137	1,090633078	0,911448233	0,964877956	0,657423
P17047	LAMP2	0,035501189	1,47897584	0,992907071	1,492615901	1,203671634	0,933693529	1,258824032	1,166020815	1,147740753	1,141990152	0,657507
Q921M7	FA49B	1,085598377	0,965194997	0,981079936	0,96812669	1,072078694	1,059509889	0,977508668	0,90540662	0,86363431	0,975627636	0,65968
Q99L47	F10A1	1,00987989	1,052139676	0,937606265	1,000374168	1,000047215	0,955172566	1,025828397	1,046989925	1,035937365	1,012795094	0,659875
Q14BI2	GRM2	0,910805825	0,880106346	1,092667701	1,116420128	1,270285314	0,823403272	0,879319782	0,99166431	1,328821232	1,058698782	0,660048
Q9QWI6	SRCN1	0,994006366	0,999433825	1,062343885	0,944215924	1,145995257	0,755232711	0,96985929	0,943089557	1,014416642	0,965718692	0,66009
Q9Z2Y3	HOME1	0,879933491	0,805420958	1,328988322	0,985657229	1,060144735	0,718223999	0,811758227	0,981533636	1,124310321	0,939194183	0,662131
Q4V9Z5	SE6L2	0,704662123	1,069769942	1,085616288	1,139951647	1,112859019	0,472013521	1,032002893	1,106815915	0,910892089	0,926916687	0,663989

Q91Z53	GRHPR	1,013167025	1,083201524	0,758123896	1,145507556	1,124682153	0,981175872	0,885589739	1,075614967	0,66466406	0,946345358	0,665186
Q60737	CSK21	1,01386886	0,943031917	0,989750522	1,0533487	1,028352582	0,963506776	0,946189897	0,866321606	1,089498937	0,97877396	0,668252
P49443	PPM1A	0,943334879	1,034609764	1,060350862	0,961704496	1,084099104	0,95544903	0,966011774	0,932444931	0,975674789	0,982735926	0,669641
Q9Z0J4	NOS1	0,70499088	0,935453997	1,282215834	1,077339289	0,969241081	0,979184058	1,046698134	0,948303602	0,798240982	0,948333571	0,670282
Q9D379	HYEP	0,999935044	1,066997678	0,990325474	0,942741805	0,876553204	1,073890947	1,06172782	0,9452365	0,934658486	0,978413391	0,672664
P51150	RAB7A	0,975948316	1,002680397	1,011087571	1,010283716	0,991698861	1,009754523	1,02764874	1,014535676	0,894476884	0,987622937	0,673382
Q62261	SPTB2	0,949936576	1,062170779	1,011796973	0,976095672	1,128023532	0,938714569	0,982021344	0,990651196	1,055049864	1,018892101	0,674294
Q8BG32	PSD11	0,947697513	1,006972404	1,051037207	0,994292877	1,065493604	0,916507783	1,009656958	1,116250205	0,985915659	1,018764842	0,675511
P99029	PRDX5	0,961761375	1,02551667	1,005059742	1,007662213	0,948074369	1,05140502	1,01570118	1,04649238	0,992048045	1,010744199	0,675714
Q8C1B7	SEP11	1,123557441	0,954594791	1,03939333	0,882454438	1,069262856	1,02680078	1,000160955	0,986355889	1,023041145	1,021124325	0,676719
O70493	SNX12	0,965142147	0,990431079	0,995086494	1,04934028	1,002486387	1,045171569	0,995845672	1,050170097	0,959521	1,010638945	0,678488
Q6PHZ2	KCC2D	0,867855992	0,999517374	1,148425632	0,984201002	0,977786064	0,906699976	0,950776359	1,04143927	1,001017981	0,97554393	0,679449
Q8K1M6	DNM1L	0,933113141	1,051817878	1,016688164	0,998380817	1,050567824	0,94816313	0,957799776	0,924115897	1,040849146	0,984299155	0,679548
Q8VEH3	ARL8A	1,080891344	1,063101077	0,86244732	0,993560259	0,96546517	1,111608021	0,230802641	1,269238829	0,977493581	0,910921649	0,680248
P62880	GBB2	0,886993561	0,965748185	1,135431232	1,011827022	0,78447509	1,128732105	0,958137926	0,963039177	0,999546517	0,966786163	0,680512
Q3UVL4	VPS51	0,903373228	1,057471132	0,983145852	1,056009788	0,981754107	0,854910856	0,987631165	0,809968099	1,190691728	0,964991191	0,681323
P14211	CALR	0,979046533	1,038420936	0,943912405	1,038620126	0,95516522	0,97366605	1,052407391	1,000782544	0,955952736	0,987594788	0,682711
Q8BX70	VP13C	0,784680896	1,239065802	1,042835908	0,933417393	1,032330483	1,02845132	0,976594384	0,972667337	1,192505894	1,040509884	0,684073
Q9DBR7	MYPT1	0,776301069	1,149714311	1,019679348	1,054305272	0,965232782	0,91262241	0,93496299	0,935113831	1,086623517	0,966911106	0,684863
Q8R2R9	AP3M2	0,425639655	1,043424877	1,31653127	1,214404198	1,138713296	1,002293956	0,908028748	1,186533581	1,154196098	1,077953136	0,68652
P54823	DDX6	0,937533409	1,027783087	1,084171008	0,950512496	1,016443985	0,845723022	0,973134121	1,033891478	1,026003121	0,979039145	0,686642
P57746	VATD	0,939768439	0,997361312	1,009054686	1,053815563	1,072865926	0,937617559	1,038967476	0,998950691	0,84715717	0,979111765	0,687217
Q9CQN1	TRAP1	1,027023228	0,95316888	0,89094211	1,128865783	0,994833026	1,111356493	0,964059087	0,994860172	1,047053361	1,022432428	0,688685
Q60692	PSB6	0,984967243	0,970273752	1,036193235	1,00856577	0,992273894	1,064309745	1,080696941	0,933507267	0,810180591	0,976193688	0,689515
Q6ZWX6	IF2A	1,055064746	1,086796962	0,893393203	0,964745089	1,137204825	0,993289568	0,958322511	0,950263418	0,815390468	0,970894158	0,689775
Q8R191	SNG3	1,188596192	0,975581865	1,126937487	0,708884457	0,981622513	1,217902198	1,009434284	0,810848579	0,693083206	0,942578156	0,690863
P37804	TAGL	0,79099109	1,1467709	1,223452621	0,838785389	0,172713654	1,999064433	0,763751223	0,764315612	0,55756972	0,851482928	0,692602
Q9R0P3	ESTD	1,085057732	1,008505869	0,897631574	1,008804825	1,000333524	1,133816313	0,978829342	0,836904093	0,916070153	0,973190685	0,693543
Q61102	ABCB7	1,158447986	0,96817494	0,964260313	0,909116761	1,093596122	0,94885874	0,880614602	1,008309494	0,939369505	0,974149693	0,693717
Q9QUH0	GLRX1	0,749043833	1,052909576	1,198795525	0,999251066	0,960549652	0,932836088	0,864146633	1,079284262	0,977449028	0,962853133	0,696315
O08759	UBE3A	0,775984631	1,18580808	0,805767162	1,232440126	0,905764432	1,106469827	1,110834108	1,082483982	1,027832602	1,04667699	0,696894
P17742	PPIA	0,868232287	0,891651694	1,122931236	1,117184783	0,935918942	0,928987733	1,022149936	1,013234436	0,968231298	0,973704469	0,696999
P16125	LDHB	1,068386547	0,959956771	0,949502774	1,022153909	1,060384836	1,003362698	1,007941305	0,964058868	0,878963901	0,982942322	0,697229
Q9ERS2	NDUAD	0,938425634	1,052247977	1,009433655	0,999892734	0,923466015	1,005998517	1,083411677	1,07436773	0,991445056	1,015737799	0,699591
Q922Q4	P5CR2	1,009544857	0,951815495	0,986976451	1,051663197	1,066804004	0,983351944	0,950537498	1,020673933	1,037936288	1,011860733	0,7003
Q8BWF0	SSDH	0,999468143	1,021191462	0,973619323	1,005721072	0,965637876	1,085690416	1,009577062	0,929276576	0,943665048	0,986769395	0,701699
P47791	GSHR	1,176146993	0,847815534	0,892814362	1,083223111	0,72574516	0,985943787	1,093165174	0,951044215	1,046192262	0,96041812	0,702067
Q8VEK0	CC50A	0,202627678	1,232329431	1,434589072	1,13045382	0,834505947	1,162931354	1,101852551	1,12690283	1,277708881	1,100780312	0,703191
Q91WQ3	SYYC	0,99134443	1,02118385	0,983695018	1,003776702	1,029458439	0,978716529	1,046091131	0,968640268	0,926839553	0,989949184	0,704717
Q76MZ3	2AAA	0,936832627	1,072664621	0,994600784	0,995901968	0,869956245	1,020610264	1,041418019	1,07126295	1,097700326	1,020189561	0,705674

P62761	VISL1	0,965631986	1,00344518	1,008546436	1,022376398	0,911560538	0,993469197	1,091695273	1,051897983	0,851467016	0,980018001	0,708006
Q8R1I1	QCR9	0,961544381	1,072472826	1,099486043	0,86649675	0,899323868	0,94810284	0,979812364	1,097884151	0,95775516	0,976575677	0,708607
Q8R5J9	PRAF3	1,010367545	1,024747649	0,980124036	0,98476077	1,032221369	0,915577107	1,035686754	0,999514294	1,080172202	1,012634345	0,708893
Q0VBF8	STUM	0,715774313	0,913160318	1,25319174	1,117873628	0,965414067	0,784572755	0,998657407	0,870445948	1,141703949	0,952158825	0,711188
P62482	KCAB2	1,074570679	1,049389769	0,838256174	1,037783377	0,988102892	1,124297966	0,936890031	0,904077197	0,922576764	0,97518897	0,717157
P50544	ACADV	1,013941902	0,90086203	1,000232949	1,084963119	0,849060944	1,01520694	1,052913968	1,044870116	0,934136758	0,979237745	0,717546
Q8R0Y6	AL1L1	0,983011106	1,070581148	0,938432813	1,007974934	0,964655174	1,075980239	0,989339993	0,958829269	0,943275225	0,98641598	0,717705
Q3U0V1	FUBP2	0,755652188	1,137831238	1,098171731	1,008344843	0,991929064	0,878502504	0,923727155	0,947217652	1,097186352	0,967712545	0,719636
Q9Z2I8	SUCB2	0,949028001	1,110851992	0,987717334	0,952402673	1,094411798	0,873299396	1,074721933	0,875804973	0,964031626	0,976453945	0,719782
Q8C437	PEX5R	1,034851215	1,070069453	0,875131809	1,019947523	1,058871773	1,036547977	0,993212521	0,949112284	0,859988949	0,979546701	0,721006
B9EJ86	OSBL8	1,050606813	0,954161257	1,120952584	0,874279347	1,035847127	0,990468516	0,878913258	0,957161329	1,317599419	1,03599793	0,722428
P23506	PIMT	0,902219871	0,975425022	0,924365554	1,197989553	1,13302092	0,090160238	1,175059777	1,211964867	0,951819199	0,912405	0,732105
Q99104	MYO5A	0,970056171	1,024567034	1,039210968	0,966165827	1,159898994	1,04454487	0,795164059	0,998234617	1,136645096	1,026897527	0,732257
Q8CGP0	H2B3B	0,842826085	1,058473931	1,012713108	1,085986877	0,941493034	1,040825019	1,143377009	1,041431857	0,945945607	1,022614505	0,73361
P16460	ASSY	0,95349043	0,985444976	1,064315427	0,996749166	0,880894273	0,970887396	1,166883711	1,038533747	1,043901146	1,020220054	0,733819
Q9Z1B3	PLCB1	0,836342591	0,934279522	1,221104869	1,008273018	1,044939731	1,027942222	0,870127721	1,000968924	1,22575995	1,03394771	0,735413
Q9Z1N5	DX39B	0,914823709	0,968931582	1,054144674	1,062100035	1,026981654	0,868175997	1,051871236	1,055554457	1,092619774	1,019040624	0,735587
Q8VCT3	AMPB	1,013708929	0,946971534	0,886855516	1,152464021	1,21150806	0,038623599	0,995954327	1,251086371	1,052066634	0,909847798	0,736768
Q61490	CD166	0,86224845	1,018858142	1,190870163	0,928023245	0,918181804	0,746838429	0,952297975	1,009825032	1,193190111	0,96406667	0,7374
P51880	FABP7	0,847029467	1,07667161	0,918265325	1,158033598	0,977956355	1,172700219	1,199416426	0,898844609	0,917754616	1,033334445	0,737828
Q3UHL1	CAMKV	0,807848015	0,958698632	1,093816785	1,139636567	0,947991494	0,784328923	0,854740742	0,939688182	1,272469655	0,959843799	0,738032
P12382	PFKAL	0,981514827	1,022683796	0,989704672	1,006096706	1,056455998	0,957797584	0,976593274	0,957392638	1,11512889	1,012673677	0,738981
P48678	LMNA	0,983436718	0,986892364	1,056940491	0,972730426	0,930937374	1,000967765	1,239583296	1,025901352	0,919536337	1,023385225	0,73943
Q7TPR4	ACTN1	0,697731328	0,872041184	1,384100166	1,046127322	1,273844489	0,990782984	1,246118363	0,122411449	1,997160042	1,126063465	0,740296
P97315	CSRP1	1,051551881	1,076393372	0,883544347	0,9885104	1,103483699	0,993447904	1,014856627	0,924440717	0,860364122	0,979318614	0,740957
P80317	TCPZ	0,92402125	1,072698309	0,938579209	1,064701232	0,986748321	0,965461927	1,040516523	0,965073497	0,976257063	0,986811466	0,741359
Q64514	TPP2	0,964370159	1,015663556	1,084255498	0,935710787	1,08320794	0,965109947	0,998196557	0,847858094	1,015769618	0,982028431	0,741576
P61202	CSN2	1,103610946	0,891633554	0,943238572	1,061516928	0,79761437	1,133573382	1,128364057	0,905753417	0,882258457	0,969512737	0,741635
Q9D7X3	DUS3	0,851451738	1,046477374	1,029165156	1,072905732	1,107736234	0,935191262	1,004995381	0,906859111	0,942415883	0,979439574	0,742129
P70206	PLXA1	0,821741056	0,97474433	0,941423952	1,262090662	1,067128519	0,993885856	0,924361836	1,070494519	1,096032086	1,030380563	0,744007
Q68FH0	PKP4	0,834192085	1,013631215	1,079528305	1,072648395	1,086303829	0,959633761	0,91844798	0,973312481	0,961309032	0,979801417	0,744451
O54983	CRYM	0,935972311	0,944860034	1,138122171	0,981045484	0,73369596	0,816400956	1,082360825	1,130842451	1,506399526	1,053939944	0,745531
P63158	HMGB1	0,963009187	0,956558095	0,903593555	1,176839163	1,001105947	0,971811217	1,288142549	0,952166668	0,940242912	1,030693858	0,74576
Q68FL6	SYMC	1,052021809	1,115786889	1,015631817	0,816559484	1,111232715	0,963206167	1,077013912	1,057902191	0,909756848	1,023822367	0,746919
Q2M3X8	PHAR1	0,976577603	0,967750559	1,09530455	0,960367288	0,56958221	0,916049916	0,984043597	1,167619106	1,151417974	0,95774256	0,747138
P46096	SYT1	0,936811557	0,97461275	1,023541855	1,065033838	1,083961907	0,827806606	0,992665016	1,121216155	1,081866472	1,021503231	0,749614
P35278	RAB5C	0,936986579	1,166282001	0,861693741	1,03503768	1,062618767	1,376841346	1,103314422	0,889761432	0,174830374	0,921473268	0,749685
Q8CI94	PYGB	0,861116926	1,087458702	1,031659867	1,019764505	1,00937756	1,07425828	0,983408182	0,957677929	1,057055058	1,016355402	0,750134
Q9D6R2	IDH3A	0,971787709	0,991515392	1,00147424	1,035222659	1,000591359	0,979336286	1,029762378	1,001863339	0,958948704	0,994100413	0,750256
P80313	TCPH	0,921059704	1,108722123	0,963070814	1,007147359	0,986528009	0,866452622	1,084632126	1,029627678	0,941607271	0,981769541	0,750546

Q91XL9	OSBL1	1,085654769	1,07715744	0,99411736	0,843070431	1,108351489	1,001555265	1,010628453	1,023926228	0,949870396	1,018866366	0,751686
Q61696	HS71A	0,896025434	1,292768717	1,329338311	0,481867538	0,905600561	0,86317836	1,021648159	0,954323855	0,96430871	0,941811929	0,75212
P05063	ALDOC	1,132850854	0,874444633	0,930861016	1,061843497	1,052205165	1,026384435	0,991429623	0,997282421	0,818227264	0,977105781	0,752747
Q9CVB6	ARPC2	0,732678185	1,048190232	1,007573229	1,211558354	1,010370298	0,956641272	1,133767039	1,062672864	0,990171911	1,030724677	0,753619
Q9CZD3	SYG	0,930949562	1,125572249	1,002947054	0,940531135	0,993627714	0,995844505	0,978329611	1,004226592	1,104463167	1,015298318	0,754343
Q9CQR4	ACO13	0,897503462	1,093011003	1,00002917	1,009456365	0,895379434	1,041079765	1,091981066	0,990380147	0,887913161	0,981346715	0,75471
P70297	STAM1	0,814202356	1,167192867	0,889696432	1,128908346	1,216553938	0,844038433	0,948975351	1,115192906	1,045829706	1,034118067	0,75685
Q921H8	THIKA	0,915590096	1,121851431	0,993292071	0,969266401	0,836373071	0,892594872	0,878674384	0,827892833	1,367616363	0,960630305	0,757089
P70398	USP9X	0,881472338	1,094566688	1,003308064	1,02065291	1,100573307	1,064379721	0,887325411	0,924719505	0,923323752	0,980064339	0,757476
P36916	GNL1	0,945882371	0,99025887	1,063909449	0,99994931	1,040938448	0,997063772	1,182467464	1,153845411	0,345189633	0,943900946	0,758483
Q8K0T0	RTN1	0,945328131	1,071086029	1,040917832	0,942668007	1,012931422	1,014111182	1,04222713	1,009335393	0,971791795	1,010079384	0,759018
P46460	NSF	0,90924377	1,038122184	1,02318898	1,029445066	1,046612897	0,954405242	0,997726321	0,983256804	1,075972862	1,011594825	0,760047
Q64487	PTPRD	0,978168867	0,993478464	1,066406191	0,961946478	1,023896726	1,202370881	0,989751768	0,929416286	0,947835409	1,018654214	0,76071
Q99NB9	SF3B1	0,81811475	1,128698418	1,084072183	0,96911465	1,225874703	0,834397156	0,864694516	1,064110039	1,183666623	1,034548607	0,761435
P62814	VATB2	0,926176407	1,006686672	1,077330432	0,989806489	1,014971328	0,890334327	1,028008459	1,085304887	1,054636144	1,014651029	0,762458
Q9CQM9	GLRX3	1,02703328	1,053015986	0,936840358	0,983110376	1,047220887	1,007032246	1,064799358	0,979599669	0,952277985	1,010186029	0,763363
Q8VCN9	TBCC	0,883083931	1,057497136	1,037877108	1,021541825	1,069651589	1,039011047	0,969746949	1,015124012	0,808917047	0,980490129	0,764455
Q91ZX7	LRP1	0,920812806	1,119127447	1,003776302	0,956283444	1,017452693	1,008486877	0,93743413	0,953677825	1,017476096	0,986905524	0,767665
Q8CGP1	H2B1K	0,82370748	1,098121065	0,968061808	1,110109647	0,893642532	1,028170927	1,244073047	0,954701141	1,015461332	1,027209796	0,769516
Q91WJ8	FUBP1	0,801400335	0,948099479	1,075925179	1,174575008	0,999852311	0,790625102	0,996990592	0,953019925	1,118137259	0,971725038	0,769818
O88685	PRS6A	0,956858865	0,959614671	1,084026611	0,999499853	0,979549264	0,873167842	1,223946413	1,10880366	0,930934922	1,02328042	0,769821
Q04690	NF1	0,768858823	1,107880048	1,034426998	1,088834132	1,050606755	0,862677576	1,045036373	1,090425798	1,077231094	1,025195519	0,771715
Q8VE33	GD1L1	0,847328413	1,052494201	1,082874074	1,017303311	1,041877797	0,919958447	0,956346357	0,968122116	1,033688101	0,983998584	0,772676
Q64331	MYO6	0,939361395	1,098195089	0,948930081	1,013513434	1,05410292	1,331542917	1,06983501	1,10174102	0,068559457	0,925156265	0,774636
Q9QYB8	ADDB	0,925671583	1,077328192	0,994455431	1,002544795	1,087794078	0,935991146	0,977497959	0,992769938	1,068509186	1,012512461	0,775895
Q924N4	S12A6	1,023262516	1,02408107	0,996983642	0,955672771	0,949243039	0,875158934	1,072716979	1,018523778	1,023224411	0,987773428	0,776383
P61022	CHP1	1,004619341	0,975995194	1,005908699	1,013476766	1,019844227	0,954123917	1,085028824	1,052725665	0,804789409	0,983302408	0,776981
Q9DB72	BTBDH	1,14597759	0,988166578	0,84471612	1,021139713	1,31384994	0,759636143	1,068026634	1,084687162	0,944008781	1,034041732	0,778755
Q8VBW6	ULA1	0,922603014	1,003874243	1,046549307	1,026973435	1,207521427	0,8925053	0,951715637	0,823747946	1,010897197	0,977277501	0,779043
O09061	PSB1	1,042572985	0,93156122	0,988493109	1,037372687	0,966002628	0,915007983	1,045244693	1,099642955	1,036954872	1,012570626	0,779221
P55264	ADK	0,99532032	0,864400113	1,051204392	1,089075175	0,998362005	1,093493626	1,019731182	0,945596073	0,852222696	0,981881116	0,781145
Q99JY9	ARP3	0,859091889	1,032442875	1,006062326	1,10240291	0,962324972	0,906070376	1,038735487	1,085999617	1,094235112	1,017473113	0,782904
O88643	PAK1	0,96492552	1,066506885	1,001861532	0,966706063	1,117313226	1,028544793	0,941082103	0,945627601	0,897537415	0,986021028	0,783522
Q62421	SH3G3	1,14984775	0,957745683	0,933971723	0,958434844	1,155936664	0,886844998	0,936498084	0,970932482	0,952196397	0,980481725	0,783527
Q9QUR6	PPCE	1,319358319	1,252784787	0,086016927	1,341839967	1,318534814	1,078884125	0,911118419	1,01163073	1,076015758	1,079236769	0,784397
Q8R001	MARE2	0,9001837	1,116797012	0,890640883	1,092378404	1,169561811	0,861350552	0,993415943	0,965833429	0,896084698	0,977249287	0,786353
Q9Z0S1	BPNT1	0,863529689	0,96083628	1,063794963	1,111839068	1,019933142	0,809362745	1,021195473	1,287391172	1,001601929	1,027896892	0,786371
Q80SW1	SAHH2	0,996362177	1,007704663	0,927219883	1,068713276	0,957624686	1,113660476	1,038490777	0,974722325	0,832983307	0,983496314	0,786449
P19253	RL13A	0,786005486	0,96783917	1,13675759	1,109397755	0,868026656	1,031146502	1,091797502	0,929022061	0,962873673	0,976573279	0,786707
Q91V14	S12A5	0,934770199	1,015492333	1,069751957	0,979985511	1,090874915	0,955567907	0,938762451	0,940139303	1,016637308	0,988396377	0,788397

Q9EQ20	MMSA	1,090898325	1,027507176	0,864537916	1,017056583	0,925476889	1,018359322	1,085635583	1,07569717	0,970795439	1,015192881	0,788721
P52503	NDUS6	1,018629706	1,140790828	0,877084564	0,963494902	0,236819661	1,365148351	1,282026716	0,990673305	0,800913019	0,93511621	0,789435
P61021	RAB5B	1,033703633	1,052561486	0,961438961	0,952295919	1,026703372	1,207878562	1,226071412	0,999866483	0,692443277	1,030592621	0,791308
Q9WV92	E41L3	1,064044035	1,064849681	0,908030555	0,963075728	1,088433896	1,070998469	0,997153431	0,935069533	0,973592823	1,01304963	0,791349
Q4ACU6	SHAN3	1,066646462	0,807795026	0,992462059	1,133096452	1,113443551	0,467579842	0,809466818	0,881061013	1,458408185	0,945991882	0,791512
P52760	UK114	0,894720862	1,031141495	1,025696486	1,048441157	0,932043184	1,342590393	1,081111988	1,003379089	0,789309383	1,029686807	0,792554
P50580	PA2G4	0,905044042	0,966534748	1,088786856	1,039634355	0,97514605	0,966607075	1,081651477	1,106584806	0,94075676	1,014149233	0,79291
P62849	RS24	0,754238415	1,070359135	1,213952804	0,961449646	0,843243723	1,036940723	1,061071979	1,034895227	0,889291239	0,973088578	0,793377
Q8BIJ6	SYIM	1,010132021	1,084639524	0,95680347	0,948424986	0,984347835	1,016240384	0,962934356	0,912124528	1,069484885	0,989026398	0,794666
O54991	CNTP1	1,031119433	1,106553234	0,836115002	1,026212331	0,962362915	1,114269862	0,994298149	1,03242663	0,976454993	1,01596251	0,79556
Q60829	PPR1B	1,231298951	0,875939144	1,166934078	0,725827827	0,994462807	0,827320043	0,865067355	0,126996466	1,764468665	0,915663067	0,795811
Q8VD33	SGTB	1,082688084	1,033881439	0,92734836	0,956082118	1,072069232	0,968928635	0,955781188	1,055382938	0,880264869	0,986485372	0,797024
P56376	ACYP1	0,711938237	0,994709745	1,114530219	1,178821798	0,920572041	0,976058186	1,196548449	1,022612603	1,023388354	1,027835927	0,798165
P26516	PSMD7	0,922008463	1,072333505	1,056634073	0,94902396	1,029818069	0,859953296	1,032512606	0,978303436	1,032658938	0,986649269	0,798254
P14152	MDHC	1,022822216	0,976032609	0,96941683	1,031728345	1,04488915	1,001266317	1,025576905	0,995599195	0,887700523	0,991006418	0,798739
P29391	FRIL1	1,069824485	1,011255028	0,998829053	0,920091433	0,780596365	0,936641353	1,097471159	0,95723714	1,12863305	0,980115813	0,800693
Q9Z268	RASL1	0,908267797	0,913079394	1,13816057	1,040492239	1,114208202	0,927502103	0,927627687	0,997409837	1,123615721	1,01807271	0,800697
Q06185	ATP5I	0,780664432	1,104730333	1,133492121	0,981113114	0,804245877	0,936870225	1,107810637	1,035875274	0,996038795	0,976168162	0,801568
P48771	CX7A2	0,950605448	1,043781714	1,105576065	0,900036773	0,865927564	1,01121432	1,077257881	1,172109458	0,96415253	1,018132351	0,806264
P09103	PDIA1	0,965522963	1,063267381	0,965097985	1,006111671	1,093571186	0,684121859	1,101185929	0,920920089	1,082924521	0,976544717	0,808859
Q07076	ANXA7	1,141630947	0,882861436	1,050691493	0,924816125	0,995123106	0,954310427	0,98214078	1,055126524	0,942441678	0,985828503	0,809386
P62320	SMD3	0,801798855	1,126802008	1,102941096	0,968458041	0,933179323	0,856780948	1,142820351	1,1652997	1,01915394	1,023446852	0,810065
Q8VCW8	ACSF2	0,879065494	1,189698976	0,969771911	0,961463619	0,861902374	1,089382095	1,0470966	0,799731905	1,08942803	0,977508201	0,811192
P70414	NAC1	0,953949091	1,082701648	0,890586808	1,072762453	1,106967594	1,022177049	1,002806259	1,013649939	0,920107287	1,013141625	0,811659
Q8BLF1	NCEH1	1,130040166	0,909685222	1,045327899	0,914946713	0,869200731	0,820743974	1,076256465	1,231978229	1,122377885	1,024111457	0,816226
P42125	ECI1	1,105800225	1,028424489	0,932050148	0,933725137	0,950185453	1,187726344	1,067792864	0,912919875	0,96234699	1,016194305	0,817451
O88737	BSN	0,872964259	1,029841434	1,295267945	0,801926362	1,139732454	1,069102849	0,74545501	0,790690898	1,09556734	0,96810971	0,81905
Q8C5W0	CLMN	1,146587388	0,944841018	0,793384714	1,11518688	1,089915145	0,884831498	0,956645894	1,074730731	0,890059754	0,979236605	0,819323
P62743	AP2S1	0,823591907	1,003629671	1,191180033	0,98159839	0,907268225	1,028244902	1,123214727	1,125543987	0,916573514	1,020169071	0,819935
Q80YN3	BCAS1	1,114827013	1,094780831	0,749417131	1,040975026	1,078857309	0,997239075	1,002749373	1,00903501	1,002643386	1,018104831	0,819972
P56399	UBP5	0,981938669	1,029164557	1,012945451	0,975951323	0,959765197	0,977384004	0,990445998	1,019812772	1,084267108	1,006335016	0,821956
Q9Z0L0	TPBG	1,073929205	0,981331056	1,080332019	0,86440772	0,981835518	0,774745176	0,754839513	1,202668578	1,152710358	0,973359829	0,822137
P84084	ARF5	0,758788798	1,202525277	0,925483538	1,113202387	1,032830304	1,386512728	1,223021313	0,026168603	1,004826282	0,934671846	0,823969
Q9Z204	HNRPC	0,94332172	0,987272589	1,029636734	1,039768957	1,008604636	0,890476326	0,993808678	1,076425737	1,080797232	1,010022522	0,825451
Q91YS8	KCC1A	0,894888025	1,02936363	1,070753982	1,004994363	1,148353149	1,005288951	0,933649539	0,993094582	0,979681574	1,012013559	0,826029
Q8C419	GP158	1,024096361	0,957034555	1,068104247	0,950764838	0,896947993	1,095927036	0,992164993	1,032612418	1,033106578	1,010151804	0,826651
Q8BMG7	RBGPR	0,894736591	1,094607734	1,024757696	0,98589798	1,010427649	0,923528378	1,047859521	0,983116959	0,986405569	0,990267615	0,828354
Q8BWG8	ARRB1	0,96437009	0,961367953	1,094102319	0,980159637	0,973484624	0,816029778	1,018834344	1,109636817	1,013471479	0,986291408	0,829032
Q99PV0	PRP8	1,17719874	1,192350918	1,287382972	0,343067371	1,176571327	0,380882702	1,019320998	1,036310076	1,104075704	0,943432161	0,829283
Q9D172	ES1	0,998205719	1,024023356	0,942816704	1,034954221	1,038093509	1,16904535	1,006007723	0,969334074	0,880532924	1,012602716	0,829687

O35927	CTND2	1,111508929	1,098057168	0,811008146	0,979425757	1,205499914	0,511420893	0,999709479	1,097694446	1,020347566	0,96693446	0,830297
Q80WM4	HPLN4	0,852358662	1,132701953	0,962201567	1,052737818	0,883421324	1,063762069	1,053838304	1,011146755	0,913392151	0,985112121	0,831512
Q9Z0P4	PALM	0,840726018	1,035784514	1,098999241	1,024490227	1,06432552	0,919922064	0,987050947	1,030804074	1,061225646	1,01266565	0,832317
I00001	GFP_MALE	1,206963216	0,910522841	1,054719147	0,827794796	0,955908484	1,107179208	0,904524833	1,032411694	1,094057753	1,018816394	0,832417
Q9DBC7	KAP0	1,129167637	0,960273484	0,97352405	0,937034829	1,120385077	0,9033154	0,996418968	1,098311672	0,947312932	1,01314881	0,836142
Q62048	PEA15	0,773118126	1,003399398	1,127228526	1,096253949	0,779358807	0,863382688	1,155800995	1,074440217	1,01468976	0,977534493	0,836816
Q9DCW4	ETFB	1,076383385	0,973023662	0,960189373	0,990403579	0,951922044	1,184396002	1,036144694	0,941494359	0,946861714	1,012163763	0,83839
O88696	CLPP	0,972282643	0,98725372	1,052586457	0,98787718	0,990761032	1,083987601	0,999036265	0,971925277	0,918666653	0,992875366	0,840643
Q78PY7	SND1	0,866765075	1,016175718	1,01727678	1,099782428	0,944314793	0,955463599	1,119596918	0,972459167	1,068461507	1,012059197	0,841281
Q5SYD0	MYO1D	1,08690295	1,143585561	0,854093179	0,91541831	1,003331071	0,93453132	1,077521579	1,093325117	0,963814195	1,014504656	0,8414
P62908	RS3	0,954402193	0,975702475	1,018116231	1,051779101	1,025429011	1,049360883	0,788054615	1,037787628	1,037327536	0,987591935	0,841682
Q9D0E1	HNRPM	0,910971148	1,065456301	1,075395199	0,948177351	0,96241525	0,956865356	0,912452913	1,042309077	1,074950038	0,989798527	0,843173
P97370	AT1B3	1,131495734	1,017522658	0,867485142	0,983496466	1,057579941	1,067922751	1,065445262	1,013240749	0,861526272	1,013142995	0,84598
P15105	GLNA	0,952963103	0,976803672	1,006792904	1,063440322	0,997729202	0,994436449	1,050844828	0,994319744	0,932618368	0,993989718	0,84601
Q9D6K5	SYJ2B	0,925454139	0,97334114	1,088946351	1,01225837	1,019051118	0,997265025	1,122855691	0,969462258	0,829604224	0,987647663	0,846735
P56480	ATPB	1,028230897	0,997536001	0,98828314	0,985949962	0,994555719	0,94603833	0,994407762	1,023739923	1,023118687	0,996372085	0,847523
P24549	AL1A1	1,163417697	0,870978827	0,902670944	1,062932532	1,025343971	0,783221736	0,985361089	1,161919433	1,139487307	1,019066707	0,850819
P62737	ACTA	0,790322231	1,210268681	1,289281413	0,710127675	0,974873337	1,47644458	1,163956056	0,821682508	0,75369434	1,038130164	0,851033
Q9Z130	HNRDL	0,796994624	1,011566137	1,040082771	1,151356467	1,080175186	0,79418926	1,009259417	1,069279588	0,961700812	0,982920853	0,851061
F8VPU2	FARP1	0,95530213	1,060435287	1,05233899	0,931923593	1,032138894	1,096776594	1,006076224	0,870352431	0,944147202	0,989898269	0,852885
Q80UG5	SEPT9	1,030238848	0,986658288	1,020613314	0,96248955	1,16959846	0,858168967	1,039649209	0,96933961	1,01992601	1,011336451	0,85298
Q61330	CNTN2	1,141238605	1,110922004	1,004899276	0,742940114	1,119766635	1,066721386	1,097079971	1,052474687	0,767175353	1,020643606	0,853825
P97300	NPTN	0,963291102	0,894910625	1,124411641	1,017386632	1,059694659	0,881015256	0,923280583	0,947862451	1,124904427	0,987351475	0,855074
P10605	CATB	0,872245498	0,94215976	1,112134981	1,073459761	1,052185809	1,093016942	1,068057103	0,877001958	0,836059985	0,985264359	0,85546
Q3THG9	AASD1	1,0418664	1,005259947	0,824839215	1,128034438	1,022429059	1,093097704	1,000395452	1,089977675	0,862695315	1,013719041	0,857224
Q9WV55	VAPA	0,928236303	0,996940616	1,070051615	1,004771467	0,972901954	1,052964297	1,069541198	0,983522111	0,954861363	1,006758185	0,857752
Q99LC5	ETFA	1,042163902	1,029046443	0,893515487	1,035274169	0,961961385	1,127890041	0,984347293	0,925473889	0,953272073	0,990588936	0,85898
O88533	DDC	1,0656082	0,989060358	0,965975961	0,979355482	0,838191044	0,659169248	0,959307972	1,247680757	1,18131653	0,97713311	0,859624
Q9D880	TIM50	0,943909613	1,272625249	0,989372855	0,794092283	1,068927077	1,388371765	0,988286656	0,699404568	0,992136391	1,027425291	0,862487
P19783	COX41	0,973004471	1,008048714	1,062095412	0,956851404	0,943862894	0,950287911	1,115003558	1,060892665	0,968642387	1,007737883	0,864571
Q9JHU4	DYHC1	0,960362575	1,048188224	1,020000882	0,97144832	1,117814947	1,006369613	0,92528106	0,954475256	0,958516866	0,992491548	0,864834
Q9WUL7	ARL3	0,983709378	1,107227847	1,055201045	0,85386173	1,141687834	0,971913224	0,992063201	1,070348436	0,88482152	1,012166843	0,865483
Q9R1Q8	TAGL3	1,118624183	0,945418312	0,837556758	1,098400747	1,027737802	1,13790743	1,109895576	1,061988148	0,748213131	1,017148418	0,866645
Q61553	FSCN1	1,060291882	0,970410634	0,992429073	0,976868411	1,024763485	0,976385494	0,997172506	1,050064439	0,925507504	0,994778686	0,867694
Q8R1B4	EIF3C	0,904088355	1,123533716	0,974362731	0,998015198	1,097117639	0,925340036	1,049913309	0,914450851	0,964152022	0,990194771	0,868547
O55091	IMPCT	0,928152964	0,980732168	1,040881261	1,050233606	0,879342335	0,929788421	1,130179323	1,003101271	1,01138558	0,990759386	0,869698
Q91V41	RAB14	0,959876366	0,979325218	1,027668635	1,033129781	0,984438232	0,948103317	1,097536139	1,025389382	0,973017627	1,005696939	0,870151
Q9D0L7	ARM10	1,054449765	0,935081535	0,969078262	1,041390438	0,065004715	0,890653061	1,322652025	1,214166254	1,282241223	0,954943456	0,871142
Q9CRA7	ATP5S	1,068459208	0,959993317	0,906627366	1,06492011	0,87523689	0,910839636	1,071231149	1,162175241	0,922167232	0,98833003	0,874932
P63011	RAB3A	1,030244408	0,939940572	0,977091103	1,052723916	1,084865751	1,091982815	0,815379944	1,02570767	1,031622482	1,009911732	0,87642

Q9WVL0	MAAI	1,289080287	0,25014217	1,253580971	1,207196571	0,891875363	1,165295949	1,072964977	0,995030314	0,682622725	0,961557866	0,876912
Q9Z1S5	SEPT3	1,038058039	0,99455285	0,978065921	0,98932319	1,123628523	0,9908618	0,964705829	1,013960648	0,868450936	0,992321547	0,877512
Q9QYR6	MAP1A	0,965864615	1,042842936	0,999835698	0,991456751	1,053001434	1,039396711	0,962838635	0,952458724	0,970357052	0,995610511	0,878175
P28740	KIF2A	0,926533555	1,083418609	0,957955551	1,032092284	1,032113467	0,960226786	1,039716694	0,996292346	0,999533628	1,005576584	0,878372
P49312	ROA1	0,880518817	0,989392173	1,024588671	1,105500339	1,108496042	0,898485033	1,091472711	1,035793267	0,916570809	1,010163572	0,878848
P12787	COX5A	0,747740474	1,151837199	1,040913763	1,059508564	0,730811767	1,002823242	1,085220683	1,055807207	1,042763077	0,983485195	0,880735
Q60605	MYL6	0,954123078	1,16030113	0,797390953	1,088184839	0,838858227	1,095838544	1,173260273	0,991617699	0,9742695	1,014768849	0,881285
O54988	SLK	1,012516178	0,990137528	1,000596853	0,996749441	0,973374499	0,97855624	0,92929878	1,016662352	1,132534632	1,006085301	0,881474
Q9CR16	PPID	0,94181034	0,962017004	0,978112263	1,118060393	1,033452533	0,981527573	1,050983098	1,018964912	0,946613082	1,00630824	0,882435
Q9JJV2	PROF2	0,612074185	1,0039663	1,285747434	1,09821208	0,837690755	0,858224274	1,100934626	1,072017872	1,026210487	0,979015603	0,884556
P28571	SC6A9	1,179215489	1,059513363	0,784407606	0,976863542	1,158681218	1,065322256	0,988074818	0,925258746	0,928997839	1,013266976	0,885147
Q8BLE7	VGLU2	0,837387039	1,199862083	0,964690936	0,998059941	1,031867947	1,128523193	1,009941673	0,89551874	0,992624186	1,011695148	0,885591
Q80UM3	NAA15	0,848568532	1,143937264	0,941418213	1,066075991	1,055760832	0,959618878	0,934610729	1,116023715	0,877871123	0,988777056	0,885728
O08530	S1PR1	0,866409066	1,061864928	1,061914526	1,00981148	0,990529096	1,184143863	0,96263826	0,922735825	0,988195636	1,009648536	0,886963
P54822	PUR8	1,00803857	1,002136763	1,016132207	0,97369246	0,980053096	0,993618911	1,061074824	1,034641912	0,907934794	0,995464708	0,887112
P57776	EF1D	0,982759428	1,032698318	0,962683112	1,021859142	1,006939337	0,984353531	1,034709654	1,011148155	0,946919767	0,996814089	0,88964
P58252	EF2	0,911141795	1,095182295	0,972283723	1,021392187	1,017297488	1,01650537	1,004575131	0,930790346	1,06072998	1,005979663	0,890065
Q99KF1	TMED9	0,896387128	1,117031994	0,945460661	1,041120217	0,856438857	0,865962079	1,148681352	1,180970804	1,01090833	1,012592284	0,890873
Q80XN0	BDH	0,833554067	0,96645336	1,004276253	1,19571632	0,993718571	1,042669575	1,086790871	1,132579635	0,808539012	1,012859533	0,892075
Q8K596	NAC2	1,010759108	0,9242453	1,016321971	1,048673621	1,131189385	0,926466727	0,945591898	0,944732758	1,088408855	1,007277925	0,895796
P62281	RS11	0,953417768	0,888486764	1,148400909	1,009694559	0,791861405	1,055478492	1,278260997	0,92830702	1,015585001	1,013898583	0,89644
P43277	H13	0,837415335	0,966624973	1,039249603	1,156710089	0,867726232	1,099364906	1,016752669	0,950349322	1,118701774	1,01057898	0,897352
O35683	NDUA1	0,871380681	1,064214119	1,009275114	1,055130087	0,903962306	0,894049728	1,117566035	1,10130252	1,027203016	1,008816721	0,89817
O08749	DLDH	1,011773799	1,051500291	0,96576027	0,97096564	1,027706665	0,981227912	0,973509476	1,012985951	0,991005025	0,997287006	0,900703
P34884	MIF	0,76593075	0,998193773	1,198599999	1,037275478	0,788940814	1,095100859	1,030636154	1,081830802	1,067139572	1,01272964	0,903807
P97765	WBP2	0,895059104	0,97511084	1,027213893	1,102616163	1,159996688	1,008253647	1,019137709	0,923554424	0,8449868	0,991185853	0,904695
P10852	4F2	0,981493861	1,067754767	0,920571361	1,030180011	1,084708311	0,84834457	1,0150819	1,017081222	1,002320313	0,993507263	0,904794
Q8BVI4	DHPR	1,105012761	1,024633998	0,874278469	0,996074771	1,103162511	0,986508278	1,113073795	0,993818004	0,750882938	0,989489105	0,905155
O88485	DC1I1	0,934551474	1,089268683	1,012596644	0,963583198	1,068267271	0,912543766	1,038983095	0,981895997	1,024625752	1,005263176	0,905365
Q8VE70	PDC10	0,934979911	1,002737014	1,027423074	1,034860001	0,97900914	0,976172375	0,99285323	1,090736741	0,939740226	0,995702342	0,905671
P09671	SODM	1,065079157	1,018652826	0,948266808	0,96800121	1,041843125	1,105297395	1,004693498	0,909130401	0,909097267	0,994012337	0,906347
P12023	A4	0,989182505	0,969820892	1,015597069	1,025399533	1,073898883	0,895000237	1,003539813	0,840686955	1,147515954	0,992128368	0,906677
P46471	PRS7	0,930235394	0,988260141	1,096065155	0,985439309	1,088573237	0,915588073	1,028420512	0,998530057	0,94111271	0,994444918	0,908134
Q9QUR7	PIN1	0,888672882	1,00436056	1,02884026	1,078126299	0,96566869	1,00817318	1,016309707	1,039946047	0,992432582	1,004506041	0,908836
Q9WTR5	CAD13	0,910298334	1,016498656	1,094194229	0,979008781	1,16919861	0,958990036	0,96811442	1,005783472	0,932144525	1,006846213	0,91023
Q810U4	NRCAM	0,978861792	1,077780253	1,119154474	0,824203481	0,86620982	1,060121029	1,04287885	0,981418598	1,091948374	1,008515334	0,910679
P17809	GTR1	1,117730706	1,025280347	0,920232884	0,936756063	1,046833216	1,077457334	0,968842572	0,9919381	0,943013643	1,005616973	0,911758
Q5M8N0	CNRP1	0,867889772	0,95220819	1,176346602	1,003555436	1,076832985	0,925638351	1,046330221	0,93333203	1,056822371	1,007791191	0,911918
Q61753	SERA	1,006655571	1,07777454	0,957759502	0,957810387	1,015212294	1,010164966	1,031755225	1,04461877	0,918473816	1,004045014	0,912375
P80316	TCPE	0,987005386	1,075503705	0,947373463	0,990117446	1,067808897	0,962449066	0,967267975	1,020483611	0,963157706	0,996233451	0,913795

P16054	KPCE	1,027781653	0,87564191	1,014993847	1,08158259	1,026285144	0,781477617	1,050684223	0,985343703	1,114820431	0,991722223	0,915061
Q61656	DDX5	0,896312724	0,999737025	1,081195359	1,022754892	1,053002074	0,824208319	1,053960728	0,985925649	1,050890714	0,993597497	0,918782
P84086	CPLX2	0,99479224	0,8890208	0,946170885	1,170016075	0,899472753	0,982142683	1,131563201	0,934500195	1,090635046	1,007662776	0,919918
Q8VHJ5	MARK1	0,996570927	0,935191939	1,097702206	0,970534928	1,209240234	0,828441462	1,085465195	0,849875958	1,073186428	1,009241855	0,920012
P52480	KPYM	0,995838267	1,060403038	0,945036261	0,998722434	1,040726619	0,9753886	1,002965961	1,0024014	0,990945513	1,002485619	0,920675
Q9Z1L5	CA2D3	1,128042936	0,943802786	1,053693606	0,874460671	0,922496018	1,049041944	0,856920475	0,989224847	1,227297376	1,008996132	0,920782
Q9CZU6	CISY	1,005545646	1,036407336	0,968680606	0,989366413	1,035145153	0,969666316	1,014005175	1,009891263	0,980638912	1,001869364	0,921855
O70435	PSA3	0,983289896	0,98222047	0,971702446	1,062787189	0,994487257	1,044800655	1,081972288	0,993396483	0,904616023	1,003854541	0,923232
O09131	GSTO1	0,926754151	1,059032016	0,951289542	1,062924291	0,945462064	0,914594113	1,023973606	1,11410385	1,026923014	1,005011329	0,923709
Q8C8N2	SCAI	0,8936166	1,123592434	0,895214585	1,087576381	1,225791242	0,945418409	1,222984421	0,123946087	1,355696341	0,9747673	0,924986
Q9D6J5	NDUB8	1,012622167	1,088950376	0,844004173	1,054423285	0,848952722	0,270491185	1,298731349	1,372939836	1,096785604	0,977580139	0,9252
P84104	SRSF3	0,764143212	1,084670953	1,081787623	1,069398213	0,915973393	0,950395385	1,113663677	0,943835409	1,038338385	0,99244125	0,927921
Q80TB8	VAT1L	1,066385162	1,067644445	0,855315969	1,010654423	0,891589921	0,793230741	1,051349943	1,277048586	0,939308401	0,990505518	0,92959
O08788	DCTN1	0,846381217	1,141262331	1,036692651	0,9756638	1,034623856	1,088742942	0,771146097	1,006141975	1,060853144	0,992301603	0,929745
Q8VD37	SGIP1	0,960664357	1,036143019	0,975489565	1,027703059	1,114589277	0,915946176	0,90364334	0,959311422	1,082860435	0,99527013	0,930114
Q6P9K8	CSKI1	0,933418474	1,043064152	0,970500591	1,053016784	1,129930544	1,016385511	0,966602451	0,883069156	1,027649251	1,004727383	0,930519
Q62318	TIF1B	0,72864094	1,103862555	1,190307035	0,977189469	0,715170547	0,956627783	0,949314235	1,112196598	1,330766363	1,012815105	0,932138
Q9CR68	UCRI	1,165500071	1,056079198	0,720290743	1,058129988	0,894821026	1,488907634	0,94858494	0,976127619	0,763143567	1,014316957	0,933051
Q99NE5	RIMS1	1,006832756	1,024892306	1,071295532	0,896979406	1,287805429	0,994307395	0,86397113	0,904032998	0,988401542	1,007703699	0,934386
Q9CQ62	DECR	1,054616155	0,973540722	1,016194323	0,9556488	0,925832731	1,025888392	1,127048821	1,101458766	0,789073897	0,993860521	0,9353
Q9QYB5	ADDG	0,957688266	1,074148816	1,026640272	0,941522646	0,995572051	1,0159668	0,984037569	0,929160851	1,060078683	0,996963191	0,935719
O55042	SYUA	0,260846975	1,131192572	1,399389998	1,208570454	0,925019518	0,983841657	1,023390274	1,038423419	1,123286113	1,018792196	0,935792
O08919	NUMBL	0,790889908	1,001075927	1,118561489	1,089472675	1,034651626	0,916206006	1,030919694	0,935839803	1,113064071	1,00613624	0,93853
Q2NL51	GSK3A	0,942598568	0,965959	1,120655547	0,970786884	1,030519075	0,943928729	1,057498571	1,017633979	0,967477917	1,003411654	0,938764
P63037	DNJA1	0,911351747	1,100508442	0,97927027	1,008869541	0,896486862	0,934907138	0,85476251	0,988172232	1,369392353	1,008744219	0,939267
Q9DB20	ATPO	1,033513185	0,949650944	1,059506162	0,957329709	0,955783957	0,98702446	0,993521607	1,052843077	1,022742713	1,002383163	0,939898
Q6PHS9	CA2D2	0,536115763	1,202542406	1,100594545	1,160747286	0,867092501	1,036160246	0,615923618	1,0860863	1,321473003	0,985347134	0,94104
O08532	CA2D1	0,846662976	1,044181065	1,099623823	1,009532136	0,965494	1,024799805	0,987047427	0,988500671	1,015571334	0,996282648	0,941967
Q8VHL1	SETD7	1,114202169	0,892808705	1,017453136	0,97553599	1,114919519	0,901539892	1,049865675	1,035059655	0,874334929	0,995143934	0,943391
P35585	AP1M1	0,978301584	1,05443113	0,972766734	0,994500552	1,027118271	0,904132826	1,031456999	0,946453643	1,076698872	0,997172122	0,944425
Q8BIW1	PRUNE	0,963863274	0,961868429	1,1354192	0,938849097	0,798683738	1,013836693	1,077219595	0,958535097	1,124713272	0,994597679	0,944951
Q11011	PSA	0,957175404	1,050022676	1,018008147	0,974793774	1,044588936	0,931492775	0,99061442	0,965357343	1,080674128	1,00254552	0,945035
A2A432	CUL4B	1,433614731	0,024680291	1,303081576	1,238623402	1,174174246	0,877346594	1,071986882	0,844362361	1,137528249	1,021079666	0,945549
Q8CHT0	AL4A1	0,852316911	1,044899352	1,118853711	0,983930025	0,365350754	1,28823849	1,109583239	1,135271929	1,035636022	0,986816087	0,946296
Q8BGN3	ENPP6	0,909182972	1,291525768	0,865960444	0,933330816	1,138424177	0,534499091	1,119036198	1,16367503	1,100209669	1,011168833	0,946545
Q9EQ06	DHB11	1,081880659	1,075814717	0,940542984	0,90176164	1,067810888	1,527171457	0,919691337	0,806218724	0,734810138	1,011140509	0,947956
P80314	TCPB	0,942639428	1,047616627	0,995925722	1,013818223	0,990781756	0,934789833	1,043726391	1,042270705	0,998451433	1,002004024	0,948207
P62245	RS15A	0,926652212	1,099065383	1,005228762	0,969053643	1,065161867	1,020200053	1,134953098	0,928715308	0,871773371	1,004160739	0,948665
O35633	VIAAT	0,922581612	1,126848606	0,950891858	0,999677924	0,906477704	1,048159647	1,02884262	1,057816661	0,941398623	0,996539051	0,949421
Q91VR5	DDX1	0,930113349	1,029107362	1,013738407	1,027040881	1,026816145	0,972494707	0,999420042	0,955469867	1,055270868	1,001894326	0,949868

O55013	TPPC3	1,114975403	0,911520319	0,981104753	0,992399525	0,84138773	1,036253012	1,166270995	0,973322438	1,005149128	1,00447666	0,950795
P62855	RS26	0,442471905	1,234419068	1,232283171	1,090825856	0,881610243	1,013818053	1,099021075	1,143389411	0,804580563	0,988483869	0,95107
P70704	AT8A1	0,86601728	1,099377119	1,05027958	0,984326021	0,970489249	1,016466984	0,929487102	0,979527772	1,088563332	0,996906888	0,955713
Q08331	CALB2	0,726028184	1,153317861	0,996155768	1,124498187	1,031751573	1,061221324	1,089257348	0,993344858	0,85232583	1,005580187	0,956052
P68368	TBA4A	1,068563788	0,9895767	1,019928298	0,921931214	0,785534332	1,149982027	0,877944998	0,98967885	1,171596772	0,994947396	0,956472
P43275	H11	0,823811318	1,025761337	1,017051526	1,133375819	0,874330253	0,913643537	1,213078209	0,985846646	1,037354659	1,004850661	0,957486
P62192	PRS4	0,859843809	1,111377712	1,094743455	0,934035023	1,041086536	0,948903905	1,104373882	0,834244245	1,051157087	0,995953131	0,959187
Q5HZI9	S2551	0,843644415	0,927580506	1,147398532	1,081376548	0,728372883	0,908828691	1,144575423	1,113648917	1,076254709	0,994336125	0,959435
Q9WV18	GABR1	0,983832957	1,125073737	0,826320735	1,064772571	1,225094676	0,511364345	0,912458471	1,412892232	0,889886259	0,990339196	0,959703
Q9WUA2	SYFB	0,925055929	1,08160613	1,059142994	0,934194947	1,091697904	1,047581405	1,021968355	0,851868762	1,002184379	1,003060161	0,959715
P20152	VIME	0,845991413	1,172285299	0,947311996	1,034411292	0,982931766	0,846454185	1,221224207	0,992444037	0,933661447	0,995343128	0,961459
Q91W50	CSDE1	1,031518202	1,054382244	0,894481838	1,019617716	1,004376846	0,887171943	0,988551485	1,034197249	1,098009797	1,002461464	0,962212
Q8BLK3	LSAMP	0,870993355	1,040404532	1,037832517	1,050769597	0,935431758	0,995665995	1,035342527	0,968962364	1,075033519	1,002087233	0,965784
P09405	NUCL	0,937638644	1,034150261	1,054383214	0,97382788	1,031642686	0,924979571	0,975331866	0,961474212	1,116062994	1,001898266	0,967202
O54865	GCYB1	0,929129388	1,126723961	1,143248634	0,800898017	1,086247961	0,736506836	0,968269858	0,958469206	1,225970792	0,995092931	0,967629
O88712	CTBP1	0,965917195	0,990830771	1,031959931	1,011292103	1,081231509	0,922533738	1,018674672	0,931188955	1,038834551	0,998492685	0,968846
Q99L43	CDS2	1,208632606	1,300665314	0,411260725	1,079441355	1,304006846	1,243300286	0,405565925	0,944402968	1,153387303	1,010132666	0,96951
Q80Z24	NEGR1	0,88522595	0,960673693	1,202192041	0,951908316	1,031306577	0,921869852	0,940756968	1,009508029	1,083284039	0,997345093	0,970665
Q9D1G1	RAB1B	0,892625049	1,081015837	1,054787192	0,971571922	1,054208342	1,012923172	0,892419756	1,010437736	1,021562542	0,99831031	0,97338
Q93092	TALDO	0,975356888	1,011758051	0,94744084	1,065444221	1,120841329	1,018518204	1,017019042	0,986589091	0,847821028	0,998157739	0,974018
Q5SQX6	CYFP2	1,021625668	0,965282925	1,052790656	0,96030075	1,040838272	1,022271752	0,952229176	0,92068469	1,058201483	0,998845074	0,975299
Q9CPV4	GLOD4	0,96636888	0,965955919	0,993249853	1,074425348	1,018712614	0,948798774	1,072138287	1,005839203	0,948979743	0,998893724	0,975425
P70404	IDHG1	0,965718622	0,994285771	0,985076817	1,05491879	1,013883587	0,981294298	1,04748987	0,988444029	0,972460076	1,000714372	0,97595
Q9JLJ2	AL9A1	1,019900908	1,025806528	0,969109199	0,985183365	0,925406211	1,091870102	1,082755292	1,020580816	0,886708874	1,001464259	0,976676
Q9QYC0	ADDA	0,917992401	1,094120238	1,013083941	0,97480342	1,026728757	0,930072945	0,970477933	0,974484531	1,105222373	1,001397308	0,977184
P38647	GRP75	0,805517226	1,137711757	1,003567119	1,053203898	1,010969034	1,045459647	0,81301224	1,004992002	1,115000403	0,997886665	0,980653
Q6P9K9	NRX3A	1,002524085	1,076068171	0,947552559	0,973855185	1,100205029	0,956255049	0,977715912	0,937194545	1,033661404	1,001006388	0,981316
P20917	MAG	1,284431626	1,016826542	0,76547697	0,933264862	1,027895508	0,966216551	1,108116839	1,017657118	0,891511017	1,002279407	0,983027
P11627	L1CAM	0,945586099	1,02315313	1,079171695	0,952089076	1,07417163	1,054534593	0,958114668	0,885768902	1,032518329	1,001021624	0,983738
Q9QYG0	NDRG2	1,017090354	1,084899803	0,91852322	0,979486624	0,994890678	1,238681702	0,941315174	0,948457974	0,868798004	0,998428707	0,984508
Q5EBJ4	ERMIN	1,127132218	1,042931019	0,865034872	0,964901891	1,163390594	0,979307761	0,993624999	0,939952283	0,929566881	1,001168504	0,986883
Q9DCJ5	NDUA8	1,101984537	0,797497631	0,96437925	1,136138583	0,902953281	1,138387255	1,075334172	0,90190182	0,988528115	1,001420928	0,987287
Q9CPY7	AMPL	1,032846677	1,045663628	0,922514056	0,998975639	1,0145355	0,99087217	1,059610246	1,024733749	0,90718596	0,999387525	0,987529
Q8VDP4	CCAR2	0,836545682	1,061953872	0,990347989	1,111152457	1,026076321	0,920149523	1,01785196	0,996414963	1,042897218	1,000677997	0,990991
Q8CGK7	GNAL	0,695771961	0,803951401	1,635306339	0,864970298	1,116074306	0,211085851	0,814303106	1,394053894	1,481538881	1,003411208	0,991837
P80318	TCPG	0,926590433	1,040825447	1,020896066	1,011688054	0,99856674	0,928103365	1,033280827	1,046653936	0,991925175	0,999706009	0,992974
P47738	ALDH2	1,048920058	1,073084712	0,920964345	0,957030885	0,934037742	1,008799017	1,044964373	1,006100182	1,004849612	0,999750185	0,994924
Q9CR57	RL14	0,907658171	1,014548668	0,984839787	1,092953373	0,961647389	0,99052158	1,075519808	1,053205622	0,917912446	0,999761369	0,996095
Q6P8X1	SNX6	0,966412209	1,084461935	0,962298364	0,986827492	0,9626083	1,007330925	1,059716667	0,94957775	1,019931079	0,999832944	0,996199
Q9D9V3	ECHD1	1,028702035	1,026698384	0,924610675	1,019988906	1,08733949	1,058583149	1,039927856	0,946165862	0,869214159	1,000246103	0,996276

Q8K0U4	HS12A	1,07799004	1,019119713	0,86427283	1,038617417	1,090545947	0,927494726	0,983646077	1,044606852	0,952445981	0,999747917	0,996364
P62305	RUXE	0,715115664	1,08079334	1,236227485	0,967863512	0,811779877	0,833881929	1,097244349	1,107962409	1,150671582	1,000308029	0,998134
O55234	PSB5	0,900346357	1,023319257	1,041659309	1,034675077	1,004093422	1,011691113	1,091158411	0,994079838	0,899498316	1,00010422	0,998231
P39054	DYN2	0,903462129	1,089918529	0,923442997	1,083176345	1,350149569	1,178922991	1,223915287	1,188161873	0,05574861	0,999379666	0,998252
Q9D8Y0	EFHD2	1,177562947	0,947706558	0,90038841	0,974342085	1,14710328	1,019069345	0,979924534	0,997271564	0,856705346	1,000014814	0,999848
